# iTRAQ-based quantitative proteomic analysis provides insight for molecular mechanism of neuroticism

**DOI:** 10.1186/s12014-019-9259-8

**Published:** 2019-11-08

**Authors:** Lei Tian, Hong-Zhao You, Hao Wu, Yu Wei, Min Zheng, Lei He, Jin-Ying Liu, Shu-Zhen Guo, Yan Zhao, Ren-Lai Zhou, Xingang Hu

**Affiliations:** 1 School of Traditional Chinese Medicine, Beijing University of Chinese Medicine, Beijing, 100029 China; 2 Department of Endocrinology, Fuwai Hospital and National Center for Cardiovascular Diseases, Chinese Academy of Medical Sciences & Peking Union Medical College, Beijing, 100037 China; 3 School of Psychology, Beijing Normal University, Beijing, 100875 China; 4 Beijing University of Chinese Medicine Third Hospital, Beijing, 100029 China

**Keywords:** Neuroticism, Serum, Proteomics, iTRAQ

## Abstract

**Background:**

Neuroticism is a core personality trait and a major risk factor for several mental and physical diseases, particularly in females, who score higher on neuroticism than men, on average. However, a better understanding of the expression profiles of proteins in the circulating blood of different neurotic female populations may help elucidate the intrinsic mechanism of neurotic personality and aid prevention strategies on mental and physical diseases associated with neuroticism.

**Methods:**

In our study, female subjects were screened for inclusion by the Eysenck Personality Questionnaire (EPQ), Beck Depression Inventory (BDI), Beck Anxiety Inventory (BAI) scales and routine physical examination. Subjects who passed the examination and volunteered to participate were grouped by neuroticism using EPQ scores (0 and 1 = low neuroticism group; > 5 = high neuroticism group). Proteins in serum samples of the two neuroticism groups were identified using isobaric tags for relative and absolute quantification (iTRAQ) technology.

**Results:**

A total of 410 proteins exhibited significant differences between high and low neuroticism, 236 proteins were significantly upregulated and 174 proteins were significantly downregulated. Combine the results of GO and KEGG enrichment analysis of differences proteins between high and low neuroticism with the PPI network, it could be observed that the Alpha-synuclein (SNCA), ATP7A protein (ATP7A), Guanine nucleotide-binding protein G(I)/G(S)/G(O) subunit gamma-2 (GNG2), cyclin-dependent kinase 6 (CDK6), myeloperoxidase (MPO), azurocidin (AZU1), Histone H2B type 1-H (HIST1H2BH), Integrin alpha-M (ITGAM) and Matrix metalloproteinase-9 (MMP9) might participate in the intrinsic mechanism of neuroticism by regulating response to catecholamine stimulus, catecholamine metabolic process, limbic system development and transcriptional misregulation in cancer pathway.

**Conclusions:**

Our study revealed the characteristics of the neurotic personality proteome, which might be intrinsic mechanism of the neurotic population.

## Background

Neuroticism is a core personality dimension and is operationally defined as the tendency to experience negative emotions [1]. This trait is considered a measure of emotional instability. Compared with individuals low in neuroticism, high neuroticism individuals tend to show higher reactivity and feel threatened by events, particularly when frustrated or facing loss. Moreover, high neuroticism individuals often adopt maladaptive coping styles, such as avoidance behavior and ineffective escape strategies [2]. Hence, people with higher levels of neuroticism may experience more negative emotions such as fear, anxiety, depression, shame, guilt, and irritability [3]. Previous studies have shown that neuroticism is a major risk factor for various psychiatric disorders, particularly depression and anxiety. In addition, there is evidence that individuals with high neuroticism scores engage in more negative health behaviors, such as poor diet, lack of exercise, alcohol dependence, and substance use [4–7]. Some studies indicate that high neuroticism is associated with greater functional impairment and multimorbidity [8, 9]. More importantly, neuroticism is a potential risk factor for some physical diseases. High neuroticism is not only an independent risk factor for cardiovascular disease, but also closely associated with the morbidity and mortality of chronic diseases such as cancer and diabetes [10, 11]. One prospective study on individuals with chronic renal insufficiency found that patients with high levels of neuroticism had an estimated upper mortality rate of 37.5% [12]. Considering the evidence indicating that neuroticism is an important starting point for understanding many physical and mental diseases, it is necessary to explore the characteristics and internal mechanisms of neuroticism. In particular, women score higher on neuroticism than men, on average, and have a higher incidence of major emotional disorders.

Previous research indicates that proteins, important molecules involved in the regulation of human biology, are involved in neurotic-related negative emotional regulation. For example, proteomic research suggests that the serum levels of c-reaction protein (CRP), inter-alpha-trypsin inhibitor heavy chain H4 (ITIH4), serum amyloid A1 (SAA1), and angiopoietin-like 3 (ANGPTL3) are significantly higher in patients with depression than in healthy controls. Hence, these differentially expressed proteins may represent potential new biomarkers for the clinical diagnosis of depression [13]. Johnson et al. [14] have reported that subjects who experience panic anxiety have substantially higher levels of orexin (ORX) in cerebrospinal fluid compared with participants who do not experience panic anxiety, and that ORX antagonists are a potential novel treatment strategy for panic anxiety. Consequently, proteomic profiling of neuroticism could help to clarify the construct and elucidate the intrinsic mechanism of neurotic personality.

Recently, the rapid development of novel proteomics technology has permitted the identification of the differential expression of proteins among samples in a single experiment. Protein expression between samples can be assessed using isobaric tags for relative and absolute quantification (iTRAQ). This powerful new proteomics technology can reduce the potential variation in numerous mass spectrometry runs and thus enhance the precision of protein identification and quantification. In contrast to traditional proteomics technology, iTRAQ can label all enzymatic peptides and some hydrophobins and membrane proteins. In addition, iTRAQ technology has sensitivity and can accurately grasp the dynamic changes of differential proteins between samples using isotope labeling [15]. Thus, iTRAQ-based relative quantization is a promising and reliable approach. The technology has been used to investigate a wide range of disorders, including depression, cancer, and cardiovascular disease [13, 16, 17]. However, few studies have investigated the proteome characteristics of the neurotic personality, and there is a lack of high-throughput proteomics data support.

Thus, in this study, iTRAQ was used to screen for the differential expression of numerous serum proteins in high and low neurotic individuals. The differentially expressed proteins were analyzed using the bioinformatics tools Gene Ontology (GO) function, Kyoto Encyclopedia of Genes and Genomes (KEGG) pathway enrichment and protein–protein interactions (PPI) network analysis. The study aim was to explore the proteomic characteristics of female neurotic personality and to provide insight into the molecular mechanisms of neurotic personality.

## Materials and methods

### Participants

From September to November 2014, questionnaires were distributed on a class-by-unit basis to female undergraduates at Beijing University of Chinese Medicine who met the following inclusion criteria: (1) no organic disease or chronic illness as determined by a routine hospital physical examination; (2) no significant psychological abnormalities, depression, or anxiety; (3) no major adverse life events in the last 3 months; (4) written informed consent indicating voluntary participation in the study. Our study was conducted in accordance with “the principles of the Declaration of Helsinki” and received approval from the ethics committee of Beijing University of Chinese Medicine (project identification code: 2017BZHYLL0313).

A total of 46 participants were further screened by assessing their neuroticism scores. Criteria for inclusion were high neuroticism scores (scores above 5) or low neuroticism scores (scores of 0 or 1) on the EPQ-RSC. Groups were categorized according to neuroticism following the method used by Zhang [18]. There were 21 participants in the low neuroticism group and 25 in the high neuroticism group. There were significant inter-group differences in neuroticism but not in psychoticism and validity scale scores. There were no significant inter-group differences in age, height, and weight, indicating homogeneity between the groups.

### Sample collection and serum preparation

All participants attended routine medical examinations at the Beijing University of Traditional Chinese Medicine Center for Disease Control and Prevention Center, during which 5 ml of peripheral blood was collected in EDTA-lined tubes (BD Vacutainer ^®^K2 EDTA 10.8 mg REF 367863, BD Franklin Lakes, NJ, USA**)**. The blood was centrifuged at 1200×*g* and 4 °C for 10 min. The resultant serum samples were then stored at − 80 °C for further experimentation.

### Depletion of high abundant proteins from serum preparation

Serum samples were pooled within the high and low neuroticism groups. Large amounts of serum proteins were depleted using an iTRAQ^®^ Reagents-4plex Applications Kit—Protein and a Bio-Rad ProteoMiner Protein Enrichment Large-Capacity Kit (1633007, Bio-Rad laboratories, Hercules, CA, USA). Then, the concentration of each group protein was measured using the Bradford method, and a standard BCA assay (Thermo Scientific Pierce™ BCA Protein Assay Kit). According to the serum protein concentration results, protein normalization was verified using 12% sodium dodecyl sulfate polyacrylamide gel electrophoresis (SDS-PAGE).

### iTRAQ sample labeling and SCX-based fractionation

Before iTRAQ labeling, the protein samples had to be digested. From each of the high and low neuroticism group sample solutions, 100 μl of protein was accurately removed and the protein samples were digested using Trypsin Gold at a protein to enzyme ratio of 20:1 at 37 °C for 4 h. Trypsin Gold in the same ratio was then added to the protein samples and they were digested for 8 h. After protein digestion, the iTRAQ-labeled peptides were vacuum centrifuged to dryness then redissolved with 0.5 M TEAB. iTRAQ labeling of the high and low neuroticism group peptide samples was performed using an iTRAQ Reagent8-plex Kit, according to the manufacturer’s protocol. The peptide samples were then incubated at room temperature for 2 h. All labeled peptides were fractionated using strong cation exchange (SCX).

SCX chromatography was carried out using the Shimadzu LC-20AB HPLC Pump system. The digested peptides samples from the high and low neuroticism groups were reconstituted and acidified with 4 ml buffer A (25 mM NaH2PO4 in 25% ACN, pH 2.7) for further fractionation using strong cation exchange (SCX) chromatography. This procedure was loaded onto a 4.6 × 250 mm Ultramax SCX column comprising 5 μm particles (Phenomenex). The peptide fractions were subsequently eluted at a flow rate of 1 ml/min with a linear gradient of buffer A for 10 min and 5–35% buffer B (25 mM NaH2PO4, 1 M KCI in 25% ACN, pH 2.7) for 11 min or 35–80% buffer B for 1 min. The system was kept in 80% buffer B for 3 min and then equilibrated with buffer A for 10 min before the next injection. The protein concentrations of all eluted samples were monitored by surveying at 214 nm. Fractions were collected every 1 min. Then the eluted peptides were pooled as 20 fractions, desalted with a Strata X C18 column (Phenomenex) and vacuum-dried. All samples were stored at − 80 °C for subsequent LC/MS/MS analysis.

### LC/MS/MS analysis

Every fraction was resuspended in buffer A (5% ACN, 0.1% FA) and then centrifuged at 20,000*g* for 10 min. The supernatant was loaded on a LC-20AD nano HPLC (Shimadzu, Kyoto, Japan) by the autosampler onto a 2 cm C18 trap column. The peptides were subsequently eluted in a 10 cm analytical C18 column packed in-house. Then, peptides from every sample were eluted onto a gradient of phase B (95% ACN, 0.1% FA), starting from 5% and reaching 35% at a total flow rate at 300 nl/min (controlled by IntelliFlow technology) over about 50 min.

Data acquisition was subsequently performed using a TripleTOF 5600 System spectrometer (AB SCIEX, Concord, ON, Canada) equipped with a Nanospray III source (AB SCIEX, Concord, ON, Canada) and a pulled quartz tip as the emitter (New Objectives, Woburn, MA, USA). The MS was operated with an RP ≥ 30,000 FWHM for TOF MS scans. The survey scans were obtained in 250 ms and as many as 30 product ion scans were collected if surpassing a threshold of 120 counts per second (counts/s). The whole cycle time was fixed to 3.3 s. A sweeping collision energy setting of 35 ± 5 eV combined with iTRAQ adjusted for rolling collision energy was suitable for all precursor ions for rolling collision-induced dissociation. Dynamic exclusion was adjusted to 1/2 of peak width (15 s), and the precursor was subsequently refreshed off the exclusion list.

### Statistical analysis

Descriptive statistical analysis of the participant demographic data was conducted using means ± standard deviations (x ± s). The two-sample t-test for independent samples was used to analyze inter-group differences for data that were normally distributed. The Mann–Whitney rank-sum test was used to analyze inter-group differences for data that were not normally distributed. Statistical analyses were performed using SPSS softwarefor Windows (SPSS version 17.0).

### Protein data analysis and bioinformatics

The obtained raw MS data were converted to MGF format using an appropriate tool, and then the exported MGF files were analyzed in the UniProt human database using a local Mascot server. Quality control (QC) was performed to determine whether reanalysis was required. IQuant automated software was used to quantify the proteins. All proteins with a false discovery rate (FDR) under 1% were selected.

Protein identifications were grouped based on the peptide matches from all samples. The protein ratios were calculated according to the median of the unique peptides. Proteins with P-values under 0.05 and fold changes of more than 1.2 or less than 0.83 were deemed significantly regulated. In addition, all peptide ratios were standardized using the median protein ratio, which should be 1 after normalization. Furthermore, we performed in-depth Gene Ontology (GO) annotation and enrichment analysis of the sequence data of selected differentially expressed proteins, the DEPs were imported into Blast2GO software (Version2.7.0) for GO mapping and annotation. The GO project comprises three aspects: biological process, molecular function, and cellular component. Fisher’s exact test was used to detect significant categories, and GO terms with P-values less than 0.05 were deemed significantly enriched. Subsequent pathway annotation and mapping and enrichment analysis were carried out using the Kyoto Encyclopedia of Genes And Genomes (KEGG; http://www.genome.p/kegg/).

In addition, The Search Tool for the Retrieval of Interacting Genes (STRING) database was conducted to analyze PPIs among the DEPs between high and low neuroticism groups. The PPI network results were further analyzed by Cytoscape.

## Results

### Participant demographic characteristics

The average age of the two groups was 23.50 ± 3.46 years. There were no statistically significant inter-group differences in age, height, weight, or body mass index (BMI), suggesting that the two groups had great homogeneity. There were significant inter-group differences in BAI, BDI, and introversion and extraversion scores. This result is consistent with previous studies that found a positive correlation between neuroticism and anxiety, depressive symptoms, and introversion [19]. However, according to the scale cutoff score guidelines, the degree of depression (BDI ≤ 4) and anxiety (BAI ≤ 45) of the two groups was mild and did not meet the clinical diagnostic criteria. Therefore, the experimental results cannot be explained by inter-group differences in depression and anxiety. Table 1 shows detailed demographic information for the two groups.

**Table 1:** Demographic characteristics of high and low neuroticism participants

	All subjects	High neuroticism (4250.6%)	Low neuroticism (4149.4%)	Sig.
Age	23.50 ± 3.46	23.12 ± 3.54	23.5 ± 3.40	0.409
High	162.64 ± 4.68	160.42 ± 4.76	164.10 ± 4.25	0.52
Weight	53.87 ± 6.63	53.84 ± 6.61	53.91 ± 6.81	0.947
BMI	20.35 ± 2.21	20.66 ± 2.33	19.98 ± 2.06	0.487
BAI	33.16 ± 8.46	37.13 ± 9.20	28.45 ± 4.03	0.000**
BDI	4.76 ± 4.48	8.36 ± 2.81	0.476 ± 0.51	0.000**
EPQ-P	48.89 ± 6.63	49.90 ± 6.17	48.88 ± 7.29	0.611
EPQ-E	47.57 ± 10.80	42.98 ± 10.05	51.84 ± 10.29	0.012*
EPQ-N	49.69 ± 14.41	69.17 ± 5.62	34.82 ± 1.64	0.000**
EPQ-L	50.26 ± 9.42	48.37 ± 9.99	52.52 ± 8.36	0.138

* *P* < 0.05; ** *P* < 0.01

### Protein identification and quantification analysis

We used LC/MS/MS combined with iTRAQ-based quantitative proteome technology to investigate differentially expressed proteins in high and low neuroticism participants. In total, 301,242 spectrums were generated, of which 60,148 peptide spectrum matches (PSMs) were assigned to 11,484 peptides (10,285 unique peptides) after 1% FDR was used. These identified peptides correspond to an array of 2389 proteins between high and low neuroticism (Additional file 1: Table S1).

### Differences in protein expression profiles between high and low neuroticism groups

To further screen for proteins with significant inter-group differences, we defined significantly differential proteins for the two groups with a fold difference greater than 1.2 or less than 0.83, a P-value < 0.05, a selected peptide chain > 1, and a confidence interval > 95%. A total of 410 proteins exhibited significant differences (P < 0.05) between high and low neuroticism; 236 proteins were significantly upregulated and 174 proteins were significantly downregulated (Additional file 1: Tables S2, S3).

### GO annotation and enrichment analysis of differentially expressed proteins (DEPs)

To investigate trends in the specific protein functional categories that were annotated in neuroticism, a GO category annotation analysis of the significant differentially expressed proteins was performed. The GO annotation analysis comprises biological processes, molecular functions and cellular components.

**Fig. 1:**
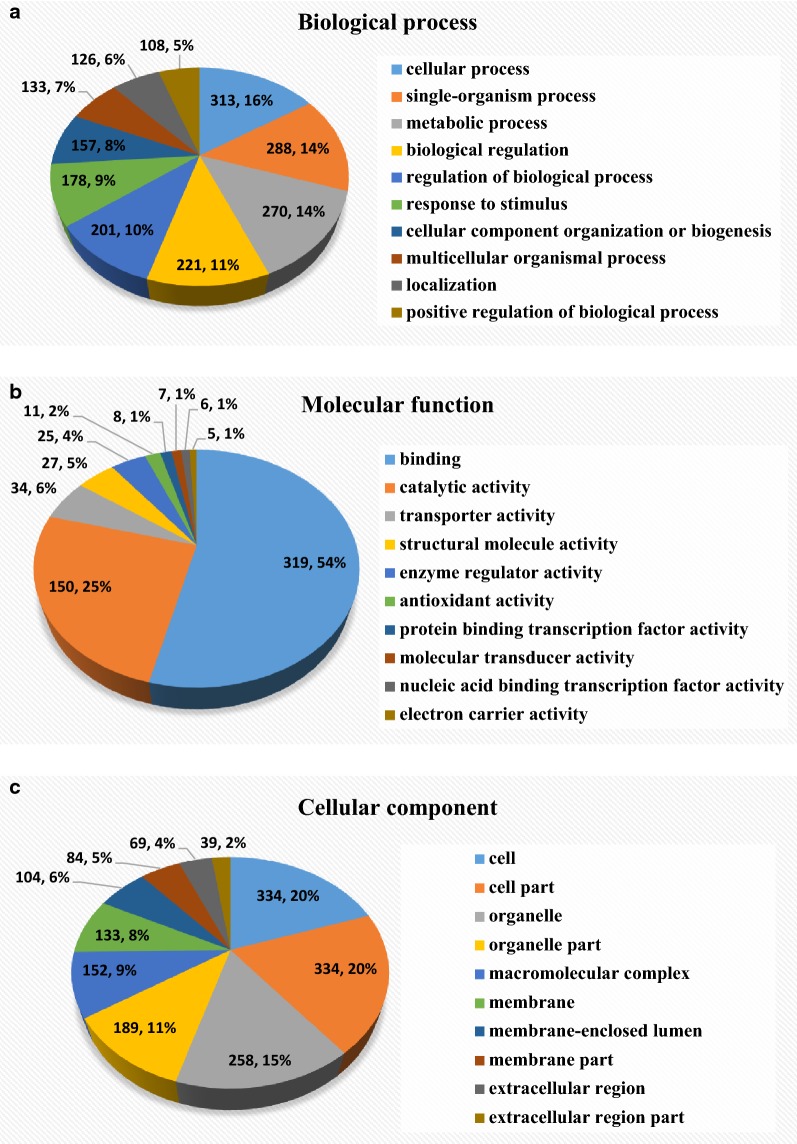
Pie charts of the GO annotation of DEPs with differential abundances in biological process, cellular component and molecular function. **a** Gene Ontology (GO) function categories in Biological process. **b** The Gene Ontology (GO) function categories in Molecular function. **c** Gene Ontology (GO) function categories in Cellular function

As summarized in Fig. 1a, the top three biological processes were cellular process (313.16%), single-organism process (288.14%) and metabolic process (270.14%). The top three molecular function mainly include binding (319.54%), catalytic activity (150.25%) and transporter activity (34.6%) (Fig. 1b). As for cellular component category, cell (334.20%), cell part (334.20%) and organelle (258,15%) were the top three annotation terms (Fig. 1c).

**Fig. 2:**
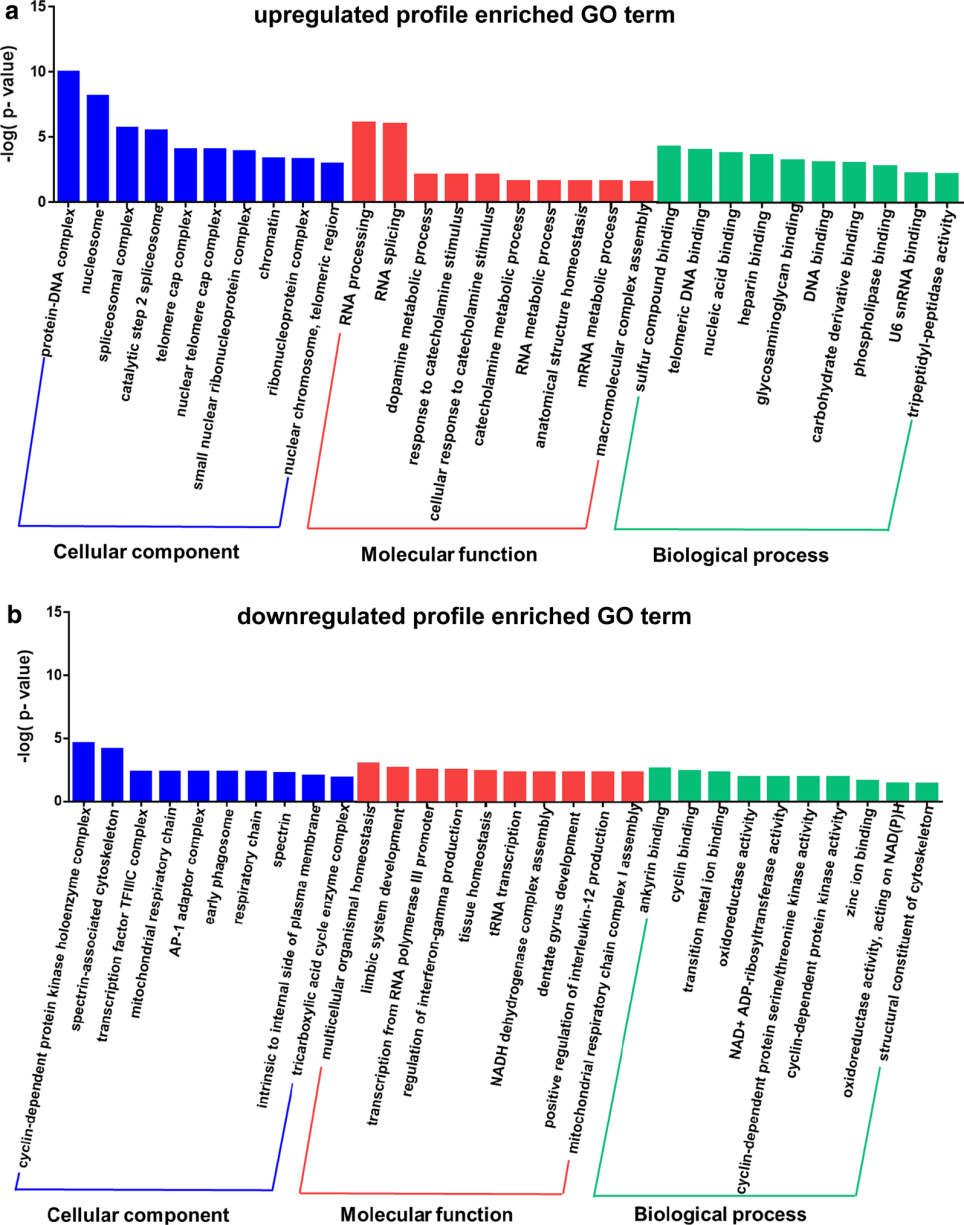
Analysis of differential proteins of high vs. low neuroticism enriched GO terms. **a** The significantly differentially abundant proteins in the upregulated profile of high vs. low neuroticism mainly enriched GO terms. **b** The downregulated profile of high vs. low neuroticism mainly enriched GO terms

To further understand the biological processes participating in neuroticism, differentially expressed proteins in high vs. low neuroticism were enriched to terms using the Fisher exact test. We filtered out categories with P-values less than 0.05 that were significantly enriched GO categories. The significantly differentially abundant proteins in the upregulated profile of high vs. low neuroticism were mainly related to RNA processing, RNA splicing, dopamine metabolic process, response to catecholamine stimulus and catecholamine metabolic process and so on (Fig. 2a). Furthermore, the significantly differentially abundant proteins in the downregulated profile of high vs. low neuroticism were mainly related to multicellular organismal homeostasis, limbic system development, transcription from RNA polymeraseIII promoter, regulation of interferon-gamma production and tissue homeostasis (Fig. 2b). Among them, the limbic system development, response to catecholamine stimulus and catecholamine metabolic process are most related to emotional regulation. Moreover, we found that Guanine nucleotide-binding protein G(I)/G(S)/G(O) subunit gamma-2 (GNG2), ATP7A protein (ATP7A) and Alpha-synuclein (SNCA) were enriched in response to catecholamine stimulus and catecholamine metabolic process. Moreover, the expression of cDNA FLJ56829, highly similar to Neurogenic differentiation factor 6 (B4DS85), cyclin-dependent kinase 6 (CDK6), reelin (RELN) and Nuclear receptor subfamily 0 group B member 1 (NR0B1) were significantly enriched in limbic system development (Table 2).

**Table 2:** Proteins involved in screening of GO enrichment terms

Accession numbers	Gene name	Description	Ratio	P
Response to catecholamine stimulus				
Q4W5L2	SNCA	Alpha-synuclein	1.4	1.30E−05**
P59768	GNG2	Guanine nucleotide-binding protein G(I)/G(S)/G(O) subunit gamma-2	1.35	0.002955**
Catecholamine metabolic process				
Q762B6	ATP7A	ATP7A protein	1.34	0.000539**
Q4W5L2	SNCA	Guanine nucleotide-binding protein G(I)/G(S)/G(O) subunit gamma-2	1.35	0.002955**
Limbic system development				
B4DS85	–	cDNA FLJ56829, highly similar to Neurogenic differentiation factor 6	0.79	0.002573**
Q00534	CDK6	Cyclin-dependent kinase 6	0.77	3.39E−05**
P78509	RELN	Reelin	0.77	0.0001789**
P51843	NR0B1	Nuclear receptor DAX1	0.71	0.03306*

* *P* < 0.05; ** *P* < 0.01

### KEGG pathway enrichment analysis of DEPs

To study the major biochemical metabolic pathways and signal transduction pathways involved in neuroticism, KEGG pathway enrichment analysis was performed on significantly differentially expressed proteins in the two groups.

**Fig. 3:**
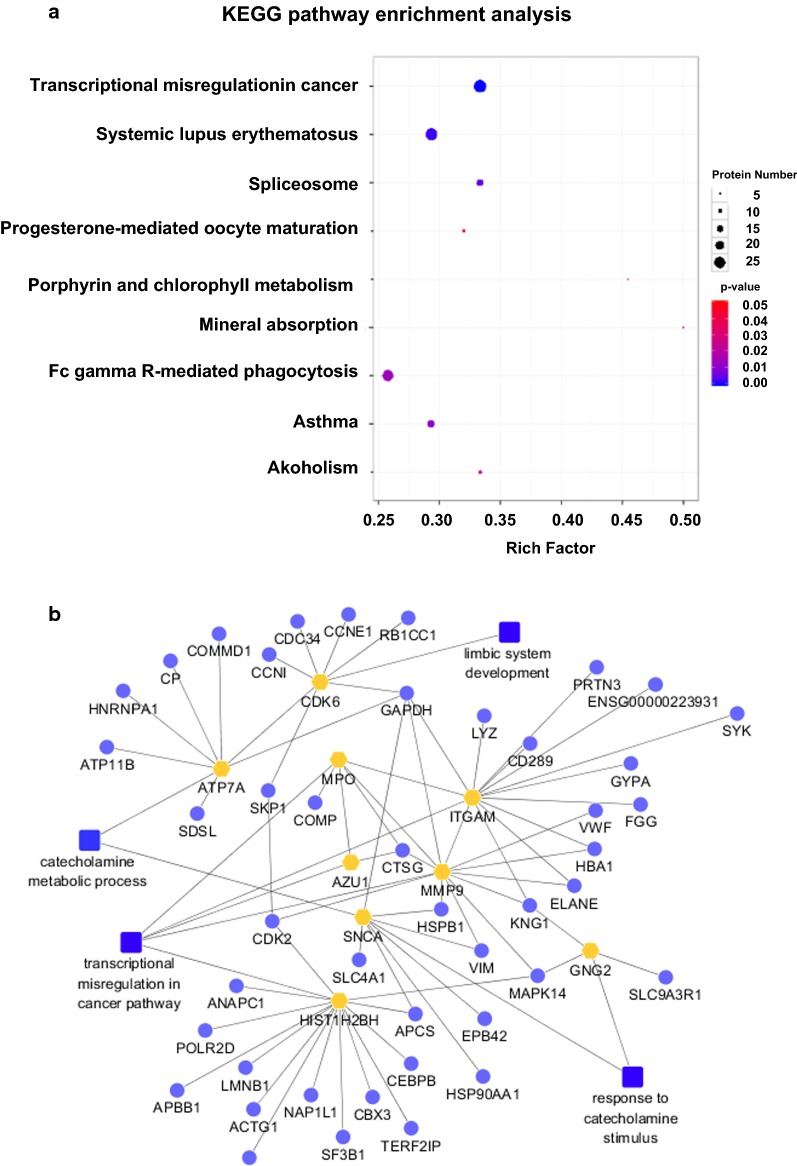
KEGG pathway enrichment and PPI network analysis of differentially aboundant proteins. **a** Statistics of KEGG pathway enrichment of differentially expressed proteins in high vs. low neuroticism. Rich Factor is the ratio of differentially expressed protein number annotated in this pathway term to all protein number annotated in this pathway term. Greater Rich Factor means greater intensiveness. P-value ranges from 0 to 1, and less P-value means greater intensiveness. **b** The protein–protein network model was performed by Cytoscape based on information get from functional analysis, including fold change of protein/gene, protein–protein interaction, mainly GO enrichment terms in biological process and the mostly KEGG enrichment pathway

KEGG enrichment analysis indicated that, in the high neuroticism group compared with the low neuroticism group, the pathways transcriptional misregulation in cancer (P = 0.0001), systemic lupus erythematosus (P = 0.00148), spliceosome (P = 0.0046), asthma (P = 0.0112), Fc gamma R-mediated phagocytosis (P = 0.0138), mineral absorption (P = 0.0156), porphyrin and chlorophyll metabolism (P = 0.0247), alcoholism (P = 0.0264) and progesterone-mediated oocyte maturation (P = 0.045) were significantly enriched. Transcriptional misregulation in cancer was one of the most abundantly enriched pathways (Fig. 3a). A total of 28 significant differential proteins were included in this pathway. Such as the expression of integrin alpha-M (ITGAM), azurocidin (AZU1), histone H2B type 1-O (HIST1H2BH), histone H2B type 1-O (HIST1H2BO), myeloperoxidase (MPO), matrix metalloproteinase-9 (MMP9), Immunoglobulin heavy constant delta (IGHD) and NF-IL6 (CEBPB) were significantly upregulated; Ubiquitously transcribed tetratricopeptide repeat protein Y-linked transcript variant 166 (UTY), Protein PML (PML) and CCAAT/enhancer-binding protein epsilon (CEBPE) were significantly downregulated (Table 3).

**Table 3:** Proteins involved in transcriptional misregulation in cancer

Accession numbers	Gene name	Description	Ratio	*P*
P11215	ITGAM	Integrin alpha-M	1.36	0.004657**
P20160	AZU1	Azurocidin	1.83	1.11E−08**
K7EMV3	H3F3B	Histone H3	2.17	5.39E−08**
Q569I7	–	Uncharacterized protein	1.31	0.005027**
A2N0T3	VH6DJ	VH6DJ protein	1.4	0.01061**
A2J1M8	–	Rheumatoid factor RF-IP12	0.79	0.016**
Q6ZRN6	–	cDNA FLJ46220 fis, clone TESTI4013774	0.72	0.001376**
Q6GMX6	IGH@	IGH@ protein	0.75	8.17E−05**
A2JA14	–	Anti-mucin1 heavy chain variable region	0.68	1.04E−08**
Q5TEC6	HIST2H3PS2	Histone H3	2.13	4.72E−06**
Q0ZCH4	–	Immunglobulin heavy chain variable region	1.26	0.0008512**
P05164	MPO	Myeloperoxidase	1.74	3.18E−10**
A2J1N6	–	Rheumatoid factor RF-ET9	1.23	0.03918**
B2MUD5	ELA2	Neutrophil elastase	2.05	3.34E−09**
Q15744	CEBPE	CCAAT/enhancer-binding protein epsilon	0.79	5.47E−09**
P04438	–	Ig heavy chain V-II region SESS	1.68	2.01E−05**
P14780	MMP9	Matrix metalloproteinase-9	1.87	1.61E−07**
Q93079	HIST1H2BH	Histone H2B type 1-H	4.31	3.00E−14**
S6BGD4	–	IgG H chain	1.37	0.0001422**
P01880	IGHD	Ig delta chain C region	1.25	0.0003251**
A0A068LKQ2	–	Ig heavy chain variable region	0.78	0.001062**
P23527	HIST1H2BO	Histone H2B type 1-O	3.29	7.91E−16**
F4MH86	UTY	Ubiquitously transcribed tetratricopeptide repeat protein Y-linked transcript variant 166	0.82	0.0282**
S6B2B6	–	IgG H chain	1.21	0.02172**
P59666	DEFA3	Defensin, alpha3, neutrophil-specific	2.99	4.69E−05**
P01768	–	Ig heavy chain V-III region CAM	0.81	0.001137**
H3BT57	PML	Protein PML	0.73	0.000317**
Q99557	–	NF-IL6	1.64	0.006268**

* P < 0.05; ** *P* < 0.01

### Protein–protein interactions (PPI)

For purpose of further elucidate molecular mechanism of neuroticism, the protein–protein network was performed by the publicly available program STRING. The 410 DEPs between high and low neuroticism and the proteins enriched in response to catecholamine stimulus, catecholamine metabolic process, limbic system development and transcriptional misregulation in cancer pathway were analyzed by this molecular interaction tool. The results of those PPI network contained 20 notes. The mainly high-degree hub nodes including SNCA, ATP7A, GNG2, CDK6, MPO, AZU1, HIST1H2BH, ITGAM and MMP9. Those high-degree hub node proteins might play an crucial role in regulating the intrinsic mechanism of neuroticism (Fig. 3b).

## Discussion

Neuroticism is one of the most widely accepted personality traits and a core component of many personality theories and models. Research confirms that neuroticism is a robust risk marker for a wide range of psychiatric disorders, particularly internalizing disorders [20]. In this study, iTRAQ was first used to screen for differentially expressed serum proteins in the high and low neuroticism groups and was combined with bioinformatics analysis to explore the molecular mechanisms of neuroticism. From the results of bioinformatics analysis of differences in proteins between high and low neuroticism, we found that SNCA, ATP7A, GNG2, CDK6, MPO, AZU1, HIST1H2BH, ITGAM and MMP9 might participate in the intrinsic mechanism of neuroticism by regulating response to catecholamine stimulus, catecholamine metabolic process, limbic system development and transcriptional misregulation in cancer pathway.

### Proteins involved in response to catecholamine stimulus and catecholamine metabolic process

Catecholamine is a monoamine neurotransmitter and part of a group of substances containing catechol or 3,4-dihydroxyphenyl and amidogen. Catecholamine neurotransmitters are widely involved in regulating physiological and behavioral functions, including mood, cognition, aggressive behavior, motor activity, and sleep arousal [21, 22]. The catecholamine dopamine (DA) regulates many critical functions, such as movement, emotion, and cognition [23, 24]. Research using positron emission tomography demonstrates that pain, stress, metabolic stress, and psychological stress can induce dopamine release [25–27]. Previous studies also suggest that individual differences in personality might play a major role in the response to stress; individuals with high neuroticism show a significantly blunted DA response to stress [28]. Zhao et al. [29] found significant downregulation of norepinephrine in a mouse model of depression-like symptoms and showed that antidepressant drugs can improve depressive symptoms by upregulating norepinephrine. In summary, current research mostly uses animal models, and a correlation between catecholamines and neuroticism in humans has rarely been reported. Our study is the first to show that, compared with individuals with low neuroticism, those with high neuroticism had significantly upregulated proteins in the response to catecholamine stimulus and more vigorous metabolism of catecholamine neurotransmitters. Based on these results, we suggest the following. As high neuroticism individuals tend to show greater susceptibility to stress compared with low neuroticism individuals, high neuroticism individuals release more catechol neurotransmitters such as dopamine and norepinephrine when faced with stress, and the increased catecholamine neurotransmitter level compensates for the stress-related activation of the catecholamine neurotransmitter metabolic pathway. Therefore, we suggest that the regulation of response to catecholamine stimulus and catecholamine metabolism-related pathways may be an important intrinsic mechanism underlying the development of neuroticism. Furthermore, we found that GNG2, ATP7A and SNCA were significantly upregulated in high vs. low neuroticism and enriched in the pathways related to response to catecholamine stimulus. In addition, GNG2, ATP7A and SNCA also as high-degree hub nodes in PPI network, are involved as a modulator or transducer in adenylate cyclase-activating dopamine receptor signaling and the cellular response to catecholamine stimulus systems. We suggest that the GNG2, ATP7A and SNCA participate in the intrinsic mechanism of neuroticism by regulating response to catecholamine stimulus and catecholamine metabolic process pathways.

### Proteins involved in limbic system development

The limbic system includes subcortical regions (comprising the hypothalamus and amygdala) and cortical regions (comprising the hippocampal formation and regions of the neocortex, including the insular cortex, cingulate gyrus, and parahippocampal gyrus) [30]. The limbic system is implicated in regulating emotions and behavioral responses. Previous studies indicate that limbic system dysfunctions are associated with the etiology of mental disorders such as depression and posttraumatic stress disorder [31, 32]. In 1937, Papez made the following landmark observation about the limbic system: “The hypothalamus, the anterior thalamic nucleus, the cingulate gyrus, the hippocampus and their interconnections, constitute a harmonious mechanism which may elaborate the functions of central emotion as well as participate in the emotional expression.” [33]. In recent years, some studies on neurotic personality mechanisms have focused on the close relationship between neuroticism and the limbic system. However, neuroticism is the tendency to experience negative emotions; the amygdala in the limbic system is the hub of responses to negative stimuli (particularly threat information) processing. Recent studies show consistently that neuroticism is associated with enhanced amygdala activity [34–36]. Based on previous study findings, Ormel et al. [1] proposed that neuroticism may be mediated by enhanced activation of an arousal pathway that includes the right amygdala and the medial prefrontal cortex (PFC). Subsequent studies based on fMRI and diffusion tensor imaging have confirmed this hypothesis [37]. In addition to its connections with limbic system dysfunction, neuroticism is associated with reduced integrity of multiple nerve fiber bundles and marginal-frontal loop structures [38, 39]. In the present study, proteins in neurotic females were significantly downregulated in the limbic system development pathway. This finding is consistent with the results of previous studies showing that the limbic system loop structure is incomplete in neuroticism. Moreover, we found B4DS85, CDK6, RELN and NR0B1 were significantly enriched in limbic system development. Additionally, we found that CDK6 was high-degree hub node in PPI network. Caron et al. [40] found that CDK6 controls the proliferation of hippocampal progenitors and that CDK6 kinase activity is regulated by p27. Therefore, we further suggest that the abnormal expression of CDK6 affects the structure and function of the limbic system in neuroticism.

### Proteins involved in transcriptional misregulation in cancer

Neuroticism as a crucial marker of vulnerability for mental as well as physical disorder. Inflammatory markers have been confirmed predict similar mental and physical disease as neuroticism. Previous studies demonstrated that neuroticism have been related to the elevated serum levels of C-reactive protein (CRP), interleukin (IL)-6 and higher leukocyte counts [41–43]. The latest findings also illustrated that high neuroticism was associated with an up-regulation of inflammatory agents. Neuroticism may promote the production of pro-inflammatory messenger molecules which are related to the development of depression [44]. Chronic inflammation is known to accelerate and deteriorate malignancy [45]. Previous studies have shown that personality traits are not only a sign of various psychiatric disorders, but are also closely related to cancer, particularly female-specific cancer. Neuroticism is the personality trait most associated with breast cancer and is also a major risk factor for breast cancer survival [46]. Additionally, some studies indicate that higher levels of neuroticism enhance the risk of depression and anxiety in patients with multiple cancer types. Similarly, among patients with lung cancer, neuroticism increases the risk of depression and anxiety, and is particularly associated with higher levels of anxiety in female patients [47]. Our study found that the expression of ITGAM, AZU1 MPO, MMP9, HIST1H2BH, HIST1H2BO, IGHD and CEBPB were significantly upregulated, UTY, PML and CEBPE were significantly downregulated. Among them the ITGAM, AZU1, MPO, MMP9 and HIST1H2BH were high-degree hub node in PPI network. These proteins, which are involved in the regulation of inflammatory and immune responses, show significantly abnormal expression levels in neuroticism, leading to a significant upregulation of transcriptional misregulation in cancer pathways. We hypothesize that inflammation lead to immune system dysfunction causes abnormalities in the transcriptional misregulation in cancer pathway, which may be a major factor in the association between neuroticism and susceptibility to some cancer. However, our study is based on the iTRAQ technique to confirm the abnormal transcriptional regulation of cancer and inflammation in neuroticism, the relationship between neuroticism and the transcriptional misregulation in cancer pathway (and the abnormal expression of inflammation and immune-related proteins in this pathway from this study) requires further study.

## Conclusions

In this study, we initially revealed the characteristics of the neurotic personality proteome. iTRAQ was used to identified the characterize differentially expressed proteins between high and low neuroticism, which found that SNCA, ATP7A, GNG2, CDK6, MPO, AZU1, HIST1H2BH, ITGAM and MMP9 might participate in the intrinsic mechanism of neuroticism by regulating response to catecholamine stimulus, catecholamine metabolic process, limbic system development and transcriptional misregulation in cancer pathway. Our study revealed the characteristics of the neurotic personality proteome, which might be intrinsic mechanism of the neurotic population and could contribute to better diagnosis and treatment of several physical and mental disorders related to neurotic personality.

### Limitations

This study had several limitations. First, the sample size was small owing to limited funds. The generalizability of the proteomic specificity to a larger population is limited and studies using large sample sizes are needed for verification. Second, this study selected females with more sensitive emotional responses and generally higher neuroticism scores. This also limits the application of our findings to a larger neurotic population. Third, the iTRAQ method used is a semi-quantitative technology. Accurate absolute quantitation is required to determine differences in the levels of individual proteins. Furthermore, the intrinsic mechanism explored in this study and the significantly differential proteins identified here await further study and validation.

## Supplementary information

**Additional file 1: Table S1.** The list of differentially expressed protein between high and low neuroticism by iTRAQ analysis. **Table S2.** The list of up-regulated significant differentially expressed protein between high and low neuroticism by iTRAQ analysis. **Table S3.** The list of down-regulated significant differentially expressed protein between high and low neuroticism by iTRAQ analysis.

## Data Availability

The datasets used or analysed during the current study are available from the corresponding author on reasonable request.

## Abbreviations

EPQ: Eysenck Personality Questionnaire
BDI: Beck Depression Inventory
BAI: Beck Anxiety Inventory
iTRAQ: isobaric tag for relative and absolute quantitation
CRP: c-reaction protein
ITIH4: inter-alpha-trypsin inhibitor heavy chain H4
SAA1: serum amyloid A1
ANGPTL3: angiopoietin-like 3
ORX: orexin
GO: Gene Ontology
KEGG: Kyoto Encyclopedia of Genes and Genomes
LC–MS/MS: liquid chromatography–tandem mass spectrometry
FDR: false discovery rate
SCX: strong cation exchange
SDS-PAGE: sodium dodecyl sulfate polyacrylamide gel electrophoresis
QC: quality control
PSM: peptide spectrum matches
DEPs: differentially expressed proteins
GNG2: G(I)/G(S)/G(O) subunit gamma-2
SNCA: Alpha-synuclein
CDK6: cyclin-dependent kinase 6
RELN: reelin
NR0B1: nuclear receptor subfamily 0 group B member 1
ITGAM: integrin alpha-M
AZU1: azurocidin
HIST1H2BH: histone H2B type 1-O
HIST1H2BO: histone H2B type 1-O
MPO: myeloperoxidase
MMP9: matrix metalloproteinase-9
IGHD: immunoglobulin heavy constant delta
CEBPB: NF-IL6
UTY: ubiquitously transcribed tetratricopeptide repeat protein Y-linked transcript variant 166
PML: protein PML
CEBPE: CCAAT/enhancer-binding protein epsilon

## References

[1] Ormel J, Bastiaansen A, Riese H (2013). The biological and psychological basis of neuroticism: current status and future directions. Neurosci Biobehav Rev.

[2] Canli T (2008). Toward a neurogenetic theory of neuroticism. Ann N Y Acad Sci.

[3] Kotov R, Gamez W, Schmidt F (2010). Linking “big” personality traits to anxiety, depressive, and substance use disorders: a meta-analysis. Psychol Bull.

[4] De Moor MH, Beem AL, Stubbe JH (2006). Regular exercise, anxiety, depression and personality: a population-based study. Prev Med.

[5] Hakulinen C, Hintsanen M, Munafò MR (2015). Personality and smoking: individual-participant meta-analysis of nine cohort studies. Addiction (Abingdon, England)..

[6] Keller C, Siegrist M (2015). Does personality influence eating styles and food choices? Direct and indirect effects. Appetite.

[7] Kuntsche E, Von Fischer M, Gmel G (2008). Personality factors and alcohol use: a mediator analysis of drinking motives. Pers Individ Differ.

[8] Hoerger M, Coletta M, Sorensen S (2016). Personality and perceived health in spousal caregivers of patients with lung cancer: the roles of neuroticism and extraversion. J Aging Res.

[9] Chapman BP, Lyness JM, Duberstein P (2007). Personality and medical illness burden among older adults in primary care. Psychosom Med.

[10] Gale CR, Čukić I, Batty GD (2017). When is higher neuroticism protective against death?. Findings from UK biobank. Psychol Sci.

[11] Puig-Perez S, Villada C, Pulopulos MM (2016). How are neuroticism and depression related to the psychophysiological stress response to acute stress in healthy older people?. Physiol Behav.

[12] Christensen AJ, Ehlers SL, Wiebe JS (2002). Patient personality and mortality: a 4-year prospective examination of chronic renal insufficiency. Health Psychol.

[13] Wang Q, Su X, Jiang X (2016). iTRAQ technology-based identification of human peripheral serum proteins associated with depression. Neuroscience.

[14] Johnson PL, Truitt W, Fitz SD (2010). A key role for orexin in panic anxiety. Nat Med.

[15] Liu T, Hu J, Li H (2009). iTRAQ-based shotgun neuroproteomics. Methods Mol Biol (Clifton, NJ)..

[16] Sharma R, Gowda H, Chavan S (2015). Proteomic signature of endothelial dysfunction identified in the serum of acute ischemic stroke patients by the iTRAQ-based LC-MS approach. J Proteome Res.

[17] Yi Wang, Zhao Yu, Wei Jiang (2018). iTRAQ-based proteomic analysis reveals recovery of impaired mitochondrial function in ischemic myocardium by Shenmai formula. J Proteome Res.

[18] Zhang W, Zhou R, Wang Q (2013). Sensitivity of the late positive potentials evoked by emotional pictures to neuroticism during the menstrual cycle. Neurosci Lett.

[19] Chang CC, Chang HA, Fang WH (2017). Gender-specific association between serotonin transporter polymorphisms (5-HTTLPR and rs25531) and neuroticism, anxiety and depression in well-defined healthy Han Chinese. J Affect Disord.

[20] Lahey BB (2009). Public health significance of neuroticism. Am Psychol.

[21] Jayanthi LD, Ramamoorthy S (2005). Regulation of monoamine transporters: influence of psychostimulants and therapeutic antidepressants. AAPS J.

[22] Mechan AO, Fowler A, Seifert N (2011). Monoamine reuptake inhibition and mood-enhancing potential of a specified oregano extract. Br J Nutr.

[23] Carlsson A, Waters N, Holm-Waters S (2001). Interactions between monoamines, glutamate, and GABA in schizophrenia: new evidence. Annu Rev Pharmacol Toxicol.

[24] Greengard P (2001). The neurobiology of slow synaptic transmission. Science (New York, NY).

[25] Scott DJ, Heitzeg MM, Koeppe RA (2006). Variations in the human pain stress experience mediated by ventral and dorsal basal ganglia dopamine activity. J Neurosci.

[26] Pruessner JC, Champagne F, Meaney MJ (2004). Dopamine release in response to a psychological stress in humans and its relationship to early life maternal care: a positron emission tomography study using [11C]raclopride. J Neurosci.

[27] Soliman A, O’Driscoll GA, Pruessner J (2008). Stress-induced dopamine release in humans at risk of psychosis: a [11C]raclopride PET study. Neuropsychopharmacology.

[28] Suridjan I, Boileau I, Bagby M (2012). Dopamine response to psychosocial stress in humans and its relationship to individual differences in personality traits. J Psychiatric Res.

[29] Zhao J, Niu C, Wang J (2018). The depressive-like behaviors of chronic unpredictable mild stress-treated mice, ameliorated by Tibetan medicine Zuotai: involvement in the hypothalamic-pituitary-adrenal (HPA) axis pathway. Neuropsychiatr Dis Treat.

[30] Braun K (2011). The prefrontal-limbic system: development, neuroanatomy, function, and implications for socioemotional development. Clin Perinatol.

[31] George Bush (2011). Cingulate, frontal, and parietal cortical dysfunction in attention-deficit/hyperactivity disorder. Biol Psychiat.

[32] Bock J, Braun K (2011). The impact of perinatal stress on the functional maturation of prefronto-cortical synaptic circuits: implications for the pathophysiology of ADHD?. Prog Brain Res.

[33] Papez JW (1995). A proposed mechanism of emotion. J Neuropsychiatry Clin Neurosci.

[34] Carolin Brück, Benjamin Kreifelts, Evangelia Kaza (2011). Impact of personality on the cerebral processing of emotional prosody. NeuroImage..

[35] Chan SW, Norbury R, Goodwin GM (2009). Risk for depression and neural responses to fearful facial expressions of emotion. Br J Psychiatry.

[36] Cunningham WA, Arbuckle NL, Jahn A (2010). Aspects of neuroticism and the amygdala: chronic tuning from motivational styles. Neuropsychologia.

[37] Cremers HR, Demenescu LR, Aleman A (2010). Neuroticism modulates amygdala-prefrontal connectivity in response to negative emotional facial expressions. NeuroImage.

[38] Bjørnebekk A, Fjell AM, Walhovd KB (2013). Neuronal correlates of the five factor model (FFM) of human personality: Multimodal imaging in a large healthy sample. NeuroImage.

[39] Xu J, Potenza MN (2012). White matter integrity and five-factor personality measures in healthy adults. NeuroImage.

[40] Caron N, Genin EC, Marlier Q (2018). Proliferation of hippocampal progenitors relies on p27-dependent regulation of Cdk6 kinase activity. Cell Mol Life Sci.

[41] McManus DD, Beaulieu LM, Mick E (2013). Relationship among circulating inflammatory proteins, platelet gene expression, and cardiovascular risk. Arterioscler Thromb Vasc Biol.

[42] Turiano NA, Mroczek DK, Moynihan J (2013). Big 5 personality traits and interleukin-6: evidence for ‘healthy neuroticism’ in a US population sample. Brain Behav Immun.

[43] Sutin AR, Terracciano A, Deiana B (2010). High neuroticism and low conscientiousness are associated with interleukin-6. Psychol Med.

[44] Schmidt FM, Sander C, Minkwitz J (2018). Serum markers of inflammation mediate the positive association between neuroticism and depression. Front Psychiatry.

[45] Bishayee A (2014). The role of inflammation and liver cancer. Adv Exp Med Biol..

[46] Minami Y, Hosokawa T, Nakaya N (2015). Personality and breast cancer risk and survival: the Miyagi cohort study. Breast Cancer Res Treat.

[47] Ken Shimizu, Naoki Nakaya, Kumi Saito-Nakaya (2015). Personality traits and coping styles explain anxiety in lung cancer patients to a greater extent than other factors. Jpn J Clin Oncol.

