# Supplementary material for: iTRAQ-based quantitative proteomic analysis provides insight for molecular mechanism of neuroticism
Source: Clin Proteomics. 2019 Nov 8;16:38. doi: 10.1186/s12014-019-9259-8 (PMC6839193; doi:10.1186/s12014-019-9259-8)
Supplement: Supplementary file 1 — Additional file 1: Table S1. The list of differentially expressed protein between high and low neuroticism by iTRAQ analysis. Table S2. The list of up-regulated significant differentially expressed protein between high and low neuroticism by iTRAQ analysis. Table S3. The list of down-regulated significant differentially expressed protein between high and low neuroticism by iTRAQ analysis. [file 12014_2019_9259_MOESM1_ESM.doc]

**Table S1 The list of differentially expressed protein between high and low neuroticism by iTRAQ analysis**

| **No.** | **Protein_ID** |  | **Uniq_Pep_Num** | **Ratio_HIGH-VS-LOW** | **SD_HIGH-VS-LOW** | **Quant_Num_HIGH-VS-LOW** | **Pvalue_HIGH-VS-LOW** |
| --- | --- | --- | --- | --- | --- | --- | --- |
| **1** | sp|Q9Y5P6|GMPPB_HUMAN |  | 3 | 0.98 | 0.005 | 16 | 0.35 |
| **2** | tr|E9PQN4|E9PQN4_HUMAN |  | 1 | 0.92 | 0.108 | 16 | 0.3428 |
| **3** | tr|V9H0D6|V9H0D6_HUMAN |  | 1 | 1.34 | 0.377 | 16 | 0.04492 |
| **4** | tr|B8ZZY2|B8ZZY2_HUMAN |  | 4 | 0.91 | 0.019 | 16 | 0.0186 |
| **5** | sp|P84085|ARF5_HUMAN |  | 1 | 1.12 | 0.115 | 16 | 0.1879 |
| **6** | sp|P19784|CSK22_HUMAN |  | 4 | 0.92 | 0.01 | 16 | 0.004545 |
| **7** | tr|F5GY03|F5GY03_HUMAN |  | 3 | 0.92 | 0.007 | 16 | 0.002541 |
| **8** | sp|P18428|LBP_HUMAN |  | 10 | 1.02 | 0.051 | 16 | 0.7873 |
| **9** | tr|Q53T09|Q53T09_HUMAN |  | 1 | 1.55 | 0.043 | 16 | 2.17E-08 |
| **10** | tr|Q9P043|Q9P043_HUMAN |  | 1 | 1.03 | 0.014 | 16 | 0.2644 |
| **11** | sp|P62333|PRS10_HUMAN |  | 12 | 0.9 | 0.003 | 16 | 7.89E-06 |
| **12** | sp|Q8TBN0|R3GEF_HUMAN |  | 1 | 0.85 | 0.035 | 16 | 0.007208 |
| **13** | sp|Q00169|PIPNA_HUMAN |  | 6 | 1.21 | 0.065 | 16 | 0.005594 |
| **14** | tr|W8QEY1|W8QEY1_HUMAN |  | 2 | 2.23 | 0.54 | 16 | 7.01E-06 |
| **15** | sp|Q99460|PSMD1_HUMAN |  | 33 | 0.98 | 0.002 | 16 | 0.07663 |
| **16** | tr|B3KRR4|B3KRR4_HUMAN |  | 2 | 0.88 | 0.008 | 16 | 8.41E-05 |
| **17** | tr|E5RJT0|E5RJT0_HUMAN |  | 1 | 1.1 | 0.024 | 16 | 0.02297 |
| **18** | tr|F8W1N9|F8W1N9_HUMAN |  | 1 | 1.23 | 1.117 | 16 | 0.4044 |
| **19** | tr|D6W5C0|D6W5C0_HUMAN |  | 8 | 1.22 | 0.013 | 16 | 9.85E-07 |
| **20** | tr|B4DXW9|B4DXW9_HUMAN |  | 1 | 0.99 | 0.016 | 16 | 0.8565 |
| **21** | sp|P01611|KV119_HUMAN |  | 1 | 0.85 | 0.022 | 16 | 0.001295 |
| **22** | sp|P49006|MRP_HUMAN |  | 1 | 1.02 | 0.017 | 16 | 0.6256 |
| **23** | tr|K7ERI9|K7ERI9_HUMAN |  | 4 | 1.14 | 0.011 | 16 | 0.0001015 |
| **24** | tr|E9PJU8|E9PJU8_HUMAN |  | 1 | 0.96 | 0.105 | 16 | 0.6079 |
| **25** | tr|X5D767|X5D767_HUMAN |  | 5 | 0.83 | 0.015 | 16 | 6.50E-05 |
| **26** | tr|E7ESU0|E7ESU0_HUMAN |  | 6 | 1 | 0.015 | 16 | 0.9083 |
| **27** | tr|A6NKB8|A6NKB8_HUMAN |  | 1 | 1.09 | 0.126 | 16 | 0.3326 |
| **28** | tr|E9PI77|E9PI77_HUMAN |  | 1 | 1.11 | 0.005 | 16 | 3.78E-05 |
| **29** | tr|B7ZKK0|B7ZKK0_HUMAN |  | 1 | 1.02 | 0.015 | 16 | 0.6146 |
| **30** | tr|F8WDS9|F8WDS9_HUMAN |  | 2 | 1.08 | 0.01 | 16 | 0.008276 |
| **31** | sp|P02741|CRP_HUMAN |  | 4 | 1.26 | 0.744 | 16 | 0.2554 |
| **32** | sp|Q92905|CSN5_HUMAN |  | 10 | 0.98 | 0.003 | 16 | 0.1962 |
| **33** | sp|Q86YS7|C2CD5_HUMAN |  | 6 | 1.08 | 0.005 | 16 | 0.0001791 |
| **34** | tr|H3BP78|H3BP78_HUMAN |  | 1 | 0.97 | 0.061 | 16 | 0.6851 |
| **35** | sp|O43747|AP1G1_HUMAN |  | 2 | 1.12 | 0.036 | 16 | 0.02616 |
| **36** | sp|Q14520|HABP2_HUMAN |  | 5 | 0.93 | 0.008 | 16 | 0.005609 |
| **37** | sp|Q9H2M9|RBGPR_HUMAN |  | 11 | 0.95 | 0.004 | 16 | 0.007551 |
| **38** | tr|Q6FHU0|Q6FHU0_HUMAN |  | 2 | 0.98 | 0.024 | 16 | 0.6269 |
| **39** | tr|Q05D78|Q05D78_HUMAN |  | 5 | 1.05 | 0.012 | 16 | 0.07223 |
| **40** | tr|H0YAS9|H0YAS9_HUMAN |  | 1 | 1.01 | 0.077 | 16 | 0.8881 |
| **41** | sp|P35579|MYH9_HUMAN |  | 70 | 1.07 | 0.005 | 16 | 0.0007837 |
| **42** | sp|Q14258|TRI25_HUMAN |  | 6 | 1.01 | 0.012 | 16 | 0.7344 |
| **43** | tr|C9JIK8|C9JIK8_HUMAN |  | 2 | 0.99 | 0.016 | 16 | 0.736 |
| **44** | tr|D3DUP2|D3DUP2_HUMAN |  | 14 | 0.92 | 0.04 | 16 | 0.1466 |
| **45** | tr|B3KV64|B3KV64_HUMAN |  | 2 | 0.93 | 0.021 | 16 | 0.0837 |
| **46** | tr|H0YC20|H0YC20_HUMAN |  | 1 | 0.91 | 0.029 | 16 | 0.04638 |
| **47** | tr|E9PEZ3|E9PEZ3_HUMAN |  | 31 | 0.99 | 0.006 | 16 | 0.7609 |
| **48** | tr|C9J3F6|C9J3F6_HUMAN |  | 3 | 0.87 | 0.02 | 16 | 0.002315 |
| **49** | tr|E9PIB2|E9PIB2_HUMAN |  | 1 | 1.03 | 0.056 | 16 | 0.5918 |
| **50** | sp|Q05209|PTN12_HUMAN |  | 1 | 1.07 | 0.125 | 16 | 0.4241 |
| **51** | tr|A0A024R1J4|A0A024R1J4_HUMAN |  | 1 | 0.96 | 0.024 | 16 | 0.3742 |
| **52** | tr|Q8N1B8|Q8N1B8_HUMAN |  | 1 | 0.93 | 0.128 | 16 | 0.434 |
| **53** | tr|B4DQ79|B4DQ79_HUMAN |  | 1 | 1 | 0.409 | 16 | 0.9975 |
| **54** | tr|C9JPA8|C9JPA8_HUMAN |  | 2 | 0.9 | 0.006 | 16 | 0.0001138 |
| **55** | tr|Q8NAJ6|Q8NAJ6_HUMAN |  | 1 | 1.05 | 0.049 | 16 | 0.4239 |
| **56** | tr|Q6FIA3|Q6FIA3_HUMAN |  | 6 | 1.1 | 0.017 | 16 | 0.009143 |
| **57** | sp|Q13561|DCTN2_HUMAN |  | 16 | 0.91 | 0.005 | 16 | 0.000244 |
| **58** | sp|Q8IVD9|NUDC3_HUMAN |  | 3 | 1.02 | 0.027 | 16 | 0.5574 |
| **59** | tr|H0YK97|H0YK97_HUMAN |  | 1 | 1.05 | 0.035 | 16 | 0.3246 |
| **60** | tr|A0A096LP07|A0A096LP07_HUMAN |  | 1 | 1.24 | 0.023 | 16 | 9.81E-06 |
| **61** | sp|P29218|IMPA1_HUMAN |  | 4 | 0.78 | 0.01 | 16 | 2.95E-07 |
| **62** | sp|Q9Y3I1|FBX7_HUMAN |  | 11 | 1.09 | 0.03 | 16 | 0.04708 |
| **63** | tr|B7Z9N9|B7Z9N9_HUMAN |  | 3 | 1.03 | 0.029 | 16 | 0.548 |
| **64** | tr|A0A024R7G6|A0A024R7G6_HUMAN |  | 10 | 1.06 | 0.004 | 16 | 0.002561 |
| **65** | tr|U5LKN0|U5LKN0_HUMAN |  | 8 | 1.03 | 0.077 | 16 | 0.6695 |
| **66** | tr|B3KX27|B3KX27_HUMAN |  | 1 | 0.81 | 0.043 | 16 | 0.002299 |
| **67** | sp|Q13418|ILK_HUMAN |  | 10 | 1.05 | 0.005 | 16 | 0.01506 |
| **68** | tr|C9J8R4|C9J8R4_HUMAN |  | 2 | 1.28 | 0.025 | 16 | 3.47E-06 |
| **69** | sp|Q6GMV3|PTRD1_HUMAN |  | 1 | 0.92 | 0.041 | 16 | 0.1495 |
| **70** | tr|V9HW25|V9HW25_HUMAN |  | 3 | 1.01 | 0.018 | 16 | 0.7389 |
| **71** | tr|A8K7W3|A8K7W3_HUMAN |  | 1 | 1.15 | 0.017 | 16 | 0.0003746 |
| **72** | tr|Q05BN7|Q05BN7_HUMAN |  | 1 | 0.77 | 0.009 | 16 | 1.10E-07 |
| **73** | tr|A0A087WXS7|A0A087WXS7_HUMAN |  | 10 | 0.91 | 0.002 | 16 | 9.18E-07 |
| **74** | tr|D6RF44|D6RF44_HUMAN |  | 1 | 1.49 | 0.099 | 16 | 1.75E-05 |
| **75** | tr|Q5FWF9|Q5FWF9_HUMAN |  | 1 | 0.84 | 0.074 | 16 | 0.02939 |
| **76** | sp|P13861|KAP2_HUMAN |  | 1 | 0.98 | 0.055 | 16 | 0.7432 |
| **77** | sp|P03973|SLPI_HUMAN |  | 1 | 0.99 | 0.006 | 16 | 0.6368 |
| **78** | tr|K7EMV3|K7EMV3_HUMAN |  | 1 | 2.17 | 0.222 | 16 | 5.39E-08 |
| **79** | tr|C9JYN0|C9JYN0_HUMAN |  | 1 | 0.91 | 0.009 | 16 | 0.001823 |
| **80** | sp|Q9BXD5|NPL_HUMAN |  | 6 | 1.09 | 0.005 | 16 | 0.0001216 |
| **81** | tr|B4DEW1|B4DEW1_HUMAN |  | 1 | 0.79 | 0.021 | 16 | 3.48E-05 |
| **82** | tr|Q6E433|Q6E433_HUMAN |  | 1 | 0.95 | 0.065 | 16 | 0.4607 |
| **83** | sp|P47755|CAZA2_HUMAN |  | 3 | 1.17 | 0.014 | 16 | 2.70E-05 |
| **84** | tr|Q7Z3Q8|Q7Z3Q8_HUMAN |  | 1 | 1.08 | 0.019 | 16 | 0.02662 |
| **85** | sp|Q9BU89|DOHH_HUMAN |  | 1 | 0.96 | 0.064 | 16 | 0.5585 |
| **86** | tr|B4DQ47|B4DQ47_HUMAN |  | 1 | 0.93 | 0.016 | 16 | 0.05636 |
| **87** | tr|B4DKP4|B4DKP4_HUMAN |  | 1 | 1.11 | 0.074 | 16 | 0.1219 |
| **88** | tr|B3KR68|B3KR68_HUMAN |  | 1 | 1.13 | 0.028 | 16 | 0.008984 |
| **89** | tr|Q6FH24|Q6FH24_HUMAN |  | 2 | 0.92 | 0.051 | 16 | 0.1549 |
| **90** | tr|A4QPB0|A4QPB0_HUMAN |  | 17 | 1.08 | 0.011 | 16 | 0.007744 |
| **91** | sp|P61204|ARF3_HUMAN |  | 3 | 0.98 | 0.043 | 16 | 0.6536 |
| **92** | sp|Q0JRZ9|FCHO2_HUMAN |  | 1 | 1.03 | 0.012 | 16 | 0.3629 |
| **93** | sp|P01619|KV301_HUMAN |  | 4 | 0.92 | 0.004 | 16 | 6.59E-05 |
| **94** | tr|Q7Z497|Q7Z497_HUMAN |  | 1 | 1.35 | 0.354 | 16 | 0.0344 |
| **95** | tr|E9PQI3|E9PQI3_HUMAN |  | 1 | 0.94 | 0.06 | 16 | 0.3274 |
| **96** | sp|Q9Y5K5|UCHL5_HUMAN |  | 5 | 0.99 | 0.008 | 16 | 0.522 |
| **97** | tr|Q59FG1|Q59FG1_HUMAN |  | 1 | 1.06 | 0.034 | 16 | 0.2223 |
| **98** | sp|P01624|KV306_HUMAN |  | 1 | 0.82 | 0.079 | 16 | 0.02184 |
| **99** | tr|B4DUV1|B4DUV1_HUMAN |  | 3 | 0.96 | 0.007 | 16 | 0.08712 |
| **100** | sp|Q92696|PGTA_HUMAN |  | 5 | 1.01 | 0.007 | 16 | 0.6484 |
| **101** | tr|K7ER74|K7ER74_HUMAN |  | 3 | 0.98 | 0.056 | 16 | 0.7527 |
| **102** | tr|Q05DH1|Q05DH1_HUMAN |  | 13 | 1.08 | 0.005 | 16 | 0.0003563 |
| **103** | tr|Q9BXA2|Q9BXA2_HUMAN |  | 1 | 1.45 | 0.09 | 16 | 2.33E-05 |
| **104** | sp|Q9UJ70|NAGK_HUMAN |  | 4 | 1.12 | 0.021 | 16 | 0.003396 |
| **105** | sp|O95163|ELP1_HUMAN |  | 3 | 1.17 | 0.038 | 16 | 0.004173 |
| **106** | tr|B4DRW1|B4DRW1_HUMAN |  | 1 | 0.89 | 0.105 | 16 | 0.187 |
| **107** | tr|D3DV11|D3DV11_HUMAN |  | 1 | 1.01 | 0.04 | 16 | 0.8847 |
| **108** | sp|Q9UHV9|PFD2_HUMAN |  | 3 | 0.91 | 0.029 | 16 | 0.05283 |
| **109** | tr|H0YNE5|H0YNE5_HUMAN |  | 1 | 0.93 | 0.05 | 16 | 0.2339 |
| **110** | sp|Q9H040|SPRTN_HUMAN |  | 1 | 1.1 | 0.006 | 16 | 9.86E-05 |
| **111** | tr|M0QX47|M0QX47_HUMAN |  | 2 | 1.12 | 0.012 | 16 | 0.0006867 |
| **112** | tr|B4DM84|B4DM84_HUMAN |  | 4 | 0.99 | 0.009 | 16 | 0.6996 |
| **113** | tr|D3VVL9|D3VVL9_HUMAN |  | 1 | 0.87 | 0.018 | 16 | 0.001048 |
| **114** | tr|B1ANR0|B1ANR0_HUMAN |  | 1 | 1.14 | 0.095 | 16 | 0.09373 |
| **115** | tr|C9J0K6|C9J0K6_HUMAN |  | 10 | 1.05 | 0.008 | 16 | 0.03922 |
| **116** | sp|P52907|CAZA1_HUMAN |  | 6 | 1.09 | 0.012 | 16 | 0.007047 |
| **117** | sp|P02774|VTDB_HUMAN |  | 20 | 1.29 | 0.029 | 16 | 6.31E-06 |
| **118** | sp|Q5VXD3|SAM13_HUMAN |  | 1 | 1.15 | 0.105 | 16 | 0.08389 |
| **119** | sp|Q14204|DYHC1_HUMAN |  | 1 | 2.4 | 13.59 | 16 | 0.1494 |
| **120** | sp|Q96FZ7|CHMP6_HUMAN |  | 3 | 0.84 | 0.004 | 16 | 5.16E-08 |
| **121** | sp|P60900|PSA6_HUMAN |  | 9 | 1.05 | 0.004 | 16 | 0.004975 |
| **122** | tr|B4DHX4|B4DHX4_HUMAN |  | 1 | 0.83 | 0.091 | 16 | 0.03721 |
| **123** | tr|Q9H3K5|Q9H3K5_HUMAN |  | 1 | 0.74 | 0.003 | 4 | 0.002687 |
| **124** | sp|P61081|UBC12_HUMAN |  | 6 | 0.96 | 0.075 | 16 | 0.5239 |
| **125** | sp|Q9BTW9|TBCD_HUMAN |  | 9 | 0.98 | 0.005 | 16 | 0.273 |
| **126** | sp|P01714|LV301_HUMAN |  | 1 | 1.03 | 0.005 | 16 | 0.07529 |
| **127** | sp|Q8TEH3|DEN1A_HUMAN |  | 1 | 1.08 | 0.082 | 16 | 0.306 |
| **128** | sp|P49720|PSB3_HUMAN |  | 3 | 1.07 | 0.008 | 16 | 0.004478 |
| **129** | sp|Q07960|RHG01_HUMAN |  | 11 | 1.11 | 0.055 | 16 | 0.07126 |
| **130** | sp|P05109|S10A8_HUMAN |  | 4 | 0.91 | 0.018 | 16 | 0.01437 |
| **131** | sp|Q9NR12|PDLI7_HUMAN |  | 1 | 1.29 | 0.137 | 16 | 0.006661 |
| **132** | sp|P01591|IGJ_HUMAN |  | 2 | 0.85 | 0.013 | 16 | 9.02E-05 |
| **133** | sp|Q14974|IMB1_HUMAN |  | 23 | 0.9 | 0.002 | 16 | 4.84E-08 |
| **134** | tr|U3KPT8|U3KPT8_HUMAN |  | 1 | 1.07 | 0.016 | 16 | 0.05436 |
| **135** | tr|Q16519|Q16519_HUMAN |  | 1 | 0.9 | 0.016 | 16 | 0.006842 |
| **136** | sp|P04275|VWF_HUMAN |  | 1 | 0.94 | 0.089 | 16 | 0.4278 |
| **137** | tr|E9PNS3|E9PNS3_HUMAN |  | 1 | 0.97 | 0.065 | 16 | 0.6095 |
| **138** | tr|Q8J008|Q8J008_HUMAN |  | 1 | 1.01 | 0.025 | 16 | 0.8696 |
| **139** | tr|Q59G85|Q59G85_HUMAN |  | 1 | 1.09 | 0.047 | 16 | 0.1336 |
| **140** | sp|P63104|1433Z_HUMAN |  | 2 | 1.26 | 0.011 | 16 | 4.16E-08 |
| **141** | tr|A8K048|A8K048_HUMAN |  | 18 | 0.98 | 0.002 | 16 | 0.1669 |
| **142** | sp|P08311|CATG_HUMAN |  | 6 | 2.12 | 0.103 | 16 | 5.28E-10 |
| **143** | tr|B4DG10|B4DG10_HUMAN |  | 1 | 0.93 | 0.039 | 16 | 0.1651 |
| **144** | sp|P67870|CSK2B_HUMAN |  | 6 | 0.93 | 0.004 | 16 | 0.0004948 |
| **145** | sp|P62166|NCS1_HUMAN |  | 2 | 1.07 | 0.062 | 16 | 0.3024 |
| **146** | sp|A8MVM7|YD021_HUMAN |  | 1 | 1.26 | 0.205 | 16 | 0.03911 |
| **147** | sp|Q9Y490|TLN1_HUMAN |  | 66 | 1.02 | 0.021 | 16 | 0.6653 |
| **148** | sp|P28074|PSB5_HUMAN |  | 11 | 1.05 | 0.003 | 16 | 0.001202 |
| **149** | sp|O14498|ISLR_HUMAN |  | 1 | 1.14 | 0.144 | 16 | 0.172 |
| **150** | sp|P17655|CAN2_HUMAN |  | 8 | 0.91 | 0.03 | 16 | 0.05262 |
| **151** | tr|B7Z756|B7Z756_HUMAN |  | 1 | 1.06 | 0.079 | 16 | 0.3948 |
| **152** | tr|Q8WY19|Q8WY19_HUMAN |  | 1 | 0.92 | 0.023 | 16 | 0.06505 |
| **153** | sp|O00154|BACH_HUMAN |  | 8 | 0.99 | 0.011 | 16 | 0.7757 |
| **154** | sp|P01625|KV402_HUMAN |  | 1 | 0.95 | 0.007 | 16 | 0.03312 |
| **155** | tr|A2RRE5|A2RRE5_HUMAN |  | 1 | 0.95 | 0.057 | 16 | 0.4057 |
| **156** | sp|P02647|APOA1_HUMAN |  | 30 | 1.21 | 0.014 | 16 | 3.28E-06 |
| **157** | sp|P50395|GDIB_HUMAN |  | 3 | 0.99 | 0.025 | 16 | 0.7736 |
| **158** | sp|O00151|PDLI1_HUMAN |  | 4 | 1.06 | 0.028 | 16 | 0.1468 |
| **159** | tr|H0Y8Y3|H0Y8Y3_HUMAN |  | 1 | 1.03 | 0.067 | 16 | 0.622 |
| **160** | sp|P01616|KV203_HUMAN |  | 1 | 1.15 | 0.066 | 16 | 0.03198 |
| **161** | tr|A0A087WW89|A0A087WW89_HUMAN |  | 2 | 0.89 | 0.008 | 16 | 0.0002912 |
| **162** | tr|B5MC96|B5MC96_HUMAN |  | 1 | 1.87 | 2.822 | 16 | 0.05676 |
| **163** | sp|P48735|IDHP_HUMAN |  | 1 | 1.17 | 0.114 | 16 | 0.06205 |
| **164** | tr|A8K4R4|A8K4R4_HUMAN |  | 1 | 1.14 | 0.073 | 16 | 0.05667 |
| **165** | tr|Q8IUB0|Q8IUB0_HUMAN |  | 5 | 1 | 0.016 | 16 | 0.9126 |
| **166** | sp|Q9BT78|CSN4_HUMAN |  | 20 | 0.98 | 0.002 | 16 | 0.08299 |
| **167** | tr|Q7M4S4|Q7M4S4_HUMAN |  | 1 | 1.12 | 0.044 | 16 | 0.04307 |
| **168** | sp|Q9Y2J4|AMOL2_HUMAN |  | 1 | 0.99 | 0.066 | 16 | 0.8587 |
| **169** | sp|Q5TGY3|AHDC1_HUMAN |  | 1 | 1.74 | 2.98 | 16 | 0.1056 |
| **170** | sp|P23526|SAHH_HUMAN |  | 3 | 1.07 | 0.009 | 16 | 0.01356 |
| **171** | tr|H3BPK3|H3BPK3_HUMAN |  | 5 | 0.92 | 0.036 | 16 | 0.1066 |
| **172** | tr|B3KP05|B3KP05_HUMAN |  | 1 | 0.91 | 0.023 | 16 | 0.02693 |
| **173** | tr|B4DGY8|B4DGY8_HUMAN |  | 1 | 1.03 | 0.106 | 16 | 0.73 |
| **174** | sp|Q9H9Q2|CSN7B_HUMAN |  | 2 | 0.95 | 0.006 | 16 | 0.02389 |
| **175** | tr|K4DI93|K4DI93_HUMAN |  | 1 | 0.91 | 0.092 | 16 | 0.2442 |
| **176** | tr|H0YJY7|H0YJY7_HUMAN |  | 1 | 0.97 | 0.181 | 16 | 0.7922 |
| **177** | sp|P0CW22|RS17L_HUMAN |  | 1 | 0.95 | 0.014 | 16 | 0.08164 |
| **178** | tr|V9GYZ0|V9GYZ0_HUMAN |  | 3 | 0.92 | 0.011 | 16 | 0.007097 |
| **179** | tr|F6T1Q0|F6T1Q0_HUMAN |  | 5 | 1.09 | 0.007 | 16 | 0.0006623 |
| **180** | sp|P31948|STIP1_HUMAN |  | 9 | 1.1 | 0.013 | 16 | 0.004434 |
| **181** | tr|H3BTA2|H3BTA2_HUMAN |  | 1 | 0.79 | 0.105 | 16 | 0.01856 |
| **182** | sp|Q15386|UBE3C_HUMAN |  | 4 | 0.99 | 0.013 | 16 | 0.7588 |
| **183** | sp|P02743|SAMP_HUMAN |  | 8 | 1.42 | 0.023 | 16 | 1.23E-08 |
| **184** | sp|P22792|CPN2_HUMAN |  | 7 | 1.25 | 0.008 | 16 | 7.35E-09 |
| **185** | sp|Q96ER3|SAAL1_HUMAN |  | 1 | 0.88 | 0.023 | 16 | 0.005315 |
| **186** | sp|P35219|CAH8_HUMAN |  | 4 | 0.91 | 0.023 | 16 | 0.03302 |
| **187** | tr|A8K4W8|A8K4W8_HUMAN |  | 1 | 1 | 0.016 | 16 | 0.8837 |
| **188** | sp|Q9NWV8|BABA1_HUMAN |  | 2 | 0.96 | 0.003 | 16 | 0.005135 |
| **189** | sp|P67775|PP2AA_HUMAN |  | 2 | 1.08 | 0.009 | 16 | 0.004784 |
| **190** | sp|P02790|HEMO_HUMAN |  | 6 | 1.09 | 0.031 | 16 | 0.05843 |
| **191** | tr|Q562M3|Q562M3_HUMAN |  | 1 | 0.99 | 0.022 | 16 | 0.8239 |
| **192** | sp|Q9Y4X5|ARI1_HUMAN |  | 2 | 0.85 | 0.014 | 16 | 0.0001146 |
| **193** | tr|H3BN55|H3BN55_HUMAN |  | 2 | 1.25 | 0.02 | 16 | 3.10E-06 |
| **194** | tr|B7ZLZ7|B7ZLZ7_HUMAN |  | 9 | 1 | 0.001 | 16 | 0.712 |
| **195** | sp|P80748|LV302_HUMAN |  | 1 | 1.17 | 0.069 | 16 | 0.0188 |
| **196** | sp|P01613|KV121_HUMAN |  | 1 | 0.93 | 0.018 | 16 | 0.04794 |
| **197** | sp|Q9H0U4|RAB1B_HUMAN |  | 2 | 1.08 | 0.005 | 16 | 0.0003303 |
| **198** | tr|Q05DK5|Q05DK5_HUMAN |  | 15 | 1.05 | 0.065 | 16 | 0.4232 |
| **199** | sp|O00160|MYO1F_HUMAN |  | 3 | 0.84 | 0.053 | 16 | 0.01567 |
| **200** | tr|B2RAR0|B2RAR0_HUMAN |  | 7 | 1.13 | 0.015 | 16 | 0.0008586 |
| **201** | sp|P78318|IGBP1_HUMAN |  | 7 | 0.93 | 0.003 | 16 | 0.0002142 |
| **202** | sp|P11277|SPTB1_HUMAN |  | 2 | 0.81 | 0.012 | 16 | 5.37E-06 |
| **203** | tr|Q8WVC2|Q8WVC2_HUMAN |  | 1 | 1.07 | 0.091 | 16 | 0.3847 |
| **204** | sp|Q13131|AAPK1_HUMAN |  | 4 | 0.98 | 0.013 | 16 | 0.5678 |
| **205** | tr|C9JNM8|C9JNM8_HUMAN |  | 1 | 0.88 | 0.049 | 16 | 0.04508 |
| **206** | tr|B1Q3B3|B1Q3B3_HUMAN |  | 1 | 0.78 | 0.04 | 16 | 0.0005802 |
| **207** | sp|P13671|CO6_HUMAN |  | 2 | 1.04 | 0.026 | 16 | 0.3239 |
| **208** | sp|Q08211|DHX9_HUMAN |  | 9 | 1.12 | 0.022 | 16 | 0.006907 |
| **209** | tr|E5RFX8|E5RFX8_HUMAN |  | 1 | 0.8 | 0.007 | 16 | 7.20E-08 |
| **210** | tr|C7DUW4|C7DUW4_HUMAN |  | 7 | 0.95 | 0.005 | 16 | 0.01381 |
| **211** | tr|B4DY09|B4DY09_HUMAN |  | 3 | 1.19 | 0.019 | 16 | 6.97E-05 |
| **212** | tr|B3KRM2|B3KRM2_HUMAN |  | 1 | 0.86 | 0.043 | 16 | 0.01547 |
| **213** | sp|Q5EBM0|CMPK2_HUMAN |  | 1 | 1.18 | 0.049 | 16 | 0.004791 |
| **214** | sp|Q13630|FCL_HUMAN |  | 1 | 1.06 | 0.004 | 16 | 0.0006664 |
| **215** | tr|J3KNE3|J3KNE3_HUMAN |  | 1 | 1.08 | 0.036 | 16 | 0.1128 |
| **216** | tr|I3L2V0|I3L2V0_HUMAN |  | 1 | 0.91 | 0.027 | 16 | 0.0481 |
| **217** | sp|P20742|PZP_HUMAN |  | 8 | 1.09 | 0.066 | 16 | 0.1929 |
| **218** | tr|H0YKU7|H0YKU7_HUMAN |  | 1 | 1.23 | 0.096 | 16 | 0.009705 |
| **219** | tr|K9J972|K9J972_HUMAN |  | 1 | 1.28 | 0.365 | 16 | 0.07945 |
| **220** | tr|F8VV49|F8VV49_HUMAN |  | 1 | 1.13 | 0.143 | 16 | 0.1805 |
| **221** | tr|H7C352|H7C352_HUMAN |  | 1 | 1.02 | 0.027 | 16 | 0.6706 |
| **222** | tr|Q9H032|Q9H032_HUMAN |  | 1 | 0.94 | 0.022 | 16 | 0.1451 |
| **223** | tr|B4DMH3|B4DMH3_HUMAN |  | 4 | 1.22 | 0.014 | 16 | 2.14E-06 |
| **224** | tr|B3KTM6|B3KTM6_HUMAN |  | 1 | 1.18 | 0.008 | 16 | 8.28E-07 |
| **225** | sp|Q86SG5|S1A7A_HUMAN |  | 1 | 0.72 | 0.455 | 16 | 0.1135 |
| **226** | tr|Q569I7|Q569I7_HUMAN |  | 1 | 1.31 | 0.144 | 16 | 0.005027 |
| **227** | tr|O14724|O14724_HUMAN |  | 1 | 0.61 | 0.024 | 16 | 4.39E-08 |
| **228** | tr|B4DGP9|B4DGP9_HUMAN |  | 1 | 1.09 | 0.024 | 16 | 0.02887 |
| **229** | sp|P58397|ATS12_HUMAN |  | 1 | 1.8 | 0.946 | 16 | 0.005083 |
| **230** | tr|B4DLW1|B4DLW1_HUMAN |  | 1 | 0.97 | 0.092 | 16 | 0.652 |
| **231** | tr|H0YDB2|H0YDB2_HUMAN |  | 1 | 0.9 | 0.11 | 16 | 0.2642 |
| **232** | tr|Q86WV2|Q86WV2_HUMAN |  | 1 | 1.08 | 0.054 | 16 | 0.2171 |
| **233** | tr|B7Z7R2|B7Z7R2_HUMAN |  | 1 | 1.04 | 0.023 | 16 | 0.2687 |
| **234** | tr|B2RC09|B2RC09_HUMAN |  | 1 | 1.07 | 0.092 | 16 | 0.3787 |
| **235** | sp|Q52LW3|RHG29_HUMAN |  | 1 | 1.91 | 2.242 | 16 | 0.02874 |
| **236** | sp|P08603|CFAH_HUMAN |  | 1 | 1.09 | 0.024 | 16 | 0.02674 |
| **237** | sp|P11413|G6PD_HUMAN |  | 19 | 1.13 | 0.005 | 16 | 3.42E-06 |
| **238** | tr|Q9BTI6|Q9BTI6_HUMAN |  | 2 | 0.79 | 0.048 | 16 | 0.001533 |
| **239** | tr|A0PJ51|A0PJ51_HUMAN |  | 2 | 1.15 | 0.069 | 16 | 0.04062 |
| **240** | tr|H7BZL2|H7BZL2_HUMAN |  | 1 | 0.99 | 0.066 | 16 | 0.8846 |
| **241** | tr|A8KA24|A8KA24_HUMAN |  | 1 | 0.75 | 0.034 | 16 | 7.67E-05 |
| **242** | tr|A2N0T3|A2N0T3_HUMAN |  | 1 | 1.4 | 0.301 | 16 | 0.01061 |
| **243** | sp|Q8TBF2|PGFS_HUMAN |  | 2 | 0.85 | 0.035 | 16 | 0.006969 |
| **244** | tr|Q5JZH0|Q5JZH0_HUMAN |  | 1 | 1.05 | 0.115 | 16 | 0.5601 |
| **245** | tr|D3DTH7|D3DTH7_HUMAN |  | 1 | 1.09 | 0.025 | 16 | 0.04224 |
| **246** | tr|B4DUJ3|B4DUJ3_HUMAN |  | 1 | 0.84 | 0.051 | 16 | 0.01457 |
| **247** | sp|P46934|NEDD4_HUMAN |  | 1 | 0.86 | 0.014 | 16 | 0.0003741 |
| **248** | tr|A0N5G5|A0N5G5_HUMAN |  | 1 | 0.89 | 0.019 | 16 | 0.007939 |
| **249** | sp|O60518|RNBP6_HUMAN |  | 1 | 0.86 | 0.015 | 16 | 0.0004488 |
| **250** | sp|P11215|ITAM_HUMAN |  | 3 | 1.36 | 0.186 | 16 | 0.004657 |
| **251** | tr|F6KPG5|F6KPG5_HUMAN |  | 38 | 1.01 | 0.089 | 16 | 0.8799 |
| **252** | tr|C9JJU7|C9JJU7_HUMAN |  | 1 | 1.3 | 0.057 | 16 | 0.0001405 |
| **253** | tr|H7C2Q3|H7C2Q3_HUMAN |  | 1 | 1 | 0.038 | 16 | 0.929 |
| **254** | sp|Q15435|PP1R7_HUMAN |  | 12 | 1.11 | 0.008 | 16 | 0.0002304 |
| **255** | tr|B4DU91|B4DU91_HUMAN |  | 3 | 1.04 | 0.027 | 16 | 0.3652 |
| **256** | tr|B3KN49|B3KN49_HUMAN |  | 1 | 0.94 | 0.058 | 16 | 0.3184 |
| **257** | tr|H0YIC9|H0YIC9_HUMAN |  | 2 | 1 | 0.009 | 16 | 0.9483 |
| **258** | sp|Q8WWZ8|OIT3_HUMAN |  | 1 | 1.16 | 0.235 | 16 | 0.1994 |
| **259** | tr|M0QYV0|M0QYV0_HUMAN |  | 2 | 1.14 | 0.085 | 16 | 0.07268 |
| **260** | tr|B2RCX0|B2RCX0_HUMAN |  | 1 | 1.03 | 0.036 | 16 | 0.4753 |
| **261** | sp|P25786|PSA1_HUMAN |  | 5 | 1.07 | 0.003 | 16 | 8.02E-05 |
| **262** | sp|Q86YW5|TRML1_HUMAN |  | 1 | 0.96 | 0.094 | 16 | 0.5781 |
| **263** | sp|P38919|IF4A3_HUMAN |  | 1 | 1.22 | 0.079 | 16 | 0.00777 |
| **264** | sp|Q6ICL3|TNG2_HUMAN |  | 3 | 1.1 | 0.005 | 16 | 9.34E-05 |
| **265** | tr|H7C3A0|H7C3A0_HUMAN |  | 1 | 0.96 | 0.066 | 16 | 0.593 |
| **266** | tr|C0JYY2|C0JYY2_HUMAN |  | 2 | 0.97 | 0.02 | 16 | 0.3494 |
| **267** | tr|A0A087X234|A0A087X234_HUMAN |  | 1 | 4.07 | 14.878 | 16 | 0.006126 |
| **268** | tr|Q8N1A3|Q8N1A3_HUMAN |  | 1 | 1.28 | 0.046 | 16 | 9.96E-05 |
| **269** | tr|A8K781|A8K781_HUMAN |  | 21 | 0.94 | 0.004 | 16 | 0.001036 |
| **270** | tr|Q9H3Z3|Q9H3Z3_HUMAN |  | 1 | 0.83 | 0.037 | 16 | 0.003124 |
| **271** | sp|P35580|MYH10_HUMAN |  | 24 | 0.87 | 0.009 | 16 | 0.0001111 |
| **272** | tr|Q6DC98|Q6DC98_HUMAN |  | 2 | 1.44 | 0.095 | 16 | 4.50E-05 |
| **273** | tr|A0A024RC87|A0A024RC87_HUMAN |  | 21 | 0.89 | 0.008 | 16 | 0.0001239 |
| **274** | sp|O75955|FLOT1_HUMAN |  | 18 | 0.93 | 0.059 | 16 | 0.2526 |
| **275** | tr|F5GZP0|F5GZP0_HUMAN |  | 2 | 1.03 | 0.082 | 16 | 0.6656 |
| **276** | tr|A0A024RB67|A0A024RB67_HUMAN |  | 3 | 0.77 | 0.006 | 16 | 4.01E-09 |
| **277** | tr|E7EXB4|E7EXB4_HUMAN |  | 1 | 0.63 | 0.131 | 16 | 0.00104 |
| **278** | tr|B4DPJ2|B4DPJ2_HUMAN |  | 9 | 1.02 | 0.009 | 16 | 0.4476 |
| **279** | sp|O75116|ROCK2_HUMAN |  | 10 | 1.03 | 0.005 | 16 | 0.1678 |
| **280** | sp|P02765|FETUA_HUMAN |  | 7 | 1.17 | 0.011 | 16 | 9.14E-06 |
| **281** | tr|J3K000|J3K000_HUMAN |  | 1 | 1.02 | 0.023 | 16 | 0.5224 |
| **282** | tr|G3XAP6|G3XAP6_HUMAN |  | 1 | 0.92 | 0.015 | 16 | 0.01516 |
| **283** | sp|Q6A163|K1C39_HUMAN |  | 1 | 0.92 | 0.033 | 16 | 0.08248 |
| **284** | tr|A0A0A0MSV6|A0A0A0MSV6_HUMAN |  | 6 | 0.96 | 0.01 | 16 | 0.1618 |
| **285** | sp|O95373|IPO7_HUMAN |  | 14 | 0.8 | 0.004 | 16 | 2.89E-09 |
| **286** | tr|B4DHN4|B4DHN4_HUMAN |  | 1 | 0.76 | 0.018 | 16 | 3.39E-06 |
| **287** | tr|W0S0D3|W0S0D3_HUMAN |  | 1 | 0.93 | 0.051 | 16 | 0.2131 |
| **288** | tr|H6VRG3|H6VRG3_HUMAN |  | 1 | 0.92 | 0.165 | 16 | 0.4648 |
| **289** | tr|B3VL17|B3VL17_HUMAN |  | 8 | 0.91 | 0.056 | 16 | 0.1447 |
| **290** | sp|Q9Y678|COPG1_HUMAN |  | 1 | 0.88 | 0.019 | 16 | 0.00432 |
| **291** | tr|B4DJ98|B4DJ98_HUMAN |  | 1 | 1.2 | 0.123 | 16 | 0.03455 |
| **292** | sp|P31949|S10AB_HUMAN |  | 1 | 0.9 | 0.036 | 16 | 0.04747 |
| **293** | tr|A8K9T2|A8K9T2_HUMAN |  | 13 | 0.93 | 0.003 | 16 | 0.0002465 |
| **294** | sp|P62136|PP1A_HUMAN |  | 1 | 1.05 | 0.006 | 16 | 0.03374 |
| **295** | tr|Q8IZI0|Q8IZI0_HUMAN |  | 1 | 0.95 | 0.041 | 16 | 0.3485 |
| **296** | tr|B2R983|B2R983_HUMAN |  | 3 | 0.96 | 0.011 | 16 | 0.1347 |
| **297** | sp|P08514|ITA2B_HUMAN |  | 12 | 0.93 | 0.057 | 16 | 0.2682 |
| **298** | sp|P46019|KPB2_HUMAN |  | 6 | 0.85 | 0.006 | 16 | 1.80E-06 |
| **299** | tr|B7ZLC9|B7ZLC9_HUMAN |  | 2 | 1.17 | 0.214 | 16 | 0.1723 |
| **300** | tr|F8VYN5|F8VYN5_HUMAN |  | 1 | 1.3 | 0.096 | 16 | 0.001368 |
| **301** | tr|A0A024R8V7|A0A024R8V7_HUMAN |  | 1 | 1.09 | 0.032 | 16 | 0.06138 |
| **302** | tr|B3KXI7|B3KXI7_HUMAN |  | 1 | 0.84 | 0.005 | 16 | 2.58E-07 |
| **303** | sp|O43707|ACTN4_HUMAN |  | 13 | 1.11 | 0.003 | 16 | 3.23E-07 |
| **304** | tr|H0YDA1|H0YDA1_HUMAN |  | 1 | 0.78 | 0.025 | 16 | 5.27E-05 |
| **305** | tr|B7Z8A2|B7Z8A2_HUMAN |  | 6 | 1.04 | 0.01 | 16 | 0.1325 |
| **306** | sp|Q5T447|HECD3_HUMAN |  | 4 | 0.93 | 0.005 | 16 | 0.002918 |
| **307** | sp|Q6VN20|RBP10_HUMAN |  | 8 | 1.1 | 0.03 | 16 | 0.03998 |
| **308** | tr|D6R9I9|D6R9I9_HUMAN |  | 3 | 1.06 | 0.008 | 16 | 0.01766 |
| **309** | tr|E9PC74|E9PC74_HUMAN |  | 3 | 1.09 | 0.175 | 16 | 0.3809 |
| **310** | sp|P03950|ANGI_HUMAN |  | 1 | 0.96 | 0.02 | 16 | 0.2221 |
| **311** | sp|O60493|SNX3_HUMAN |  | 3 | 1.11 | 0.029 | 16 | 0.01828 |
| **312** | tr|B4DMW4|B4DMW4_HUMAN |  | 1 | 1.01 | 0.017 | 16 | 0.7138 |
| **313** | tr|H3BUD2|H3BUD2_HUMAN |  | 1 | 0.89 | 0.051 | 16 | 0.07655 |
| **314** | sp|P00739|HPTR_HUMAN |  | 2 | 0.9 | 0.02 | 16 | 0.01602 |
| **315** | tr|I3L1J2|I3L1J2_HUMAN |  | 6 | 1.05 | 0.002 | 16 | 0.000134 |
| **316** | tr|A6NLN1|A6NLN1_HUMAN |  | 4 | 1.1 | 0.025 | 16 | 0.01729 |
| **317** | sp|P23381|SYWC_HUMAN |  | 2 | 0.92 | 0.011 | 16 | 0.007268 |
| **318** | tr|B3KP90|B3KP90_HUMAN |  | 6 | 1.03 | 0.01 | 16 | 0.3245 |
| **319** | tr|D3DWK4|D3DWK4_HUMAN |  | 1 | 0.96 | 0.041 | 16 | 0.4825 |
| **320** | tr|A8K477|A8K477_HUMAN |  | 12 | 0.98 | 0.009 | 16 | 0.3661 |
| **321** | sp|Q15084|PDIA6_HUMAN |  | 7 | 1.07 | 0.003 | 16 | 0.0001742 |
| **322** | sp|Q9BT73|PSMG3_HUMAN |  | 2 | 1.6 | 0.816 | 16 | 0.01756 |
| **323** | sp|O75531|BAF_HUMAN |  | 4 | 1.04 | 0.055 | 16 | 0.455 |
| **324** | tr|E9PS77|E9PS77_HUMAN |  | 1 | 0.96 | 0.006 | 16 | 0.03304 |
| **325** | tr|B4E1T1|B4E1T1_HUMAN |  | 3 | 0.89 | 0.051 | 16 | 0.08153 |
| **326** | tr|Q4TT60|Q4TT60_HUMAN |  | 1 | 1 | 0.11 | 16 | 0.974 |
| **327** | tr|Q59HG6|Q59HG6_HUMAN |  | 1 | 1.31 | 0.046 | 16 | 3.42E-05 |
| **328** | tr|S6BGD6|S6BGD6_HUMAN |  | 1 | 0.82 | 0.024 | 16 | 0.0003996 |
| **329** | sp|Q9UJX2|CDC23_HUMAN |  | 1 | 1.08 | 0.133 | 16 | 0.3756 |
| **330** | tr|E7ER27|E7ER27_HUMAN |  | 1 | 1.35 | 0.191 | 16 | 0.0065 |
| **331** | sp|O75170|PP6R2_HUMAN |  | 1 | 0.97 | 0.024 | 16 | 0.4626 |
| **332** | tr|H7C5M4|H7C5M4_HUMAN |  | 1 | 1.07 | 0.044 | 16 | 0.2189 |
| **333** | sp|P02675|FIBB_HUMAN |  | 36 | 1.09 | 0.023 | 16 | 0.03061 |
| **334** | tr|Q6PK82|Q6PK82_HUMAN |  | 1 | 1.17 | 0.317 | 16 | 0.2406 |
| **335** | tr|Q9NPK3|Q9NPK3_HUMAN |  | 9 | 0.94 | 0.008 | 16 | 0.01265 |
| **336** | tr|B4DDJ7|B4DDJ7_HUMAN |  | 1 | 1.2 | 0.292 | 16 | 0.1595 |
| **337** | tr|E9PP36|E9PP36_HUMAN |  | 2 | 1.07 | 0.018 | 16 | 0.04438 |
| **338** | sp|P26640|SYVC_HUMAN |  | 4 | 1.02 | 0.027 | 16 | 0.5858 |
| **339** | sp|Q12986|NFX1_HUMAN |  | 2 | 0.89 | 0.039 | 16 | 0.03875 |
| **340** | tr|Q5T3N1|Q5T3N1_HUMAN |  | 1 | 0.89 | 0.116 | 16 | 0.2343 |
| **341** | sp|P62633|CNBP_HUMAN |  | 2 | 0.67 | 0.125 | 16 | 0.002125 |
| **342** | sp|Q5T0N5|FBP1L_HUMAN |  | 3 | 0.95 | 0.008 | 16 | 0.04422 |
| **343** | sp|Q6ZMI0|PPR21_HUMAN |  | 2 | 0.95 | 0.067 | 16 | 0.4846 |
| **344** | tr|Q53GW1|Q53GW1_HUMAN |  | 15 | 0.93 | 0.016 | 16 | 0.0305 |
| **345** | sp|P54105|ICLN_HUMAN |  | 1 | 0.9 | 0.007 | 16 | 0.0002636 |
| **346** | tr|A0A024R5A3|A0A024R5A3_HUMAN |  | 13 | 1.07 | 0.006 | 16 | 0.001575 |
| **347** | sp|Q9H3U1|UN45A_HUMAN |  | 2 | 1.14 | 0.013 | 16 | 0.0001944 |
| **348** | tr|T2DL33|T2DL33_HUMAN |  | 2 | 0.85 | 0.046 | 16 | 0.01187 |
| **349** | tr|B4DJQ7|B4DJQ7_HUMAN |  | 3 | 0.98 | 0.017 | 16 | 0.4996 |
| **350** | tr|D6RBM3|D6RBM3_HUMAN |  | 1 | 0.83 | 0.025 | 16 | 0.0007742 |
| **351** | tr|L8E745|L8E745_HUMAN |  | 1 | 0.89 | 0.013 | 16 | 0.001448 |
| **352** | tr|Q53H03|Q53H03_HUMAN |  | 9 | 0.94 | 0.004 | 16 | 0.00158 |
| **353** | sp|P09871|C1S_HUMAN |  | 19 | 0.89 | 0.005 | 16 | 1.12E-05 |
| **354** | sp|Q9H814|PHAX_HUMAN |  | 2 | 0.97 | 0.024 | 16 | 0.4718 |
| **355** | sp|Q9NX55|HYPK_HUMAN |  | 1 | 0.79 | 0.026 | 16 | 8.89E-05 |
| **356** | tr|F5H345|F5H345_HUMAN |  | 8 | 0.87 | 0.034 | 16 | 0.01416 |
| **357** | tr|H7C1M3|H7C1M3_HUMAN |  | 1 | 0.85 | 0.009 | 16 | 1.37E-05 |
| **358** | tr|H3BNG3|H3BNG3_HUMAN |  | 1 | 0.93 | 0.037 | 16 | 0.1598 |
| **359** | sp|P25788|PSA3_HUMAN |  | 3 | 0.99 | 0.006 | 16 | 0.6937 |
| **360** | tr|L8ECK9|L8ECK9_HUMAN |  | 1 | 1.05 | 0.008 | 16 | 0.04933 |
| **361** | sp|Q9NZN3|EHD3_HUMAN |  | 5 | 1.04 | 0.014 | 16 | 0.1868 |
| **362** | sp|P05160|F13B_HUMAN |  | 13 | 1 | 0.01 | 16 | 0.9902 |
| **363** | tr|B4E3V6|B4E3V6_HUMAN |  | 1 | 1.02 | 0.02 | 16 | 0.6207 |
| **364** | tr|B2R4X4|B2R4X4_HUMAN |  | 1 | 0.78 | 0.023 | 16 | 2.68E-05 |
| **365** | sp|Q9GZZ9|UBA5_HUMAN |  | 3 | 0.97 | 0.002 | 16 | 0.03481 |
| **366** | tr|Q6MZX6|Q6MZX6_HUMAN |  | 1 | 1.05 | 0.007 | 16 | 0.03616 |
| **367** | tr|A8K5M4|A8K5M4_HUMAN |  | 3 | 0.95 | 0.045 | 16 | 0.3208 |
| **368** | sp|Q969R2|OSBP2_HUMAN |  | 1 | 1.02 | 0.005 | 16 | 0.2549 |
| **369** | sp|Q9H3D4|P63_HUMAN |  | 1 | 1.06 | 0.026 | 16 | 0.1467 |
| **370** | tr|B3KNN3|B3KNN3_HUMAN |  | 1 | 0.75 | 0.063 | 16 | 0.001043 |
| **371** | tr|C9IZG4|C9IZG4_HUMAN |  | 4 | 1 | 0.005 | 16 | 0.949 |
| **372** | tr|J3QLH1|J3QLH1_HUMAN |  | 1 | 1.25 | 0.081 | 16 | 0.003339 |
| **373** | tr|G3XAN0|G3XAN0_HUMAN |  | 1 | 0.98 | 0.018 | 16 | 0.5075 |
| **374** | tr|C9JVT3|C9JVT3_HUMAN |  | 3 | 1 | 0.018 | 16 | 0.9561 |
| **375** | tr|Q76N53|Q76N53_HUMAN |  | 1 | 0.88 | 0.029 | 16 | 0.01591 |
| **376** | tr|B2RDW0|B2RDW0_HUMAN |  | 10 | 1.08 | 0.01 | 16 | 0.007253 |
| **377** | sp|Q6S8J3|POTEE_HUMAN |  | 1 | 0.85 | 0.116 | 16 | 0.1 |
| **378** | sp|Q9Y2V2|CHSP1_HUMAN |  | 1 | 1.04 | 0.009 | 16 | 0.1281 |
| **379** | tr|Q53FW2|Q53FW2_HUMAN |  | 8 | 1.21 | 0.003 | 16 | 3.63E-10 |
| **380** | tr|Q9UNU2|Q9UNU2_HUMAN |  | 1 | 0.95 | 0.034 | 16 | 0.2695 |
| **381** | sp|P55058|PLTP_HUMAN |  | 9 | 0.93 | 0.016 | 16 | 0.04592 |
| **382** | sp|Q8WWA0|ITLN1_HUMAN |  | 2 | 1.01 | 0.028 | 16 | 0.8224 |
| **383** | sp|Q99733|NP1L4_HUMAN |  | 5 | 0.83 | 0.004 | 16 | 1.51E-08 |
| **384** | sp|P07737|PROF1_HUMAN |  | 2 | 1.18 | 0.043 | 16 | 0.003283 |
| **385** | tr|H3BUM9|H3BUM9_HUMAN |  | 1 | 1.08 | 0.106 | 16 | 0.3394 |
| **386** | sp|Q96FJ2|DYL2_HUMAN |  | 1 | 1.19 | 0.057 | 16 | 0.006288 |
| **387** | tr|D6RC06|D6RC06_HUMAN |  | 1 | 0.94 | 0.017 | 16 | 0.105 |
| **388** | sp|Q9NWU2|GID8_HUMAN |  | 4 | 0.94 | 0.012 | 16 | 0.03246 |
| **389** | tr|M0QXA7|M0QXA7_HUMAN |  | 1 | 0.97 | 0.014 | 16 | 0.2967 |
| **390** | tr|B3KMT7|B3KMT7_HUMAN |  | 1 | 0.91 | 0.072 | 16 | 0.2059 |
| **391** | tr|B2WTI3|B2WTI3_HUMAN |  | 2 | 0.97 | 0.083 | 16 | 0.6701 |
| **392** | tr|X5DR54|X5DR54_HUMAN |  | 6 | 0.89 | 0.013 | 16 | 0.001871 |
| **393** | sp|Q9BQI7|PSD2_HUMAN |  | 1 | 0.93 | 0.071 | 16 | 0.298 |
| **394** | tr|Q8N6B4|Q8N6B4_HUMAN |  | 1 | 1.17 | 0.153 | 16 | 0.09495 |
| **395** | tr|B4DWZ7|B4DWZ7_HUMAN |  | 2 | 1.05 | 0.011 | 16 | 0.06801 |
| **396** | tr|I3L3Y1|I3L3Y1_HUMAN |  | 1 | 0.86 | 0.037 | 16 | 0.01053 |
| **397** | tr|A0A087X225|A0A087X225_HUMAN |  | 1 | 0.97 | 0.046 | 16 | 0.646 |
| **398** | sp|P16152|CBR1_HUMAN |  | 1 | 1.03 | 0.017 | 16 | 0.3085 |
| **399** | sp|O95630|STABP_HUMAN |  | 3 | 1.03 | 0.011 | 16 | 0.2771 |
| **400** | tr|H3BNF0|H3BNF0_HUMAN |  | 4 | 0.89 | 0.005 | 16 | 2.13E-05 |
| **401** | tr|B3KR49|B3KR49_HUMAN |  | 1 | 1.04 | 0.013 | 16 | 0.2106 |
| **402** | sp|Q96P70|IPO9_HUMAN |  | 14 | 0.87 | 0.001 | 16 | 2.26E-10 |
| **403** | sp|O60884|DNJA2_HUMAN |  | 2 | 1.04 | 0.013 | 16 | 0.213 |
| **404** | tr|B2RBZ5|B2RBZ5_HUMAN |  | 1 | 1.13 | 0.133 | 16 | 0.1647 |
| **405** | tr|Q53HU7|Q53HU7_HUMAN |  | 2 | 0.99 | 0.022 | 16 | 0.7783 |
| **406** | tr|H7BZI1|H7BZI1_HUMAN |  | 2 | 0.83 | 0.017 | 16 | 8.49E-05 |
| **407** | sp|Q15172|2A5A_HUMAN |  | 1 | 0.83 | 0.041 | 16 | 0.004951 |
| **408** | tr|H7BZQ3|H7BZQ3_HUMAN |  | 1 | 1.11 | 0.109 | 16 | 0.1937 |
| **409** | sp|O75113|N4BP1_HUMAN |  | 1 | 1.07 | 0.042 | 16 | 0.2122 |
| **410** | tr|B4DHN5|B4DHN5_HUMAN |  | 2 | 0.99 | 0.026 | 16 | 0.7438 |
| **411** | tr|Q6NSF2|Q6NSF2_HUMAN |  | 6 | 0.88 | 0.085 | 16 | 0.1121 |
| **412** | tr|B3KSL3|B3KSL3_HUMAN |  | 1 | 1.13 | 0.192 | 16 | 0.2413 |
| **413** | tr|B4DDZ9|B4DDZ9_HUMAN |  | 1 | 0.97 | 0.087 | 16 | 0.7109 |
| **414** | sp|P51148|RAB5C_HUMAN |  | 2 | 0.99 | 0.006 | 16 | 0.5322 |
| **415** | sp|O00187|MASP2_HUMAN |  | 8 | 1.03 | 0.013 | 16 | 0.3481 |
| **416** | sp|P30613|KPYR_HUMAN |  | 26 | 1.07 | 0.005 | 16 | 0.001069 |
| **417** | tr|B1PL87|B1PL87_HUMAN |  | 3 | 0.92 | 0.019 | 16 | 0.02556 |
| **418** | tr|A0A024R674|A0A024R674_HUMAN |  | 1 | 0.72 | 0.016 | 16 | 2.41E-07 |
| **419** | sp|Q8TDY2|RBCC1_HUMAN |  | 2 | 0.82 | 0.019 | 16 | 0.0001213 |
| **420** | tr|V9HW89|V9HW89_HUMAN |  | 8 | 0.97 | 0.006 | 16 | 0.1311 |
| **421** | tr|Q9BSE8|Q9BSE8_HUMAN |  | 1 | 1.22 | 0.079 | 16 | 0.006071 |
| **422** | tr|Q59FU3|Q59FU3_HUMAN |  | 3 | 0.84 | 0.02 | 16 | 0.0003837 |
| **423** | tr|V9GZ55|V9GZ55_HUMAN |  | 1 | 0.95 | 0.056 | 16 | 0.4035 |
| **424** | tr|B7Z6M7|B7Z6M7_HUMAN |  | 1 | 0.97 | 0.091 | 16 | 0.6767 |
| **425** | sp|Q9NRN7|ADPPT_HUMAN |  | 1 | 0.9 | 0.016 | 16 | 0.007899 |
| **426** | tr|Q70T18|Q70T18_HUMAN |  | 1 | 0.89 | 0.031 | 16 | 0.02501 |
| **427** | tr|H0YMZ1|H0YMZ1_HUMAN |  | 11 | 1.07 | 0.004 | 16 | 0.0002376 |
| **428** | sp|Q13616|CUL1_HUMAN |  | 15 | 1.09 | 0.003 | 16 | 4.68E-06 |
| **429** | tr|H3BTI9|H3BTI9_HUMAN |  | 1 | 0.9 | 0.026 | 16 | 0.02881 |
| **430** | sp|P02760|AMBP_HUMAN |  | 5 | 1.02 | 0.004 | 16 | 0.2163 |
| **431** | tr|E5RI09|E5RI09_HUMAN |  | 1 | 1.01 | 0.072 | 16 | 0.8414 |
| **432** | tr|D6RGZ3|D6RGZ3_HUMAN |  | 1 | 0.87 | 0.029 | 16 | 0.008737 |
| **433** | tr|H0YJ31|H0YJ31_HUMAN |  | 5 | 0.88 | 0.017 | 16 | 0.002304 |
| **434** | sp|Q9Y3Z3|SAMH1_HUMAN |  | 2 | 1.12 | 0.058 | 16 | 0.05929 |
| **435** | tr|Q5T6W5|Q5T6W5_HUMAN |  | 11 | 0.88 | 0.004 | 16 | 1.90E-06 |
| **436** | sp|O60664|PLIN3_HUMAN |  | 7 | 1.09 | 0.026 | 16 | 0.04895 |
| **437** | sp|Q8WW22|DNJA4_HUMAN |  | 1 | 0.97 | 0.009 | 16 | 0.1895 |
| **438** | tr|Q53FW9|Q53FW9_HUMAN |  | 5 | 0.95 | 0.014 | 16 | 0.1283 |
| **439** | sp|Q6UWE0|LRSM1_HUMAN |  | 1 | 1.06 | 0.119 | 16 | 0.5084 |
| **440** | tr|E7EU96|E7EU96_HUMAN |  | 11 | 0.92 | 0.007 | 16 | 0.001529 |
| **441** | sp|P62312|LSM6_HUMAN |  | 1 | 1.26 | 0.084 | 16 | 0.00262 |
| **442** | tr|Q53HU9|Q53HU9_HUMAN |  | 21 | 0.97 | 0.008 | 16 | 0.2551 |
| **443** | tr|H7C492|H7C492_HUMAN |  | 1 | 0.79 | 0.033 | 16 | 0.000403 |
| **444** | tr|D6RC22|D6RC22_HUMAN |  | 1 | 1.16 | 0.076 | 16 | 0.03419 |
| **445** | sp|Q9HA65|TBC17_HUMAN |  | 6 | 0.88 | 0.009 | 16 | 0.000234 |
| **446** | sp|P12004|PCNA_HUMAN |  | 1 | 1.03 | 0.03 | 16 | 0.53 |
| **447** | sp|Q9BRP4|PAAF1_HUMAN |  | 1 | 1.02 | 0.009 | 16 | 0.4135 |
| **448** | tr|E5RG67|E5RG67_HUMAN |  | 1 | 1.02 | 0.052 | 16 | 0.7537 |
| **449** | sp|P30043|BLVRB_HUMAN |  | 9 | 0.98 | 0.03 | 16 | 0.6696 |
| **450** | tr|B4DYU0|B4DYU0_HUMAN |  | 10 | 0.98 | 0.013 | 16 | 0.5505 |
| **451** | tr|V9GZ38|V9GZ38_HUMAN |  | 1 | 1.04 | 0.071 | 16 | 0.5404 |
| **452** | tr|C9J6W9|C9J6W9_HUMAN |  | 1 | 0.99 | 0.102 | 16 | 0.8655 |
| **453** | sp|A6NDG6|PGP_HUMAN |  | 9 | 0.92 | 0.002 | 16 | 5.93E-06 |
| **454** | sp|P48444|COPD_HUMAN |  | 4 | 1.02 | 0.01 | 16 | 0.3594 |
| **455** | sp|Q9UK55|ZPI_HUMAN |  | 1 | 0.9 | 0.041 | 16 | 0.06883 |
| **456** | tr|B4DSD8|B4DSD8_HUMAN |  | 4 | 0.98 | 0.051 | 16 | 0.6909 |
| **457** | tr|A0A024QZJ7|A0A024QZJ7_HUMAN |  | 9 | 0.95 | 0.01 | 16 | 0.08284 |
| **458** | sp|P03951|FA11_HUMAN |  | 2 | 0.92 | 0.021 | 16 | 0.04568 |
| **459** | tr|A0A024R1G6|A0A024R1G6_HUMAN |  | 2 | 0.97 | 0.017 | 16 | 0.3441 |
| **460** | sp|Q92625|ANS1A_HUMAN |  | 1 | 0.91 | 0.225 | 16 | 0.474 |
| **461** | sp|P55769|NH2L1_HUMAN |  | 1 | 1.29 | 0.625 | 16 | 0.1579 |
| **462** | sp|P69891|HBG1_HUMAN |  | 1 | 1.2 | 0.149 | 16 | 0.05619 |
| **463** | tr|B0QYH3|B0QYH3_HUMAN |  | 2 | 1.01 | 0.011 | 16 | 0.8107 |
| **464** | tr|E5RGM0|E5RGM0_HUMAN |  | 1 | 0.95 | 0.036 | 16 | 0.284 |
| **465** | tr|Q8N210|Q8N210_HUMAN |  | 1 | 1.14 | 0.059 | 16 | 0.03468 |
| **466** | sp|P07437|TBB5_HUMAN |  | 2 | 0.99 | 0.007 | 16 | 0.6177 |
| **467** | tr|F8WAR7|F8WAR7_HUMAN |  | 1 | 0.95 | 0.078 | 16 | 0.4847 |
| **468** | tr|Q59FA7|Q59FA7_HUMAN |  | 3 | 1.03 | 0.004 | 16 | 0.07411 |
| **469** | sp|P01008|ANT3_HUMAN |  | 15 | 1.04 | 0.104 | 16 | 0.5939 |
| **470** | tr|B7Z5N4|B7Z5N4_HUMAN |  | 2 | 1.16 | 0.011 | 16 | 1.89E-05 |
| **471** | tr|B4E2V5|B4E2V5_HUMAN |  | 7 | 0.82 | 0.03 | 16 | 0.0007149 |
| **472** | sp|Q9Y315|DEOC_HUMAN |  | 5 | 1.06 | 0.005 | 16 | 0.004107 |
| **473** | sp|Q6UXB2|VCC1_HUMAN |  | 1 | 0.54 | 0.047 | 16 | 4.20E-07 |
| **474** | tr|B4DHW4|B4DHW4_HUMAN |  | 3 | 0.91 | 0.008 | 16 | 0.001672 |
| **475** | sp|P53004|BIEA_HUMAN |  | 5 | 0.8 | 0.038 | 16 | 0.0008497 |
| **476** | sp|P35241|RADI_HUMAN |  | 3 | 0.99 | 0.013 | 16 | 0.7572 |
| **477** | sp|P23396|RS3_HUMAN |  | 6 | 1 | 0.003 | 16 | 0.8226 |
| **478** | sp|O14980|XPO1_HUMAN |  | 4 | 0.94 | 0.001 | 16 | 2.81E-06 |
| **479** | tr|B4DH19|B4DH19_HUMAN |  | 17 | 1.09 | 0.034 | 16 | 0.07813 |
| **480** | sp|O00487|PSDE_HUMAN |  | 7 | 1.01 | 0.004 | 16 | 0.4663 |
| **481** | tr|B4DL66|B4DL66_HUMAN |  | 1 | 0.93 | 0.032 | 16 | 0.1178 |
| **482** | sp|P0C0L4|CO4A_HUMAN |  | 35 | 1.13 | 0.009 | 16 | 4.06E-05 |
| **483** | tr|D6R9W4|D6R9W4_HUMAN |  | 1 | 0.83 | 0.009 | 16 | 4.96E-06 |
| **484** | sp|Q6ZT12|UBR3_HUMAN |  | 1 | 1.1 | 0.038 | 16 | 0.0662 |
| **485** | sp|P56537|IF6_HUMAN |  | 5 | 1.1 | 0.008 | 16 | 0.0005101 |
| **486** | sp|Q8WXX5|DNJC9_HUMAN |  | 3 | 1.19 | 0.039 | 16 | 0.001372 |
| **487** | tr|K7EQA1|K7EQA1_HUMAN |  | 2 | 0.93 | 0.054 | 16 | 0.2243 |
| **488** | tr|Q05CK9|Q05CK9_HUMAN |  | 8 | 0.79 | 0.006 | 16 | 8.41E-09 |
| **489** | tr|Q53FH8|Q53FH8_HUMAN |  | 4 | 0.97 | 0.002 | 16 | 0.01293 |
| **490** | tr|E5RI99|E5RI99_HUMAN |  | 1 | 0.91 | 0.038 | 16 | 0.09849 |
| **491** | tr|M0R370|M0R370_HUMAN |  | 1 | 1.08 | 0.025 | 16 | 0.05847 |
| **492** | tr|Q29988|Q29988_HUMAN |  | 1 | 1.16 | 0.089 | 16 | 0.05371 |
| **493** | tr|F5GZF7|F5GZF7_HUMAN |  | 1 | 1.11 | 0.098 | 15 | 0.199 |
| **494** | sp|Q86VX9|MON1A_HUMAN |  | 1 | 1.05 | 0.735 | 16 | 0.8256 |
| **495** | sp|P30041|PRDX6_HUMAN |  | 17 | 0.9 | 0.026 | 16 | 0.02194 |
| **496** | sp|Q6PCE3|PGM2L_HUMAN |  | 9 | 0.88 | 0.007 | 16 | 4.12E-05 |
| **497** | tr|Q0D2N5|Q0D2N5_HUMAN |  | 1 | 0.91 | 0.05 | 16 | 0.1239 |
| **498** | tr|E7EX73|E7EX73_HUMAN |  | 10 | 1.12 | 0.008 | 16 | 8.88E-05 |
| **499** | sp|Q9H496|IFG15_HUMAN |  | 2 | 0.86 | 0.009 | 16 | 2.82E-05 |
| **500** | sp|Q13011|ECH1_HUMAN |  | 1 | 1.13 | 0.182 | 16 | 0.2435 |
| **501** | sp|P05089|ARGI1_HUMAN |  | 2 | 0.85 | 0.062 | 16 | 0.03246 |
| **502** | sp|Q9UNS2|CSN3_HUMAN |  | 7 | 0.98 | 0.004 | 16 | 0.1376 |
| **503** | tr|B4DRG7|B4DRG7_HUMAN |  | 3 | 1.01 | 0.02 | 16 | 0.6952 |
| **504** | sp|Q8IY17|PLPL6_HUMAN |  | 1 | 0.88 | 0.058 | 16 | 0.05942 |
| **505** | sp|Q9UPN7|PP6R1_HUMAN |  | 10 | 0.9 | 0.003 | 16 | 2.79E-06 |
| **506** | tr|A0A0A0MTP3|A0A0A0MTP3_HUMAN |  | 2 | 0.71 | 0.024 | 16 | 1.96E-06 |
| **507** | sp|Q63HN8|RN213_HUMAN |  | 1 | 0.79 | 0.028 | 16 | 0.0002068 |
| **508** | tr|E9PI38|E9PI38_HUMAN |  | 3 | 0.95 | 0.021 | 16 | 0.205 |
| **509** | tr|E7EPJ1|E7EPJ1_HUMAN |  | 1 | 1.06 | 0.033 | 16 | 0.1854 |
| **510** | tr|A2J1M8|A2J1M8_HUMAN |  | 1 | 0.79 | 0.097 | 16 | 0.016 |
| **511** | tr|K7ER46|K7ER46_HUMAN |  | 1 | 0.75 | 0.047 | 16 | 0.0003288 |
| **512** | sp|Q9NY12|GAR1_HUMAN |  | 1 | 1.55 | 0.059 | 16 | 1.59E-07 |
| **513** | tr|B5MDQ0|B5MDQ0_HUMAN |  | 1 | 1.11 | 0.106 | 16 | 0.2042 |
| **514** | sp|P30273|FCERG_HUMAN |  | 1 | 0.86 | 0.029 | 16 | 0.006024 |
| **515** | sp|P20339|RAB5A_HUMAN |  | 2 | 1.05 | 0.046 | 16 | 0.4101 |
| **516** | tr|B1N7B6|B1N7B6_HUMAN |  | 1 | 0.89 | 0.031 | 16 | 0.02757 |
| **517** | tr|Q5NV70|Q5NV70_HUMAN |  | 1 | 1.08 | 0.246 | 16 | 0.5295 |
| **518** | tr|H7C540|H7C540_HUMAN |  | 1 | 1.31 | 0.008 | 16 | 4.75E-10 |
| **519** | sp|P81605|DCD_HUMAN |  | 1 | 1.12 | 0.058 | 16 | 0.06596 |
| **520** | tr|S6BGF9|S6BGF9_HUMAN |  | 1 | 1.08 | 0.038 | 16 | 0.1361 |
| **521** | tr|Q5CAQ5|Q5CAQ5_HUMAN |  | 19 | 1.03 | 0.009 | 16 | 0.3043 |
| **522** | tr|F5H5I5|F5H5I5_HUMAN |  | 1 | 0.97 | 0.009 | 16 | 0.1795 |
| **523** | sp|P48729|KC1A_HUMAN |  | 5 | 0.97 | 0.007 | 16 | 0.2424 |
| **524** | sp|Q9Y6Z7|COL10_HUMAN |  | 2 | 0.89 | 0.02 | 16 | 0.005757 |
| **525** | tr|A8K4G7|A8K4G7_HUMAN |  | 1 | 1.06 | 0.028 | 16 | 0.1542 |
| **526** | tr|J3QLE5|J3QLE5_HUMAN |  | 1 | 1.88 | 0.038 | 16 | 1.49E-11 |
| **527** | tr|B4DR80|B4DR80_HUMAN |  | 7 | 0.95 | 0.004 | 16 | 0.002708 |
| **528** | sp|P08238|HS90B_HUMAN |  | 5 | 1.16 | 0.007 | 16 | 1.56E-06 |
| **529** | tr|B4DDV4|B4DDV4_HUMAN |  | 1 | 0.95 | 0.056 | 16 | 0.4235 |
| **530** | tr|X6RFL8|X6RFL8_HUMAN |  | 4 | 1.13 | 0.011 | 16 | 0.0001601 |
| **531** | tr|E9PI78|E9PI78_HUMAN |  | 1 | 0.76 | 0.006 | 8 | 4.79E-05 |
| **532** | tr|A2VCT2|A2VCT2_HUMAN |  | 1 | 1.27 | 0.046 | 16 | 0.0001319 |
| **533** | sp|O60763|USO1_HUMAN |  | 16 | 0.95 | 0.002 | 16 | 0.001768 |
| **534** | sp|P00747|PLMN_HUMAN |  | 22 | 1.04 | 0.003 | 16 | 0.02781 |
| **535** | tr|A0A024R258|A0A024R258_HUMAN |  | 2 | 0.97 | 0.019 | 16 | 0.4235 |
| **536** | sp|P36959|GMPR1_HUMAN |  | 2 | 0.96 | 0.015 | 16 | 0.181 |
| **537** | tr|B3KWP7|B3KWP7_HUMAN |  | 1 | 1.28 | 0.04 | 16 | 4.03E-05 |
| **538** | tr|H0Y360|H0Y360_HUMAN |  | 2 | 1.29 | 0.041 | 16 | 4.02E-05 |
| **539** | sp|O43617|TPPC3_HUMAN |  | 2 | 0.91 | 0.021 | 16 | 0.02433 |
| **540** | tr|L7RSM2|L7RSM2_HUMAN |  | 2 | 1.23 | 0.018 | 16 | 5.68E-06 |
| **541** | sp|P61626|LYSC_HUMAN |  | 7 | 1.33 | 0.035 | 16 | 4.75E-06 |
| **542** | tr|B4DKZ2|B4DKZ2_HUMAN |  | 8 | 0.98 | 0.026 | 16 | 0.6317 |
| **543** | tr|A8K9K1|A8K9K1_HUMAN |  | 4 | 0.88 | 0.025 | 16 | 0.01007 |
| **544** | sp|P51570|GALK1_HUMAN |  | 2 | 0.9 | 0.004 | 16 | 1.68E-05 |
| **545** | tr|J3KS22|J3KS22_HUMAN |  | 9 | 1.02 | 0.006 | 16 | 0.4301 |
| **546** | tr|Q8N4L6|Q8N4L6_HUMAN |  | 1 | 0.83 | 0.038 | 16 | 0.003985 |
| **547** | tr|Q6ZRN6|Q6ZRN6_HUMAN |  | 1 | 0.72 | 0.082 | 16 | 0.001376 |
| **548** | sp|O95147|DUS14_HUMAN |  | 1 | 1.02 | 0.015 | 16 | 0.5161 |
| **549** | tr|B4E3A7|B4E3A7_HUMAN |  | 1 | 1.21 | 0.067 | 16 | 0.005753 |
| **550** | tr|J3QLC6|J3QLC6_HUMAN |  | 1 | 0.85 | 0.025 | 16 | 0.001479 |
| **551** | tr|Q6GMX6|Q6GMX6_HUMAN |  | 1 | 0.75 | 0.034 | 16 | 8.17E-05 |
| **552** | tr|A1L305|A1L305_HUMAN |  | 1 | 1.18 | 0.053 | 16 | 0.006806 |
| **553** | sp|P19623|SPEE_HUMAN |  | 2 | 1.06 | 0.011 | 16 | 0.0488 |
| **554** | tr|A0A0A0MQR8|A0A0A0MQR8_HUMAN |  | 2 | 0.99 | 0.011 | 16 | 0.7014 |
| **555** | sp|Q27J81|INF2_HUMAN |  | 7 | 0.91 | 0.03 | 16 | 0.06898 |
| **556** | tr|A0A087WTE0|A0A087WTE0_HUMAN |  | 1 | 0.92 | 0.35 | 16 | 0.6095 |
| **557** | sp|Q96M27|PRRC1_HUMAN |  | 2 | 1.16 | 0.023 | 16 | 0.0008294 |
| **558** | tr|B0QYW5|B0QYW5_HUMAN |  | 1 | 0.86 | 0.033 | 16 | 0.006778 |
| **559** | sp|P35606|COPB2_HUMAN |  | 2 | 1.2 | 0.005 | 16 | 1.49E-08 |
| **560** | tr|B4DMZ5|B4DMZ5_HUMAN |  | 2 | 0.91 | 0.013 | 16 | 0.00626 |
| **561** | tr|H0YA27|H0YA27_HUMAN |  | 1 | 1.31 | 0.029 | 16 | 2.33E-06 |
| **562** | tr|A8K9A9|A8K9A9_HUMAN |  | 8 | 1.02 | 0.025 | 16 | 0.5648 |
| **563** | tr|E9PEW8|E9PEW8_HUMAN |  | 1 | 0.89 | 0.025 | 16 | 0.01495 |
| **564** | sp|P11678|PERE_HUMAN |  | 4 | 1.72 | 0.16 | 16 | 2.99E-06 |
| **565** | tr|A2JA14|A2JA14_HUMAN |  | 1 | 0.68 | 0.013 | 16 | 1.04E-08 |
| **566** | sp|P62304|RUXE_HUMAN |  | 1 | 1.39 | 0.123 | 16 | 0.0004667 |
| **567** | sp|P61088|UBE2N_HUMAN |  | 3 | 0.95 | 0.09 | 16 | 0.5073 |
| **568** | tr|M0QXC8|M0QXC8_HUMAN |  | 2 | 1.06 | 0.005 | 16 | 0.004421 |
| **569** | sp|Q9UNN5|FAF1_HUMAN |  | 14 | 1.05 | 0.002 | 16 | 0.000705 |
| **570** | tr|K7ENG2|K7ENG2_HUMAN |  | 1 | 1.17 | 0.091 | 16 | 0.03841 |
| **571** | sp|O43866|CD5L_HUMAN |  | 13 | 1.02 | 0.049 | 16 | 0.6798 |
| **572** | tr|B4DNZ4|B4DNZ4_HUMAN |  | 1 | 1.01 | 0.113 | 16 | 0.9452 |
| **573** | tr|B4DUL0|B4DUL0_HUMAN |  | 1 | 0.89 | 0.026 | 16 | 0.01284 |
| **574** | tr|A0A087X0I9|A0A087X0I9_HUMAN |  | 1 | 0.88 | 0.01 | 16 | 0.0001944 |
| **575** | tr|Q8WVW5|Q8WVW5_HUMAN |  | 4 | 1.22 | 0.015 | 16 | 3.69E-06 |
| **576** | tr|B7Z6S0|B7Z6S0_HUMAN |  | 2 | 1.02 | 0.015 | 16 | 0.5872 |
| **577** | tr|Q9UK54|Q9UK54_HUMAN |  | 1 | 1.47 | 0.135 | 16 | 0.0001257 |
| **578** | sp|Q8IYU2|HACE1_HUMAN |  | 2 | 1.05 | 0.055 | 16 | 0.3853 |
| **579** | tr|I3L3E6|I3L3E6_HUMAN |  | 1 | 0.84 | 0.074 | 16 | 0.03302 |
| **580** | tr|F5H7C6|F5H7C6_HUMAN |  | 7 | 0.94 | 0.005 | 16 | 0.002491 |
| **581** | sp|P02549|SPTA1_HUMAN |  | 81 | 0.84 | 0.005 | 16 | 1.44E-07 |
| **582** | tr|K7EJT5|K7EJT5_HUMAN |  | 1 | 0.93 | 0.006 | 16 | 0.00348 |
| **583** | tr|Q86U57|Q86U57_HUMAN |  | 1 | 1.12 | 0.172 | 16 | 0.2516 |
| **584** | sp|Q6DN90|IQEC1_HUMAN |  | 3 | 0.97 | 0.019 | 16 | 0.4087 |
| **585** | sp|Q9H4M9|EHD1_HUMAN |  | 12 | 1.05 | 0.001 | 16 | 0.0001365 |
| **586** | tr|K7ES31|K7ES31_HUMAN |  | 1 | 1.03 | 0.052 | 16 | 0.6241 |
| **587** | tr|B2RMS9|B2RMS9_HUMAN |  | 19 | 1.08 | 0.014 | 16 | 0.01176 |
| **588** | tr|E9PJX0|E9PJX0_HUMAN |  | 1 | 1.56 | 0.148 | 16 | 3.51E-05 |
| **589** | tr|A8K2S1|A8K2S1_HUMAN |  | 2 | 0.82 | 0.014 | 16 | 1.98E-05 |
| **590** | tr|B4DYP5|B4DYP5_HUMAN |  | 8 | 1 | 0.006 | 16 | 0.8326 |
| **591** | tr|A0A087WUQ6|A0A087WUQ6_HUMAN |  | 7 | 1.08 | 0.007 | 16 | 0.00249 |
| **592** | sp|Q9H479|FN3K_HUMAN |  | 10 | 1.09 | 0.018 | 16 | 0.01218 |
| **593** | tr|A2VDJ4|A2VDJ4_HUMAN |  | 1 | 1.04 | 0.029 | 16 | 0.3428 |
| **594** | sp|Q06323|PSME1_HUMAN |  | 12 | 1.04 | 0.05 | 16 | 0.4876 |
| **595** | tr|B3KQ05|B3KQ05_HUMAN |  | 1 | 1.12 | 0.04 | 16 | 0.03008 |
| **596** | sp|P62805|H4_HUMAN |  | 6 | 4.07 | 0.311 | 16 | 7.67E-13 |
| **597** | sp|P57772|SELB_HUMAN |  | 6 | 1.07 | 0.008 | 16 | 0.00862 |
| **598** | sp|Q9H871|RMD5A_HUMAN |  | 2 | 1 | 0.05 | 16 | 0.9395 |
| **599** | tr|A0A087X1V9|A0A087X1V9_HUMAN |  | 1 | 0.93 | 0.013 | 16 | 0.02136 |
| **600** | tr|A2MYC8|A2MYC8_HUMAN |  | 1 | 1.13 | 0.113 | 16 | 0.1351 |
| **601** | tr|B2R5G8|B2R5G8_HUMAN |  | 1 | 1.28 | 0.074 | 16 | 0.0007963 |
| **602** | tr|B5MD47|B5MD47_HUMAN |  | 1 | 1.12 | 0.386 | 16 | 0.4372 |
| **603** | sp|P78371|TCPB_HUMAN |  | 28 | 0.91 | 0.008 | 16 | 0.001069 |
| **604** | tr|B3KX77|B3KX77_HUMAN |  | 1 | 0.98 | 0.082 | 16 | 0.7914 |
| **605** | tr|C9IZD3|C9IZD3_HUMAN |  | 2 | 1.06 | 0.014 | 16 | 0.04622 |
| **606** | tr|A0A087WY81|A0A087WY81_HUMAN |  | 1 | 0.97 | 0.113 | 16 | 0.6983 |
| **607** | tr|B3KRA8|B3KRA8_HUMAN |  | 2 | 1.12 | 0.019 | 16 | 0.002894 |
| **608** | tr|B4DEA3|B4DEA3_HUMAN |  | 6 | 1.19 | 0.012 | 16 | 5.73E-06 |
| **609** | tr|B4DXH2|B4DXH2_HUMAN |  | 1 | 1 | 0.07 | 16 | 0.9799 |
| **610** | tr|B4DIU3|B4DIU3_HUMAN |  | 2 | 0.96 | 0.037 | 16 | 0.391 |
| **611** | sp|Q15113|PCOC1_HUMAN |  | 6 | 0.98 | 0.002 | 16 | 0.03173 |
| **612** | tr|Q5JQQ4|Q5JQQ4_HUMAN |  | 1 | 0.95 | 0.051 | 16 | 0.4337 |
| **613** | sp|Q8NB15|ZN511_HUMAN |  | 1 | 0.83 | 0.031 | 16 | 0.001744 |
| **614** | tr|D6RA00|D6RA00_HUMAN |  | 1 | 1.07 | 0.016 | 16 | 0.0523 |
| **615** | sp|Q99832|TCPH_HUMAN |  | 23 | 0.91 | 0.001 | 16 | 1.13E-09 |
| **616** | sp|O95782|AP2A1_HUMAN |  | 20 | 0.93 | 0.006 | 16 | 0.004286 |
| **617** | sp|P02042|HBD_HUMAN |  | 3 | 0.84 | 0.027 | 16 | 0.001646 |
| **618** | tr|A0A024R8I2|A0A024R8I2_HUMAN |  | 23 | 0.98 | 0.001 | 16 | 0.05574 |
| **619** | tr|B7Z2K7|B7Z2K7_HUMAN |  | 4 | 1.03 | 0.012 | 16 | 0.2299 |
| **620** | sp|P62140|PP1B_HUMAN |  | 1 | 1.04 | 0.039 | 16 | 0.4144 |
| **621** | tr|H0Y6F2|H0Y6F2_HUMAN |  | 1 | 0.82 | 0.018 | 16 | 7.99E-05 |
| **622** | sp|P05771|KPCB_HUMAN |  | 2 | 0.98 | 0.03 | 16 | 0.6096 |
| **623** | tr|E9PBS1|E9PBS1_HUMAN |  | 10 | 1.01 | 0.008 | 16 | 0.8135 |
| **624** | tr|Q6N091|Q6N091_HUMAN |  | 1 | 0.85 | 0.015 | 16 | 0.0001964 |
| **625** | tr|H7C2Z6|H7C2Z6_HUMAN |  | 2 | 1.36 | 0.133 | 16 | 0.001264 |
| **626** | sp|A6NDU8|CE051_HUMAN |  | 2 | 1.01 | 0.016 | 16 | 0.7853 |
| **627** | tr|H3BUN9|H3BUN9_HUMAN |  | 1 | 1.17 | 0.082 | 16 | 0.03049 |
| **628** | tr|B3KN59|B3KN59_HUMAN |  | 1 | 0.91 | 0.005 | 16 | 9.10E-05 |
| **629** | tr|E7EMC6|E7EMC6_HUMAN |  | 3 | 1.41 | 0.069 | 16 | 1.54E-05 |
| **630** | tr|A0A024R862|A0A024R862_HUMAN |  | 29 | 0.93 | 0.002 | 16 | 1.38E-05 |
| **631** | tr|Q6VFQ6|Q6VFQ6_HUMAN |  | 1 | 0.88 | 0.031 | 16 | 0.0133 |
| **632** | tr|D6RGE2|D6RGE2_HUMAN |  | 3 | 0.74 | 0.011 | 16 | 6.40E-08 |
| **633** | sp|Q8WUM4|PDC6I_HUMAN |  | 22 | 0.99 | 0.127 | 16 | 0.9241 |
| **634** | sp|Q4G0F5|VP26B_HUMAN |  | 4 | 1.08 | 0.007 | 16 | 0.002264 |
| **635** | sp|P33897|ABCD1_HUMAN |  | 1 | 0.97 | 0.018 | 16 | 0.4449 |
| **636** | tr|B1ANH0|B1ANH0_HUMAN |  | 4 | 0.89 | 0.065 | 16 | 0.09415 |
| **637** | tr|Q7Z6G4|Q7Z6G4_HUMAN |  | 1 | 0.76 | 0.009 | 16 | 4.45E-08 |
| **638** | tr|H0Y512|H0Y512_HUMAN |  | 5 | 1.2 | 0.026 | 16 | 0.0001748 |
| **639** | sp|Q96GD0|PLPP_HUMAN |  | 6 | 0.91 | 0.021 | 16 | 0.02079 |
| **640** | tr|A0A0A0MRE5|A0A0A0MRE5_HUMAN |  | 7 | 0.88 | 0.006 | 16 | 1.84E-05 |
| **641** | tr|Q53GN8|Q53GN8_HUMAN |  | 4 | 1 | 0.024 | 16 | 0.9176 |
| **642** | tr|H7BY16|H7BY16_HUMAN |  | 1 | 1.15 | 0.012 | 16 | 6.16E-05 |
| **643** | sp|Q92878|RAD50_HUMAN |  | 2 | 0.94 | 0.011 | 16 | 0.05263 |
| **644** | tr|A2NW98|A2NW98_HUMAN |  | 1 | 0.88 | 0.047 | 16 | 0.04801 |
| **645** | tr|A6PVN7|A6PVN7_HUMAN |  | 1 | 0.89 | 0.044 | 16 | 0.06102 |
| **646** | sp|O75594|PGRP1_HUMAN |  | 1 | 1.18 | 0.049 | 16 | 0.004508 |
| **647** | sp|Q9H939|PPIP2_HUMAN |  | 1 | 1.06 | 0.015 | 16 | 0.07243 |
| **648** | tr|M0QX44|M0QX44_HUMAN |  | 1 | 0.52 | 0.067 | 16 | 1.98E-06 |
| **649** | tr|A0PJ62|A0PJ62_HUMAN |  | 1 | 1.21 | 0.068 | 16 | 0.005595 |
| **650** | tr|B7Z1F8|B7Z1F8_HUMAN |  | 2 | 1.07 | 0.037 | 16 | 0.1568 |
| **651** | sp|Q8N0W3|FUK_HUMAN |  | 12 | 0.97 | 0.008 | 16 | 0.1748 |
| **652** | tr|K7EKA8|K7EKA8_HUMAN |  | 1 | 1.24 | 0.848 | 16 | 0.3083 |
| **653** | tr|F8WD05|F8WD05_HUMAN |  | 1 | 1.16 | 0.241 | 16 | 0.2143 |
| **654** | tr|E9PKY5|E9PKY5_HUMAN |  | 1 | 1.18 | 0.074 | 16 | 0.01672 |
| **655** | sp|P01743|HV102_HUMAN |  | 1 | 0.92 | 0.03 | 16 | 0.07067 |
| **656** | tr|Q96GX3|Q96GX3_HUMAN |  | 2 | 0.93 | 0.022 | 16 | 0.06192 |
| **657** | sp|Q9HCJ0|TNR6C_HUMAN |  | 1 | 1.15 | 0.121 | 16 | 0.09818 |
| **658** | sp|P50591|TNF10_HUMAN |  | 1 | 1.19 | 0.104 | 16 | 0.02892 |
| **659** | sp|P10599|THIO_HUMAN |  | 3 | 1.07 | 0.11 | 16 | 0.3925 |
| **660** | tr|B3VL31|B3VL31_HUMAN |  | 3 | 0.97 | 0.043 | 16 | 0.5354 |
| **661** | tr|A0A087WXL8|A0A087WXL8_HUMAN |  | 1 | 1.1 | 0.018 | 16 | 0.01096 |
| **662** | tr|A0A087X0Q9|A0A087X0Q9_HUMAN |  | 10 | 0.98 | 0.005 | 16 | 0.2838 |
| **663** | sp|P53621|COPA_HUMAN |  | 4 | 1.16 | 0.017 | 16 | 0.0001623 |
| **664** | tr|B2R7R5|B2R7R5_HUMAN |  | 9 | 1.18 | 0.004 | 16 | 1.66E-08 |
| **665** | tr|B4DF70|B4DF70_HUMAN |  | 9 | 0.83 | 0.056 | 16 | 0.01257 |
| **666** | sp|Q8NGV0|OR2Y1_HUMAN |  | 1 | 1 | 0.035 | 16 | 0.9411 |
| **667** | tr|C9JW46|C9JW46_HUMAN |  | 1 | 0.97 | 0.053 | 16 | 0.6044 |
| **668** | tr|K7ERY7|K7ERY7_HUMAN |  | 1 | 1.08 | 0.015 | 16 | 0.01904 |
| **669** | tr|Q8TAF6|Q8TAF6_HUMAN |  | 5 | 1.07 | 0.015 | 16 | 0.0507 |
| **670** | sp|Q7Z5V6|PPR32_HUMAN |  | 1 | 0.85 | 0.015 | 16 | 0.0002804 |
| **671** | sp|Q76LX8|ATS13_HUMAN |  | 8 | 0.93 | 0.008 | 16 | 0.005256 |
| **672** | sp|Q9NQE9|HINT3_HUMAN |  | 1 | 0.87 | 0.034 | 16 | 0.0145 |
| **673** | tr|B4E318|B4E318_HUMAN |  | 1 | 0.68 | 0.076 | 16 | 0.0002868 |
| **674** | sp|Q8IXQ3|CI040_HUMAN |  | 1 | 1.48 | 0.097 | 16 | 1.77E-05 |
| **675** | tr|H0YGS7|H0YGS7_HUMAN |  | 2 | 0.98 | 0.029 | 16 | 0.7002 |
| **676** | tr|G1UI32|G1UI32_HUMAN |  | 1 | 1.11 | 0.126 | 16 | 0.2257 |
| **677** | tr|B4E0S6|B4E0S6_HUMAN |  | 4 | 1.12 | 0.031 | 16 | 0.02002 |
| **678** | tr|H0YBG6|H0YBG6_HUMAN |  | 2 | 0.85 | 0.024 | 16 | 0.001224 |
| **679** | sp|Q96IV0|NGLY1_HUMAN |  | 2 | 1 | 0.033 | 16 | 0.9162 |
| **680** | tr|I3L239|I3L239_HUMAN |  | 1 | 1.17 | 0.015 | 16 | 3.98E-05 |
| **681** | sp|Q58WW2|DCAF6_HUMAN |  | 20 | 0.95 | 0.005 | 16 | 0.01818 |
| **682** | sp|P61201|CSN2_HUMAN |  | 15 | 0.92 | 0.003 | 16 | 2.91E-05 |
| **683** | sp|Q96QR8|PURB_HUMAN |  | 1 | 1 | 0.22 | 14 | 0.9786 |
| **684** | sp|Q92496|FHR4_HUMAN |  | 1 | 0.89 | 0.025 | 16 | 0.01206 |
| **685** | tr|Q16441|Q16441_HUMAN |  | 1 | 1.01 | 0.117 | 16 | 0.9181 |
| **686** | sp|Q9NR46|SHLB2_HUMAN |  | 10 | 0.9 | 0.005 | 16 | 1.82E-05 |
| **687** | tr|A0A087WUA5|A0A087WUA5_HUMAN |  | 1 | 0.94 | 0.099 | 16 | 0.4622 |
| **688** | tr|Q2HIY3|Q2HIY3_HUMAN |  | 1 | 1.27 | 0.057 | 16 | 0.0004426 |
| **689** | sp|P55103|INHBC_HUMAN |  | 3 | 0.98 | 0.029 | 16 | 0.6979 |
| **690** | tr|H0Y2Y8|H0Y2Y8_HUMAN |  | 5 | 1.04 | 0.008 | 16 | 0.1136 |
| **691** | tr|C9JF62|C9JF62_HUMAN |  | 1 | 0.89 | 0.075 | 16 | 0.1297 |
| **692** | tr|H7BZH1|H7BZH1_HUMAN |  | 1 | 0.98 | 0.056 | 16 | 0.7147 |
| **693** | tr|E5RGS4|E5RGS4_HUMAN |  | 1 | 1.11 | 0.046 | 16 | 0.05261 |
| **694** | tr|Q5JP02|Q5JP02_HUMAN |  | 2 | 1.03 | 0.01 | 16 | 0.2652 |
| **695** | tr|H7C169|H7C169_HUMAN |  | 1 | 0.79 | 0.032 | 16 | 0.0002884 |
| **696** | sp|P16452|EPB42_HUMAN |  | 7 | 0.74 | 0.019 | 16 | 1.49E-06 |
| **697** | sp|Q5SYC1|CLVS2_HUMAN |  | 1 | 2.42 | 14.665 | 16 | 0.1578 |
| **698** | tr|E5RGA2|E5RGA2_HUMAN |  | 2 | 0.92 | 0.017 | 16 | 0.03056 |
| **699** | tr|B1AR62|B1AR62_HUMAN |  | 1 | 1.1 | 0.053 | 16 | 0.1062 |
| **700** | sp|Q96NA2|RILP_HUMAN |  | 14 | 0.97 | 0.002 | 16 | 0.008439 |
| **701** | sp|P46109|CRKL_HUMAN |  | 3 | 1.09 | 0.01 | 16 | 0.001899 |
| **702** | tr|B4E397|B4E397_HUMAN |  | 1 | 1.21 | 0.042 | 16 | 0.001182 |
| **703** | tr|B4DHN0|B4DHN0_HUMAN |  | 8 | 1.07 | 0.004 | 16 | 0.000905 |
| **704** | tr|B7ZBJ4|B7ZBJ4_HUMAN |  | 1 | 1.11 | 0.026 | 16 | 0.01444 |
| **705** | tr|H0YD72|H0YD72_HUMAN |  | 1 | 1.2 | 0.021 | 16 | 6.93E-05 |
| **706** | sp|Q9Y285|SYFA_HUMAN |  | 4 | 1.02 | 0.022 | 16 | 0.5998 |
| **707** | tr|E5RK75|E5RK75_HUMAN |  | 1 | 1.04 | 0.02 | 16 | 0.3119 |
| **708** | tr|G3V126|G3V126_HUMAN |  | 6 | 1.04 | 0.02 | 16 | 0.2492 |
| **709** | tr|Q9UL90|Q9UL90_HUMAN |  | 2 | 1.04 | 0.016 | 16 | 0.2092 |
| **710** | tr|H0YBR0|H0YBR0_HUMAN |  | 1 | 1.07 | 0.106 | 16 | 0.3882 |
| **711** | sp|Q14847|LASP1_HUMAN |  | 3 | 0.88 | 0.001 | 16 | 6.27E-10 |
| **712** | sp|Q9H1A4|APC1_HUMAN |  | 3 | 0.76 | 0.013 | 16 | 4.98E-07 |
| **713** | tr|F2Z2A1|F2Z2A1_HUMAN |  | 1 | 1.09 | 0.052 | 16 | 0.1429 |
| **714** | sp|O94875|SRBS2_HUMAN |  | 1 | 1.24 | 0.015 | 16 | 8.49E-07 |
| **715** | sp|P07305|H10_HUMAN |  | 1 | 1.12 | 0.056 | 16 | 0.06862 |
| **716** | sp|P01861|IGHG4_HUMAN |  | 3 | 0.95 | 0.017 | 16 | 0.1213 |
| **717** | sp|Q4G0X9|CCD40_HUMAN |  | 1 | 1.21 | 0.037 | 16 | 0.0004823 |
| **718** | tr|B1AH77|B1AH77_HUMAN |  | 2 | 1.21 | 0.038 | 16 | 0.0006837 |
| **719** | tr|B4E1D8|B4E1D8_HUMAN |  | 16 | 1.03 | 0.008 | 16 | 0.2913 |
| **720** | tr|E5RGQ3|E5RGQ3_HUMAN |  | 1 | 0.96 | 0.076 | 16 | 0.6112 |
| **721** | sp|P25787|PSA2_HUMAN |  | 10 | 1.03 | 0.003 | 16 | 0.01758 |
| **722** | tr|B4E0I8|B4E0I8_HUMAN |  | 5 | 1.16 | 0.052 | 16 | 0.01493 |
| **723** | sp|Q9UJV8|PURG_HUMAN |  | 1 | 0.54 | 0.046 | 16 | 3.24E-07 |
| **724** | tr|Q6FHJ5|Q6FHJ5_HUMAN |  | 1 | 1 | 0.019 | 16 | 0.8925 |
| **725** | tr|H0YH25|H0YH25_HUMAN |  | 1 | 0.86 | 0.366 | 16 | 0.3832 |
| **726** | tr|G3V1P3|G3V1P3_HUMAN |  | 1 | 0.83 | 0.014 | 16 | 4.09E-05 |
| **727** | sp|P30153|2AAA_HUMAN |  | 18 | 0.91 | 0.005 | 16 | 8.70E-05 |
| **728** | sp|P29692|EF1D_HUMAN |  | 6 | 1.01 | 0.227 | 16 | 0.9375 |
| **729** | tr|Q0PNF2|Q0PNF2_HUMAN |  | 1 | 0.82 | 0.098 | 16 | 0.03528 |
| **730** | tr|Q5TEC6|Q5TEC6_HUMAN |  | 1 | 2.13 | 0.421 | 16 | 4.72E-06 |
| **731** | tr|A0A0A0MTH9|A0A0A0MTH9_HUMAN |  | 1 | 0.9 | 0.023 | 16 | 0.01957 |
| **732** | tr|A0A087X1J7|A0A087X1J7_HUMAN |  | 5 | 1.21 | 0.009 | 16 | 2.64E-07 |
| **733** | sp|P04220|MUCB_HUMAN |  | 1 | 0.89 | 0.045 | 16 | 0.05449 |
| **734** | sp|P48740|MASP1_HUMAN |  | 4 | 0.95 | 0.002 | 16 | 0.001542 |
| **735** | sp|P37802|TAGL2_HUMAN |  | 4 | 0.92 | 0.025 | 16 | 0.04869 |
| **736** | tr|F5H4Q5|F5H4Q5_HUMAN |  | 3 | 0.99 | 0.005 | 16 | 0.683 |
| **737** | sp|P41226|UBA7_HUMAN |  | 7 | 1.07 | 0.015 | 16 | 0.02595 |
| **738** | sp|P40306|PSB10_HUMAN |  | 3 | 0.94 | 0.015 | 16 | 0.05517 |
| **739** | sp|P49327|FAS_HUMAN |  | 44 | 1.03 | 0.003 | 16 | 0.08843 |
| **740** | tr|Q8TCK8|Q8TCK8_HUMAN |  | 1 | 0.94 | 0.053 | 16 | 0.3165 |
| **741** | tr|A0A087WZW8|A0A087WZW8_HUMAN |  | 1 | 0.95 | 0.019 | 16 | 0.1965 |
| **742** | tr|J3KSG3|J3KSG3_HUMAN |  | 1 | 0.85 | 0.04 | 16 | 0.007188 |
| **743** | sp|Q93009|UBP7_HUMAN |  | 19 | 1.04 | 0.001 | 16 | 0.0005079 |
| **744** | tr|B4DDM0|B4DDM0_HUMAN |  | 2 | 1.13 | 0.03 | 16 | 0.01073 |
| **745** | sp|P01767|HV306_HUMAN |  | 4 | 0.94 | 0.012 | 16 | 0.0297 |
| **746** | tr|Q9BQD2|Q9BQD2_HUMAN |  | 3 | 1.03 | 0.017 | 16 | 0.3345 |
| **747** | tr|Q6PIL8|Q6PIL8_HUMAN |  | 1 | 0.79 | 0.129 | 16 | 0.03704 |
| **748** | tr|Q53H26|Q53H26_HUMAN |  | 1 | 1.03 | 0.071 | 16 | 0.6578 |
| **749** | tr|D6RJH0|D6RJH0_HUMAN |  | 1 | 1.01 | 0.099 | 16 | 0.9458 |
| **750** | sp|Q16512|PKN1_HUMAN |  | 3 | 1.06 | 0.005 | 16 | 0.008455 |
| **751** | sp|P14174|MIF_HUMAN |  | 2 | 1.17 | 0.06 | 16 | 0.01436 |
| **752** | tr|B7Z7P8|B7Z7P8_HUMAN |  | 10 | 0.97 | 0.007 | 16 | 0.203 |
| **753** | tr|C9JXR7|C9JXR7_HUMAN |  | 2 | 0.85 | 0.041 | 16 | 0.009093 |
| **754** | tr|K7EN45|K7EN45_HUMAN |  | 3 | 1.01 | 0.011 | 16 | 0.7714 |
| **755** | tr|Q658J0|Q658J0_HUMAN |  | 3 | 0.96 | 0.028 | 16 | 0.3391 |
| **756** | tr|A8K6Y1|A8K6Y1_HUMAN |  | 5 | 0.89 | 0.024 | 16 | 0.0145 |
| **757** | tr|Q5HY54|Q5HY54_HUMAN |  | 41 | 1.09 | 0.054 | 16 | 0.1257 |
| **758** | tr|E7EUT5|E7EUT5_HUMAN |  | 7 | 0.83 | 0.004 | 16 | 1.18E-08 |
| **759** | tr|B0YJC4|B0YJC4_HUMAN |  | 16 | 2.02 | 0.021 | 16 | 2.36E-14 |
| **760** | tr|Q8TAS2|Q8TAS2_HUMAN |  | 1 | 0.66 | 0.255 | 16 | 0.01655 |
| **761** | tr|B4DNY3|B4DNY3_HUMAN |  | 6 | 1.06 | 0.109 | 16 | 0.5086 |
| **762** | sp|O95747|OXSR1_HUMAN |  | 14 | 0.87 | 0.041 | 16 | 0.02038 |
| **763** | tr|E5RI98|E5RI98_HUMAN |  | 1 | 1.13 | 0.025 | 16 | 0.004603 |
| **764** | tr|B4DNK4|B4DNK4_HUMAN |  | 13 | 1.08 | 0.098 | 16 | 0.3139 |
| **765** | tr|A0A075B6H6|A0A075B6H6_HUMAN |  | 1 | 1 | 0.009 | 16 | 0.8461 |
| **766** | tr|Q5VZR0|Q5VZR0_HUMAN |  | 2 | 0.97 | 0.027 | 16 | 0.5467 |
| **767** | sp|P35527|K1C9_HUMAN |  | 11 | 1.07 | 0.233 | 16 | 0.5746 |
| **768** | tr|C9JGV7|C9JGV7_HUMAN |  | 1 | 4.18 | 13.652 | 16 | 0.003618 |
| **769** | tr|B2R6L0|B2R6L0_HUMAN |  | 1 | 0.9 | 0.033 | 16 | 0.0544 |
| **770** | tr|E9PSG0|E9PSG0_HUMAN |  | 1 | 1.1 | 0.135 | 16 | 0.2823 |
| **771** | sp|Q9BY43|CHM4A_HUMAN |  | 1 | 1 | 0.004 | 16 | 0.8592 |
| **772** | tr|A0A087WVD7|A0A087WVD7_HUMAN |  | 3 | 0.97 | 0.011 | 16 | 0.2092 |
| **773** | tr|A0A087WV30|A0A087WV30_HUMAN |  | 1 | 1.24 | 0.256 | 16 | 0.07576 |
| **774** | sp|P62277|RS13_HUMAN |  | 2 | 1.05 | 0.011 | 16 | 0.0562 |
| **775** | tr|Q59HC1|Q59HC1_HUMAN |  | 2 | 0.97 | 0.016 | 16 | 0.3097 |
| **776** | sp|O43765|SGTA_HUMAN |  | 9 | 1.08 | 0.006 | 16 | 0.001175 |
| **777** | tr|F5H365|F5H365_HUMAN |  | 3 | 1.12 | 0.031 | 16 | 0.01705 |
| **778** | tr|B4DXG0|B4DXG0_HUMAN |  | 3 | 1.22 | 0.039 | 16 | 0.0004771 |
| **779** | sp|Q14697|GANAB_HUMAN |  | 11 | 1.12 | 0.006 | 16 | 1.13E-05 |
| **780** | tr|A0A087WYW7|A0A087WYW7_HUMAN |  | 1 | 1.19 | 0.186 | 16 | 0.1054 |
| **781** | tr|E9PNF5|E9PNF5_HUMAN |  | 2 | 1.02 | 0.021 | 16 | 0.5371 |
| **782** | tr|H0Y3Y3|H0Y3Y3_HUMAN |  | 1 | 0.93 | 0.018 | 16 | 0.05962 |
| **783** | tr|B8ZZU8|B8ZZU8_HUMAN |  | 2 | 1.11 | 0.024 | 16 | 0.0125 |
| **784** | tr|Q05DE9|Q05DE9_HUMAN |  | 2 | 1.16 | 0.013 | 16 | 6.13E-05 |
| **785** | tr|Q5JQ44|Q5JQ44_HUMAN |  | 2 | 1.24 | 0.04 | 16 | 0.0002629 |
| **786** | tr|H0Y2Q8|H0Y2Q8_HUMAN |  | 1 | 1.1 | 0.046 | 16 | 0.07076 |
| **787** | sp|P68366|TBA4A_HUMAN |  | 1 | 0.91 | 0.003 | 16 | 5.15E-06 |
| **788** | sp|P28070|PSB4_HUMAN |  | 6 | 1.03 | 0.004 | 16 | 0.1003 |
| **789** | tr|B4DV28|B4DV28_HUMAN |  | 11 | 1.11 | 0.027 | 16 | 0.01526 |
| **790** | tr|B2R815|B2R815_HUMAN |  | 3 | 0.98 | 0.105 | 16 | 0.8224 |
| **791** | sp|P01023|A2MG_HUMAN |  | 23 | 1.06 | 0.003 | 16 | 0.0005102 |
| **792** | sp|P26038|MOES_HUMAN |  | 15 | 1.08 | 0.003 | 16 | 0.0001195 |
| **793** | sp|P11172|UMPS_HUMAN |  | 5 | 0.92 | 0.023 | 16 | 0.05214 |
| **794** | tr|B2R9V7|B2R9V7_HUMAN |  | 2 | 0.95 | 0.029 | 16 | 0.2933 |
| **795** | tr|L8ECM8|L8ECM8_HUMAN |  | 1 | 1.3 | 0.476 | 16 | 0.1051 |
| **796** | tr|K7ENT6|K7ENT6_HUMAN |  | 1 | 0.93 | 0.013 | 16 | 0.02091 |
| **797** | sp|P20042|IF2B_HUMAN |  | 4 | 0.94 | 0.008 | 16 | 0.02137 |
| **798** | tr|B7Z8U8|B7Z8U8_HUMAN |  | 1 | 1.1 | 0.014 | 16 | 0.004595 |
| **799** | tr|B4DFY5|B4DFY5_HUMAN |  | 3 | 1.04 | 0.062 | 16 | 0.5805 |
| **800** | tr|E7DVW7|E7DVW7_HUMAN |  | 1 | 1.03 | 0.062 | 16 | 0.6936 |
| **801** | sp|P16157|ANK1_HUMAN |  | 14 | 0.85 | 0.02 | 16 | 0.0005726 |
| **802** | tr|A0A087WSY6|A0A087WSY6_HUMAN |  | 1 | 1.03 | 0.159 | 16 | 0.7544 |
| **803** | tr|A0A087WYC8|A0A087WYC8_HUMAN |  | 1 | 1.1 | 0.027 | 16 | 0.02934 |
| **804** | tr|B3KQX1|B3KQX1_HUMAN |  | 1 | 1 | 0.038 | 16 | 0.9243 |
| **805** | sp|Q07954|LRP1_HUMAN |  | 5 | 1.05 | 0.012 | 16 | 0.1133 |
| **806** | sp|Q9BZV1|UBXN6_HUMAN |  | 4 | 1.02 | 0.034 | 16 | 0.732 |
| **807** | sp|P04632|CPNS1_HUMAN |  | 1 | 1.04 | 0.005 | 16 | 0.02652 |
| **808** | tr|H0Y905|H0Y905_HUMAN |  | 1 | 1.06 | 0.036 | 16 | 0.1952 |
| **809** | tr|C9J5C3|C9J5C3_HUMAN |  | 4 | 1.02 | 0.008 | 16 | 0.4885 |
| **810** | sp|O75558|STX11_HUMAN |  | 1 | 0.84 | 0.035 | 16 | 0.002961 |
| **811** | sp|P13224|GP1BB_HUMAN |  | 2 | 0.92 | 0.035 | 16 | 0.112 |
| **812** | tr|A0A0A0MSP7|A0A0A0MSP7_HUMAN |  | 2 | 0.86 | 0.02 | 16 | 0.001195 |
| **813** | sp|Q9P2R3|ANFY1_HUMAN |  | 22 | 1.01 | 0.001 | 16 | 0.571 |
| **814** | sp|P46060|RAGP1_HUMAN |  | 13 | 0.87 | 0.006 | 16 | 1.01E-05 |
| **815** | sp|P30154|2AAB_HUMAN |  | 8 | 0.94 | 0.008 | 16 | 0.01848 |
| **816** | sp|Q92522|H1X_HUMAN |  | 1 | 2.56 | 0.251 | 16 | 2.55E-09 |
| **817** | tr|F2RM37|F2RM37_HUMAN |  | 4 | 0.86 | 0.017 | 16 | 0.0004683 |
| **818** | tr|A8K067|A8K067_HUMAN |  | 1 | 1.01 | 0.366 | 15 | 0.9586 |
| **819** | tr|B4DHR0|B4DHR0_HUMAN |  | 1 | 1.09 | 0.04 | 16 | 0.07861 |
| **820** | sp|P30049|ATPD_HUMAN |  | 1 | 1.05 | 0.031 | 16 | 0.3168 |
| **821** | sp|P28066|PSA5_HUMAN |  | 11 | 1.05 | 0.004 | 16 | 0.00594 |
| **822** | sp|Q9BWP8|COL11_HUMAN |  | 4 | 0.9 | 0.009 | 16 | 0.001295 |
| **823** | tr|A0A068LKQ8|A0A068LKQ8_HUMAN |  | 1 | 0.91 | 0.023 | 16 | 0.03589 |
| **824** | tr|A8K6Z6|A8K6Z6_HUMAN |  | 1 | 1.03 | 0.006 | 16 | 0.1513 |
| **825** | sp|Q04917|1433F_HUMAN |  | 3 | 1.11 | 0.006 | 16 | 7.25E-05 |
| **826** | tr|G3V5X6|G3V5X6_HUMAN |  | 2 | 1.07 | 0.02 | 16 | 0.05404 |
| **827** | tr|A2NB44|A2NB44_HUMAN |  | 1 | 0.92 | 0.146 | 16 | 0.4114 |
| **828** | tr|A0A024RAB6|A0A024RAB6_HUMAN |  | 2 | 1.06 | 0.022 | 16 | 0.1059 |
| **829** | tr|K7EJT0|K7EJT0_HUMAN |  | 1 | 0.93 | 0.067 | 16 | 0.3125 |
| **830** | tr|B3KPF0|B3KPF0_HUMAN |  | 1 | 1.07 | 0.018 | 16 | 0.07016 |
| **831** | tr|A0JLP2|A0JLP2_HUMAN |  | 1 | 1.18 | 0.199 | 16 | 0.1306 |
| **832** | tr|F8WFC3|F8WFC3_HUMAN |  | 1 | 1.29 | 0.036 | 16 | 1.72E-05 |
| **833** | tr|Q53HL1|Q53HL1_HUMAN |  | 3 | 1.04 | 0.013 | 16 | 0.1644 |
| **834** | sp|O15212|PFD6_HUMAN |  | 2 | 1.05 | 0.049 | 16 | 0.4217 |
| **835** | sp|P55036|PSMD4_HUMAN |  | 12 | 0.94 | 0.004 | 16 | 0.002147 |
| **836** | tr|A0A087WU91|A0A087WU91_HUMAN |  | 2 | 1.05 | 0.051 | 16 | 0.4313 |
| **837** | tr|E5RJ76|E5RJ76_HUMAN |  | 1 | 1 | 0.328 | 16 | 0.9945 |
| **838** | tr|Q5STU3|Q5STU3_HUMAN |  | 5 | 1.31 | 0.011 | 16 | 5.49E-09 |
| **839** | sp|O75396|SC22B_HUMAN |  | 4 | 0.82 | 0.036 | 16 | 0.001834 |
| **840** | tr|H0Y6Z4|H0Y6Z4_HUMAN |  | 1 | 1.25 | 0.791 | 16 | 0.2788 |
| **841** | sp|Q9NPE2|NGRN_HUMAN |  | 1 | 1.09 | 0.057 | 16 | 0.142 |
| **842** | tr|Q09LL5|Q09LL5_HUMAN |  | 4 | 1 | 0.026 | 16 | 0.9601 |
| **843** | tr|Q8N5Z7|Q8N5Z7_HUMAN |  | 1 | 0.75 | 0.005 | 16 | 3.11E-10 |
| **844** | tr|H0Y9J1|H0Y9J1_HUMAN |  | 1 | 1.02 | 0.069 | 16 | 0.7755 |
| **845** | tr|A0A024R9Y3|A0A024R9Y3_HUMAN |  | 34 | 0.92 | 0.003 | 16 | 2.73E-05 |
| **846** | tr|Q53F51|Q53F51_HUMAN |  | 4 | 1.09 | 0.01 | 16 | 0.004194 |
| **847** | sp|P53618|COPB_HUMAN |  | 4 | 1.01 | 0.029 | 16 | 0.8891 |
| **848** | tr|Q53FI1|Q53FI1_HUMAN |  | 4 | 1.03 | 0.009 | 16 | 0.2458 |
| **849** | tr|Q5CZ91|Q5CZ91_HUMAN |  | 1 | 1.12 | 0.131 | 16 | 0.216 |
| **850** | sp|Q8TAF3|WDR48_HUMAN |  | 1 | 0.96 | 0.073 | 16 | 0.5193 |
| **851** | tr|E7EX53|E7EX53_HUMAN |  | 1 | 0.94 | 0.031 | 16 | 0.2148 |
| **852** | tr|B4DDF4|B4DDF4_HUMAN |  | 2 | 0.97 | 0.029 | 16 | 0.4763 |
| **853** | tr|H0Y9P1|H0Y9P1_HUMAN |  | 1 | 1.19 | 0.171 | 16 | 0.08656 |
| **854** | sp|P22061|PIMT_HUMAN |  | 14 | 0.95 | 0.071 | 16 | 0.4277 |
| **855** | sp|Q15746|MYLK_HUMAN |  | 1 | 1.26 | 0.069 | 16 | 0.001419 |
| **856** | tr|H0YC05|H0YC05_HUMAN |  | 1 | 1.06 | 0.063 | 16 | 0.3388 |
| **857** | sp|O95248|MTMR5_HUMAN |  | 2 | 1.08 | 0.052 | 16 | 0.1895 |
| **858** | sp|P13667|PDIA4_HUMAN |  | 6 | 1.04 | 0.009 | 16 | 0.08714 |
| **859** | tr|Q76N57|Q76N57_HUMAN |  | 1 | 0.94 | 0.033 | 16 | 0.2176 |
| **860** | tr|B3KV60|B3KV60_HUMAN |  | 2 | 0.96 | 0.007 | 16 | 0.07388 |
| **861** | sp|Q15814|TBCC_HUMAN |  | 1 | 0.66 | 0.027 | 16 | 5.72E-07 |
| **862** | sp|P04114|APOB_HUMAN |  | 1 | 0.87 | 0.017 | 16 | 0.001666 |
| **863** | tr|B4DY46|B4DY46_HUMAN |  | 7 | 1.01 | 0.012 | 16 | 0.6335 |
| **864** | tr|E5RJP9|E5RJP9_HUMAN |  | 1 | 0.55 | 0.059 | 16 | 2.08E-06 |
| **865** | sp|P50552|VASP_HUMAN |  | 2 | 0.98 | 0.054 | 16 | 0.736 |
| **866** | sp|P30048|PRDX3_HUMAN |  | 1 | 1.14 | 0.109 | 16 | 0.1013 |
| **867** | tr|B7Z3K0|B7Z3K0_HUMAN |  | 1 | 1.03 | 0.016 | 16 | 0.3206 |
| **868** | tr|C9JSU7|C9JSU7_HUMAN |  | 1 | 1.05 | 0.036 | 16 | 0.3351 |
| **869** | tr|Q9BR63|Q9BR63_HUMAN |  | 2 | 0.96 | 0.033 | 16 | 0.4141 |
| **870** | sp|P00390|GSHR_HUMAN |  | 1 | 0.96 | 0.034 | 16 | 0.411 |
| **871** | tr|A0A075B6H7|A0A075B6H7_HUMAN |  | 1 | 0.94 | 0.079 | 16 | 0.4023 |
| **872** | tr|K7ELP0|K7ELP0_HUMAN |  | 1 | 1.09 | 0.03 | 16 | 0.05401 |
| **873** | sp|P23919|KTHY_HUMAN |  | 5 | 0.91 | 0.078 | 16 | 0.2375 |
| **874** | tr|A0A087WSY5|A0A087WSY5_HUMAN |  | 2 | 0.77 | 0.032 | 16 | 0.0001167 |
| **875** | sp|P80217|IN35_HUMAN |  | 3 | 1.12 | 0.095 | 16 | 0.1336 |
| **876** | tr|H0YMU3|H0YMU3_HUMAN |  | 1 | 1.63 | 2.147 | 16 | 0.1042 |
| **877** | sp|P13798|ACPH_HUMAN |  | 25 | 0.9 | 0.001 | 16 | 1.64E-09 |
| **878** | sp|Q9H3K6|BOLA2_HUMAN |  | 2 | 0.94 | 0.04 | 16 | 0.216 |
| **879** | tr|H0Y539|H0Y539_HUMAN |  | 4 | 0.93 | 0.01 | 16 | 0.01863 |
| **880** | tr|C9JBI3|C9JBI3_HUMAN |  | 1 | 1.08 | 0.081 | 16 | 0.2534 |
| **881** | tr|B4DZ42|B4DZ42_HUMAN |  | 1 | 0.97 | 0.079 | 16 | 0.6802 |
| **882** | sp|Q9ULP9|TBC24_HUMAN |  | 6 | 0.86 | 0.031 | 16 | 0.007279 |
| **883** | sp|Q86VN1|VPS36_HUMAN |  | 2 | 1.02 | 0.097 | 16 | 0.766 |
| **884** | tr|A8K8U1|A8K8U1_HUMAN |  | 30 | 1.01 | 0.013 | 16 | 0.7474 |
| **885** | tr|Q9BUD9|Q9BUD9_HUMAN |  | 1 | 0.93 | 0.015 | 16 | 0.03799 |
| **886** | sp|Q15173|2A5B_HUMAN |  | 2 | 0.89 | 0.007 | 16 | 0.000147 |
| **887** | tr|Q0ZCH4|Q0ZCH4_HUMAN |  | 1 | 1.26 | 0.062 | 16 | 0.0008512 |
| **888** | sp|P23142|FBLN1_HUMAN |  | 4 | 0.91 | 0.01 | 16 | 0.002621 |
| **889** | tr|B3KW95|B3KW95_HUMAN |  | 1 | 1.19 | 0.094 | 16 | 0.02765 |
| **890** | sp|Q53TN4|CYBR1_HUMAN |  | 1 | 0.85 | 0.03 | 16 | 0.003156 |
| **891** | sp|Q15813|TBCE_HUMAN |  | 1 | 0.83 | 0.041 | 16 | 0.003807 |
| **892** | sp|Q9BW85|CCD94_HUMAN |  | 1 | 1.1 | 0.143 | 16 | 0.2861 |
| **893** | tr|B7ZAN4|B7ZAN4_HUMAN |  | 1 | 1.07 | 0.079 | 16 | 0.366 |
| **894** | sp|P13639|EF2_HUMAN |  | 4 | 0.99 | 0.007 | 16 | 0.5801 |
| **895** | tr|Q53T47|Q53T47_HUMAN |  | 1 | 0.79 | 0.035 | 16 | 0.0004975 |
| **896** | sp|Q96BS2|CHP3_HUMAN |  | 2 | 0.98 | 0.005 | 16 | 0.2327 |
| **897** | tr|Q14484|Q14484_HUMAN |  | 1 | 0.82 | 0.111 | 16 | 0.04372 |
| **898** | tr|J3KP49|J3KP49_HUMAN |  | 1 | 0.99 | 0.025 | 16 | 0.7429 |
| **899** | tr|X5DP03|X5DP03_HUMAN |  | 1 | 0.87 | 0.029 | 16 | 0.008995 |
| **900** | tr|Q6ZN40|Q6ZN40_HUMAN |  | 1 | 1.2 | 0.063 | 16 | 0.005984 |
| **901** | tr|B3KSR5|B3KSR5_HUMAN |  | 1 | 0.95 | 0.036 | 16 | 0.2726 |
| **902** | sp|Q9NRY5|F1142_HUMAN |  | 2 | 0.92 | 0.015 | 16 | 0.01426 |
| **903** | tr|B2R6H7|B2R6H7_HUMAN |  | 10 | 0.93 | 0.009 | 16 | 0.008271 |
| **904** | tr|H7BZJ3|H7BZJ3_HUMAN |  | 1 | 1.29 | 0.073 | 16 | 0.0006466 |
| **905** | tr|B2RCG9|B2RCG9_HUMAN |  | 4 | 1.01 | 0.007 | 16 | 0.7185 |
| **906** | tr|Q71V99|Q71V99_HUMAN |  | 5 | 0.91 | 0.004 | 16 | 0.0001081 |
| **907** | sp|P26447|S10A4_HUMAN |  | 3 | 1.11 | 0.033 | 16 | 0.02971 |
| **908** | sp|Q13619|CUL4A_HUMAN |  | 1 | 0.94 | 0.011 | 16 | 0.03943 |
| **909** | tr|B3KYB6|B3KYB6_HUMAN |  | 1 | 1.03 | 0.04 | 16 | 0.5518 |
| **910** | sp|P27348|1433T_HUMAN |  | 10 | 1.11 | 0.004 | 16 | 7.56E-06 |
| **911** | sp|P22891|PROZ_HUMAN |  | 1 | 1.18 | 0.178 | 16 | 0.1068 |
| **912** | tr|A0A087WWP8|A0A087WWP8_HUMAN |  | 1 | 1.04 | 0.047 | 16 | 0.4276 |
| **913** | sp|P80108|PHLD_HUMAN |  | 12 | 0.91 | 0.002 | 16 | 1.68E-06 |
| **914** | tr|A0A0A0MSJ2|A0A0A0MSJ2_HUMAN |  | 2 | 1.01 | 0.041 | 16 | 0.8642 |
| **915** | tr|C9JMT8|C9JMT8_HUMAN |  | 1 | 0.86 | 0.015 | 16 | 0.0004669 |
| **916** | tr|Q6ICM2|Q6ICM2_HUMAN |  | 2 | 0.88 | 0.018 | 16 | 0.00209 |
| **917** | sp|P48047|ATPO_HUMAN |  | 1 | 1.07 | 0.103 | 16 | 0.3722 |
| **918** | tr|A0A024R5J9|A0A024R5J9_HUMAN |  | 1 | 0.82 | 0.107 | 16 | 0.04082 |
| **919** | tr|Q7KZX8|Q7KZX8_HUMAN |  | 8 | 1.07 | 0.005 | 16 | 0.001145 |
| **920** | tr|C9J9K3|C9J9K3_HUMAN |  | 8 | 1.06 | 0.007 | 16 | 0.0152 |
| **921** | sp|P20160|CAP7_HUMAN |  | 3 | 1.83 | 0.088 | 16 | 1.11E-08 |
| **922** | sp|O94919|ENDD1_HUMAN |  | 1 | 0.67 | 0.022 | 16 | 2.33E-07 |
| **923** | tr|Q9UFG1|Q9UFG1_HUMAN |  | 1 | 0.96 | 0.128 | 16 | 0.6249 |
| **924** | tr|B4DZY7|B4DZY7_HUMAN |  | 3 | 0.98 | 0.04 | 16 | 0.7359 |
| **925** | tr|O15256|O15256_HUMAN |  | 2 | 0.97 | 0.01 | 16 | 0.2547 |
| **926** | tr|B4E3A8|B4E3A8_HUMAN |  | 3 | 1.19 | 0.015 | 16 | 2.06E-05 |
| **927** | sp|Q99598|TSNAX_HUMAN |  | 3 | 1.21 | 0.015 | 16 | 5.17E-06 |
| **928** | sp|Q15008|PSMD6_HUMAN |  | 15 | 0.96 | 0.006 | 16 | 0.04641 |
| **929** | sp|P02730|B3AT_HUMAN |  | 18 | 0.62 | 0.022 | 16 | 4.37E-08 |
| **930** | tr|B4DXF1|B4DXF1_HUMAN |  | 1 | 1.44 | 0.1 | 16 | 4.86E-05 |
| **931** | tr|A0A087WXQ5|A0A087WXQ5_HUMAN |  | 1 | 0.96 | 0.015 | 16 | 0.1827 |
| **932** | tr|F8VXB1|F8VXB1_HUMAN |  | 2 | 1.14 | 0.021 | 16 | 0.001613 |
| **933** | tr|Q2NLD4|Q2NLD4_HUMAN |  | 6 | 0.95 | 0.008 | 16 | 0.04715 |
| **934** | sp|Q68CJ6|SLIP_HUMAN |  | 1 | 0.88 | 0.053 | 16 | 0.05445 |
| **935** | sp|O43776|SYNC_HUMAN |  | 1 | 0.91 | 0.021 | 16 | 0.02005 |
| **936** | tr|Q8N6Z2|Q8N6Z2_HUMAN |  | 1 | 1.11 | 0.02 | 16 | 0.00871 |
| **937** | sp|Q9H4B7|TBB1_HUMAN |  | 13 | 0.91 | 0.004 | 16 | 4.62E-05 |
| **938** | tr|A0A024R529|A0A024R529_HUMAN |  | 6 | 0.84 | 0.026 | 16 | 0.00141 |
| **939** | sp|P55072|TERA_HUMAN |  | 41 | 0.89 | 0.002 | 16 | 1.17E-08 |
| **940** | tr|H3BT53|H3BT53_HUMAN |  | 1 | 1.19 | 0.172 | 16 | 0.08652 |
| **941** | tr|B4DRY5|B4DRY5_HUMAN |  | 1 | 0.87 | 0.039 | 16 | 0.0226 |
| **942** | tr|Q05CW7|Q05CW7_HUMAN |  | 1 | 1.09 | 0.105 | 15 | 0.305 |
| **943** | tr|K9JIK7|K9JIK7_HUMAN |  | 1 | 0.4 | 0.038 | 16 | 2.78E-09 |
| **944** | tr|B2RA39|B2RA39_HUMAN |  | 4 | 0.92 | 0.005 | 16 | 0.0005195 |
| **945** | tr|B4E107|B4E107_HUMAN |  | 3 | 1.01 | 0.033 | 16 | 0.797 |
| **946** | tr|A8K4W5|A8K4W5_HUMAN |  | 1 | 1 | 0.008 | 16 | 0.8565 |
| **947** | sp|Q9BX59|TPSNR_HUMAN |  | 1 | 1.01 | 0.098 | 16 | 0.8709 |
| **948** | tr|Q5T7U0|Q5T7U0_HUMAN |  | 1 | 0.83 | 0.009 | 16 | 3.63E-06 |
| **949** | sp|P00748|FA12_HUMAN |  | 6 | 0.87 | 0.012 | 16 | 0.0002105 |
| **950** | tr|C9JYK5|C9JYK5_HUMAN |  | 1 | 1.02 | 0.028 | 16 | 0.6572 |
| **951** | sp|Q9BV20|MTNA_HUMAN |  | 3 | 0.95 | 0.069 | 16 | 0.4927 |
| **952** | tr|Q3Y9I8|Q3Y9I8_HUMAN |  | 1 | 0.78 | 0.042 | 16 | 0.0007223 |
| **953** | sp|O00299|CLIC1_HUMAN |  | 3 | 1.33 | 0.023 | 16 | 2.98E-07 |
| **954** | tr|X5D7T7|X5D7T7_HUMAN |  | 2 | 0.95 | 0.017 | 16 | 0.1101 |
| **955** | sp|P06727|APOA4_HUMAN |  | 24 | 1.29 | 0.02 | 16 | 8.18E-07 |
| **956** | sp|Q9HAU6|TCTP8_HUMAN |  | 1 | 1.15 | 0.141 | 16 | 0.1233 |
| **957** | sp|P24844|MYL9_HUMAN |  | 3 | 1.13 | 0.006 | 16 | 7.58E-06 |
| **958** | tr|A0A087WZB5|A0A087WZB5_HUMAN |  | 3 | 1.08 | 0.014 | 16 | 0.0177 |
| **959** | tr|B4E3H3|B4E3H3_HUMAN |  | 1 | 0.94 | 0.059 | 16 | 0.3554 |
| **960** | sp|Q15404|RSU1_HUMAN |  | 6 | 1.03 | 0.004 | 16 | 0.07567 |
| **961** | tr|E9PJD9|E9PJD9_HUMAN |  | 2 | 1.05 | 0.034 | 16 | 0.3349 |
| **962** | tr|G3V2F7|G3V2F7_HUMAN |  | 2 | 1.13 | 0.125 | 16 | 0.1694 |
| **963** | sp|P27824|CALX_HUMAN |  | 2 | 1.12 | 0.035 | 16 | 0.0271 |
| **964** | sp|Q86UX7|URP2_HUMAN |  | 24 | 0.97 | 0.015 | 16 | 0.3311 |
| **965** | sp|P02671|FIBA_HUMAN |  | 22 | 1.08 | 0.024 | 16 | 0.05518 |
| **966** | sp|O00231|PSD11_HUMAN |  | 18 | 1.01 | 0.004 | 16 | 0.6345 |
| **967** | tr|D6RHH4|D6RHH4_HUMAN |  | 3 | 0.95 | 0.022 | 16 | 0.2438 |
| **968** | tr|B3KVX5|B3KVX5_HUMAN |  | 2 | 1.06 | 0.017 | 16 | 0.1002 |
| **969** | sp|Q9NTJ3|SMC4_HUMAN |  | 6 | 1.03 | 0.015 | 16 | 0.3633 |
| **970** | tr|Q14485|Q14485_HUMAN |  | 2 | 0.94 | 0.034 | 16 | 0.2115 |
| **971** | tr|A0A024QZR0|A0A024QZR0_HUMAN |  | 3 | 0.83 | 0.054 | 16 | 0.01002 |
| **972** | tr|H0UID2|H0UID2_HUMAN |  | 1 | 0.87 | 0.071 | 16 | 0.06365 |
| **973** | sp|P14543|NID1_HUMAN |  | 1 | 0.88 | 0.078 | 16 | 0.1036 |
| **974** | sp|Q96PD5|PGRP2_HUMAN |  | 6 | 0.68 | 0.078 | 16 | 0.0003171 |
| **975** | sp|P18054|LOX12_HUMAN |  | 3 | 0.95 | 0.036 | 16 | 0.314 |
| **976** | tr|F8UU18|F8UU18_HUMAN |  | 1 | 0.94 | 0.015 | 16 | 0.06497 |
| **977** | tr|B3KRI9|B3KRI9_HUMAN |  | 2 | 0.81 | 0.003 | 16 | 8.88E-10 |
| **978** | tr|G3V4P7|G3V4P7_HUMAN |  | 2 | 0.99 | 0.026 | 16 | 0.8972 |
| **979** | sp|P25398|RS12_HUMAN |  | 2 | 1.1 | 0.053 | 16 | 0.1046 |
| **980** | tr|Q5HYM1|Q5HYM1_HUMAN |  | 1 | 1.12 | 0.121 | 16 | 0.1919 |
| **981** | tr|B5BUK7|B5BUK7_HUMAN |  | 1 | 1.1 | 0.091 | 16 | 0.1976 |
| **982** | tr|H7C599|H7C599_HUMAN |  | 1 | 1.24 | 0.47 | 16 | 0.1855 |
| **983** | sp|Q13201|MMRN1_HUMAN |  | 1 | 1.03 | 0.018 | 16 | 0.4413 |
| **984** | tr|A0A096LNV3|A0A096LNV3_HUMAN |  | 3 | 0.9 | 0.014 | 16 | 0.003553 |
| **985** | sp|Q9UJU6|DBNL_HUMAN |  | 2 | 0.98 | 0.001 | 16 | 0.09046 |
| **986** | tr|B3KXA9|B3KXA9_HUMAN |  | 1 | 1.05 | 0.032 | 16 | 0.3125 |
| **987** | sp|P15374|UCHL3_HUMAN |  | 1 | 1.19 | 0.026 | 16 | 0.0003158 |
| **988** | sp|Q96IC2|REXON_HUMAN |  | 2 | 0.75 | 0.017 | 16 | 1.36E-06 |
| **989** | tr|H0Y7F1|H0Y7F1_HUMAN |  | 1 | 1.08 | 0.115 | 16 | 0.3757 |
| **990** | tr|Q59EZ3|Q59EZ3_HUMAN |  | 1 | 0.85 | 0.024 | 16 | 0.001677 |
| **991** | tr|H0YAK2|H0YAK2_HUMAN |  | 1 | 1.02 | 0.144 | 16 | 0.8294 |
| **992** | sp|P11586|C1TC_HUMAN |  | 1 | 0.78 | 0.015 | 16 | 3.09E-06 |
| **993** | sp|Q15185|TEBP_HUMAN |  | 3 | 0.82 | 0.016 | 16 | 4.34E-05 |
| **994** | tr|B3KUV8|B3KUV8_HUMAN |  | 1 | 0.87 | 0.038 | 16 | 0.01579 |
| **995** | sp|Q15149|PLEC_HUMAN |  | 3 | 1.04 | 0.023 | 16 | 0.3492 |
| **996** | tr|H0YIA2|H0YIA2_HUMAN |  | 1 | 0.8 | 0.012 | 16 | 3.08E-06 |
| **997** | sp|Q9NYB0|TE2IP_HUMAN |  | 1 | 1.79 | 1.512 | 16 | 0.02098 |
| **998** | tr|F5H3C1|F5H3C1_HUMAN |  | 1 | 1 | 0.009 | 16 | 0.9081 |
| **999** | tr|B7ZM73|B7ZM73_HUMAN |  | 2 | 1.13 | 0.067 | 16 | 0.0553 |
| **1000** | tr|H7C3T2|H7C3T2_HUMAN |  | 9 | 0.9 | 0.007 | 16 | 0.0002498 |
| **1001** | sp|P62820|RAB1A_HUMAN |  | 2 | 1.07 | 0.023 | 16 | 0.107 |
| **1002** | tr|H3BVD1|H3BVD1_HUMAN |  | 2 | 0.88 | 0.012 | 16 | 0.000619 |
| **1003** | sp|Q9BRX5|PSF3_HUMAN |  | 2 | 0.99 | 0.01 | 16 | 0.7259 |
| **1004** | sp|P36551|HEM6_HUMAN |  | 1 | 1.01 | 0.079 | 16 | 0.8365 |
| **1005** | tr|J3KNI6|J3KNI6_HUMAN |  | 1 | 0.88 | 0.02 | 16 | 0.005687 |
| **1006** | tr|B2R860|B2R860_HUMAN |  | 3 | 1.15 | 0.032 | 16 | 0.004654 |
| **1007** | tr|Q496A9|Q496A9_HUMAN |  | 1 | 1.22 | 0.402 | 16 | 0.1824 |
| **1008** | tr|Q8TCZ8|Q8TCZ8_HUMAN |  | 1 | 1.34 | 6.056 | 16 | 0.5904 |
| **1009** | sp|Q6PD74|AAGAB_HUMAN |  | 1 | 0.98 | 0.031 | 16 | 0.7003 |
| **1010** | sp|P24666|PPAC_HUMAN |  | 6 | 0.9 | 0.004 | 16 | 5.37E-06 |
| **1011** | tr|B4DWN1|B4DWN1_HUMAN |  | 1 | 0.67 | 0.073 | 16 | 0.000222 |
| **1012** | tr|B7Z7M2|B7Z7M2_HUMAN |  | 1 | 1.18 | 0.081 | 16 | 0.02491 |
| **1013** | sp|Q9H9G7|AGO3_HUMAN |  | 1 | 1.01 | 0.088 | 16 | 0.846 |
| **1014** | sp|P49588|SYAC_HUMAN |  | 3 | 0.98 | 0.014 | 16 | 0.5088 |
| **1015** | tr|A8MXL6|A8MXL6_HUMAN |  | 1 | 0.99 | 0.042 | 16 | 0.8197 |
| **1016** | tr|Q2VEU1|Q2VEU1_HUMAN |  | 3 | 1.01 | 0.01 | 16 | 0.6012 |
| **1017** | tr|B4DPN0|B4DPN0_HUMAN |  | 2 | 1.21 | 0.043 | 16 | 0.0008805 |
| **1018** | sp|Q8WZA9|IRGQ_HUMAN |  | 9 | 0.87 | 0.027 | 16 | 0.005396 |
| **1019** | sp|Q8IY33|MILK2_HUMAN |  | 3 | 1.05 | 0.015 | 16 | 0.1454 |
| **1020** | tr|Q8NAK8|Q8NAK8_HUMAN |  | 1 | 1.08 | 0.128 | 16 | 0.3834 |
| **1021** | tr|Q8TEJ7|Q8TEJ7_HUMAN |  | 1 | 0.83 | 0.016 | 16 | 6.80E-05 |
| **1022** | sp|O15173|PGRC2_HUMAN |  | 2 | 1.07 | 0.033 | 16 | 0.1475 |
| **1023** | sp|P22392|NDKB_HUMAN |  | 5 | 1.16 | 0.021 | 16 | 0.0004969 |
| **1024** | sp|Q08AM6|VAC14_HUMAN |  | 4 | 0.93 | 0.009 | 16 | 0.006699 |
| **1025** | tr|Q86U25|Q86U25_HUMAN |  | 1 | 0.79 | 0.044 | 16 | 0.001045 |
| **1026** | tr|F5GYN4|F5GYN4_HUMAN |  | 5 | 1.12 | 0.028 | 16 | 0.01416 |
| **1027** | tr|B3KSC2|B3KSC2_HUMAN |  | 1 | 1.18 | 0.021 | 16 | 0.0001907 |
| **1028** | sp|Q9Y4P8|WIPI2_HUMAN |  | 4 | 1.09 | 0.011 | 16 | 0.002634 |
| **1029** | tr|B3KN28|B3KN28_HUMAN |  | 2 | 1.07 | 0.103 | 16 | 0.4171 |
| **1030** | sp|Q9H9A6|LRC40_HUMAN |  | 7 | 0.95 | 0.032 | 16 | 0.2517 |
| **1031** | tr|A0A087WUA1|A0A087WUA1_HUMAN |  | 1 | 1.01 | 0.011 | 16 | 0.7529 |
| **1032** | tr|B7ZA62|B7ZA62_HUMAN |  | 3 | 0.9 | 0.018 | 16 | 0.008261 |
| **1033** | tr|M0R2L9|M0R2L9_HUMAN |  | 1 | 1.21 | 0.136 | 16 | 0.0401 |
| **1034** | tr|D6RF86|D6RF86_HUMAN |  | 3 | 0.89 | 0.012 | 16 | 0.001417 |
| **1035** | tr|K7EJ32|K7EJ32_HUMAN |  | 1 | 1.05 | 0.066 | 16 | 0.4904 |
| **1036** | sp|P62195|PRS8_HUMAN |  | 9 | 0.99 | 0.003 | 16 | 0.4418 |
| **1037** | sp|P51149|RAB7A_HUMAN |  | 1 | 0.99 | 0.012 | 16 | 0.7741 |
| **1038** | tr|Q32Q39|Q32Q39_HUMAN |  | 1 | 1.16 | 0.046 | 16 | 0.008403 |
| **1039** | sp|Q9HBV1|POPD3_HUMAN |  | 1 | 1.09 | 0.021 | 16 | 0.02698 |
| **1040** | sp|P05164|PERM_HUMAN |  | 23 | 1.74 | 0.042 | 16 | 3.18E-10 |
| **1041** | sp|Q86YV6|MYLK4_HUMAN |  | 1 | 1.23 | 0.014 | 16 | 1.47E-06 |
| **1042** | tr|L8E853|L8E853_HUMAN |  | 1 | 0.76 | 0.008 | 16 | 1.92E-08 |
| **1043** | tr|J3QL14|J3QL14_HUMAN |  | 1 | 0.94 | 0.063 | 16 | 0.3385 |
| **1044** | sp|O00232|PSD12_HUMAN |  | 18 | 1 | 0.003 | 16 | 0.9396 |
| **1045** | tr|A0A024R035|A0A024R035_HUMAN |  | 9 | 0.93 | 0.047 | 16 | 0.2055 |
| **1046** | tr|F5H2F4|F5H2F4_HUMAN |  | 1 | 0.87 | 0.077 | 16 | 0.08477 |
| **1047** | tr|A0A024RCR6|A0A024RCR6_HUMAN |  | 12 | 0.91 | 0.011 | 16 | 0.003478 |
| **1048** | tr|B2R5H5|B2R5H5_HUMAN |  | 2 | 1.08 | 0.006 | 16 | 0.0004996 |
| **1049** | sp|Q08380|LG3BP_HUMAN |  | 14 | 0.91 | 0.02 | 16 | 0.02217 |
| **1050** | tr|E9PK89|E9PK89_HUMAN |  | 1 | 1.5 | 0.626 | 16 | 0.02262 |
| **1051** | tr|B2R8Z4|B2R8Z4_HUMAN |  | 1 | 0.86 | 0.054 | 16 | 0.02767 |
| **1052** | tr|Q53GB6|Q53GB6_HUMAN |  | 2 | 1.04 | 0.03 | 16 | 0.3578 |
| **1053** | sp|Q9UKK9|NUDT5_HUMAN |  | 5 | 1.5 | 0.031 | 16 | 1.04E-08 |
| **1054** | tr|A0A024R500|A0A024R500_HUMAN |  | 1 | 1.01 | 0.11 | 16 | 0.9215 |
| **1055** | sp|P51665|PSMD7_HUMAN |  | 12 | 1.07 | 0.011 | 16 | 0.0146 |
| **1056** | tr|A2J1N6|A2J1N6_HUMAN |  | 1 | 1.23 | 0.167 | 16 | 0.03918 |
| **1057** | sp|Q8TCT1|PHOP1_HUMAN |  | 1 | 1.06 | 0.105 | 16 | 0.5063 |
| **1058** | sp|O75131|CPNE3_HUMAN |  | 4 | 1.13 | 0.011 | 16 | 0.000183 |
| **1059** | tr|Q6IQ43|Q6IQ43_HUMAN |  | 1 | 0.88 | 0.024 | 16 | 0.009206 |
| **1060** | tr|A0N8J1|A0N8J1_HUMAN |  | 1 | 0.98 | 0.114 | 16 | 0.8555 |
| **1061** | sp|Q8N3F0|MTURN_HUMAN |  | 1 | 0.85 | 0.003 | 16 | 6.19E-09 |
| **1062** | tr|Q7Z4Q5|Q7Z4Q5_HUMAN |  | 1 | 1.3 | 0.039 | 16 | 2.24E-05 |
| **1063** | tr|Q9UES0|Q9UES0_HUMAN |  | 4 | 1.02 | 0.022 | 16 | 0.5354 |
| **1064** | tr|E9PJ21|E9PJ21_HUMAN |  | 1 | 0.89 | 0.007 | 16 | 5.25E-05 |
| **1065** | sp|Q7Z4H3|HDDC2_HUMAN |  | 2 | 0.86 | 0.03 | 16 | 0.005253 |
| **1066** | tr|H0YJ10|H0YJ10_HUMAN |  | 1 | 1 | 0.066 | 16 | 0.9733 |
| **1067** | tr|B4DTV0|B4DTV0_HUMAN |  | 1 | 0.9 | 0.026 | 16 | 0.02798 |
| **1068** | tr|I3L0H8|I3L0H8_HUMAN |  | 2 | 0.99 | 0.023 | 16 | 0.8172 |
| **1069** | tr|E9PL57|E9PL57_HUMAN |  | 2 | 1.05 | 0.011 | 16 | 0.06341 |
| **1070** | tr|H0Y723|H0Y723_HUMAN |  | 1 | 0.94 | 0.053 | 16 | 0.3004 |
| **1071** | tr|C9JZW3|C9JZW3_HUMAN |  | 1 | 1.1 | 0.07 | 16 | 0.1389 |
| **1072** | tr|B4DQG2|B4DQG2_HUMAN |  | 1 | 0.82 | 0.011 | 16 | 5.52E-06 |
| **1073** | tr|J3KNF4|J3KNF4_HUMAN |  | 7 | 1.11 | 0.003 | 16 | 2.86E-07 |
| **1074** | sp|A6NIZ1|RP1BL_HUMAN |  | 4 | 1.04 | 0.004 | 16 | 0.0208 |
| **1075** | sp|P02747|C1QC_HUMAN |  | 5 | 0.93 | 0.002 | 16 | 3.14E-06 |
| **1076** | tr|H3BS66|H3BS66_HUMAN |  | 1 | 0.94 | 0.012 | 16 | 0.0407 |
| **1077** | tr|A0A087WYC5|A0A087WYC5_HUMAN |  | 1 | 0.96 | 0.038 | 16 | 0.4588 |
| **1078** | tr|E5RG81|E5RG81_HUMAN |  | 2 | 1.16 | 0.204 | 16 | 0.1699 |
| **1079** | sp|Q14166|TTL12_HUMAN |  | 16 | 1.11 | 0.006 | 16 | 7.11E-05 |
| **1080** | sp|Q9BXJ9|NAA15_HUMAN |  | 2 | 0.87 | 0.011 | 16 | 0.000273 |
| **1081** | tr|B4E1S3|B4E1S3_HUMAN |  | 1 | 1.14 | 0.148 | 16 | 0.1634 |
| **1082** | tr|H7C1X3|H7C1X3_HUMAN |  | 1 | 0.93 | 0.025 | 16 | 0.09429 |
| **1083** | tr|H0Y9Q1|H0Y9Q1_HUMAN |  | 2 | 0.96 | 0.037 | 16 | 0.4518 |
| **1084** | sp|Q5T619|ZN648_HUMAN |  | 1 | 0.92 | 0.089 | 16 | 0.273 |
| **1085** | sp|Q7Z7N9|T179B_HUMAN |  | 1 | 0.92 | 0.09 | 16 | 0.3162 |
| **1086** | tr|F2Z2V0|F2Z2V0_HUMAN |  | 3 | 1.12 | 0.013 | 16 | 0.0005918 |
| **1087** | tr|D6RAC8|D6RAC8_HUMAN |  | 1 | 1.43 | 0.117 | 16 | 0.0001371 |
| **1088** | tr|C8C504|C8C504_HUMAN |  | 1 | 0.85 | 0.015 | 16 | 0.0002779 |
| **1089** | sp|Q96T76|MMS19_HUMAN |  | 5 | 0.92 | 0.014 | 16 | 0.02376 |
| **1090** | sp|P30050|RL12_HUMAN |  | 2 | 0.99 | 0.029 | 16 | 0.7814 |
| **1091** | tr|B3KNN7|B3KNN7_HUMAN |  | 23 | 0.94 | 0.003 | 16 | 0.001186 |
| **1092** | sp|Q96KK5|H2A1H_HUMAN |  | 3 | 2.74 | 0.168 | 16 | 3.27E-11 |
| **1093** | tr|Q5H964|Q5H964_HUMAN |  | 1 | 0.86 | 0.028 | 16 | 0.005561 |
| **1094** | tr|G3V1D1|G3V1D1_HUMAN |  | 3 | 1.07 | 0.024 | 16 | 0.09228 |
| **1095** | sp|Q96B54|ZN428_HUMAN |  | 3 | 0.95 | 0.036 | 16 | 0.3503 |
| **1096** | tr|Q5VU59|Q5VU59_HUMAN |  | 8 | 1.01 | 0.008 | 16 | 0.7908 |
| **1097** | tr|B4DKV1|B4DKV1_HUMAN |  | 1 | 1.01 | 0.098 | 16 | 0.8559 |
| **1098** | tr|E9PPM9|E9PPM9_HUMAN |  | 1 | 1.16 | 0.083 | 16 | 0.04321 |
| **1099** | tr|J3KRP8|J3KRP8_HUMAN |  | 1 | 0.91 | 0.009 | 16 | 0.001644 |
| **1100** | tr|Q53FR6|Q53FR6_HUMAN |  | 1 | 1.63 | 0.949 | 16 | 0.02135 |
| **1101** | sp|Q96K76|UBP47_HUMAN |  | 16 | 0.94 | 0.001 | 16 | 4.20E-06 |
| **1102** | tr|G3V1J0|G3V1J0_HUMAN |  | 1 | 0.97 | 0.033 | 16 | 0.5664 |
| **1103** | tr|J3KPF0|J3KPF0_HUMAN |  | 1 | 1.02 | 0.085 | 16 | 0.777 |
| **1104** | sp|Q9BRG1|VPS25_HUMAN |  | 1 | 1.19 | 0.159 | 16 | 0.07955 |
| **1105** | tr|D6RAJ6|D6RAJ6_HUMAN |  | 4 | 0.99 | 0.01 | 16 | 0.7785 |
| **1106** | sp|P13807|GYS1_HUMAN |  | 4 | 0.98 | 0.027 | 16 | 0.5877 |
| **1107** | tr|H0YFL7|H0YFL7_HUMAN |  | 1 | 1.03 | 0.023 | 16 | 0.428 |
| **1108** | tr|Q9NXK9|Q9NXK9_HUMAN |  | 1 | 0.68 | 0.029 | 16 | 2.23E-06 |
| **1109** | tr|Q9NWJ6|Q9NWJ6_HUMAN |  | 1 | 1.12 | 0.024 | 16 | 0.007179 |
| **1110** | sp|P04424|ARLY_HUMAN |  | 8 | 1.01 | 0.06 | 16 | 0.853 |
| **1111** | tr|B2R960|B2R960_HUMAN |  | 10 | 1.23 | 0.019 | 16 | 5.76E-06 |
| **1112** | tr|B2MUD5|B2MUD5_HUMAN |  | 3 | 2.05 | 0.118 | 16 | 3.34E-09 |
| **1113** | tr|E7EQG2|E7EQG2_HUMAN |  | 4 | 1.06 | 0.004 | 16 | 0.001545 |
| **1114** | tr|E9PL74|E9PL74_HUMAN |  | 1 | 1.14 | 0.03 | 16 | 0.004892 |
| **1115** | sp|Q16401|PSMD5_HUMAN |  | 21 | 0.98 | 0.001 | 16 | 0.03069 |
| **1116** | tr|Q45KI0|Q45KI0_HUMAN |  | 1 | 0.97 | 0.012 | 16 | 0.2444 |
| **1117** | sp|P01703|LV105_HUMAN |  | 1 | 1.26 | 0.103 | 16 | 0.005752 |
| **1118** | sp|P61970|NTF2_HUMAN |  | 5 | 1.26 | 0.035 | 16 | 5.83E-05 |
| **1119** | sp|Q86X55|CARM1_HUMAN |  | 14 | 0.97 | 0.007 | 16 | 0.1165 |
| **1120** | tr|Q6FI03|Q6FI03_HUMAN |  | 5 | 1.01 | 0.008 | 16 | 0.7056 |
| **1121** | tr|Q59FW9|Q59FW9_HUMAN |  | 3 | 1.03 | 0.019 | 16 | 0.3838 |
| **1122** | sp|Q9NS66|GP173_HUMAN |  | 1 | 0.99 | 0.041 | 16 | 0.8946 |
| **1123** | tr|Q0IJ56|Q0IJ56_HUMAN |  | 6 | 1.41 | 0.072 | 16 | 2.29E-05 |
| **1124** | tr|B4DGI3|B4DGI3_HUMAN |  | 3 | 0.93 | 0.023 | 16 | 0.06854 |
| **1125** | sp|O95295|SNAPN_HUMAN |  | 1 | 0.9 | 0.129 | 16 | 0.2655 |
| **1126** | tr|A8K5T0|A8K5T0_HUMAN |  | 1 | 0.94 | 0.049 | 16 | 0.268 |
| **1127** | sp|Q8NBP7|PCSK9_HUMAN |  | 3 | 0.96 | 0.038 | 16 | 0.4309 |
| **1128** | sp|Q13464|ROCK1_HUMAN |  | 7 | 1.03 | 0.002 | 16 | 0.04068 |
| **1129** | tr|D6RAC4|D6RAC4_HUMAN |  | 1 | 1.2 | 0.029 | 16 | 0.0002387 |
| **1130** | sp|Q9BRT3|MIEN1_HUMAN |  | 1 | 1.09 | 0.042 | 16 | 0.09697 |
| **1131** | sp|Q15181|IPYR_HUMAN |  | 6 | 1.1 | 0.004 | 16 | 1.54E-05 |
| **1132** | tr|J3KRH4|J3KRH4_HUMAN |  | 1 | 1.09 | 0.339 | 16 | 0.5352 |
| **1133** | tr|Q8TAR2|Q8TAR2_HUMAN |  | 1 | 0.89 | 0.033 | 16 | 0.02895 |
| **1134** | tr|H0YME5|H0YME5_HUMAN |  | 1 | 1.06 | 0.089 | 16 | 0.4546 |
| **1135** | tr|B7Z2R3|B7Z2R3_HUMAN |  | 1 | 1.1 | 0.067 | 16 | 0.1358 |
| **1136** | tr|A0A024R1S5|A0A024R1S5_HUMAN |  | 7 | 1 | 0.008 | 16 | 0.8957 |
| **1137** | tr|Q6PIX2|Q6PIX2_HUMAN |  | 2 | 1.39 | 0.081 | 16 | 6.36E-05 |
| **1138** | sp|Q9Y230|RUVB2_HUMAN |  | 24 | 0.95 | 0.021 | 16 | 0.1823 |
| **1139** | tr|Q6IBT1|Q6IBT1_HUMAN |  | 5 | 1.04 | 0.004 | 16 | 0.01771 |
| **1140** | tr|Q9BSS9|Q9BSS9_HUMAN |  | 2 | 0.99 | 0.022 | 16 | 0.7733 |
| **1141** | tr|B4DUI5|B4DUI5_HUMAN |  | 1 | 1.04 | 0.092 | 16 | 0.5858 |
| **1142** | tr|B4DUE0|B4DUE0_HUMAN |  | 3 | 1.13 | 0.008 | 16 | 5.14E-05 |
| **1143** | sp|P62306|RUXF_HUMAN |  | 1 | 1.33 | 0.055 | 16 | 5.13E-05 |
| **1144** | tr|B3KTT0|B3KTT0_HUMAN |  | 5 | 1.07 | 0.004 | 16 | 0.0003735 |
| **1145** | tr|A0A075B7B5|A0A075B7B5_HUMAN |  | 4 | 0.93 | 0.009 | 16 | 0.008805 |
| **1146** | tr|G1UI18|G1UI18_HUMAN |  | 1 | 1.08 | 0.13 | 16 | 0.397 |
| **1147** | sp|Q13564|ULA1_HUMAN |  | 5 | 1.18 | 0.011 | 16 | 5.65E-06 |
| **1148** | sp|Q13188|STK3_HUMAN |  | 1 | 1.14 | 0.03 | 16 | 0.005619 |
| **1149** | tr|U3KQ84|U3KQ84_HUMAN |  | 1 | 1.3 | 0.017 | 16 | 1.75E-07 |
| **1150** | tr|B2RBP3|B2RBP3_HUMAN |  | 6 | 1.21 | 0.015 | 16 | 4.84E-06 |
| **1151** | sp|P12270|TPR_HUMAN |  | 1 | 0.84 | 0.037 | 16 | 0.00477 |
| **1152** | tr|J3QSB7|J3QSB7_HUMAN |  | 2 | 0.88 | 0.059 | 16 | 0.05919 |
| **1153** | tr|B4E356|B4E356_HUMAN |  | 4 | 1.49 | 0.011 | 16 | 9.59E-12 |
| **1154** | sp|Q6QNY0|BL1S3_HUMAN |  | 1 | 0.95 | 0.137 | 16 | 0.6241 |
| **1155** | sp|Q9NQ79|CRAC1_HUMAN |  | 6 | 0.9 | 0.007 | 16 | 0.0002974 |
| **1156** | sp|Q6GMV2|SMYD5_HUMAN |  | 3 | 0.89 | 0.018 | 16 | 0.006324 |
| **1157** | tr|B4DZF1|B4DZF1_HUMAN |  | 2 | 1.06 | 0.009 | 16 | 0.03194 |
| **1158** | sp|P05198|IF2A_HUMAN |  | 11 | 0.92 | 0.008 | 16 | 0.002409 |
| **1159** | tr|J3QR68|J3QR68_HUMAN |  | 4 | 1 | 0.038 | 16 | 0.9264 |
| **1160** | tr|D3DVA8|D3DVA8_HUMAN |  | 2 | 1.01 | 0.023 | 16 | 0.8907 |
| **1161** | tr|Q6PKI6|Q6PKI6_HUMAN |  | 1 | 0.93 | 0.017 | 16 | 0.05456 |
| **1162** | sp|Q9ULE4|F184B_HUMAN |  | 1 | 0.92 | 0.085 | 16 | 0.3088 |
| **1163** | sp|Q99497|PARK7_HUMAN |  | 6 | 0.99 | 0.056 | 16 | 0.8718 |
| **1164** | sp|P62318|SMD3_HUMAN |  | 1 | 1.26 | 0.052 | 16 | 0.0003359 |
| **1165** | sp|Q6P1N0|C2D1A_HUMAN |  | 14 | 0.98 | 0.01 | 16 | 0.3596 |
| **1166** | tr|A0A096LPF0|A0A096LPF0_HUMAN |  | 1 | 1.24 | 0.044 | 16 | 0.0003839 |
| **1167** | sp|Q7L5Y1|ENOF1_HUMAN |  | 7 | 1.06 | 0.054 | 16 | 0.3237 |
| **1168** | sp|Q13185|CBX3_HUMAN |  | 1 | 1.38 | 0.108 | 16 | 0.0003439 |
| **1169** | tr|E9PQ98|E9PQ98_HUMAN |  | 2 | 1.05 | 0.012 | 16 | 0.07382 |
| **1170** | sp|Q8TEX9|IPO4_HUMAN |  | 9 | 0.96 | 0.023 | 16 | 0.3129 |
| **1171** | tr|F8VXL2|F8VXL2_HUMAN |  | 2 | 1.08 | 0.003 | 16 | 1.84E-05 |
| **1172** | sp|Q13103|SPP24_HUMAN |  | 3 | 1.09 | 0.033 | 16 | 0.07388 |
| **1173** | tr|B7Z3I9|B7Z3I9_HUMAN |  | 13 | 1.24 | 0.005 | 16 | 6.73E-10 |
| **1174** | sp|Q00610|CLH1_HUMAN |  | 59 | 0.95 | 0.007 | 16 | 0.01665 |
| **1175** | sp|Q5VYK3|ECM29_HUMAN |  | 9 | 0.99 | 0.014 | 16 | 0.6269 |
| **1176** | tr|B3KUI5|B3KUI5_HUMAN |  | 2 | 1.06 | 0.018 | 16 | 0.07995 |
| **1177** | sp|Q9BQE3|TBA1C_HUMAN |  | 1 | 0.96 | 0.107 | 16 | 0.5937 |
| **1178** | tr|B4DHK9|B4DHK9_HUMAN |  | 1 | 0.89 | 0.026 | 16 | 0.01745 |
| **1179** | sp|O75822|EIF3J_HUMAN |  | 6 | 0.95 | 0.009 | 16 | 0.0451 |
| **1180** | sp|Q06210|GFPT1_HUMAN |  | 1 | 1.01 | 0.049 | 16 | 0.834 |
| **1181** | sp|Q7Z2W4|ZCCHV_HUMAN |  | 4 | 0.79 | 0.011 | 16 | 8.55E-07 |
| **1182** | tr|Q9UL88|Q9UL88_HUMAN |  | 1 | 0.95 | 0.017 | 16 | 0.1184 |
| **1183** | tr|K7EKF6|K7EKF6_HUMAN |  | 3 | 0.97 | 0.055 | 16 | 0.6443 |
| **1184** | sp|Q8WYN0|ATG4A_HUMAN |  | 5 | 0.99 | 0.006 | 16 | 0.5175 |
| **1185** | sp|P02100|HBE_HUMAN |  | 1 | 0.74 | 0.037 | 16 | 8.34E-05 |
| **1186** | sp|O94804|STK10_HUMAN |  | 3 | 0.96 | 0.016 | 16 | 0.2184 |
| **1187** | sp|P04217|A1BG_HUMAN |  | 4 | 1.05 | 0.007 | 16 | 0.03165 |
| **1188** | tr|E9PPG9|E9PPG9_HUMAN |  | 1 | 1.22 | 0.199 | 16 | 0.06234 |
| **1189** | sp|Q6P9F7|LRC8B_HUMAN |  | 1 | 1.05 | 0.063 | 16 | 0.4273 |
| **1190** | tr|A2NX48|A2NX48_HUMAN |  | 1 | 1.79 | 0.152 | 16 | 6.88E-07 |
| **1191** | sp|Q15018|F175B_HUMAN |  | 5 | 1.07 | 0.008 | 16 | 0.006018 |
| **1192** | sp|P06753|TPM3_HUMAN |  | 1 | 0.97 | 0.07 | 16 | 0.7103 |
| **1193** | tr|Q59H12|Q59H12_HUMAN |  | 1 | 1.06 | 0.162 | 16 | 0.5589 |
| **1194** | tr|Q8N247|Q8N247_HUMAN |  | 1 | 0.98 | 1.357 | 16 | 0.9393 |
| **1195** | tr|H3BN77|H3BN77_HUMAN |  | 1 | 0.94 | 0.01 | 16 | 0.03183 |
| **1196** | tr|B7Z1L2|B7Z1L2_HUMAN |  | 1 | 0.93 | 0.098 | 16 | 0.3611 |
| **1197** | sp|P48059|LIMS1_HUMAN |  | 2 | 0.98 | 0.012 | 16 | 0.3982 |
| **1198** | tr|B4DR60|B4DR60_HUMAN |  | 1 | 0.82 | 0.178 | 16 | 0.1146 |
| **1199** | sp|Q9NVW2|RNF12_HUMAN |  | 1 | 1.35 | 0.366 | 16 | 0.03508 |
| **1200** | tr|Q9UM85|Q9UM85_HUMAN |  | 1 | 0.78 | 0.045 | 16 | 0.0007631 |
| **1201** | tr|B8ZZG1|B8ZZG1_HUMAN |  | 4 | 1.09 | 0.018 | 16 | 0.0199 |
| **1202** | tr|H7C4I0|H7C4I0_HUMAN |  | 1 | 1.02 | 0.065 | 16 | 0.7896 |
| **1203** | tr|D3JV41|D3JV41_HUMAN |  | 4 | 1.19 | 0.035 | 16 | 0.001245 |
| **1204** | sp|P51843|NR0B1_HUMAN |  | 1 | 0.71 | 0.243 | 16 | 0.03306 |
| **1205** | tr|A0A087X2C0|A0A087X2C0_HUMAN |  | 4 | 0.88 | 0.048 | 16 | 0.03789 |
| **1206** | tr|D6RBG2|D6RBG2_HUMAN |  | 1 | 1.25 | 0.12 | 16 | 0.01108 |
| **1207** | sp|P35908|K22E_HUMAN |  | 5 | 0.87 | 0.061 | 16 | 0.04741 |
| **1208** | sp|Q16531|DDB1_HUMAN |  | 35 | 1 | 0.004 | 16 | 0.8987 |
| **1209** | tr|F6S289|F6S289_HUMAN |  | 1 | 0.95 | 0.009 | 16 | 0.05473 |
| **1210** | sp|P61006|RAB8A_HUMAN |  | 1 | 0.89 | 0.028 | 16 | 0.02012 |
| **1211** | sp|Q8NHG7|SVIP_HUMAN |  | 1 | 0.84 | 0.031 | 16 | 0.00263 |
| **1212** | sp|Q9UL46|PSME2_HUMAN |  | 11 | 0.98 | 0.051 | 16 | 0.7095 |
| **1213** | sp|Q93034|CUL5_HUMAN |  | 7 | 0.89 | 0.022 | 16 | 0.01005 |
| **1214** | tr|F8W1S5|F8W1S5_HUMAN |  | 1 | 0.97 | 0.017 | 16 | 0.3295 |
| **1215** | sp|Q8NG06|TRI58_HUMAN |  | 8 | 0.91 | 0.012 | 16 | 0.00643 |
| **1216** | tr|B4DYX0|B4DYX0_HUMAN |  | 1 | 0.88 | 0.036 | 16 | 0.01906 |
| **1217** | tr|H0YGG4|H0YGG4_HUMAN |  | 2 | 0.88 | 0.023 | 16 | 0.00703 |
| **1218** | tr|F8W1I5|F8W1I5_HUMAN |  | 1 | 1.37 | 0.009 | 16 | 1.01E-10 |
| **1219** | tr|S4R456|S4R456_HUMAN |  | 1 | 0.98 | 0.033 | 16 | 0.6345 |
| **1220** | sp|Q14738|2A5D_HUMAN |  | 7 | 1.06 | 0.01 | 16 | 0.03849 |
| **1221** | sp|Q10567|AP1B1_HUMAN |  | 2 | 1.01 | 0.012 | 16 | 0.7074 |
| **1222** | tr|Q5F2F8|Q5F2F8_HUMAN |  | 1 | 0.99 | 0.113 | 16 | 0.885 |
| **1223** | tr|B7Z6B3|B7Z6B3_HUMAN |  | 1 | 0.93 | 0.029 | 16 | 0.1007 |
| **1224** | tr|B4DII5|B4DII5_HUMAN |  | 9 | 0.91 | 0.004 | 16 | 9.09E-05 |
| **1225** | tr|B2R888|B2R888_HUMAN |  | 4 | 0.86 | 0.041 | 16 | 0.01527 |
| **1226** | sp|P30566|PUR8_HUMAN |  | 1 | 0.94 | 0.146 | 16 | 0.5592 |
| **1227** | tr|H0Y7N2|H0Y7N2_HUMAN |  | 1 | 1.28 | 0.091 | 16 | 0.002059 |
| **1228** | tr|F8WCU1|F8WCU1_HUMAN |  | 1 | 1 | 0.042 | 15 | 0.9331 |
| **1229** | tr|Q86TY5|Q86TY5_HUMAN |  | 3 | 1.04 | 0.036 | 16 | 0.4369 |
| **1230** | sp|P42345|MTOR_HUMAN |  | 1 | 0.92 | 0.017 | 16 | 0.03518 |
| **1231** | tr|Q05BS0|Q05BS0_HUMAN |  | 3 | 0.91 | 0.01 | 16 | 0.00279 |
| **1232** | tr|E7ETW3|E7ETW3_HUMAN |  | 1 | 1.17 | 0.428 | 16 | 0.3195 |
| **1233** | tr|B7ZL14|B7ZL14_HUMAN |  | 1 | 0.97 | 0.009 | 16 | 0.2227 |
| **1234** | tr|B4E0B7|B4E0B7_HUMAN |  | 4 | 0.98 | 0.01 | 16 | 0.4725 |
| **1235** | sp|P12724|ECP_HUMAN |  | 4 | 1.86 | 0.382 | 16 | 5.13E-05 |
| **1236** | sp|O00399|DCTN6_HUMAN |  | 1 | 0.99 | 0.022 | 16 | 0.7666 |
| **1237** | tr|H0YJC6|H0YJC6_HUMAN |  | 1 | 0.9 | 0.04 | 16 | 0.05916 |
| **1238** | sp|Q5TDH0|DDI2_HUMAN |  | 8 | 1.14 | 0.011 | 16 | 7.37E-05 |
| **1239** | tr|Q13707|Q13707_HUMAN |  | 1 | 1.06 | 0.045 | 16 | 0.2495 |
| **1240** | sp|Q13608|PEX6_HUMAN |  | 1 | 0.81 | 0.133 | 16 | 0.05211 |
| **1241** | tr|H0YDS4|H0YDS4_HUMAN |  | 1 | 0.97 | 0.023 | 16 | 0.3834 |
| **1242** | tr|B7Z5S9|B7Z5S9_HUMAN |  | 12 | 0.98 | 0.007 | 16 | 0.4299 |
| **1243** | sp|Q9Y623|MYH4_HUMAN |  | 1 | 1.05 | 0.075 | 16 | 0.4486 |
| **1244** | tr|B3KP61|B3KP61_HUMAN |  | 1 | 1.64 | 0.906 | 16 | 0.01661 |
| **1245** | tr|A8MX94|A8MX94_HUMAN |  | 3 | 0.97 | 0.015 | 16 | 0.4017 |
| **1246** | sp|Q15166|PON3_HUMAN |  | 4 | 1 | 0.004 | 16 | 0.9014 |
| **1247** | sp|P07954|FUMH_HUMAN |  | 1 | 0.83 | 0.063 | 16 | 0.01677 |
| **1248** | sp|Q96H20|SNF8_HUMAN |  | 1 | 1.01 | 0.172 | 16 | 0.9032 |
| **1249** | tr|H0YJP0|H0YJP0_HUMAN |  | 3 | 0.97 | 0.077 | 16 | 0.7074 |
| **1250** | tr|H7BZK8|H7BZK8_HUMAN |  | 1 | 1.08 | 0.113 | 16 | 0.3384 |
| **1251** | sp|O00507|USP9Y_HUMAN |  | 1 | 0.68 | 0.041 | 16 | 1.49E-05 |
| **1252** | tr|H0UID5|H0UID5_HUMAN |  | 13 | 0.95 | 0.008 | 16 | 0.03285 |
| **1253** | sp|O00629|IMA3_HUMAN |  | 9 | 0.96 | 0.005 | 16 | 0.03006 |
| **1254** | tr|H3BMQ8|H3BMQ8_HUMAN |  | 1 | 1.07 | 0.056 | 16 | 0.2554 |
| **1255** | sp|P01009|A1AT_HUMAN |  | 16 | 1.19 | 0.503 | 16 | 0.2954 |
| **1256** | tr|J3KRP0|J3KRP0_HUMAN |  | 12 | 1.02 | 0.02 | 16 | 0.5543 |
| **1257** | tr|Q9HCC1|Q9HCC1_HUMAN |  | 1 | 0.88 | 0.045 | 16 | 0.03376 |
| **1258** | tr|B3KXI1|B3KXI1_HUMAN |  | 1 | 0.96 | 0.078 | 16 | 0.5966 |
| **1259** | sp|P69892|HBG2_HUMAN |  | 1 | 1 | 0.083 | 16 | 0.9565 |
| **1260** | tr|Q9UL70|Q9UL70_HUMAN |  | 1 | 0.92 | 0.035 | 16 | 0.09831 |
| **1261** | sp|Q13617|CUL2_HUMAN |  | 15 | 1.12 | 0.011 | 16 | 0.0003136 |
| **1262** | tr|C9JPM4|C9JPM4_HUMAN |  | 2 | 0.93 | 0.02 | 16 | 0.05149 |
| **1263** | sp|P01137|TGFB1_HUMAN |  | 2 | 0.95 | 0.021 | 16 | 0.2166 |
| **1264** | tr|Q9NSK3|Q9NSK3_HUMAN |  | 4 | 1.07 | 0.011 | 16 | 0.02062 |
| **1265** | tr|B4DWY1|B4DWY1_HUMAN |  | 1 | 1.14 | 0.093 | 16 | 0.08143 |
| **1266** | sp|P07900|HS90A_HUMAN |  | 15 | 1.21 | 0.013 | 16 | 3.40E-06 |
| **1267** | tr|B3KVC6|B3KVC6_HUMAN |  | 1 | 0.9 | 0.008 | 16 | 0.0003768 |
| **1268** | tr|V9HW62|V9HW62_HUMAN |  | 6 | 0.94 | 0.004 | 16 | 0.004348 |
| **1269** | sp|Q6ZS30|NBEL1_HUMAN |  | 1 | 1 | 0.073 | 16 | 0.9507 |
| **1270** | tr|Q8NF51|Q8NF51_HUMAN |  | 1 | 0.88 | 0.097 | 16 | 0.1441 |
| **1271** | sp|Q15744|CEBPE_HUMAN |  | 1 | 0.79 | 0.005 | 16 | 5.47E-09 |
| **1272** | tr|B4E184|B4E184_HUMAN |  | 9 | 1 | 0.005 | 16 | 0.9973 |
| **1273** | tr|X6RCC3|X6RCC3_HUMAN |  | 1 | 0.86 | 0.018 | 16 | 0.0007394 |
| **1274** | sp|Q9UPU5|UBP24_HUMAN |  | 27 | 0.97 | 0.006 | 16 | 0.152 |
| **1275** | tr|Q5HYI5|Q5HYI5_HUMAN |  | 1 | 0.87 | 0.023 | 16 | 0.002682 |
| **1276** | tr|B4DEW5|B4DEW5_HUMAN |  | 1 | 1.14 | 0.102 | 16 | 0.09592 |
| **1277** | sp|P01612|KV120_HUMAN |  | 2 | 0.93 | 0.03 | 16 | 0.1427 |
| **1278** | sp|P02776|PLF4_HUMAN |  | 1 | 0.99 | 0.023 | 16 | 0.7024 |
| **1279** | sp|P54577|SYYC_HUMAN |  | 3 | 1.01 | 0.034 | 16 | 0.8954 |
| **1280** | tr|A2JA16|A2JA16_HUMAN |  | 1 | 0.93 | 0.011 | 16 | 0.01828 |
| **1281** | tr|Q53SQ6|Q53SQ6_HUMAN |  | 4 | 1.07 | 0.009 | 16 | 0.009369 |
| **1282** | tr|C9IZP8|C9IZP8_HUMAN |  | 1 | 1.11 | 0.094 | 16 | 0.1575 |
| **1283** | tr|Q4VB86|Q4VB86_HUMAN |  | 15 | 0.81 | 0.019 | 16 | 7.20E-05 |
| **1284** | sp|Q9NZJ9|NUDT4_HUMAN |  | 3 | 0.97 | 0.005 | 16 | 0.1147 |
| **1285** | sp|O75340|PDCD6_HUMAN |  | 4 | 1.09 | 0.006 | 16 | 0.0002008 |
| **1286** | tr|E9PFD4|E9PFD4_HUMAN |  | 1 | 0.91 | 0.097 | 16 | 0.2435 |
| **1287** | sp|P01781|HV320_HUMAN |  | 1 | 1.01 | 0.035 | 16 | 0.8657 |
| **1288** | tr|Q5UGI6|Q5UGI6_HUMAN |  | 6 | 0.91 | 0.009 | 16 | 0.001197 |
| **1289** | sp|Q6XQN6|PNCB_HUMAN |  | 13 | 1.06 | 0.005 | 16 | 0.002834 |
| **1290** | sp|P07237|PDIA1_HUMAN |  | 1 | 1.51 | 0.036 | 16 | 1.72E-08 |
| **1291** | tr|Q9NUN4|Q9NUN4_HUMAN |  | 1 | 1.17 | 0.046 | 16 | 0.005559 |
| **1292** | tr|Q68DY8|Q68DY8_HUMAN |  | 1 | 1.04 | 0.033 | 16 | 0.4277 |
| **1293** | sp|Q9P0R6|GSKIP_HUMAN |  | 1 | 1.04 | 0.039 | 16 | 0.4043 |
| **1294** | sp|O76003|GLRX3_HUMAN |  | 8 | 1.16 | 0.02 | 16 | 0.0003062 |
| **1295** | sp|P19474|RO52_HUMAN |  | 6 | 0.99 | 0.003 | 16 | 0.5442 |
| **1296** | tr|Q53G71|Q53G71_HUMAN |  | 9 | 1.09 | 0.016 | 16 | 0.01133 |
| **1297** | sp|Q8N7G0|PO5F2_HUMAN |  | 1 | 1.02 | 0.026 | 16 | 0.6862 |
| **1298** | sp|Q8NBF2|NHLC2_HUMAN |  | 6 | 0.99 | 0.002 | 16 | 0.3981 |
| **1299** | sp|Q9Y333|LSM2_HUMAN |  | 1 | 1.07 | 0.019 | 16 | 0.06048 |
| **1300** | tr|H0YLI6|H0YLI6_HUMAN |  | 1 | 0.66 | 0.027 | 16 | 5.83E-07 |
| **1301** | tr|B3KX15|B3KX15_HUMAN |  | 1 | 1.51 | 0.24 | 16 | 0.0008793 |
| **1302** | sp|Q9HBH1|DEFM_HUMAN |  | 1 | 0.92 | 0.101 | 16 | 0.3502 |
| **1303** | tr|B5KVR8|B5KVR8_HUMAN |  | 4 | 0.95 | 0.006 | 16 | 0.02613 |
| **1304** | tr|B0QYA8|B0QYA8_HUMAN |  | 1 | 0.95 | 0.112 | 16 | 0.5578 |
| **1305** | sp|Q96MG7|MAGG1_HUMAN |  | 1 | 1.02 | 0.099 | 16 | 0.8274 |
| **1306** | tr|Q1HP67|Q1HP67_HUMAN |  | 8 | 0.89 | 0.006 | 16 | 3.25E-05 |
| **1307** | sp|Q15126|PMVK_HUMAN |  | 1 | 0.79 | 0.035 | 16 | 0.0004081 |
| **1308** | tr|Q59F49|Q59F49_HUMAN |  | 1 | 1.01 | 0.044 | 16 | 0.8309 |
| **1309** | sp|P08697|A2AP_HUMAN |  | 8 | 1.09 | 0.018 | 16 | 0.01706 |
| **1310** | sp|P49247|RPIA_HUMAN |  | 4 | 1.03 | 0.028 | 16 | 0.4565 |
| **1311** | sp|P67936|TPM4_HUMAN |  | 5 | 0.91 | 0.007 | 16 | 0.001105 |
| **1312** | tr|B7Z7L4|B7Z7L4_HUMAN |  | 5 | 1.04 | 0.005 | 16 | 0.06008 |
| **1313** | tr|K7EJE6|K7EJE6_HUMAN |  | 1 | 0.87 | 0.019 | 16 | 0.001857 |
| **1314** | tr|Q2TU64|Q2TU64_HUMAN |  | 1 | 0.92 | 0.006 | 16 | 0.0003969 |
| **1315** | tr|Q9BPW0|Q9BPW0_HUMAN |  | 1 | 0.96 | 0.132 | 16 | 0.6957 |
| **1316** | tr|B7Z637|B7Z637_HUMAN |  | 8 | 0.93 | 0.007 | 16 | 0.005186 |
| **1317** | sp|Q96MD2|CL066_HUMAN |  | 1 | 0.92 | 0.084 | 16 | 0.267 |
| **1318** | tr|E9PS17|E9PS17_HUMAN |  | 12 | 1 | 0.003 | 16 | 0.8726 |
| **1319** | tr|B4E3V2|B4E3V2_HUMAN |  | 1 | 0.99 | 0.032 | 16 | 0.8764 |
| **1320** | sp|P23743|DGKA_HUMAN |  | 7 | 1.05 | 0.008 | 16 | 0.05618 |
| **1321** | sp|Q7Z333|SETX_HUMAN |  | 1 | 1.07 | 0.06 | 16 | 0.244 |
| **1322** | sp|P02763|A1AG1_HUMAN |  | 1 | 1.09 | 0.057 | 16 | 0.1499 |
| **1323** | sp|Q8NFR7|CC148_HUMAN |  | 1 | 2.79 | 4.783 | 16 | 0.005182 |
| **1324** | sp|P23109|AMPD1_HUMAN |  | 1 | 1.08 | 0.029 | 16 | 0.08907 |
| **1325** | tr|F8W9B8|F8W9B8_HUMAN |  | 1 | 1.15 | 0.026 | 16 | 0.002237 |
| **1326** | tr|Q9BTS4|Q9BTS4_HUMAN |  | 1 | 0.9 | 0.316 | 16 | 0.4984 |
| **1327** | tr|Q5TE63|Q5TE63_HUMAN |  | 1 | 1.12 | 0.019 | 16 | 0.004362 |
| **1328** | sp|Q8IUR0|TPPC5_HUMAN |  | 1 | 1.04 | 0.1 | 16 | 0.6091 |
| **1329** | tr|B4DS69|B4DS69_HUMAN |  | 1 | 1.17 | 0.155 | 16 | 0.09746 |
| **1330** | sp|Q5T4S7|UBR4_HUMAN |  | 66 | 0.85 | 0.005 | 16 | 4.05E-07 |
| **1331** | tr|F2Z2Y6|F2Z2Y6_HUMAN |  | 1 | 1.34 | 0.026 | 16 | 3.82E-07 |
| **1332** | tr|B4E0P8|B4E0P8_HUMAN |  | 4 | 1.04 | 0.013 | 16 | 0.153 |
| **1333** | tr|B4DV85|B4DV85_HUMAN |  | 1 | 1.05 | 0.011 | 16 | 0.06216 |
| **1334** | sp|P46783|RS10_HUMAN |  | 2 | 1.03 | 0.036 | 16 | 0.4983 |
| **1335** | tr|Q6ZUM0|Q6ZUM0_HUMAN |  | 1 | 1.03 | 0.08 | 16 | 0.6446 |
| **1336** | sp|Q96C19|EFHD2_HUMAN |  | 3 | 1.04 | 0.014 | 16 | 0.2532 |
| **1337** | sp|Q9BUH6|CI142_HUMAN |  | 4 | 0.79 | 0.036 | 16 | 0.0003965 |
| **1338** | sp|Q9NQH7|XPP3_HUMAN |  | 1 | 1.25 | 0.011 | 16 | 9.20E-08 |
| **1339** | sp|Q86U17|SPA11_HUMAN |  | 1 | 0.86 | 0.022 | 16 | 0.002344 |
| **1340** | tr|A1A5C4|A1A5C4_HUMAN |  | 1 | 1.06 | 0.054 | 16 | 0.3151 |
| **1341** | sp|Q5R372|RBG1L_HUMAN |  | 5 | 1.02 | 0.011 | 16 | 0.4024 |
| **1342** | tr|B3KNB9|B3KNB9_HUMAN |  | 5 | 1.06 | 0.011 | 16 | 0.04422 |
| **1343** | sp|Q9BXJ0|C1QT5_HUMAN |  | 1 | 1.04 | 0.125 | 12 | 0.6874 |
| **1344** | sp|P12259|FA5_HUMAN |  | 6 | 1.14 | 0.009 | 16 | 2.47E-05 |
| **1345** | tr|A0A0A0MTR1|A0A0A0MTR1_HUMAN |  | 2 | 0.86 | 0.011 | 16 | 7.61E-05 |
| **1346** | tr|B1AP52|B1AP52_HUMAN |  | 1 | 0.93 | 0.107 | 16 | 0.4032 |
| **1347** | tr|B4E2Y2|B4E2Y2_HUMAN |  | 5 | 1.01 | 0.008 | 16 | 0.7732 |
| **1348** | tr|I6L957|I6L957_HUMAN |  | 1 | 1.8 | 0.262 | 16 | 1.54E-05 |
| **1349** | tr|B4DYT5|B4DYT5_HUMAN |  | 1 | 0.88 | 0.034 | 16 | 0.01622 |
| **1350** | sp|Q9Y6I8|PXMP4_HUMAN |  | 1 | 3.3 | 9.192 | 16 | 0.008293 |
| **1351** | tr|H7C5E3|H7C5E3_HUMAN |  | 1 | 1.19 | 0.108 | 16 | 0.03781 |
| **1352** | sp|Q6ZUJ8|BCAP_HUMAN |  | 5 | 0.93 | 0.006 | 16 | 0.00258 |
| **1353** | tr|H0UI76|H0UI76_HUMAN |  | 1 | 0.78 | 0.039 | 16 | 0.0004685 |
| **1354** | tr|Q6LAM1|Q6LAM1_HUMAN |  | 1 | 1.03 | 0.03 | 16 | 0.443 |
| **1355** | tr|Q6ZTF9|Q6ZTF9_HUMAN |  | 2 | 0.82 | 0.023 | 16 | 0.0002223 |
| **1356** | sp|P08237|PFKAM_HUMAN |  | 6 | 0.92 | 0.002 | 16 | 1.62E-05 |
| **1357** | sp|P23083|HV103_HUMAN |  | 1 | 1.02 | 0.114 | 16 | 0.8017 |
| **1358** | tr|H7BZK6|H7BZK6_HUMAN |  | 1 | 0.87 | 0.085 | 16 | 0.09187 |
| **1359** | tr|Q5JZ02|Q5JZ02_HUMAN |  | 1 | 1.09 | 0.079 | 16 | 0.2232 |
| **1360** | sp|Q9UMX5|NENF_HUMAN |  | 1 | 6.6 | 14.832 | 16 | 3.39E-05 |
| **1361** | tr|Q9BS45|Q9BS45_HUMAN |  | 1 | 1.16 | 0.449 | 16 | 0.3548 |
| **1362** | sp|Q96PU5|NED4L_HUMAN |  | 1 | 0.97 | 0.008 | 16 | 0.1675 |
| **1363** | tr|H0YGL6|H0YGL6_HUMAN |  | 3 | 0.99 | 0.005 | 16 | 0.4924 |
| **1364** | tr|B4E1J2|B4E1J2_HUMAN |  | 1 | 0.93 | 0.01 | 16 | 0.01331 |
| **1365** | sp|Q9HD89|RETN_HUMAN |  | 1 | 1.84 | 0.643 | 16 | 0.000817 |
| **1366** | sp|P43652|AFAM_HUMAN |  | 8 | 1.04 | 0.003 | 16 | 0.009722 |
| **1367** | sp|P04004|VTNC_HUMAN |  | 9 | 0.96 | 0.005 | 16 | 0.04355 |
| **1368** | sp|P01620|KV302_HUMAN |  | 1 | 0.87 | 0.011 | 16 | 0.0001262 |
| **1369** | tr|I7HJJ0|I7HJJ0_HUMAN |  | 1 | 1.26 | 0.046 | 16 | 0.0002552 |
| **1370** | tr|F5GXM3|F5GXM3_HUMAN |  | 5 | 1.03 | 0.028 | 16 | 0.5439 |
| **1371** | tr|Q9UL85|Q9UL85_HUMAN |  | 1 | 0.92 | 0.043 | 16 | 0.1304 |
| **1372** | sp|Q15485|FCN2_HUMAN |  | 4 | 0.95 | 0.013 | 16 | 0.07426 |
| **1373** | tr|L0R6E1|L0R6E1_HUMAN |  | 1 | 1.06 | 0.037 | 16 | 0.2576 |
| **1374** | tr|B4DWM6|B4DWM6_HUMAN |  | 1 | 1 | 0.036 | 16 | 0.937 |
| **1375** | tr|B3KSI4|B3KSI4_HUMAN |  | 6 | 0.98 | 0.026 | 16 | 0.5796 |
| **1376** | sp|Q9UGR2|Z3H7B_HUMAN |  | 1 | 1.16 | 0.105 | 16 | 0.06468 |
| **1377** | sp|P63208|SKP1_HUMAN |  | 1 | 1.22 | 0.019 | 16 | 1.42E-05 |
| **1378** | sp|Q7RTS7|K2C74_HUMAN |  | 2 | 1.04 | 0.069 | 16 | 0.5133 |
| **1379** | sp|Q9BX10|GTPB2_HUMAN |  | 1 | 1.04 | 0.006 | 16 | 0.09292 |
| **1380** | sp|P06730|IF4E_HUMAN |  | 3 | 0.99 | 0.015 | 16 | 0.736 |
| **1381** | tr|B4E304|B4E304_HUMAN |  | 10 | 0.95 | 0.002 | 16 | 0.0003564 |
| **1382** | sp|P04921|GLPC_HUMAN |  | 1 | 0.86 | 0.027 | 16 | 0.004519 |
| **1383** | tr|B2RA57|B2RA57_HUMAN |  | 2 | 0.95 | 0.034 | 16 | 0.2693 |
| **1384** | tr|B4E3E6|B4E3E6_HUMAN |  | 2 | 1.18 | 0.025 | 16 | 0.0003328 |
| **1385** | sp|Q9H5L6|THAP9_HUMAN |  | 1 | 1.12 | 0.039 | 16 | 0.03125 |
| **1386** | sp|P11226|MBL2_HUMAN |  | 5 | 1 | 0.011 | 16 | 0.8893 |
| **1387** | sp|Q08495|DEMA_HUMAN |  | 7 | 1 | 0.013 | 16 | 0.9137 |
| **1388** | tr|A0A024QZE7|A0A024QZE7_HUMAN |  | 1 | 0.98 | 0.014 | 16 | 0.4465 |
| **1389** | sp|Q16819|MEP1A_HUMAN |  | 1 | 1.05 | 0.073 | 16 | 0.5014 |
| **1390** | sp|O15511|ARPC5_HUMAN |  | 1 | 1.25 | 0.018 | 16 | 2.26E-06 |
| **1391** | tr|K7EK33|K7EK33_HUMAN |  | 2 | 1.16 | 0.008 | 16 | 1.97E-06 |
| **1392** | tr|Q6N092|Q6N092_HUMAN |  | 1 | 1.09 | 0.01 | 16 | 0.002197 |
| **1393** | sp|Q9NUL7|DDX28_HUMAN |  | 1 | 1.03 | 0.086 | 16 | 0.69 |
| **1394** | sp|P13645|K1C10_HUMAN |  | 14 | 0.93 | 0.087 | 16 | 0.3287 |
| **1395** | tr|E7EQB2|E7EQB2_HUMAN |  | 2 | 3.26 | 1.119 | 16 | 3.73E-07 |
| **1396** | tr|B3KMF4|B3KMF4_HUMAN |  | 1 | 0.99 | 0.134 | 16 | 0.9497 |
| **1397** | tr|A3RKG7|A3RKG7_HUMAN |  | 6 | 1.01 | 0.013 | 16 | 0.8434 |
| **1398** | sp|Q15366|PCBP2_HUMAN |  | 1 | 0.96 | 0.012 | 16 | 0.1408 |
| **1399** | sp|Q5T1M5|FKB15_HUMAN |  | 2 | 1.04 | 0.035 | 16 | 0.4041 |
| **1400** | tr|H0Y5R6|H0Y5R6_HUMAN |  | 2 | 1.34 | 0.059 | 16 | 5.45E-05 |
| **1401** | tr|B4E1M5|B4E1M5_HUMAN |  | 1 | 1.19 | 0.016 | 16 | 3.58E-05 |
| **1402** | tr|B4DST5|B4DST5_HUMAN |  | 3 | 1.12 | 0.028 | 16 | 0.01328 |
| **1403** | tr|A0A024R889|A0A024R889_HUMAN |  | 1 | 1.03 | 0.039 | 16 | 0.5693 |
| **1404** | sp|Q96HB5|CC120_HUMAN |  | 1 | 1.08 | 0.009 | 16 | 0.003308 |
| **1405** | tr|F5H3C5|F5H3C5_HUMAN |  | 1 | 1.1 | 0.067 | 16 | 0.1461 |
| **1406** | tr|Q96CV8|Q96CV8_HUMAN |  | 2 | 1.03 | 0.039 | 16 | 0.5949 |
| **1407** | sp|P69905|HBA_HUMAN |  | 2 | 0.87 | 0.044 | 16 | 0.02357 |
| **1408** | tr|Q69YG3|Q69YG3_HUMAN |  | 1 | 1.09 | 0.047 | 16 | 0.1206 |
| **1409** | sp|O00186|STXB3_HUMAN |  | 1 | 0.96 | 0.01 | 16 | 0.1432 |
| **1410** | tr|A5CKE2|A5CKE2_HUMAN |  | 2 | 0.91 | 0.066 | 16 | 0.1933 |
| **1411** | tr|G3V3G7|G3V3G7_HUMAN |  | 1 | 1.04 | 0.088 | 16 | 0.5923 |
| **1412** | tr|A2ACR1|A2ACR1_HUMAN |  | 3 | 1.07 | 0.014 | 16 | 0.02601 |
| **1413** | tr|B7Z2S1|B7Z2S1_HUMAN |  | 1 | 0.97 | 0.018 | 16 | 0.3709 |
| **1414** | sp|Q9Y5S9|RBM8A_HUMAN |  | 2 | 1.23 | 0.008 | 16 | 2.80E-08 |
| **1415** | tr|B7Z4P9|B7Z4P9_HUMAN |  | 2 | 1.1 | 0.01 | 16 | 0.0008608 |
| **1416** | tr|G3V2S6|G3V2S6_HUMAN |  | 3 | 0.81 | 0.013 | 16 | 6.63E-06 |
| **1417** | tr|Q6MZQ6|Q6MZQ6_HUMAN |  | 1 | 1.08 | 0.163 | 16 | 0.4398 |
| **1418** | tr|Q4ZFW8|Q4ZFW8_HUMAN |  | 2 | 0.87 | 0.027 | 16 | 0.007536 |
| **1419** | tr|F8VXB0|F8VXB0_HUMAN |  | 2 | 1 | 0.077 | 16 | 0.9625 |
| **1420** | tr|K7EK91|K7EK91_HUMAN |  | 1 | 1 | 0.059 | 16 | 0.9461 |
| **1421** | tr|B4DW89|B4DW89_HUMAN |  | 1 | 2.09 | 0.344 | 16 | 2.09E-06 |
| **1422** | sp|P04196|HRG_HUMAN |  | 11 | 0.9 | 0.016 | 16 | 0.00556 |
| **1423** | tr|Q4W5Q4|Q4W5Q4_HUMAN |  | 9 | 1.01 | 0.005 | 16 | 0.4152 |
| **1424** | tr|Q6NS95|Q6NS95_HUMAN |  | 2 | 1.03 | 0.011 | 16 | 0.2151 |
| **1425** | sp|P49189|AL9A1_HUMAN |  | 11 | 1.08 | 0.002 | 16 | 1.59E-06 |
| **1426** | sp|Q9UHG2|PCSK1_HUMAN |  | 1 | 1.1 | 0.153 | 16 | 0.336 |
| **1427** | sp|Q9NQX3|GEPH_HUMAN |  | 10 | 0.95 | 0.004 | 16 | 0.005218 |
| **1428** | sp|Q96H79|ZCCHL_HUMAN |  | 1 | 1.07 | 0.058 | 16 | 0.2593 |
| **1429** | sp|P49427|UB2R1_HUMAN |  | 1 | 0.75 | 0.023 | 16 | 9.13E-06 |
| **1430** | tr|Q9UQD4|Q9UQD4_HUMAN |  | 4 | 1.05 | 0.01 | 16 | 0.0612 |
| **1431** | tr|C9J4L5|C9J4L5_HUMAN |  | 1 | 1.04 | 0.036 | 16 | 0.3888 |
| **1432** | tr|A0A024RDB8|A0A024RDB8_HUMAN |  | 3 | 1.14 | 0.013 | 16 | 0.0002143 |
| **1433** | sp|O00410|IPO5_HUMAN |  | 22 | 0.87 | 0.003 | 16 | 1.53E-07 |
| **1434** | sp|P04430|KV122_HUMAN |  | 1 | 0.92 | 0.057 | 16 | 0.1741 |
| **1435** | tr|G3V3B5|G3V3B5_HUMAN |  | 1 | 0.88 | 0.058 | 16 | 0.0578 |
| **1436** | sp|P54920|SNAA_HUMAN |  | 16 | 1.07 | 0.012 | 16 | 0.02233 |
| **1437** | tr|A2J1N7|A2J1N7_HUMAN |  | 1 | 0.96 | 0.031 | 16 | 0.3894 |
| **1438** | sp|O43257|ZNHI1_HUMAN |  | 1 | 0.98 | 0.08 | 16 | 0.7903 |
| **1439** | tr|H7C3T4|H7C3T4_HUMAN |  | 1 | 1.13 | 0.043 | 16 | 0.02874 |
| **1440** | sp|O75348|VATG1_HUMAN |  | 3 | 1.11 | 0.011 | 16 | 0.0009566 |
| **1441** | tr|Q4LE74|Q4LE74_HUMAN |  | 1 | 1.24 | 0.042 | 16 | 0.000296 |
| **1442** | sp|O60784|TOM1_HUMAN |  | 3 | 1.01 | 0.025 | 16 | 0.837 |
| **1443** | sp|Q13057|COASY_HUMAN |  | 3 | 1 | 0.006 | 16 | 0.8183 |
| **1444** | tr|F8VWH5|F8VWH5_HUMAN |  | 1 | 1.05 | 0.178 | 16 | 0.6176 |
| **1445** | sp|P00488|F13A_HUMAN |  | 13 | 1.04 | 0.006 | 16 | 0.07207 |
| **1446** | sp|Q9NQS1|AVEN_HUMAN |  | 1 | 0.79 | 0.314 | 16 | 0.1515 |
| **1447** | tr|B2RD51|B2RD51_HUMAN |  | 3 | 1.03 | 0.025 | 16 | 0.5132 |
| **1448** | sp|P28072|PSB6_HUMAN |  | 5 | 1.07 | 0.009 | 16 | 0.00899 |
| **1449** | tr|Q59H55|Q59H55_HUMAN |  | 1 | 1.05 | 0.473 | 16 | 0.7775 |
| **1450** | tr|A0A087WW77|A0A087WW77_HUMAN |  | 1 | 0.82 | 0.089 | 16 | 0.02677 |
| **1451** | tr|D6RID6|D6RID6_HUMAN |  | 1 | 1.17 | 0.065 | 16 | 0.02019 |
| **1452** | tr|B4DRD5|B4DRD5_HUMAN |  | 1 | 0.68 | 0.063 | 16 | 0.0001294 |
| **1453** | tr|R4GMR5|R4GMR5_HUMAN |  | 12 | 0.98 | 0.004 | 16 | 0.2098 |
| **1454** | tr|H7C034|H7C034_HUMAN |  | 1 | 1.11 | 0.077 | 16 | 0.1218 |
| **1455** | tr|A0A087X0Q4|A0A087X0Q4_HUMAN |  | 1 | 0.72 | 0.074 | 16 | 0.0008425 |
| **1456** | sp|Q5QJ74|TBCEL_HUMAN |  | 8 | 0.98 | 0.022 | 16 | 0.5648 |
| **1457** | tr|J3KSL8|J3KSL8_HUMAN |  | 2 | 0.98 | 0.058 | 16 | 0.7062 |
| **1458** | tr|H0YB22|H0YB22_HUMAN |  | 1 | 1 | 0.04 | 16 | 0.9842 |
| **1459** | tr|B4DRV5|B4DRV5_HUMAN |  | 2 | 0.93 | 0.008 | 16 | 0.005049 |
| **1460** | tr|B4E0N9|B4E0N9_HUMAN |  | 1 | 2.02 | 1.7 | 16 | 0.0071 |
| **1461** | tr|Q5T0D2|Q5T0D2_HUMAN |  | 1 | 1.27 | 0.067 | 16 | 0.0008348 |
| **1462** | tr|J3KRK5|J3KRK5_HUMAN |  | 1 | 0.62 | 0.029 | 16 | 2.29E-07 |
| **1463** | tr|A8K2W3|A8K2W3_HUMAN |  | 11 | 1.17 | 0.02 | 16 | 0.0002259 |
| **1464** | sp|P52209|6PGD_HUMAN |  | 4 | 0.88 | 0.018 | 16 | 0.00278 |
| **1465** | tr|H7BXI1|H7BXI1_HUMAN |  | 2 | 0.84 | 0.027 | 16 | 0.001224 |
| **1466** | tr|A5D905|A5D905_HUMAN |  | 1 | 0.98 | 0.046 | 16 | 0.7644 |
| **1467** | sp|Q9BTY7|HGH1_HUMAN |  | 11 | 0.96 | 0.007 | 16 | 0.07147 |
| **1468** | tr|Q8IUL9|Q8IUL9_HUMAN |  | 1 | 0.95 | 0.017 | 16 | 0.1338 |
| **1469** | sp|P01763|HV302_HUMAN |  | 1 | 0.87 | 0.045 | 16 | 0.02527 |
| **1470** | tr|B2R4M6|B2R4M6_HUMAN |  | 4 | 1.11 | 0.036 | 16 | 0.03823 |
| **1471** | sp|P49770|EI2BB_HUMAN |  | 3 | 1 | 0.012 | 16 | 0.9596 |
| **1472** | tr|B4DQZ7|B4DQZ7_HUMAN |  | 2 | 0.93 | 0.071 | 16 | 0.3054 |
| **1473** | tr|B3KPC7|B3KPC7_HUMAN |  | 1 | 0.68 | 0.051 | 16 | 4.59E-05 |
| **1474** | tr|B4DPP8|B4DPP8_HUMAN |  | 5 | 0.82 | 0.009 | 16 | 2.23E-06 |
| **1475** | sp|P05090|APOD_HUMAN |  | 3 | 1.12 | 0.02 | 16 | 0.003508 |
| **1476** | tr|A0A024R201|A0A024R201_HUMAN |  | 1 | 0.96 | 0.002 | 16 | 0.002486 |
| **1477** | sp|P08779|K1C16_HUMAN |  | 2 | 1.04 | 0.041 | 16 | 0.4671 |
| **1478** | tr|B7Z550|B7Z550_HUMAN |  | 6 | 1 | 0.003 | 16 | 0.7192 |
| **1479** | tr|B4DEW9|B4DEW9_HUMAN |  | 3 | 1 | 0.036 | 16 | 0.9285 |
| **1480** | sp|P28289|TMOD1_HUMAN |  | 18 | 1 | 0.027 | 16 | 0.9152 |
| **1481** | sp|Q9UI26|IPO11_HUMAN |  | 4 | 0.97 | 0.01 | 16 | 0.2627 |
| **1482** | tr|B7Z832|B7Z832_HUMAN |  | 2 | 0.84 | 0.06 | 16 | 0.02177 |
| **1483** | tr|H7C2X7|H7C2X7_HUMAN |  | 1 | 0.92 | 0.059 | 16 | 0.1865 |
| **1484** | tr|F8WF90|F8WF90_HUMAN |  | 1 | 1.04 | 0.096 | 16 | 0.614 |
| **1485** | tr|B4DUI8|B4DUI8_HUMAN |  | 1 | 1.07 | 0.112 | 16 | 0.448 |
| **1486** | tr|A4D104|A4D104_HUMAN |  | 1 | 1 | 0.009 | 16 | 0.8753 |
| **1487** | tr|F8WDD7|F8WDD7_HUMAN |  | 2 | 1.27 | 0.089 | 16 | 0.002728 |
| **1488** | sp|P04438|HV208_HUMAN |  | 1 | 1.68 | 0.198 | 16 | 2.01E-05 |
| **1489** | tr|Q53FG4|Q53FG4_HUMAN |  | 1 | 1.37 | 0.085 | 16 | 0.0001369 |
| **1490** | tr|B7Z4M5|B7Z4M5_HUMAN |  | 2 | 0.89 | 0.014 | 16 | 0.002777 |
| **1491** | sp|P26641|EF1G_HUMAN |  | 8 | 1.03 | 0.128 | 16 | 0.7106 |
| **1492** | sp|Q12792|TWF1_HUMAN |  | 1 | 0.78 | 0.033 | 16 | 0.0002217 |
| **1493** | tr|A0A024R5S9|A0A024R5S9_HUMAN |  | 3 | 0.99 | 0.009 | 16 | 0.7051 |
| **1494** | sp|P83110|HTRA3_HUMAN |  | 1 | 0.89 | 0.031 | 16 | 0.02001 |
| **1495** | sp|P14780|MMP9_HUMAN |  | 2 | 1.87 | 0.146 | 16 | 1.61E-07 |
| **1496** | sp|Q13200|PSMD2_HUMAN |  | 30 | 0.94 | 0.005 | 16 | 0.003614 |
| **1497** | tr|B2RDJ6|B2RDJ6_HUMAN |  | 5 | 0.93 | 0.008 | 16 | 0.007098 |
| **1498** | tr|H0YJ98|H0YJ98_HUMAN |  | 1 | n/a | 0.008 | 0 | - |
| **1499** | tr|Q6MZL2|Q6MZL2_HUMAN |  | 3 | 1.01 | 0.008 | 16 | 0.8044 |
| **1500** | sp|P57737|CORO7_HUMAN |  | 1 | 1.12 | 0.012 | 16 | 0.0006474 |
| **1501** | sp|Q8WVL7|ANR49_HUMAN |  | 3 | 1.04 | 0.004 | 16 | 0.04675 |
| **1502** | tr|D3DQT9|D3DQT9_HUMAN |  | 1 | 0.85 | 0.01 | 16 | 2.60E-05 |
| **1503** | tr|C9JRD2|C9JRD2_HUMAN |  | 8 | 1 | 0.003 | 16 | 0.9578 |
| **1504** | sp|P29144|TPP2_HUMAN |  | 20 | 1.21 | 0.033 | 16 | 0.0003016 |
| **1505** | sp|P01605|KV113_HUMAN |  | 1 | 1.21 | 0.075 | 16 | 0.006895 |
| **1506** | sp|Q96T51|RUFY1_HUMAN |  | 1 | 0.84 | 0.01 | 16 | 1.21E-05 |
| **1507** | sp|Q58FF8|H90B2_HUMAN |  | 1 | 1.08 | 0.156 | 16 | 0.4052 |
| **1508** | sp|Q09161|NCBP1_HUMAN |  | 6 | 0.92 | 0.011 | 16 | 0.005507 |
| **1509** | tr|Q5T4U8|Q5T4U8_HUMAN |  | 3 | 0.89 | 0.005 | 16 | 2.30E-05 |
| **1510** | tr|B2R8U8|B2R8U8_HUMAN |  | 1 | 1.23 | 0.233 | 16 | 0.0742 |
| **1511** | tr|H3BQD4|H3BQD4_HUMAN |  | 1 | 1.46 | 0.106 | 16 | 4.97E-05 |
| **1512** | sp|P01701|LV103_HUMAN |  | 1 | 0.99 | 0.02 | 16 | 0.8571 |
| **1513** | sp|Q13228|SBP1_HUMAN |  | 12 | 0.89 | 0.004 | 16 | 3.62E-06 |
| **1514** | sp|Q02790|FKBP4_HUMAN |  | 1 | 1.08 | 0.056 | 16 | 0.221 |
| **1515** | tr|H0YBV2|H0YBV2_HUMAN |  | 1 | 0.84 | 0.086 | 16 | 0.04523 |
| **1516** | tr|A2KBC6|A2KBC6_HUMAN |  | 2 | 0.92 | 0.006 | 16 | 0.000376 |
| **1517** | sp|Q92835|SHIP1_HUMAN |  | 4 | 1 | 0.044 | 16 | 0.9347 |
| **1518** | sp|Q9BQA1|MEP50_HUMAN |  | 6 | 0.95 | 0.004 | 16 | 0.01302 |
| **1519** | tr|A0A068LN17|A0A068LN17_HUMAN |  | 1 | 1.3 | 0.416 | 16 | 0.08057 |
| **1520** | sp|Q9Y3C8|UFC1_HUMAN |  | 1 | 1.03 | 0.03 | 16 | 0.4396 |
| **1521** | sp|P35998|PRS7_HUMAN |  | 27 | 0.95 | 0.003 | 16 | 0.005305 |
| **1522** | sp|Q9NQP4|PFD4_HUMAN |  | 5 | 0.94 | 0.007 | 16 | 0.009576 |
| **1523** | tr|Q4TZM4|Q4TZM4_HUMAN |  | 1 | 0.93 | 0.042 | 16 | 0.2083 |
| **1524** | sp|Q9HAH1|ZN556_HUMAN |  | 1 | 0.98 | 0.063 | 16 | 0.7787 |
| **1525** | tr|Q5SRP5|Q5SRP5_HUMAN |  | 1 | 0.96 | 0.006 | 16 | 0.05036 |
| **1526** | tr|K7ES92|K7ES92_HUMAN |  | 1 | 1.15 | 0.035 | 16 | 0.006864 |
| **1527** | sp|Q9NPB8|GPCP1_HUMAN |  | 6 | 1.09 | 0.037 | 16 | 0.09054 |
| **1528** | tr|E5RHG8|E5RHG8_HUMAN |  | 3 | 1.07 | 0.012 | 16 | 0.02264 |
| **1529** | sp|Q96SE0|ABHD1_HUMAN |  | 1 | 0.93 | 0.048 | 16 | 0.2005 |
| **1530** | tr|Q762B6|Q762B6_HUMAN |  | 1 | 1.34 | 0.097 | 16 | 0.000539 |
| **1531** | tr|A0A024R223|A0A024R223_HUMAN |  | 10 | 0.95 | 0.006 | 16 | 0.02027 |
| **1532** | tr|Q53HB3|Q53HB3_HUMAN |  | 21 | 0.94 | 0.005 | 16 | 0.004351 |
| **1533** | sp|P01780|HV319_HUMAN |  | 1 | 0.88 | 0.073 | 16 | 0.1003 |
| **1534** | sp|Q8TCG1|CIP2A_HUMAN |  | 1 | 1.31 | 0.818 | 16 | 0.193 |
| **1535** | tr|B4DP93|B4DP93_HUMAN |  | 1 | 1.13 | 0.014 | 16 | 0.0005686 |
| **1536** | tr|B7Z7V7|B7Z7V7_HUMAN |  | 1 | 0.75 | 0.054 | 16 | 0.0006929 |
| **1537** | tr|Q2XP30|Q2XP30_HUMAN |  | 1 | 1.08 | 0.129 | 16 | 0.3778 |
| **1538** | tr|H0YDY4|H0YDY4_HUMAN |  | 1 | 1.07 | 0.028 | 16 | 0.1149 |
| **1539** | sp|P12273|PIP_HUMAN |  | 1 | 1.02 | 0.191 | 16 | 0.8708 |
| **1540** | tr|B3KPF2|B3KPF2_HUMAN |  | 1 | 1.5 | 0.436 | 16 | 0.008923 |
| **1541** | tr|Q9UL93|Q9UL93_HUMAN |  | 1 | 0.89 | 0.018 | 16 | 0.005187 |
| **1542** | tr|B2R514|B2R514_HUMAN |  | 9 | 1.03 | 0.002 | 16 | 0.01885 |
| **1543** | sp|P02533|K1C14_HUMAN |  | 2 | 0.92 | 0.126 | 16 | 0.3927 |
| **1544** | tr|F8W1R7|F8W1R7_HUMAN |  | 6 | 1.19 | 0.01 | 16 | 2.59E-06 |
| **1545** | tr|Q59FS8|Q59FS8_HUMAN |  | 1 | 0.98 | 0.1 | 16 | 0.818 |
| **1546** | sp|Q562E7|WDR81_HUMAN |  | 15 | 0.88 | 0.004 | 16 | 3.33E-06 |
| **1547** | tr|R4GMS4|R4GMS4_HUMAN |  | 1 | 1.22 | 0.197 | 16 | 0.06751 |
| **1548** | tr|U3KPS2|U3KPS2_HUMAN |  | 2 | 2.01 | 0.143 | 16 | 1.98E-08 |
| **1549** | tr|Q59ES1|Q59ES1_HUMAN |  | 4 | 1.03 | 0.006 | 16 | 0.1303 |
| **1550** | tr|H7BZM7|H7BZM7_HUMAN |  | 1 | 0.8 | 0.046 | 16 | 0.002155 |
| **1551** | sp|O75157|T22D2_HUMAN |  | 1 | 1.01 | 0.094 | 16 | 0.9178 |
| **1552** | tr|H7C548|H7C548_HUMAN |  | 1 | 0.73 | 0.002 | 8 | 1.25E-06 |
| **1553** | sp|P04180|LCAT_HUMAN |  | 8 | 1 | 0.005 | 16 | 0.9831 |
| **1554** | tr|B2RAY1|B2RAY1_HUMAN |  | 8 | 1.04 | 0.004 | 16 | 0.03882 |
| **1555** | tr|E9PJP1|E9PJP1_HUMAN |  | 1 | 1.27 | 0.059 | 16 | 0.0004009 |
| **1556** | tr|B4DV64|B4DV64_HUMAN |  | 1 | 1.54 | 0.077 | 16 | 1.07E-06 |
| **1557** | tr|Q548W1|Q548W1_HUMAN |  | 1 | 1.05 | 0.159 | 14 | 0.6587 |
| **1558** | sp|P48426|PI42A_HUMAN |  | 7 | 1.21 | 0.008 | 16 | 6.73E-08 |
| **1559** | tr|C9JN83|C9JN83_HUMAN |  | 1 | 1.13 | 0.025 | 16 | 0.004213 |
| **1560** | sp|Q14126|DSG2_HUMAN |  | 4 | 1.05 | 0.017 | 16 | 0.1353 |
| **1561** | sp|Q93079|H2B1H_HUMAN |  | 2 | 4.31 | 0.232 | 16 | 3.00E-14 |
| **1562** | tr|B0AZS5|B0AZS5_HUMAN |  | 2 | 1.02 | 0.01 | 16 | 0.4855 |
| **1563** | tr|G3V5B9|G3V5B9_HUMAN |  | 2 | 0.93 | 0.156 | 16 | 0.4633 |
| **1564** | tr|E9PGG1|E9PGG1_HUMAN |  | 2 | 1.14 | 0.009 | 16 | 2.73E-05 |
| **1565** | tr|H0YE34|H0YE34_HUMAN |  | 1 | 0.89 | 0.068 | 16 | 0.1095 |
| **1566** | tr|Q53ET9|Q53ET9_HUMAN |  | 7 | 0.94 | 0.006 | 16 | 0.006352 |
| **1567** | sp|Q9H8W4|PKHF2_HUMAN |  | 4 | 0.9 | 0.004 | 16 | 2.08E-05 |
| **1568** | sp|Q01970|PLCB3_HUMAN |  | 2 | 1.03 | 0.063 | 16 | 0.6047 |
| **1569** | tr|B8ZZZ0|B8ZZZ0_HUMAN |  | 1 | 1 | 0.085 | 16 | 0.9973 |
| **1570** | tr|Q53H56|Q53H56_HUMAN |  | 5 | 0.96 | 0.005 | 16 | 0.03938 |
| **1571** | tr|Q6MZU6|Q6MZU6_HUMAN |  | 3 | 0.98 | 0.03 | 16 | 0.6931 |
| **1572** | tr|E9PGT1|E9PGT1_HUMAN |  | 3 | 1.12 | 0.007 | 16 | 3.91E-05 |
| **1573** | tr|B4E366|B4E366_HUMAN |  | 2 | 1.24 | 0.03 | 16 | 5.13E-05 |
| **1574** | tr|S6B294|S6B294_HUMAN |  | 1 | 0.91 | 0.037 | 16 | 0.06607 |
| **1575** | tr|E7EQ14|E7EQ14_HUMAN |  | 1 | 0.94 | 0.036 | 16 | 0.2307 |
| **1576** | tr|O15302|O15302_HUMAN |  | 1 | 1.3 | 0.161 | 16 | 0.009293 |
| **1577** | tr|A0A087WTP2|A0A087WTP2_HUMAN |  | 1 | 1.19 | 0.014 | 16 | 1.42E-05 |
| **1578** | tr|M0R261|M0R261_HUMAN |  | 2 | 0.83 | 0.02 | 16 | 0.0001983 |
| **1579** | tr|Q9BU08|Q9BU08_HUMAN |  | 24 | 0.92 | 0.002 | 16 | 4.54E-06 |
| **1580** | tr|Q6GMX0|Q6GMX0_HUMAN |  | 1 | 0.92 | 0.043 | 16 | 0.1485 |
| **1581** | tr|A0A024QZ63|A0A024QZ63_HUMAN |  | 15 | 0.93 | 0.01 | 16 | 0.01221 |
| **1582** | tr|A0A024RC24|A0A024RC24_HUMAN |  | 4 | 0.98 | 0.008 | 16 | 0.2998 |
| **1583** | tr|A0A087WXT8|A0A087WXT8_HUMAN |  | 1 | 1.01 | 0.041 | 16 | 0.9168 |
| **1584** | tr|I3L0V5|I3L0V5_HUMAN |  | 1 | 1.22 | 0.038 | 16 | 0.0005106 |
| **1585** | tr|U6A3P2|U6A3P2_HUMAN |  | 1 | 0.94 | 0.06 | 16 | 0.3722 |
| **1586** | tr|H0YNE9|H0YNE9_HUMAN |  | 1 | 0.63 | 0.027 | 16 | 1.67E-07 |
| **1587** | sp|Q92890|UFD1_HUMAN |  | 1 | 0.86 | 0.017 | 16 | 0.0004785 |
| **1588** | tr|B2R9F2|B2R9F2_HUMAN |  | 1 | 1.14 | 0.066 | 16 | 0.04381 |
| **1589** | tr|J3KRN5|J3KRN5_HUMAN |  | 1 | 0.96 | 0.03 | 16 | 0.3684 |
| **1590** | tr|B2R944|B2R944_HUMAN |  | 1 | 0.76 | 0.052 | 16 | 0.0007453 |
| **1591** | sp|P10909|CLUS_HUMAN |  | 13 | 1.01 | 0.002 | 16 | 0.2055 |
| **1592** | tr|V9HW13|V9HW13_HUMAN |  | 3 | 1.06 | 0.02 | 16 | 0.1016 |
| **1593** | sp|Q9UJC5|SH3L2_HUMAN |  | 1 | 0.9 | 0.068 | 16 | 0.164 |
| **1594** | tr|G3V0G3|G3V0G3_HUMAN |  | 1 | 0.92 | 0.066 | 16 | 0.2403 |
| **1595** | tr|H7BXH2|H7BXH2_HUMAN |  | 15 | 0.92 | 0.006 | 16 | 0.000832 |
| **1596** | sp|Q9Y608|LRRF2_HUMAN |  | 2 | 1 | 0.052 | 16 | 0.9931 |
| **1597** | sp|P17858|PFKAL_HUMAN |  | 15 | 0.99 | 0.003 | 16 | 0.3155 |
| **1598** | sp|O75663|TIPRL_HUMAN |  | 1 | 1.05 | 0.004 | 16 | 0.007033 |
| **1599** | sp|P52597|HNRPF_HUMAN |  | 4 | 1.01 | 0.021 | 16 | 0.7548 |
| **1600** | tr|H7C488|H7C488_HUMAN |  | 2 | 0.91 | 0.022 | 16 | 0.02482 |
| **1601** | sp|P61960|UFM1_HUMAN |  | 1 | 1.12 | 0.08 | 16 | 0.1148 |
| **1602** | tr|H0YBP2|H0YBP2_HUMAN |  | 1 | 1.66 | 1.02 | 7 | 0.1343 |
| **1603** | sp|P21281|VATB2_HUMAN |  | 11 | 1.08 | 0.005 | 16 | 0.000292 |
| **1604** | sp|Q9H444|CHM4B_HUMAN |  | 7 | 1 | 0.003 | 16 | 0.9704 |
| **1605** | tr|Q8N2H2|Q8N2H2_HUMAN |  | 1 | 1.15 | 0.242 | 16 | 0.2444 |
| **1606** | sp|Q9NRW1|RAB6B_HUMAN |  | 2 | 0.99 | 0.012 | 16 | 0.7668 |
| **1607** | sp|P46736|BRCC3_HUMAN |  | 1 | 1.03 | 0.008 | 16 | 0.2467 |
| **1608** | tr|H7C3K3|H7C3K3_HUMAN |  | 2 | 1.09 | 0.012 | 16 | 0.005346 |
| **1609** | sp|P40197|GPV_HUMAN |  | 3 | 0.88 | 0.027 | 16 | 0.01233 |
| **1610** | sp|Q14315|FLNC_HUMAN |  | 1 | 0.88 | 0.028 | 16 | 0.01203 |
| **1611** | sp|Q9UIM3|FKBPL_HUMAN |  | 1 | 1.16 | 0.029 | 16 | 0.001896 |
| **1612** | sp|Q9C0I1|MTMRC_HUMAN |  | 1 | 0.99 | 0.012 | 16 | 0.7026 |
| **1613** | tr|A0A024QYX7|A0A024QYX7_HUMAN |  | 3 | 1.02 | 0.033 | 16 | 0.6657 |
| **1614** | sp|Q13164|MK07_HUMAN |  | 3 | 1.03 | 0.074 | 16 | 0.6245 |
| **1615** | tr|A2J1N5|A2J1N5_HUMAN |  | 1 | 1.01 | 0.035 | 16 | 0.8379 |
| **1616** | tr|D3DQX7|D3DQX7_HUMAN |  | 1 | 1.02 | 0.044 | 16 | 0.7284 |
| **1617** | sp|P07333|CSF1R_HUMAN |  | 1 | 1.08 | 0.039 | 16 | 0.1166 |
| **1618** | sp|Q9NSK0|KLC4_HUMAN |  | 2 | 0.92 | 0.002 | 16 | 2.37E-06 |
| **1619** | sp|P58401|NRX2B_HUMAN |  | 1 | 0.98 | 0.039 | 16 | 0.7422 |
| **1620** | tr|E9PCY7|E9PCY7_HUMAN |  | 1 | 0.88 | 0.003 | 16 | 6.09E-07 |
| **1621** | tr|K7ER15|K7ER15_HUMAN |  | 2 | 0.95 | 0.007 | 16 | 0.04244 |
| **1622** | tr|Q5TD07|Q5TD07_HUMAN |  | 2 | 0.83 | 0.061 | 16 | 0.0128 |
| **1623** | tr|F5H157|F5H157_HUMAN |  | 1 | 1.04 | 0.009 | 16 | 0.09652 |
| **1624** | tr|Q6ZP85|Q6ZP85_HUMAN |  | 1 | 1.01 | 0.066 | 16 | 0.8563 |
| **1625** | tr|I3L1K6|I3L1K6_HUMAN |  | 8 | 0.9 | 0.009 | 16 | 0.001071 |
| **1626** | tr|V9HW35|V9HW35_HUMAN |  | 3 | 0.83 | 0.009 | 16 | 3.27E-06 |
| **1627** | sp|P27169|PON1_HUMAN |  | 11 | 0.99 | 0.008 | 16 | 0.7758 |
| **1628** | tr|Q7Z2K5|Q7Z2K5_HUMAN |  | 1 | 0.87 | 0.054 | 16 | 0.04309 |
| **1629** | sp|P01598|KV106_HUMAN |  | 2 | 0.9 | 0.036 | 16 | 0.05915 |
| **1630** | sp|A8K010|CF176_HUMAN |  | 1 | 1.1 | 0.066 | 7 | 0.3518 |
| **1631** | tr|A0A024R094|A0A024R094_HUMAN |  | 2 | 1.06 | 0.03 | 16 | 0.1573 |
| **1632** | sp|P01011|AACT_HUMAN |  | 9 | 1.57 | 0.033 | 16 | 2.20E-09 |
| **1633** | sp|Q15555|MARE2_HUMAN |  | 2 | 1.16 | 0.112 | 16 | 0.08011 |
| **1634** | tr|C9JPH9|C9JPH9_HUMAN |  | 1 | 0.66 | 0.076 | 16 | 0.0001784 |
| **1635** | tr|O43705|O43705_HUMAN |  | 1 | 0.95 | 0.082 | 16 | 0.5155 |
| **1636** | sp|Q16864|VATF_HUMAN |  | 2 | 1 | 0.03 | 16 | 0.9098 |
| **1637** | sp|Q9Y265|RUVB1_HUMAN |  | 16 | 0.96 | 0.014 | 16 | 0.1726 |
| **1638** | sp|P62269|RS18_HUMAN |  | 1 | 0.98 | 0.028 | 16 | 0.6143 |
| **1639** | sp|P00734|THRB_HUMAN |  | 30 | 0.89 | 0.006 | 16 | 3.91E-05 |
| **1640** | tr|H7C576|H7C576_HUMAN |  | 1 | 0.98 | 0.038 | 16 | 0.7019 |
| **1641** | tr|B3KS44|B3KS44_HUMAN |  | 1 | 1.07 | 0.035 | 16 | 0.1821 |
| **1642** | tr|Q59GZ2|Q59GZ2_HUMAN |  | 3 | 1.12 | 0.017 | 16 | 0.001608 |
| **1643** | tr|A0A024R0G8|A0A024R0G8_HUMAN |  | 2 | 0.96 | 0.038 | 16 | 0.475 |
| **1644** | tr|B3KSJ4|B3KSJ4_HUMAN |  | 1 | 1.52 | 0.072 | 16 | 1.44E-06 |
| **1645** | tr|S6BGD4|S6BGD4_HUMAN |  | 1 | 1.37 | 0.086 | 16 | 0.0001422 |
| **1646** | sp|P01880|IGHD_HUMAN |  | 2 | 1.25 | 0.045 | 16 | 0.0003251 |
| **1647** | tr|H7BZ93|H7BZ93_HUMAN |  | 1 | 0.46 | 0.087 | 16 | 2.64E-06 |
| **1648** | sp|Q712K3|UB2R2_HUMAN |  | 1 | 1.37 | 0.242 | 16 | 0.008864 |
| **1649** | sp|Q9H8S9|MOB1A_HUMAN |  | 4 | 1.03 | 0.028 | 16 | 0.5528 |
| **1650** | sp|Q15691|MARE1_HUMAN |  | 9 | 0.99 | 0.006 | 16 | 0.6698 |
| **1651** | sp|P01762|HV301_HUMAN |  | 3 | 0.88 | 0.018 | 16 | 0.001886 |
| **1652** | tr|H7C123|H7C123_HUMAN |  | 1 | 0.96 | 0.036 | 16 | 0.3686 |
| **1653** | tr|A8K9T9|A8K9T9_HUMAN |  | 28 | 0.94 | 0.064 | 16 | 0.3255 |
| **1654** | tr|B4E3S6|B4E3S6_HUMAN |  | 2 | 0.97 | 0.023 | 16 | 0.4634 |
| **1655** | sp|Q9Y5K6|CD2AP_HUMAN |  | 1 | 1.08 | 0.028 | 16 | 0.08702 |
| **1656** | tr|B3KVA9|B3KVA9_HUMAN |  | 1 | 1.15 | 0.055 | 16 | 0.02201 |
| **1657** | tr|Q8N5Y3|Q8N5Y3_HUMAN |  | 3 | 1.04 | 0.031 | 16 | 0.4155 |
| **1658** | tr|B4DI15|B4DI15_HUMAN |  | 3 | 1.2 | 0.032 | 16 | 0.0004175 |
| **1659** | sp|P31146|COR1A_HUMAN |  | 1 | 1.08 | 0.007 | 16 | 0.001439 |
| **1660** | tr|D3DP75|D3DP75_HUMAN |  | 7 | 1 | 0.009 | 16 | 0.8736 |
| **1661** | tr|H0YC44|H0YC44_HUMAN |  | 1 | 0.83 | 0.073 | 16 | 0.0253 |
| **1662** | tr|A0A024R8B1|A0A024R8B1_HUMAN |  | 2 | 1.14 | 0.113 | 16 | 0.1107 |
| **1663** | sp|Q8NEX9|DR9C7_HUMAN |  | 1 | 1.19 | 1.53 | 16 | 0.5461 |
| **1664** | tr|B4DSH1|B4DSH1_HUMAN |  | 1 | 0.95 | 0.084 | 16 | 0.5321 |
| **1665** | sp|P35542|SAA4_HUMAN |  | 1 | 1.24 | 0.058 | 16 | 0.001118 |
| **1666** | tr|J3QLD1|J3QLD1_HUMAN |  | 1 | 0.83 | 0.016 | 16 | 6.45E-05 |
| **1667** | sp|Q9NZZ3|CHMP5_HUMAN |  | 2 | 0.77 | 0.008 | 16 | 5.66E-08 |
| **1668** | tr|D3DS15|D3DS15_HUMAN |  | 1 | 0.91 | 0.048 | 16 | 0.1087 |
| **1669** | tr|Q5NV62|Q5NV62_HUMAN |  | 1 | 0.85 | 0.15 | 16 | 0.1348 |
| **1670** | tr|Q6AI18|Q6AI18_HUMAN |  | 4 | 0.85 | 0.012 | 16 | 7.96E-05 |
| **1671** | sp|Q96KB5|TOPK_HUMAN |  | 1 | 0.94 | 0.024 | 16 | 0.1398 |
| **1672** | tr|Q96IR1|Q96IR1_HUMAN |  | 4 | 0.96 | 0.003 | 16 | 0.01386 |
| **1673** | tr|Q75N88|Q75N88_HUMAN |  | 1 | 1.14 | 0.052 | 16 | 0.02506 |
| **1674** | tr|H0YEP5|H0YEP5_HUMAN |  | 1 | 1.05 | 0.016 | 16 | 0.1364 |
| **1675** | tr|H7BZN7|H7BZN7_HUMAN |  | 1 | 0.93 | 0.16 | 16 | 0.4854 |
| **1676** | tr|Q86UW0|Q86UW0_HUMAN |  | 1 | 1.8 | 0.174 | 16 | 1.49E-06 |
| **1677** | sp|P09496|CLCA_HUMAN |  | 5 | 0.92 | 0.013 | 16 | 0.01989 |
| **1678** | tr|Q5JR08|Q5JR08_HUMAN |  | 3 | 1.08 | 0.006 | 16 | 0.0007827 |
| **1679** | sp|P22314|UBA1_HUMAN |  | 4 | 1.1 | 0.007 | 16 | 0.0002634 |
| **1680** | tr|B4DGW3|B4DGW3_HUMAN |  | 4 | 1.21 | 0.008 | 16 | 1.30E-07 |
| **1681** | sp|Q6B0K9|HBM_HUMAN |  | 4 | 0.96 | 0.006 | 16 | 0.07089 |
| **1682** | tr|A8KAK1|A8KAK1_HUMAN |  | 2 | 1.16 | 0.046 | 16 | 0.0101 |
| **1683** | tr|E9PHZ2|E9PHZ2_HUMAN |  | 1 | 0.88 | 0.042 | 16 | 0.02894 |
| **1684** | sp|Q2KHT3|CL16A_HUMAN |  | 1 | 1.07 | 0.02 | 16 | 0.06813 |
| **1685** | tr|B4E326|B4E326_HUMAN |  | 2 | 0.86 | 0.026 | 16 | 0.003498 |
| **1686** | tr|B4DDL6|B4DDL6_HUMAN |  | 1 | 1.18 | 0.016 | 16 | 4.89E-05 |
| **1687** | tr|H3BQA7|H3BQA7_HUMAN |  | 1 | 0.86 | 0.018 | 16 | 0.0007601 |
| **1688** | sp|P23193|TCEA1_HUMAN |  | 1 | 0.85 | 0.017 | 16 | 0.0003183 |
| **1689** | sp|P10155|RO60_HUMAN |  | 5 | 0.83 | 0.012 | 16 | 1.64E-05 |
| **1690** | tr|B3KNA1|B3KNA1_HUMAN |  | 3 | 0.94 | 0.014 | 16 | 0.07052 |
| **1691** | sp|O95433|AHSA1_HUMAN |  | 6 | 1.02 | 0.036 | 16 | 0.6857 |
| **1692** | tr|C9JJ54|C9JJ54_HUMAN |  | 3 | 0.9 | 0.033 | 16 | 0.04504 |
| **1693** | tr|C9JXX4|C9JXX4_HUMAN |  | 1 | 0.94 | 0.034 | 16 | 0.2432 |
| **1694** | sp|Q9Y371|SHLB1_HUMAN |  | 5 | 0.98 | 0.013 | 16 | 0.5222 |
| **1695** | tr|F5GWF8|F5GWF8_HUMAN |  | 1 | 1.12 | 0.465 | 12 | 0.5416 |
| **1696** | tr|C9JFE4|C9JFE4_HUMAN |  | 1 | 0.93 | 0.013 | 16 | 0.02523 |
| **1697** | sp|P06733|ENOA_HUMAN |  | 14 | 0.93 | 0.058 | 16 | 0.2886 |
| **1698** | sp|P02656|APOC3_HUMAN |  | 6 | 0.99 | 0.01 | 16 | 0.7333 |
| **1699** | tr|B3KVN0|B3KVN0_HUMAN |  | 7 | 0.55 | 0.025 | 16 | 7.64E-09 |
| **1700** | sp|Q01432|AMPD3_HUMAN |  | 1 | 1.28 | 0.011 | 16 | 1.82E-08 |
| **1701** | sp|O00429|DNM1L_HUMAN |  | 6 | 1.01 | 0.01 | 16 | 0.6411 |
| **1702** | tr|B4DG60|B4DG60_HUMAN |  | 4 | 1 | 0.012 | 16 | 0.9507 |
| **1703** | tr|A0A024R872|A0A024R872_HUMAN |  | 2 | 1.02 | 0.032 | 16 | 0.7343 |
| **1704** | tr|A0A068LKQ2|A0A068LKQ2_HUMAN |  | 1 | 0.78 | 0.048 | 16 | 0.001062 |
| **1705** | tr|Q1JQ76|Q1JQ76_HUMAN |  | 2 | 0.96 | 0.032 | 16 | 0.3434 |
| **1706** | sp|P07360|CO8G_HUMAN |  | 4 | 0.93 | 0.01 | 16 | 0.01784 |
| **1707** | sp|P10644|KAP0_HUMAN |  | 5 | 1.03 | 0.008 | 16 | 0.1376 |
| **1708** | tr|B7Z410|B7Z410_HUMAN |  | 1 | 1.17 | 0.058 | 16 | 0.01149 |
| **1709** | tr|K7EMA7|K7EMA7_HUMAN |  | 1 | 1.03 | 0.007 | 16 | 0.2199 |
| **1710** | tr|Q53FT8|Q53FT8_HUMAN |  | 10 | 1.04 | 0.001 | 16 | 0.0001091 |
| **1711** | tr|E9PGT6|E9PGT6_HUMAN |  | 5 | 0.81 | 0.021 | 16 | 0.0001103 |
| **1712** | sp|Q14644|RASA3_HUMAN |  | 1 | 1.53 | 0.086 | 16 | 2.85E-06 |
| **1713** | tr|H0YHP2|H0YHP2_HUMAN |  | 1 | 0.99 | 0.197 | 16 | 0.9065 |
| **1714** | tr|B7Z5Q2|B7Z5Q2_HUMAN |  | 22 | 1.69 | 0.035 | 16 | 2.34E-10 |
| **1715** | sp|Q04756|HGFA_HUMAN |  | 3 | 1.1 | 0.019 | 16 | 0.01302 |
| **1716** | tr|B3KUR3|B3KUR3_HUMAN |  | 2 | 0.93 | 0.013 | 16 | 0.02801 |
| **1717** | tr|B4DH46|B4DH46_HUMAN |  | 1 | 1.02 | 0.038 | 16 | 0.7445 |
| **1718** | tr|Q71US4|Q71US4_HUMAN |  | 1 | 0.94 | 0.029 | 16 | 0.1878 |
| **1719** | tr|B4E386|B4E386_HUMAN |  | 1 | 1.33 | 0.686 | 16 | 0.1339 |
| **1720** | tr|Q9UL86|Q9UL86_HUMAN |  | 1 | 1.13 | 0.164 | 16 | 0.2114 |
| **1721** | tr|B7Z5A7|B7Z5A7_HUMAN |  | 2 | 1.01 | 0.041 | 16 | 0.8211 |
| **1722** | tr|R4GMR9|R4GMR9_HUMAN |  | 1 | 1.06 | 0.103 | 12 | 0.5564 |
| **1723** | tr|F8VUA7|F8VUA7_HUMAN |  | 1 | 0.9 | 0.02 | 16 | 0.01508 |
| **1724** | tr|E9PBM9|E9PBM9_HUMAN |  | 1 | 0.75 | 0.106 | 16 | 0.007508 |
| **1725** | tr|A0A087WYT5|A0A087WYT5_HUMAN |  | 1 | 0.91 | 0.127 | 16 | 0.3421 |
| **1726** | tr|B4DP50|B4DP50_HUMAN |  | 1 | 1.12 | 0.049 | 16 | 0.05185 |
| **1727** | tr|C9JGI3|C9JGI3_HUMAN |  | 1 | 1.1 | 0.12 | 16 | 0.2858 |
| **1728** | sp|Q9BPX3|CND3_HUMAN |  | 3 | 0.97 | 0.013 | 16 | 0.393 |
| **1729** | sp|Q9HCL0|PCD18_HUMAN |  | 1 | 0.94 | 0.057 | 16 | 0.2986 |
| **1730** | tr|E7EX90|E7EX90_HUMAN |  | 1 | 0.91 | 0.006 | 16 | 0.0002601 |
| **1731** | sp|P61019|RAB2A_HUMAN |  | 1 | 1.16 | 0.011 | 16 | 2.48E-05 |
| **1732** | tr|B1AK87|B1AK87_HUMAN |  | 6 | 1.08 | 0.01 | 16 | 0.005045 |
| **1733** | tr|A0A087X0K1|A0A087X0K1_HUMAN |  | 2 | 1.01 | 0.022 | 16 | 0.8199 |
| **1734** | sp|P62826|RAN_HUMAN |  | 9 | 0.98 | 0.024 | 16 | 0.6198 |
| **1735** | sp|O00743|PPP6_HUMAN |  | 10 | 0.99 | 0.001 | 16 | 0.1871 |
| **1736** | tr|Q14730|Q14730_HUMAN |  | 1 | 1.35 | 0.063 | 16 | 4.98E-05 |
| **1737** | tr|A2IPI6|A2IPI6_HUMAN |  | 1 | 1.27 | 0.382 | 16 | 0.09941 |
| **1738** | tr|A2A283|A2A283_HUMAN |  | 1 | 1.03 | 0.072 | 16 | 0.6201 |
| **1739** | sp|Q9NTX5|ECHD1_HUMAN |  | 6 | 0.89 | 0.02 | 16 | 0.008568 |
| **1740** | tr|Q7RTQ9|Q7RTQ9_HUMAN |  | 5 | 1.1 | 0.016 | 16 | 0.004258 |
| **1741** | tr|A0A087WWB5|A0A087WWB5_HUMAN |  | 1 | 1.04 | 0.086 | 16 | 0.604 |
| **1742** | tr|F8WDW5|F8WDW5_HUMAN |  | 1 | 1.09 | 0.113 | 16 | 0.2793 |
| **1743** | sp|P52790|HXK3_HUMAN |  | 1 | 0.91 | 0.032 | 16 | 0.05798 |
| **1744** | tr|B4DKD6|B4DKD6_HUMAN |  | 1 | 0.92 | 0.032 | 16 | 0.112 |
| **1745** | tr|E5RK61|E5RK61_HUMAN |  | 1 | 0.97 | 0.012 | 16 | 0.2564 |
| **1746** | tr|F8VS29|F8VS29_HUMAN |  | 1 | 1.21 | 0.042 | 16 | 0.0009425 |
| **1747** | sp|P61086|UBE2K_HUMAN |  | 1 | 0.92 | 0.083 | 16 | 0.2719 |
| **1748** | tr|Q5CAQ4|Q5CAQ4_HUMAN |  | 1 | 0.92 | 0.168 | 16 | 0.4464 |
| **1749** | tr|Q6FI54|Q6FI54_HUMAN |  | 2 | 1.12 | 0.021 | 16 | 0.004277 |
| **1750** | sp|P55010|IF5_HUMAN |  | 6 | 0.93 | 0.002 | 16 | 5.99E-06 |
| **1751** | tr|C9JI91|C9JI91_HUMAN |  | 1 | 0.92 | 0.053 | 16 | 0.1965 |
| **1752** | tr|A0A024QZN4|A0A024QZN4_HUMAN |  | 16 | 1.07 | 0.006 | 16 | 0.003162 |
| **1753** | sp|Q3YEC7|RABL6_HUMAN |  | 1 | 0.99 | 0.043 | 16 | 0.9041 |
| **1754** | tr|D6RAL7|D6RAL7_HUMAN |  | 1 | 0.87 | 0.065 | 16 | 0.06316 |
| **1755** | tr|H3BQF1|H3BQF1_HUMAN |  | 3 | 0.94 | 0.006 | 16 | 0.007453 |
| **1756** | tr|H3BUG4|H3BUG4_HUMAN |  | 2 | 1.02 | 0.01 | 16 | 0.4633 |
| **1757** | sp|Q8TAT6|NPL4_HUMAN |  | 4 | 1.02 | 0.007 | 16 | 0.3124 |
| **1758** | sp|Q8WXI9|P66B_HUMAN |  | 1 | 0.88 | 0.008 | 16 | 0.0001419 |
| **1759** | sp|P49721|PSB2_HUMAN |  | 8 | 0.99 | 0.003 | 16 | 0.4473 |
| **1760** | tr|D6RCQ0|D6RCQ0_HUMAN |  | 1 | 1.17 | 0.038 | 16 | 0.003178 |
| **1761** | tr|A6NJA2|A6NJA2_HUMAN |  | 16 | 0.83 | 0.064 | 16 | 0.01464 |
| **1762** | sp|P35573|GDE_HUMAN |  | 8 | 0.91 | 0.021 | 16 | 0.02154 |
| **1763** | sp|P31946|1433B_HUMAN |  | 4 | 1.17 | 0.017 | 16 | 7.12E-05 |
| **1764** | tr|A0A0A0MSI9|A0A0A0MSI9_HUMAN |  | 1 | 0.95 | 0.086 | 16 | 0.5112 |
| **1765** | sp|Q92973|TNPO1_HUMAN |  | 7 | 0.94 | 0.004 | 16 | 0.001505 |
| **1766** | sp|P05546|HEP2_HUMAN |  | 6 | 0.85 | 0.178 | 16 | 0.1807 |
| **1767** | tr|M0R210|M0R210_HUMAN |  | 3 | 0.96 | 0.029 | 16 | 0.3278 |
| **1768** | tr|Q59GF8|Q59GF8_HUMAN |  | 16 | 1 | 0.005 | 16 | 0.8237 |
| **1769** | tr|B0AZL7|B0AZL7_HUMAN |  | 4 | 1.04 | 0.027 | 16 | 0.3819 |
| **1770** | sp|Q96IJ6|GMPPA_HUMAN |  | 3 | 0.98 | 0.01 | 16 | 0.5501 |
| **1771** | tr|I3L4A2|I3L4A2_HUMAN |  | 5 | 0.93 | 0.006 | 16 | 0.002531 |
| **1772** | sp|Q86TP1|PRUNE_HUMAN |  | 6 | 0.95 | 0.004 | 16 | 0.00552 |
| **1773** | sp|O00602|FCN1_HUMAN |  | 1 | 1.22 | 0.041 | 16 | 0.0006718 |
| **1774** | tr|B3KX11|B3KX11_HUMAN |  | 3 | 0.92 | 0.004 | 16 | 0.0001281 |
| **1775** | tr|Q0VGA5|Q0VGA5_HUMAN |  | 1 | 0.82 | 0.015 | 16 | 2.91E-05 |
| **1776** | sp|Q70J99|UN13D_HUMAN |  | 3 | 0.86 | 0.011 | 16 | 0.0001026 |
| **1777** | tr|A0A0A0MTP6|A0A0A0MTP6_HUMAN |  | 1 | 0.95 | 0.058 | 16 | 0.3991 |
| **1778** | tr|Q8NAH3|Q8NAH3_HUMAN |  | 3 | 0.97 | 0.042 | 16 | 0.525 |
| **1779** | tr|Q8N3Y0|Q8N3Y0_HUMAN |  | 1 | 0.92 | 0.053 | 16 | 0.1753 |
| **1780** | sp|P08493|MGP_HUMAN |  | 1 | 0.97 | 0.049 | 16 | 0.5888 |
| **1781** | tr|A0A024RB10|A0A024RB10_HUMAN |  | 3 | 0.8 | 0.038 | 16 | 0.00112 |
| **1782** | tr|A8KAJ3|A8KAJ3_HUMAN |  | 5 | 0.97 | 0.025 | 16 | 0.4944 |
| **1783** | tr|A0A075B7G8|A0A075B7G8_HUMAN |  | 1 | 0.74 | 0.07 | 16 | 0.001318 |
| **1784** | tr|D6RBY0|D6RBY0_HUMAN |  | 1 | 0.96 | 0.108 | 16 | 0.6187 |
| **1785** | tr|Q5R207|Q5R207_HUMAN |  | 1 | 1.05 | 0.044 | 16 | 0.3721 |
| **1786** | tr|H0Y786|H0Y786_HUMAN |  | 1 | 1.37 | 0.093 | 16 | 0.0002004 |
| **1787** | tr|A2MYE2|A2MYE2_HUMAN |  | 1 | 0.78 | 0.024 | 16 | 4.34E-05 |
| **1788** | tr|Q5QPQ1|Q5QPQ1_HUMAN |  | 2 | 0.81 | 0.031 | 16 | 0.0006273 |
| **1789** | sp|P05387|RLA2_HUMAN |  | 5 | 0.89 | 0.045 | 16 | 0.06495 |
| **1790** | sp|Q9UI10|EI2BD_HUMAN |  | 2 | 0.92 | 0.017 | 16 | 0.02996 |
| **1791** | sp|Q07507|DERM_HUMAN |  | 2 | 0.93 | 0.015 | 16 | 0.03123 |
| **1792** | tr|H0YMM0|H0YMM0_HUMAN |  | 1 | 1.01 | 0.961 | 16 | 0.9758 |
| **1793** | sp|Q9UHP3|UBP25_HUMAN |  | 2 | 0.98 | 0.01 | 16 | 0.5395 |
| **1794** | tr|E9PKF8|E9PKF8_HUMAN |  | 1 | 1.09 | 0.088 | 16 | 0.239 |
| **1795** | sp|Q9BZE9|ASPC1_HUMAN |  | 9 | 0.87 | 0.009 | 16 | 5.54E-05 |
| **1796** | tr|B3KY56|B3KY56_HUMAN |  | 1 | 0.92 | 0.132 | 16 | 0.3889 |
| **1797** | tr|B5MCT7|B5MCT7_HUMAN |  | 4 | 1.03 | 0.009 | 16 | 0.2999 |
| **1798** | tr|C3W5P5|C3W5P5_HUMAN |  | 1 | 0.74 | 0.003 | 4 | 0.002435 |
| **1799** | tr|Q6PH87|Q6PH87_HUMAN |  | 1 | 1.04 | 0.055 | 16 | 0.4881 |
| **1800** | tr|B4DKL7|B4DKL7_HUMAN |  | 1 | 1.3 | 0.074 | 16 | 0.0005537 |
| **1801** | sp|Q07812|BAX_HUMAN |  | 1 | 0.92 | 0.03 | 16 | 0.1055 |
| **1802** | tr|A8K6V7|A8K6V7_HUMAN |  | 4 | 0.96 | 0.02 | 16 | 0.3178 |
| **1803** | tr|B4DRL9|B4DRL9_HUMAN |  | 1 | 0.92 | 0.032 | 16 | 0.07942 |
| **1804** | sp|Q8TD19|NEK9_HUMAN |  | 2 | 1.15 | 0.037 | 16 | 0.008493 |
| **1805** | sp|Q15628|TRADD_HUMAN |  | 2 | 0.9 | 0.04 | 16 | 0.06952 |
| **1806** | tr|F5H2R5|F5H2R5_HUMAN |  | 1 | 1.12 | 0.03 | 16 | 0.01626 |
| **1807** | sp|Q9BS40|LXN_HUMAN |  | 2 | 1.08 | 0.011 | 16 | 0.006104 |
| **1808** | tr|A0A087WX17|A0A087WX17_HUMAN |  | 7 | 0.99 | 0.009 | 16 | 0.708 |
| **1809** | tr|A0A0A0MSJ3|A0A0A0MSJ3_HUMAN |  | 4 | 0.99 | 0.012 | 16 | 0.7492 |
| **1810** | tr|B3KNK4|B3KNK4_HUMAN |  | 1 | 0.86 | 0.027 | 16 | 0.003849 |
| **1811** | tr|B4E1B3|B4E1B3_HUMAN |  | 6 | 0.9 | 0.146 | 16 | 0.3011 |
| **1812** | tr|K7EKH5|K7EKH5_HUMAN |  | 1 | 1.3 | 1.2 | 16 | 0.2873 |
| **1813** | sp|O43314|VIP2_HUMAN |  | 11 | 1.02 | 0.002 | 16 | 0.2424 |
| **1814** | tr|B3KT02|B3KT02_HUMAN |  | 1 | 1.55 | 0.279 | 16 | 0.0007711 |
| **1815** | tr|Q59H95|Q59H95_HUMAN |  | 3 | 1.13 | 0.004 | 16 | 4.92E-07 |
| **1816** | sp|Q99766|ATP5S_HUMAN |  | 1 | 0.9 | 0.032 | 16 | 0.03747 |
| **1817** | tr|Q69YH0|Q69YH0_HUMAN |  | 1 | 1.24 | 0.12 | 16 | 0.01411 |
| **1818** | tr|A2MYD4|A2MYD4_HUMAN |  | 1 | 0.94 | 0.011 | 16 | 0.04637 |
| **1819** | sp|Q8WVJ2|NUDC2_HUMAN |  | 5 | 1.04 | 0.002 | 16 | 0.00135 |
| **1820** | tr|A8K651|A8K651_HUMAN |  | 1 | 0.98 | 0.041 | 16 | 0.7544 |
| **1821** | sp|Q96SB8|SMC6_HUMAN |  | 1 | 1.09 | 0.098 | 16 | 0.285 |
| **1822** | tr|B3KQJ1|B3KQJ1_HUMAN |  | 1 | 0.79 | 0.038 | 16 | 0.0005402 |
| **1823** | tr|B3KPR5|B3KPR5_HUMAN |  | 1 | 1.01 | 0.126 | 16 | 0.8941 |
| **1824** | sp|P43686|PRS6B_HUMAN |  | 14 | 0.98 | 0.006 | 16 | 0.3208 |
| **1825** | sp|Q92530|PSMF1_HUMAN |  | 5 | 1.17 | 0.018 | 16 | 0.0001095 |
| **1826** | tr|J3QS39|J3QS39_HUMAN |  | 4 | 0.92 | 0.004 | 16 | 7.54E-05 |
| **1827** | sp|P23527|H2B1O_HUMAN |  | 2 | 3.29 | 0.068 | 16 | 7.91E-16 |
| **1828** | tr|D3DVA3|D3DVA3_HUMAN |  | 3 | 0.94 | 0.009 | 16 | 0.03638 |
| **1829** | tr|H7C1K0|H7C1K0_HUMAN |  | 1 | 0.89 | 0.041 | 16 | 0.04172 |
| **1830** | sp|P19652|A1AG2_HUMAN |  | 1 | 1.03 | 0.058 | 16 | 0.6681 |
| **1831** | sp|Q9UK41|VPS28_HUMAN |  | 5 | 0.96 | 0.003 | 16 | 0.01142 |
| **1832** | sp|Q9NQY0|BIN3_HUMAN |  | 2 | 0.89 | 0.009 | 16 | 0.000487 |
| **1833** | tr|L8E7U6|L8E7U6_HUMAN |  | 1 | 1.25 | 0.184 | 16 | 0.03465 |
| **1834** | tr|Q7Z3F8|Q7Z3F8_HUMAN |  | 1 | 0.97 | 0.068 | 16 | 0.6252 |
| **1835** | sp|Q99536|VAT1_HUMAN |  | 3 | 1.06 | 0.006 | 16 | 0.009905 |
| **1836** | tr|Q53F93|Q53F93_HUMAN |  | 16 | 0.96 | 0.036 | 16 | 0.3934 |
| **1837** | tr|B4DJD7|B4DJD7_HUMAN |  | 1 | 1.02 | 0.094 | 16 | 0.8278 |
| **1838** | sp|P60953|CDC42_HUMAN |  | 4 | 1.04 | 0.004 | 16 | 0.0303 |
| **1839** | sp|Q12955|ANK3_HUMAN |  | 1 | 0.82 | 0.015 | 16 | 4.24E-05 |
| **1840** | tr|B2R7P8|B2R7P8_HUMAN |  | 3 | 0.92 | 0.004 | 16 | 7.47E-05 |
| **1841** | tr|A0A0A0MSI0|A0A0A0MSI0_HUMAN |  | 2 | 0.85 | 0.03 | 16 | 0.003862 |
| **1842** | tr|B4DEX8|B4DEX8_HUMAN |  | 4 | 1.02 | 0.026 | 16 | 0.7091 |
| **1843** | tr|B4DJ85|B4DJ85_HUMAN |  | 3 | 0.98 | 0.019 | 16 | 0.5983 |
| **1844** | tr|Q53HU0|Q53HU0_HUMAN |  | 32 | 1.01 | 0.014 | 16 | 0.732 |
| **1845** | tr|G3XAL0|G3XAL0_HUMAN |  | 1 | 0.94 | 0.062 | 16 | 0.3868 |
| **1846** | sp|P00492|HPRT_HUMAN |  | 6 | 0.95 | 0.001 | 16 | 6.68E-05 |
| **1847** | sp|Q9P209|CEP72_HUMAN |  | 1 | 0.82 | 0.05 | 16 | 0.004922 |
| **1848** | tr|A0A024R5R2|A0A024R5R2_HUMAN |  | 1 | 1.11 | 0.013 | 16 | 0.002229 |
| **1849** | sp|Q8IUI8|CRLF3_HUMAN |  | 10 | 0.97 | 0.004 | 16 | 0.1022 |
| **1850** | tr|Q9H2G0|Q9H2G0_HUMAN |  | 9 | 0.91 | 0.005 | 16 | 0.0001385 |
| **1851** | tr|C9JAM8|C9JAM8_HUMAN |  | 1 | 0.85 | 0.121 | 16 | 0.09469 |
| **1852** | sp|P01112|RASH_HUMAN |  | 1 | 1.15 | 0.035 | 16 | 0.005563 |
| **1853** | sp|Q9H5X1|FA96A_HUMAN |  | 2 | 0.93 | 0.002 | 16 | 1.91E-05 |
| **1854** | tr|Q5HYM2|Q5HYM2_HUMAN |  | 7 | 1.15 | 0.009 | 16 | 1.85E-05 |
| **1855** | tr|B4DE78|B4DE78_HUMAN |  | 4 | 1.08 | 0.004 | 16 | 0.000136 |
| **1856** | tr|A0A087X0P6|A0A087X0P6_HUMAN |  | 1 | 0.67 | 0.018 | 16 | 7.20E-08 |
| **1857** | tr|F8W9W0|F8W9W0_HUMAN |  | 1 | 1.11 | 0.066 | 16 | 0.1189 |
| **1858** | tr|Q2N1I3|Q2N1I3_HUMAN |  | 1 | 1.11 | 0.057 | 16 | 0.09647 |
| **1859** | sp|P00338|LDHA_HUMAN |  | 3 | 1.1 | 0.087 | 16 | 0.2086 |
| **1860** | tr|C9J015|C9J015_HUMAN |  | 1 | 0.95 | 1.851 | 16 | 0.8766 |
| **1861** | sp|Q9Y613|FHOD1_HUMAN |  | 9 | 0.99 | 0.022 | 16 | 0.753 |
| **1862** | tr|Q6FI51|Q6FI51_HUMAN |  | 7 | 1.05 | 0.008 | 16 | 0.04576 |
| **1863** | tr|B4DKZ9|B4DKZ9_HUMAN |  | 2 | 1.12 | 0.086 | 16 | 0.1136 |
| **1864** | sp|Q9NZL4|HPBP1_HUMAN |  | 3 | 1.07 | 0.002 | 16 | 2.05E-05 |
| **1865** | tr|H3BVC7|H3BVC7_HUMAN |  | 1 | 0.99 | 0.026 | 16 | 0.7471 |
| **1866** | tr|Q0VG54|Q0VG54_HUMAN |  | 12 | 0.95 | 0.012 | 16 | 0.1127 |
| **1867** | sp|P21980|TGM2_HUMAN |  | 25 | 0.96 | 0.022 | 16 | 0.2982 |
| **1868** | tr|A0A087WYU2|A0A087WYU2_HUMAN |  | 1 | 1.04 | 0.085 | 16 | 0.5584 |
| **1869** | tr|F8W733|F8W733_HUMAN |  | 5 | 0.99 | 0.008 | 16 | 0.5563 |
| **1870** | tr|Q53ET2|Q53ET2_HUMAN |  | 5 | 0.97 | 0.014 | 16 | 0.328 |
| **1871** | tr|Q6NX68|Q6NX68_HUMAN |  | 1 | 0.9 | 0.01 | 16 | 0.001432 |
| **1872** | tr|Q59F96|Q59F96_HUMAN |  | 4 | 1.01 | 0.015 | 16 | 0.8603 |
| **1873** | tr|D6REX5|D6REX5_HUMAN |  | 3 | 0.91 | 0.006 | 16 | 0.0002171 |
| **1874** | sp|Q96DL1|NXPE2_HUMAN |  | 1 | 1.75 | 0.519 | 16 | 0.0008355 |
| **1875** | sp|Q9H853|TBA4B_HUMAN |  | 1 | 0.94 | 0.033 | 16 | 0.2174 |
| **1876** | sp|P02766|TTHY_HUMAN |  | 3 | 1.01 | 0.009 | 16 | 0.7276 |
| **1877** | tr|B7ZAY6|B7ZAY6_HUMAN |  | 2 | 0.88 | 0.078 | 16 | 0.09546 |
| **1878** | tr|Q96T46|Q96T46_HUMAN |  | 1 | 1 | 0.088 | 16 | 0.9947 |
| **1879** | sp|P00918|CAH2_HUMAN |  | 3 | 1.03 | 0.09 | 16 | 0.7172 |
| **1880** | tr|B3KSI7|B3KSI7_HUMAN |  | 6 | 1.11 | 0.03 | 16 | 0.02759 |
| **1881** | tr|J3QRM1|J3QRM1_HUMAN |  | 1 | 0.83 | 0.131 | 16 | 0.08506 |
| **1882** | sp|Q53H96|P5CR3_HUMAN |  | 1 | 1.04 | 0.025 | 16 | 0.3242 |
| **1883** | sp|P05556|ITB1_HUMAN |  | 1 | 0.98 | 0.074 | 16 | 0.7925 |
| **1884** | sp|Q8NFF5|FAD1_HUMAN |  | 1 | 1 | 0.008 | 16 | 0.9978 |
| **1885** | sp|O75165|DJC13_HUMAN |  | 14 | 0.94 | 0.01 | 16 | 0.03613 |
| **1886** | tr|B0YIW1|B0YIW1_HUMAN |  | 4 | 0.85 | 0.019 | 16 | 0.000517 |
| **1887** | tr|A0JLQ2|A0JLQ2_HUMAN |  | 1 | 1.48 | 0.233 | 16 | 0.001154 |
| **1888** | sp|Q9UM07|PADI4_HUMAN |  | 4 | 1.45 | 0.036 | 16 | 1.05E-07 |
| **1889** | sp|Q9NT62|ATG3_HUMAN |  | 10 | 0.95 | 0.002 | 16 | 0.0002293 |
| **1890** | tr|K7ENJ3|K7ENJ3_HUMAN |  | 4 | 1.11 | 0.069 | 16 | 0.102 |
| **1891** | tr|B4DK32|B4DK32_HUMAN |  | 3 | 0.85 | 0.02 | 16 | 0.0008894 |
| **1892** | sp|P37198|NUP62_HUMAN |  | 2 | 0.98 | 0.027 | 16 | 0.5842 |
| **1893** | sp|Q8N3D4|EH1L1_HUMAN |  | 19 | 0.98 | 0.009 | 16 | 0.365 |
| **1894** | tr|H7BYY1|H7BYY1_HUMAN |  | 2 | 0.73 | 0.014 | 16 | 1.97E-07 |
| **1895** | sp|Q8N1F7|NUP93_HUMAN |  | 3 | 1.11 | 0.023 | 16 | 0.01224 |
| **1896** | tr|F5H442|F5H442_HUMAN |  | 8 | 0.99 | 0.002 | 16 | 0.1976 |
| **1897** | tr|F8WD07|F8WD07_HUMAN |  | 1 | 1.07 | 0.051 | 16 | 0.209 |
| **1898** | tr|Q5T4K5|Q5T4K5_HUMAN |  | 1 | 0.81 | 0.031 | 16 | 0.000623 |
| **1899** | sp|P06314|KV404_HUMAN |  | 1 | 1.07 | 0.026 | 16 | 0.13 |
| **1900** | tr|D9YZV5|D9YZV5_HUMAN |  | 1 | 1.06 | 0.016 | 16 | 0.06978 |
| **1901** | tr|A0A075B6N7|A0A075B6N7_HUMAN |  | 1 | 0.91 | 0.2 | 16 | 0.4256 |
| **1902** | tr|Q8IYQ9|Q8IYQ9_HUMAN |  | 4 | 1.01 | 0.038 | 16 | 0.8262 |
| **1903** | tr|B3KMV1|B3KMV1_HUMAN |  | 1 | 0.91 | 0.008 | 16 | 0.0009955 |
| **1904** | tr|D3VVF8|D3VVF8_HUMAN |  | 4 | 0.96 | 0.007 | 16 | 0.06061 |
| **1905** | sp|O43149|ZZEF1_HUMAN |  | 18 | 0.97 | 0.003 | 16 | 0.04282 |
| **1906** | sp|Q6ZRK6|CCD73_HUMAN |  | 1 | 1.01 | 0.164 | 16 | 0.8986 |
| **1907** | tr|F8VUA6|F8VUA6_HUMAN |  | 1 | 0.98 | 0.025 | 16 | 0.6148 |
| **1908** | sp|Q9H7D7|WDR26_HUMAN |  | 5 | 1 | 0.009 | 16 | 0.9309 |
| **1909** | sp|Q8IVV2|LOXH1_HUMAN |  | 1 | 0.87 | 0.008 | 16 | 6.27E-05 |
| **1910** | tr|F5GY68|F5GY68_HUMAN |  | 1 | 1.15 | 0.01 | 16 | 2.66E-05 |
| **1911** | tr|V9HW34|V9HW34_HUMAN |  | 1 | 1.1 | 0.028 | 16 | 0.03631 |
| **1912** | sp|Q13627|DYR1A_HUMAN |  | 2 | 0.79 | 0.02 | 16 | 2.20E-05 |
| **1913** | sp|P84090|ERH_HUMAN |  | 1 | 1.98 | 0.176 | 16 | 1.21E-07 |
| **1914** | sp|P42858|HD_HUMAN |  | 11 | 0.99 | 0.004 | 16 | 0.7428 |
| **1915** | tr|B2R773|B2R773_HUMAN |  | 2 | 1.04 | 0.019 | 16 | 0.2422 |
| **1916** | tr|H7C2R7|H7C2R7_HUMAN |  | 2 | 0.96 | 0.028 | 16 | 0.4062 |
| **1917** | tr|A0A024RAV4|A0A024RAV4_HUMAN |  | 2 | 0.78 | 0.009 | 16 | 1.37E-07 |
| **1918** | tr|E7ETK0|E7ETK0_HUMAN |  | 2 | 0.89 | 0.03 | 16 | 0.02598 |
| **1919** | sp|Q13287|NMI_HUMAN |  | 1 | 1.34 | 0.209 | 16 | 0.009692 |
| **1920** | tr|A0A024R8Q8|A0A024R8Q8_HUMAN |  | 1 | 0.97 | 0.06 | 16 | 0.5892 |
| **1921** | tr|M0R1H9|M0R1H9_HUMAN |  | 1 | 1.14 | 0.02 | 16 | 0.001508 |
| **1922** | tr|K7EL99|K7EL99_HUMAN |  | 1 | 1.17 | 0.133 | 16 | 0.08023 |
| **1923** | sp|P50570|DYN2_HUMAN |  | 14 | 0.98 | 0.013 | 16 | 0.587 |
| **1924** | tr|H0YCY8|H0YCY8_HUMAN |  | 1 | 1.19 | 0.041 | 16 | 0.002176 |
| **1925** | sp|Q9NPQ8|RIC8A_HUMAN |  | 14 | 0.89 | 0.008 | 16 | 0.0001407 |
| **1926** | sp|Q96ST8|CEP89_HUMAN |  | 1 | 1.14 | 0.186 | 16 | 0.2111 |
| **1927** | sp|Q16543|CDC37_HUMAN |  | 8 | 0.95 | 0.004 | 16 | 0.006103 |
| **1928** | tr|A8K885|A8K885_HUMAN |  | 3 | 1.01 | 0.03 | 16 | 0.7415 |
| **1929** | tr|K7ESQ0|K7ESQ0_HUMAN |  | 1 | 1.12 | 0.049 | 16 | 0.04971 |
| **1930** | tr|B4DEP6|B4DEP6_HUMAN |  | 5 | 1.13 | 0.01 | 16 | 0.0001763 |
| **1931** | sp|P84095|RHOG_HUMAN |  | 1 | 1.36 | 0.097 | 16 | 0.0003102 |
| **1932** | tr|K0J5S8|K0J5S8_HUMAN |  | 4 | 0.98 | 0.017 | 16 | 0.479 |
| **1933** | tr|I3L1Q9|I3L1Q9_HUMAN |  | 1 | 0.64 | 0.034 | 16 | 1.37E-06 |
| **1934** | sp|P07195|LDHB_HUMAN |  | 2 | 1.06 | 0.116 | 16 | 0.5011 |
| **1935** | sp|Q05655|KPCD_HUMAN |  | 2 | 1.09 | 0.026 | 16 | 0.03918 |
| **1936** | tr|B4DM63|B4DM63_HUMAN |  | 2 | 1.28 | 0.026 | 16 | 3.82E-06 |
| **1937** | tr|A6NJH9|A6NJH9_HUMAN |  | 2 | 1 | 0.023 | 16 | 0.9792 |
| **1938** | tr|H7BXG7|H7BXG7_HUMAN |  | 5 | 1 | 0.015 | 16 | 0.9198 |
| **1939** | tr|Q96L35|Q96L35_HUMAN |  | 1 | 0.95 | 0.064 | 16 | 0.4526 |
| **1940** | tr|B7Z2X4|B7Z2X4_HUMAN |  | 11 | 1.25 | 0.002 | 16 | 2.11E-12 |
| **1941** | sp|Q8TDL5|BPIB1_HUMAN |  | 1 | 1.23 | 0.036 | 16 | 0.0002438 |
| **1942** | tr|D6RF93|D6RF93_HUMAN |  | 2 | 0.88 | 0.013 | 16 | 0.0008107 |
| **1943** | tr|M0R389|M0R389_HUMAN |  | 2 | 1.1 | 0.052 | 16 | 0.1109 |
| **1944** | tr|Q4W5L2|Q4W5L2_HUMAN |  | 5 | 1.4 | 0.063 | 16 | 1.30E-05 |
| **1945** | sp|A0AVK6|E2F8_HUMAN |  | 1 | 0.93 | 0.304 | 16 | 0.6193 |
| **1946** | tr|H3BR23|H3BR23_HUMAN |  | 2 | 1.04 | 0.03 | 16 | 0.4294 |
| **1947** | tr|H3BP52|H3BP52_HUMAN |  | 1 | 0.94 | 0.069 | 16 | 0.3904 |
| **1948** | tr|F6QUH3|F6QUH3_HUMAN |  | 1 | 1.04 | 0.095 | 16 | 0.5687 |
| **1949** | sp|Q9UN37|VPS4A_HUMAN |  | 2 | 0.98 | 0.01 | 16 | 0.392 |
| **1950** | tr|E9PN45|E9PN45_HUMAN |  | 1 | 1.25 | 0.154 | 16 | 0.02362 |
| **1951** | tr|A8K3D5|A8K3D5_HUMAN |  | 2 | 0.95 | 0.015 | 16 | 0.158 |
| **1952** | tr|C9JM01|C9JM01_HUMAN |  | 1 | 1.06 | 0.06 | 16 | 0.3824 |
| **1953** | tr|F6U1T9|F6U1T9_HUMAN |  | 1 | 0.98 | 0.009 | 16 | 0.382 |
| **1954** | tr|C9JA93|C9JA93_HUMAN |  | 1 | 1 | 0.07 | 16 | 0.9651 |
| **1955** | tr|Q5EFE6|Q5EFE6_HUMAN |  | 1 | 1.08 | 0.078 | 16 | 0.2982 |
| **1956** | tr|B7ZLY3|B7ZLY3_HUMAN |  | 5 | 0.95 | 0.015 | 16 | 0.1168 |
| **1957** | tr|D3DPQ1|D3DPQ1_HUMAN |  | 1 | 1.08 | 0.036 | 16 | 0.09735 |
| **1958** | sp|P01597|KV105_HUMAN |  | 1 | 0.88 | 0.007 | 16 | 4.67E-05 |
| **1959** | sp|Q99471|PFD5_HUMAN |  | 1 | 0.88 | 0.009 | 16 | 0.0001107 |
| **1960** | tr|A4FVC0|A4FVC0_HUMAN |  | 26 | 0.93 | 0.002 | 16 | 1.50E-05 |
| **1961** | tr|Q4LE79|Q4LE79_HUMAN |  | 2 | 0.9 | 0.085 | 16 | 0.1884 |
| **1962** | sp|P01024|CO3_HUMAN |  | 74 | 1.17 | 0.08 | 16 | 0.03104 |
| **1963** | sp|Q562R1|ACTBL_HUMAN |  | 2 | 1.04 | 0.021 | 16 | 0.2931 |
| **1964** | tr|H3BQP1|H3BQP1_HUMAN |  | 2 | 1.07 | 0.091 | 16 | 0.3641 |
| **1965** | sp|Q02413|DSG1_HUMAN |  | 1 | 0.59 | 0.062 | 16 | 9.15E-06 |
| **1966** | tr|A2NYQ9|A2NYQ9_HUMAN |  | 1 | 0.88 | 0.088 | 16 | 0.1391 |
| **1967** | tr|J3KPY9|J3KPY9_HUMAN |  | 1 | 0.93 | 0.03 | 16 | 0.122 |
| **1968** | tr|A0A024R4X0|A0A024R4X0_HUMAN |  | 2 | 0.84 | 0.022 | 16 | 0.0007297 |
| **1969** | sp|Q9UJ04|TSYL4_HUMAN |  | 1 | 0.87 | 0.014 | 16 | 0.0006176 |
| **1970** | tr|B7Z6W7|B7Z6W7_HUMAN |  | 1 | 1.02 | 0.037 | 16 | 0.7022 |
| **1971** | sp|Q15276|RABE1_HUMAN |  | 13 | 1 | 0.002 | 16 | 0.8914 |
| **1972** | tr|H3BN64|H3BN64_HUMAN |  | 2 | 0.91 | 0.01 | 16 | 0.002414 |
| **1973** | tr|A0A087WYU1|A0A087WYU1_HUMAN |  | 8 | 0.93 | 0.003 | 16 | 0.000147 |
| **1974** | tr|B1AJY5|B1AJY5_HUMAN |  | 7 | 1.05 | 0.005 | 16 | 0.01112 |
| **1975** | tr|A0A087WTK4|A0A087WTK4_HUMAN |  | 1 | 1.35 | 0.372 | 16 | 0.03819 |
| **1976** | tr|B4DVV1|B4DVV1_HUMAN |  | 10 | 1.07 | 0.003 | 16 | 0.0001151 |
| **1977** | sp|P04792|HSPB1_HUMAN |  | 4 | 1.33 | 0.149 | 16 | 0.003463 |
| **1978** | tr|A6XGP7|A6XGP7_HUMAN |  | 1 | 0.87 | 0.063 | 16 | 0.05116 |
| **1979** | sp|P02008|HBAZ_HUMAN |  | 8 | 1.08 | 0.216 | 16 | 0.5084 |
| **1980** | sp|P00558|PGK1_HUMAN |  | 1 | 0.92 | 0.012 | 16 | 0.01093 |
| **1981** | sp|P04040|CATA_HUMAN |  | 12 | 0.98 | 0.01 | 16 | 0.5112 |
| **1982** | tr|E9PLK3|E9PLK3_HUMAN |  | 16 | 0.91 | 0.005 | 16 | 0.0003327 |
| **1983** | tr|E1CEI4|E1CEI4_HUMAN |  | 6 | 0.98 | 0.006 | 16 | 0.3942 |
| **1984** | tr|Q4JLR8|Q4JLR8_HUMAN |  | 1 | 0.83 | 0.112 | 16 | 0.05644 |
| **1985** | sp|Q96KQ7|EHMT2_HUMAN |  | 1 | 1.06 | 0.051 | 16 | 0.2726 |
| **1986** | sp|Q8ND04|SMG8_HUMAN |  | 1 | 0.83 | 0.048 | 16 | 0.008405 |
| **1987** | tr|Q7Z2Z4|Q7Z2Z4_HUMAN |  | 1 | 1.16 | 0.086 | 16 | 0.03984 |
| **1988** | sp|P36543|VATE1_HUMAN |  | 8 | 1.09 | 0.006 | 16 | 0.0002123 |
| **1989** | sp|Q15365|PCBP1_HUMAN |  | 8 | 0.94 | 0.015 | 16 | 0.05002 |
| **1990** | sp|Q9BTE6|AASD1_HUMAN |  | 6 | 1 | 0.006 | 16 | 0.9676 |
| **1991** | sp|P11441|UBL4A_HUMAN |  | 3 | 1 | 0.008 | 16 | 0.8538 |
| **1992** | tr|A8K931|A8K931_HUMAN |  | 1 | 1.21 | 0.056 | 16 | 0.002627 |
| **1993** | tr|J9JIH1|J9JIH1_HUMAN |  | 1 | 1.51 | 0.502 | 16 | 0.01183 |
| **1994** | tr|H0Y509|H0Y509_HUMAN |  | 1 | 0.98 | 0.028 | 16 | 0.6017 |
| **1995** | sp|P35236|PTN7_HUMAN |  | 7 | 0.97 | 0.01 | 16 | 0.2944 |
| **1996** | tr|Q6FHW3|Q6FHW3_HUMAN |  | 2 | 1.15 | 0.022 | 16 | 0.001074 |
| **1997** | tr|F8VX22|F8VX22_HUMAN |  | 1 | 0.58 | 0.093 | 16 | 6.47E-05 |
| **1998** | tr|Q5T985|Q5T985_HUMAN |  | 11 | 1.2 | 0.013 | 16 | 3.80E-06 |
| **1999** | sp|Q6P4A8|PLBL1_HUMAN |  | 1 | 0.87 | 0.022 | 16 | 0.004455 |
| **2000** | tr|B3KS18|B3KS18_HUMAN |  | 1 | 1.05 | 0.013 | 16 | 0.07634 |
| **2001** | sp|P18077|RL35A_HUMAN |  | 1 | 1.05 | 0.052 | 16 | 0.3518 |
| **2002** | tr|G5E9A6|G5E9A6_HUMAN |  | 9 | 0.88 | 0.012 | 16 | 0.0007819 |
| **2003** | tr|E9PJ29|E9PJ29_HUMAN |  | 1 | 0.93 | 0.039 | 16 | 0.1849 |
| **2004** | tr|D3DSP9|D3DSP9_HUMAN |  | 1 | 1.17 | 0.03 | 16 | 0.001675 |
| **2005** | tr|A0A059RTC0|A0A059RTC0_HUMAN |  | 1 | 1.11 | 0.035 | 16 | 0.02851 |
| **2006** | tr|B4E364|B4E364_HUMAN |  | 1 | 0.97 | 0.071 | 16 | 0.6602 |
| **2007** | tr|A8K7F6|A8K7F6_HUMAN |  | 9 | 1.17 | 0.006 | 16 | 3.08E-07 |
| **2008** | sp|P48637|GSHB_HUMAN |  | 2 | 0.81 | 0.121 | 16 | 0.04668 |
| **2009** | sp|Q9Y3F4|STRAP_HUMAN |  | 5 | 1.02 | 0.015 | 16 | 0.494 |
| **2010** | tr|B4E344|B4E344_HUMAN |  | 1 | 1.11 | 0.003 | 16 | 1.16E-06 |
| **2011** | sp|O94829|IPO13_HUMAN |  | 5 | 1.04 | 0.014 | 16 | 0.154 |
| **2012** | sp|Q92539|LPIN2_HUMAN |  | 6 | 0.9 | 0.002 | 16 | 5.78E-07 |
| **2013** | tr|B4DI39|B4DI39_HUMAN |  | 10 | 1 | 0.004 | 16 | 0.8737 |
| **2014** | tr|Q49AN9|Q49AN9_HUMAN |  | 1 | 1.04 | 0.123 | 16 | 0.632 |
| **2015** | tr|Q3SYF1|Q3SYF1_HUMAN |  | 3 | 1.02 | 0.035 | 16 | 0.7204 |
| **2016** | tr|E9PCB6|E9PCB6_HUMAN |  | 1 | 2.18 | 0.429 | 16 | 3.17E-06 |
| **2017** | tr|Q96K98|Q96K98_HUMAN |  | 2 | 1.26 | 0.024 | 16 | 6.14E-06 |
| **2018** | sp|P54652|HSP72_HUMAN |  | 2 | 0.93 | 0.041 | 16 | 0.1877 |
| **2019** | tr|Q53FL4|Q53FL4_HUMAN |  | 3 | 1.13 | 0.083 | 16 | 0.09876 |
| **2020** | tr|Q59ET3|Q59ET3_HUMAN |  | 19 | 0.96 | 0.001 | 16 | 7.59E-05 |
| **2021** | tr|Q5T8U3|Q5T8U3_HUMAN |  | 1 | 1.03 | 0.03 | 16 | 0.5643 |
| **2022** | tr|J3QR40|J3QR40_HUMAN |  | 1 | 0.81 | 0.069 | 16 | 0.009855 |
| **2023** | tr|B7Z539|B7Z539_HUMAN |  | 4 | 1.1 | 0.029 | 16 | 0.02566 |
| **2024** | tr|B4DNR3|B4DNR3_HUMAN |  | 3 | 0.98 | 0.024 | 16 | 0.5726 |
| **2025** | sp|O60271|JIP4_HUMAN |  | 10 | 0.93 | 0.011 | 16 | 0.014 |
| **2026** | tr|V9HVX6|V9HVX6_HUMAN |  | 12 | 1.02 | 0.004 | 16 | 0.2403 |
| **2027** | tr|S4R3D6|S4R3D6_HUMAN |  | 1 | 0.92 | 0.02 | 16 | 0.03243 |
| **2028** | tr|G1FM90|G1FM90_HUMAN |  | 1 | 1.01 | 0.186 | 16 | 0.8976 |
| **2029** | tr|F2Z3G9|F2Z3G9_HUMAN |  | 1 | 0.88 | 0.122 | 16 | 0.2029 |
| **2030** | sp|Q5SQ64|LY66F_HUMAN |  | 1 | 1.46 | 0.344 | 16 | 0.006848 |
| **2031** | sp|O60547|GMDS_HUMAN |  | 4 | 0.89 | 0.007 | 16 | 0.0001112 |
| **2032** | sp|Q5THR3|EFCB6_HUMAN |  | 1 | 1 | 0.024 | 16 | 0.9849 |
| **2033** | tr|A0A024RAV5|A0A024RAV5_HUMAN |  | 1 | 0.98 | 0.1 | 16 | 0.7608 |
| **2034** | sp|Q8WZA0|LZIC_HUMAN |  | 3 | 1.57 | 0.096 | 16 | 2.30E-06 |
| **2035** | sp|Q9UBQ7|GRHPR_HUMAN |  | 3 | 0.92 | 0.019 | 16 | 0.03032 |
| **2036** | sp|Q9Y566|SHAN1_HUMAN |  | 1 | 1.04 | 0.045 | 16 | 0.4125 |
| **2037** | tr|A0A087X020|A0A087X020_HUMAN |  | 1 | 0.9 | 0.036 | 16 | 0.05388 |
| **2038** | sp|P55795|HNRH2_HUMAN |  | 1 | 1.09 | 0.136 | 16 | 0.3382 |
| **2039** | tr|B4DZE1|B4DZE1_HUMAN |  | 1 | 0.97 | 0.01 | 16 | 0.2162 |
| **2040** | tr|Q1ET65|Q1ET65_HUMAN |  | 2 | 1.05 | 0.146 | 16 | 0.574 |
| **2041** | tr|E5RFJ0|E5RFJ0_HUMAN |  | 1 | 1 | 0.053 | 16 | 0.9821 |
| **2042** | tr|B4E3I0|B4E3I0_HUMAN |  | 1 | 1.19 | 0.02 | 16 | 7.36E-05 |
| **2043** | tr|C9JJ34|C9JJ34_HUMAN |  | 3 | 1.01 | 0.051 | 16 | 0.9092 |
| **2044** | tr|Q53FV4|Q53FV4_HUMAN |  | 5 | 1.12 | 0.038 | 16 | 0.02478 |
| **2045** | sp|Q96G03|PGM2_HUMAN |  | 7 | 0.94 | 0.033 | 16 | 0.2233 |
| **2046** | sp|Q92621|NU205_HUMAN |  | 1 | 0.96 | 0.03 | 16 | 0.392 |
| **2047** | tr|Q86TT1|Q86TT1_HUMAN |  | 1 | 1 | 0.066 | 16 | 0.9525 |
| **2048** | tr|B4DRC7|B4DRC7_HUMAN |  | 1 | 1.05 | 0.027 | 16 | 0.2192 |
| **2049** | tr|C9JNG9|C9JNG9_HUMAN |  | 2 | 1.09 | 0.004 | 16 | 6.78E-05 |
| **2050** | sp|Q96LR2|LURA1_HUMAN |  | 1 | 1.15 | 0.074 | 16 | 0.04719 |
| **2051** | tr|B2R582|B2R582_HUMAN |  | 1 | 0.95 | 0.009 | 16 | 0.07193 |
| **2052** | sp|A0AVT1|UBA6_HUMAN |  | 2 | 1.25 | 0.023 | 16 | 8.47E-06 |
| **2053** | sp|P05154|IPSP_HUMAN |  | 3 | 0.89 | 0.013 | 16 | 0.001747 |
| **2054** | tr|B3KXN4|B3KXN4_HUMAN |  | 10 | 0.93 | 0.003 | 16 | 5.33E-05 |
| **2055** | sp|P01769|HV308_HUMAN |  | 1 | 1.1 | 0.054 | 16 | 0.09392 |
| **2056** | tr|Q149P1|Q149P1_HUMAN |  | 6 | 0.97 | 0.025 | 16 | 0.43 |
| **2057** | sp|O75367|H2AY_HUMAN |  | 1 | 1.22 | 0.041 | 16 | 0.0006141 |
| **2058** | sp|P78509|RELN_HUMAN |  | 3 | 0.77 | 0.033 | 16 | 0.0001789 |
| **2059** | sp|P0C7P3|SLN14_HUMAN |  | 2 | 0.91 | 0.052 | 16 | 0.1366 |
| **2060** | tr|Q96CA8|Q96CA8_HUMAN |  | 1 | 1.29 | 0.074 | 16 | 0.0006709 |
| **2061** | tr|A0A024R9E2|A0A024R9E2_HUMAN |  | 5 | 1.03 | 0.004 | 16 | 0.1022 |
| **2062** | sp|B1AK53|ESPN_HUMAN |  | 1 | 1.26 | 0.045 | 16 | 0.0002001 |
| **2063** | sp|A4D1P6|WDR91_HUMAN |  | 13 | 0.91 | 0.001 | 16 | 3.53E-08 |
| **2064** | tr|B4DXV1|B4DXV1_HUMAN |  | 2 | 1.25 | 0.068 | 16 | 0.0018 |
| **2065** | sp|P50238|CRIP1_HUMAN |  | 1 | 0.67 | 0.014 | 16 | 1.39E-08 |
| **2066** | sp|O15144|ARPC2_HUMAN |  | 2 | 1.23 | 0.021 | 16 | 1.59E-05 |
| **2067** | tr|X5D2F4|X5D2F4_HUMAN |  | 7 | 0.93 | 0.02 | 16 | 0.08322 |
| **2068** | sp|Q92841|DDX17_HUMAN |  | 7 | 1.07 | 0.002 | 16 | 2.11E-05 |
| **2069** | tr|F8W020|F8W020_HUMAN |  | 1 | 0.83 | 0.036 | 16 | 0.003057 |
| **2070** | tr|Q53HN4|Q53HN4_HUMAN |  | 5 | 1 | 0.013 | 16 | 0.9475 |
| **2071** | tr|V9GYG9|V9GYG9_HUMAN |  | 3 | 0.99 | 0.035 | 16 | 0.8996 |
| **2072** | tr|A0A087WWC6|A0A087WWC6_HUMAN |  | 1 | 1.08 | 0.022 | 16 | 0.05648 |
| **2073** | sp|Q9HBH5|RDH14_HUMAN |  | 1 | 1 | 0.033 | 16 | 0.935 |
| **2074** | tr|B3KY43|B3KY43_HUMAN |  | 3 | 0.94 | 0.005 | 16 | 0.002729 |
| **2075** | tr|B2RDG9|B2RDG9_HUMAN |  | 5 | 0.96 | 0.01 | 16 | 0.1708 |
| **2076** | sp|O15084|ANR28_HUMAN |  | 8 | 0.97 | 0.003 | 16 | 0.03075 |
| **2077** | tr|U3KQS7|U3KQS7_HUMAN |  | 2 | 1.11 | 0.023 | 16 | 0.01139 |
| **2078** | tr|V9GYA3|V9GYA3_HUMAN |  | 1 | 0.96 | 0.002 | 16 | 0.0006638 |
| **2079** | tr|Q5VW52|Q5VW52_HUMAN |  | 1 | 1.08 | 0.145 | 16 | 0.3925 |
| **2080** | tr|Q53SY7|Q53SY7_HUMAN |  | 21 | 0.88 | 0.005 | 16 | 3.82E-06 |
| **2081** | tr|B7Z229|B7Z229_HUMAN |  | 1 | 0.8 | 0.018 | 16 | 1.84E-05 |
| **2082** | sp|Q5JUX0|SPIN3_HUMAN |  | 1 | 1.23 | 0.294 | 16 | 0.1078 |
| **2083** | tr|K7EJL1|K7EJL1_HUMAN |  | 2 | 1.3 | 0.108 | 16 | 0.002403 |
| **2084** | tr|B3KY29|B3KY29_HUMAN |  | 5 | 0.8 | 0.005 | 16 | 1.77E-08 |
| **2085** | sp|P55060|XPO2_HUMAN |  | 23 | 0.93 | 0.006 | 16 | 0.001525 |
| **2086** | sp|P59768|GBG2_HUMAN |  | 1 | 1.35 | 0.155 | 16 | 0.002955 |
| **2087** | tr|B4DQK1|B4DQK1_HUMAN |  | 8 | 1.01 | 0.006 | 16 | 0.7457 |
| **2088** | tr|H0YA26|H0YA26_HUMAN |  | 3 | 1.02 | 0.016 | 16 | 0.6091 |
| **2089** | sp|P10720|PF4V_HUMAN |  | 1 | 1.07 | 0.032 | 16 | 0.1196 |
| **2090** | sp|P09105|HBAT_HUMAN |  | 6 | 1.03 | 0.012 | 16 | 0.306 |
| **2091** | tr|Q7Z379|Q7Z379_HUMAN |  | 1 | 1.09 | 0.087 | 16 | 0.258 |
| **2092** | tr|F4MH86|F4MH86_HUMAN |  | 1 | 0.82 | 0.092 | 16 | 0.0282 |
| **2093** | sp|P02649|APOE_HUMAN |  | 12 | 1.08 | 0.043 | 16 | 0.1424 |
| **2094** | tr|H0YJW9|H0YJW9_HUMAN |  | 1 | 0.96 | 0.029 | 16 | 0.3744 |
| **2095** | tr|L7UUZ7|L7UUZ7_HUMAN |  | 8 | 0.89 | 0.036 | 16 | 0.03368 |
| **2096** | sp|Q9GZN8|CT027_HUMAN |  | 1 | 0.91 | 0.119 | 16 | 0.297 |
| **2097** | tr|E9PIJ7|E9PIJ7_HUMAN |  | 1 | 1.24 | 0.306 | 16 | 0.09926 |
| **2098** | tr|Q8IVQ8|Q8IVQ8_HUMAN |  | 13 | 0.93 | 0.004 | 16 | 0.0001794 |
| **2099** | tr|A0A087X2D0|A0A087X2D0_HUMAN |  | 1 | 1.29 | 0.018 | 16 | 3.60E-07 |
| **2100** | sp|P01699|LV101_HUMAN |  | 1 | 0.66 | 0.104 | 16 | 0.0006919 |
| **2101** | tr|Q5QPM0|Q5QPM0_HUMAN |  | 1 | 2.03 | 0.305 | 16 | 2.03E-06 |
| **2102** | tr|G3V4X5|G3V4X5_HUMAN |  | 1 | 0.75 | 0.173 | 16 | 0.02966 |
| **2103** | tr|A0A087X1K9|A0A087X1K9_HUMAN |  | 1 | 1.08 | 0.039 | 16 | 0.1065 |
| **2104** | tr|A0A087X130|A0A087X130_HUMAN |  | 1 | 0.96 | 0.039 | 16 | 0.3817 |
| **2105** | sp|Q15907|RB11B_HUMAN |  | 2 | 1 | 0.003 | 16 | 0.8766 |
| **2106** | sp|O43488|ARK72_HUMAN |  | 4 | 0.76 | 0.065 | 16 | 0.001938 |
| **2107** | tr|Q53S54|Q53S54_HUMAN |  | 19 | 0.99 | 0.033 | 16 | 0.8419 |
| **2108** | sp|P42566|EPS15_HUMAN |  | 2 | 0.95 | 0.004 | 16 | 0.006881 |
| **2109** | tr|E9PJ89|E9PJ89_HUMAN |  | 1 | 1.15 | 0.058 | 16 | 0.02531 |
| **2110** | tr|B4DE89|B4DE89_HUMAN |  | 1 | 1.43 | 0.062 | 16 | 4.95E-06 |
| **2111** | sp|Q9H156|SLIK2_HUMAN |  | 1 | 1.05 | 0.051 | 16 | 0.402 |
| **2112** | tr|B3KN47|B3KN47_HUMAN |  | 1 | 0.97 | 0.033 | 16 | 0.5343 |
| **2113** | tr|G3V5M2|G3V5M2_HUMAN |  | 4 | 1.01 | 0.014 | 16 | 0.7612 |
| **2114** | sp|Q9BSF0|SMAKA_HUMAN |  | 1 | 1.04 | 0.027 | 16 | 0.3636 |
| **2115** | sp|Q9BRX2|PELO_HUMAN |  | 1 | 1 | 0.07 | 16 | 0.9763 |
| **2116** | tr|H0YH81|H0YH81_HUMAN |  | 3 | 1.17 | 0.031 | 16 | 0.001339 |
| **2117** | tr|E9PPL6|E9PPL6_HUMAN |  | 1 | 1.18 | 0.042 | 16 | 0.003225 |
| **2118** | sp|Q12765|SCRN1_HUMAN |  | 3 | 1.22 | 0.066 | 16 | 0.004028 |
| **2119** | tr|G3V3D4|G3V3D4_HUMAN |  | 1 | 0.84 | 0.102 | 16 | 0.06754 |
| **2120** | sp|P61966|AP1S1_HUMAN |  | 1 | 0.77 | 0.017 | 16 | 5.34E-06 |
| **2121** | sp|P08253|MMP2_HUMAN |  | 2 | 0.95 | 0.018 | 16 | 0.1726 |
| **2122** | tr|X6RCK5|X6RCK5_HUMAN |  | 3 | 0.85 | 0.01 | 16 | 1.95E-05 |
| **2123** | tr|F8W118|F8W118_HUMAN |  | 1 | 0.75 | 0.134 | 16 | 0.01442 |
| **2124** | sp|Q96A49|SYAP1_HUMAN |  | 4 | 1.08 | 0.013 | 16 | 0.01668 |
| **2125** | sp|P23258|TBG1_HUMAN |  | 1 | 0.89 | 0.024 | 16 | 0.01059 |
| **2126** | sp|Q9NRV9|HEBP1_HUMAN |  | 4 | 1.18 | 0.01 | 16 | 2.17E-06 |
| **2127** | tr|Q53EY8|Q53EY8_HUMAN |  | 9 | 0.84 | 0.093 | 16 | 0.04738 |
| **2128** | sp|Q14571|ITPR2_HUMAN |  | 1 | 1.14 | 0.027 | 16 | 0.004123 |
| **2129** | sp|Q9Y3D0|MIP18_HUMAN |  | 1 | 0.98 | 0.011 | 16 | 0.4365 |
| **2130** | tr|A8MUD9|A8MUD9_HUMAN |  | 2 | 1.09 | 0.01 | 16 | 0.002457 |
| **2131** | tr|B4DGH0|B4DGH0_HUMAN |  | 1 | 1.29 | 0.274 | 16 | 0.03971 |
| **2132** | sp|Q9H3P7|GCP60_HUMAN |  | 1 | 1.54 | 0.227 | 13 | 0.001426 |
| **2133** | tr|Q5TGM0|Q5TGM0_HUMAN |  | 5 | 0.9 | 0.001 | 16 | 1.13E-08 |
| **2134** | tr|S6B2B6|S6B2B6_HUMAN |  | 1 | 1.21 | 0.109 | 16 | 0.02172 |
| **2135** | tr|B3KU09|B3KU09_HUMAN |  | 27 | 0.93 | 0.002 | 16 | 1.97E-05 |
| **2136** | sp|Q9H2K8|TAOK3_HUMAN |  | 5 | 1 | 0.005 | 16 | 0.909 |
| **2137** | tr|Q9HAL7|Q9HAL7_HUMAN |  | 1 | 1.11 | 0.123 | 16 | 0.2122 |
| **2138** | sp|O14744|ANM5_HUMAN |  | 15 | 0.95 | 0.004 | 16 | 0.01118 |
| **2139** | tr|Q0EFC6|Q0EFC6_HUMAN |  | 8 | 0.89 | 0.005 | 16 | 1.17E-05 |
| **2140** | tr|C9JEU5|C9JEU5_HUMAN |  | 3 | 1.13 | 0.071 | 16 | 0.06657 |
| **2141** | tr|H0YN81|H0YN81_HUMAN |  | 5 | 1.02 | 0.007 | 16 | 0.2794 |
| **2142** | tr|E9PHY5|E9PHY5_HUMAN |  | 5 | 0.81 | 0.007 | 16 | 1.58E-07 |
| **2143** | sp|Q9UJW0|DCTN4_HUMAN |  | 6 | 0.92 | 0.008 | 16 | 0.003704 |
| **2144** | sp|P62258|1433E_HUMAN |  | 3 | 1.18 | 0.008 | 16 | 1.49E-06 |
| **2145** | sp|P52294|IMA5_HUMAN |  | 6 | 0.97 | 0.006 | 16 | 0.1915 |
| **2146** | tr|Q05D04|Q05D04_HUMAN |  | 1 | 1.06 | 0.072 | 16 | 0.3764 |
| **2147** | tr|A1A4F3|A1A4F3_HUMAN |  | 2 | 1.04 | 0.052 | 16 | 0.4901 |
| **2148** | sp|Q6PGP7|TTC37_HUMAN |  | 5 | 0.99 | 0.008 | 16 | 0.547 |
| **2149** | tr|B7Z5V2|B7Z5V2_HUMAN |  | 2 | 1.08 | 0.033 | 16 | 0.09152 |
| **2150** | sp|Q96AT9|RPE_HUMAN |  | 2 | 0.89 | 0.048 | 16 | 0.0643 |
| **2151** | tr|D3DP16|D3DP16_HUMAN |  | 1 | 1.54 | 0.335 | 16 | 0.00208 |
| **2152** | tr|B4E1C4|B4E1C4_HUMAN |  | 4 | 0.95 | 0.012 | 16 | 0.1074 |
| **2153** | tr|Q86U79|Q86U79_HUMAN |  | 2 | 1.13 | 0.059 | 16 | 0.04543 |
| **2154** | tr|B4DP62|B4DP62_HUMAN |  | 1 | 2.61 | 6.648 | 16 | 0.02489 |
| **2155** | sp|P53396|ACLY_HUMAN |  | 23 | 1.09 | 0.001 | 16 | 1.29E-08 |
| **2156** | tr|A8K2Y2|A8K2Y2_HUMAN |  | 10 | 0.94 | 0.014 | 16 | 0.05755 |
| **2157** | tr|C9JGR9|C9JGR9_HUMAN |  | 2 | 1.11 | 0.02 | 16 | 0.007057 |
| **2158** | sp|P31942|HNRH3_HUMAN |  | 3 | 0.97 | 0.01 | 16 | 0.2113 |
| **2159** | sp|P20851|C4BPB_HUMAN |  | 3 | 1.07 | 0.011 | 16 | 0.01872 |
| **2160** | tr|Q8IVA8|Q8IVA8_HUMAN |  | 1 | 1.03 | 0.023 | 16 | 0.4312 |
| **2161** | tr|B4DUT7|B4DUT7_HUMAN |  | 5 | 0.97 | 0.049 | 16 | 0.618 |
| **2162** | tr|A0A024R7S3|A0A024R7S3_HUMAN |  | 5 | 0.94 | 0.004 | 16 | 0.001523 |
| **2163** | sp|Q9C0C9|UBE2O_HUMAN |  | 21 | 0.87 | 0.003 | 16 | 9.99E-08 |
| **2164** | tr|Q9H9B7|Q9H9B7_HUMAN |  | 1 | 1.04 | 0.039 | 16 | 0.4638 |
| **2165** | sp|Q9H2G2|SLK_HUMAN |  | 7 | 0.98 | 0.002 | 16 | 0.04406 |
| **2166** | sp|P12814|ACTN1_HUMAN |  | 2 | 1.16 | 0.009 | 16 | 9.36E-06 |
| **2167** | sp|P42025|ACTY_HUMAN |  | 1 | 0.91 | 0.018 | 16 | 0.01379 |
| **2168** | sp|Q9Y4E8|UBP15_HUMAN |  | 20 | 0.91 | 0.005 | 16 | 5.30E-05 |
| **2169** | sp|Q5VVQ6|OTU1_HUMAN |  | 5 | 0.9 | 0.005 | 16 | 0.0001021 |
| **2170** | tr|C9JQD0|C9JQD0_HUMAN |  | 2 | 1.08 | 0.029 | 16 | 0.09004 |
| **2171** | tr|A0A075B785|A0A075B785_HUMAN |  | 1 | 1.11 | 0.053 | 16 | 0.08223 |
| **2172** | sp|P48061|SDF1_HUMAN |  | 1 | 0.88 | 0.069 | 16 | 0.07779 |
| **2173** | sp|P14868|SYDC_HUMAN |  | 14 | 0.96 | 0.007 | 16 | 0.07871 |
| **2174** | tr|A0A088AWK4|A0A088AWK4_HUMAN |  | 1 | 1.2 | 0.044 | 16 | 0.002028 |
| **2175** | tr|B7Z685|B7Z685_HUMAN |  | 1 | 1.18 | 0.208 | 16 | 0.1363 |
| **2176** | tr|Q53FE8|Q53FE8_HUMAN |  | 1 | 1.01 | 0.013 | 16 | 0.8506 |
| **2177** | sp|P02745|C1QA_HUMAN |  | 2 | 0.86 | 0.011 | 16 | 7.23E-05 |
| **2178** | tr|H3BRQ0|H3BRQ0_HUMAN |  | 3 | 1.03 | 0.036 | 16 | 0.5943 |
| **2179** | tr|I3L397|I3L397_HUMAN |  | 4 | 1.42 | 0.03 | 16 | 8.63E-08 |
| **2180** | sp|Q9BTM9|URM1_HUMAN |  | 1 | 0.92 | 0.038 | 16 | 0.1181 |
| **2181** | sp|P10153|RNAS2_HUMAN |  | 1 | 1.06 | 0.073 | 16 | 0.4031 |
| **2182** | sp|P24928|RPB1_HUMAN |  | 1 | 1 | 0.186 | 16 | 0.975 |
| **2183** | tr|H7BXY5|H7BXY5_HUMAN |  | 2 | 1.01 | 0.024 | 16 | 0.8353 |
| **2184** | sp|Q9Y2Y8|PRG3_HUMAN |  | 1 | 1.36 | 0.386 | 16 | 0.03724 |
| **2185** | tr|Q96LQ7|Q96LQ7_HUMAN |  | 1 | 2.12 | 4.065 | 16 | 0.04235 |
| **2186** | tr|E9PR03|E9PR03_HUMAN |  | 1 | 0.84 | 0.093 | 16 | 0.04935 |
| **2187** | sp|P78527|PRKDC_HUMAN |  | 2 | 1.2 | 0.099 | 16 | 0.02131 |
| **2188** | sp|Q8N392|RHG18_HUMAN |  | 4 | 1 | 0.03 | 16 | 0.9638 |
| **2189** | tr|Q5XTR9|Q5XTR9_HUMAN |  | 1 | 0.98 | 0.027 | 16 | 0.6812 |
| **2190** | sp|P13533|MYH6_HUMAN |  | 1 | 0.91 | 0.021 | 16 | 0.0196 |
| **2191** | sp|Q16610|ECM1_HUMAN |  | 6 | 0.85 | 0.009 | 16 | 1.77E-05 |
| **2192** | sp|Q9UNZ2|NSF1C_HUMAN |  | 1 | 0.94 | 0.017 | 16 | 0.1087 |
| **2193** | sp|P17987|TCPA_HUMAN |  | 15 | 0.93 | 0.002 | 16 | 4.88E-05 |
| **2194** | tr|Q8N7A2|Q8N7A2_HUMAN |  | 1 | 1.44 | 0.336 | 16 | 0.007857 |
| **2195** | tr|B2RAP9|B2RAP9_HUMAN |  | 7 | 0.97 | 0.013 | 16 | 0.2791 |
| **2196** | tr|B4DP70|B4DP70_HUMAN |  | 1 | 0.83 | 0.16 | 14 | 0.1378 |
| **2197** | tr|B4DS85|B4DS85_HUMAN |  | 1 | 0.79 | 0.055 | 16 | 0.002573 |
| **2198** | sp|P53680|AP2S1_HUMAN |  | 4 | 0.77 | 0.017 | 16 | 3.29E-06 |
| **2199** | tr|B4DUR7|B4DUR7_HUMAN |  | 4 | 0.94 | 0.008 | 16 | 0.01529 |
| **2200** | sp|Q6UWP2|DHR11_HUMAN |  | 2 | 0.94 | 0.004 | 16 | 0.00187 |
| **2201** | sp|Q96BN8|OTUL_HUMAN |  | 7 | 0.97 | 0.021 | 16 | 0.4597 |
| **2202** | tr|H3BNP9|H3BNP9_HUMAN |  | 1 | 1.14 | 0.174 | 16 | 0.2156 |
| **2203** | sp|Q06124|PTN11_HUMAN |  | 1 | 0.92 | 0.103 | 16 | 0.3192 |
| **2204** | sp|Q96RS6|NUDC1_HUMAN |  | 5 | 1.13 | 0.004 | 16 | 1.03E-06 |
| **2205** | tr|Q2NLC8|Q2NLC8_HUMAN |  | 1 | 0.85 | 0.067 | 16 | 0.03115 |
| **2206** | sp|P07357|CO8A_HUMAN |  | 6 | 1.09 | 0.003 | 16 | 9.45E-06 |
| **2207** | sp|Q709C8|VP13C_HUMAN |  | 13 | 0.94 | 0.006 | 16 | 0.01219 |
| **2208** | sp|Q00534|CDK6_HUMAN |  | 1 | 0.77 | 0.025 | 16 | 3.39E-05 |
| **2209** | tr|C9JU48|C9JU48_HUMAN |  | 2 | 1.09 | 0.035 | 16 | 0.08772 |
| **2210** | sp|Q8N1G4|LRC47_HUMAN |  | 3 | 0.89 | 0.049 | 16 | 0.07411 |
| **2211** | tr|B4DS19|B4DS19_HUMAN |  | 1 | 0.88 | 0.075 | 16 | 0.09959 |
| **2212** | sp|Q9Y570|PPME1_HUMAN |  | 9 | 0.96 | 0.042 | 16 | 0.4369 |
| **2213** | sp|Q9GZP4|PITH1_HUMAN |  | 12 | 1.29 | 0.01 | 16 | 8.72E-09 |
| **2214** | tr|A4D2P2|A4D2P2_HUMAN |  | 2 | 0.94 | 0.031 | 16 | 0.1945 |
| **2215** | tr|Q53T70|Q53T70_HUMAN |  | 2 | 1.2 | 0.184 | 16 | 0.07905 |
| **2216** | sp|Q8N122|RPTOR_HUMAN |  | 3 | 1 | 0.018 | 16 | 0.9883 |
| **2217** | sp|P09917|LOX5_HUMAN |  | 3 | 0.92 | 0.032 | 16 | 0.08721 |
| **2218** | sp|P51692|STA5B_HUMAN |  | 10 | 1.03 | 0.006 | 16 | 0.1963 |
| **2219** | sp|O75636|FCN3_HUMAN |  | 2 | 1.04 | 0.024 | 16 | 0.3047 |
| **2220** | tr|B3KML1|B3KML1_HUMAN |  | 9 | 0.99 | 0.027 | 16 | 0.7526 |
| **2221** | sp|Q9Y244|POMP_HUMAN |  | 1 | 0.86 | 0.054 | 16 | 0.03003 |
| **2222** | tr|Q53F47|Q53F47_HUMAN |  | 1 | 0.99 | 0.033 | 16 | 0.8186 |
| **2223** | tr|B4DWG1|B4DWG1_HUMAN |  | 5 | 0.99 | 0.008 | 16 | 0.5342 |
| **2224** | tr|B4DKU9|B4DKU9_HUMAN |  | 1 | 1.24 | 0.191 | 16 | 0.04683 |
| **2225** | tr|B4DDG2|B4DDG2_HUMAN |  | 1 | 1.06 | 0.757 | 16 | 0.7709 |
| **2226** | sp|Q9UIA9|XPO7_HUMAN |  | 35 | 0.95 | 0.003 | 16 | 0.0006296 |
| **2227** | sp|Q14558|KPRA_HUMAN |  | 7 | 1.11 | 0.001 | 16 | 8.62E-09 |
| **2228** | tr|Q86VX4|Q86VX4_HUMAN |  | 2 | 1.14 | 0.059 | 16 | 0.0401 |
| **2229** | tr|Q6LCG1|Q6LCG1_HUMAN |  | 1 | 1.22 | 0.089 | 16 | 0.009045 |
| **2230** | sp|P41214|EIF2D_HUMAN |  | 3 | 1.01 | 0.017 | 16 | 0.7357 |
| **2231** | tr|Q8NHX6|Q8NHX6_HUMAN |  | 2 | 0.95 | 0.059 | 16 | 0.4549 |
| **2232** | sp|Q9NZL9|MAT2B_HUMAN |  | 3 | 0.98 | 0.029 | 16 | 0.5904 |
| **2233** | tr|D6RBC1|D6RBC1_HUMAN |  | 1 | 0.95 | 0.016 | 16 | 0.1249 |
| **2234** | tr|A0A024R5M3|A0A024R5M3_HUMAN |  | 11 | 0.96 | 0.006 | 16 | 0.04428 |
| **2235** | sp|P17812|PYRG1_HUMAN |  | 8 | 1.07 | 0.009 | 16 | 0.006818 |
| **2236** | sp|P59666|DEF3_HUMAN |  | 3 | 2.99 | 1.984 | 16 | 4.69E-05 |
| **2237** | sp|Q9UKE5|TNIK_HUMAN |  | 1 | 0.9 | 0.012 | 16 | 0.002439 |
| **2238** | tr|F5H1L0|F5H1L0_HUMAN |  | 1 | 1.02 | 0.041 | 16 | 0.6377 |
| **2239** | sp|P01031|CO5_HUMAN |  | 12 | 1.12 | 0.013 | 16 | 0.0007354 |
| **2240** | sp|P50991|TCPD_HUMAN |  | 30 | 0.95 | 0.001 | 16 | 9.96E-05 |
| **2241** | sp|Q9H8M7|F188A_HUMAN |  | 2 | 0.93 | 0.001 | 16 | 4.98E-06 |
| **2242** | tr|A4Q972|A4Q972_HUMAN |  | 1 | 1.07 | 0.067 | 16 | 0.2681 |
| **2243** | tr|A0A087WYL9|A0A087WYL9_HUMAN |  | 2 | 0.88 | 0.008 | 16 | 8.91E-05 |
| **2244** | tr|A0A087WUX6|A0A087WUX6_HUMAN |  | 1 | 1.14 | 0.028 | 16 | 0.003983 |
| **2245** | tr|X6RDF7|X6RDF7_HUMAN |  | 1 | 1.11 | 0.272 | 16 | 0.4104 |
| **2246** | tr|H3BSN6|H3BSN6_HUMAN |  | 1 | 0.75 | 0.061 | 16 | 0.00111 |
| **2247** | tr|B4DM97|B4DM97_HUMAN |  | 1 | 0.99 | 0.127 | 16 | 0.9428 |
| **2248** | tr|Q6P2S0|Q6P2S0_HUMAN |  | 1 | 1.16 | 0.058 | 16 | 0.017 |
| **2249** | tr|K7EPX7|K7EPX7_HUMAN |  | 1 | 1.12 | 0.032 | 16 | 0.02054 |
| **2250** | tr|A0A024R589|A0A024R589_HUMAN |  | 1 | 0.87 | 0.009 | 16 | 5.74E-05 |
| **2251** | tr|C9JAP5|C9JAP5_HUMAN |  | 1 | 0.91 | 0.06 | 16 | 0.1481 |
| **2252** | tr|F8VZJ2|F8VZJ2_HUMAN |  | 4 | 0.96 | 0.007 | 16 | 0.08274 |
| **2253** | tr|E7EV99|E7EV99_HUMAN |  | 10 | 1.12 | 0.096 | 16 | 0.1335 |
| **2254** | tr|B2R5U1|B2R5U1_HUMAN |  | 1 | 1.09 | 0.037 | 16 | 0.09212 |
| **2255** | sp|P01603|KV111_HUMAN |  | 2 | 1.15 | 0.069 | 16 | 0.03921 |
| **2256** | tr|B3KQ20|B3KQ20_HUMAN |  | 5 | 1.03 | 0.015 | 16 | 0.3403 |
| **2257** | sp|P01593|KV101_HUMAN |  | 1 | 1.06 | 0.035 | 16 | 0.1963 |
| **2258** | sp|P11142|HSP7C_HUMAN |  | 4 | 1.1 | 0.009 | 16 | 0.0008769 |
| **2259** | tr|M4PM71|M4PM71_HUMAN |  | 1 | 1.16 | 0.051 | 16 | 0.01103 |
| **2260** | tr|A0A024R7F1|A0A024R7F1_HUMAN |  | 6 | 1.1 | 0.01 | 16 | 0.001085 |
| **2261** | sp|P43490|NAMPT_HUMAN |  | 7 | 1.03 | 0.007 | 16 | 0.1468 |
| **2262** | tr|M0QYT0|M0QYT0_HUMAN |  | 1 | 1.33 | 0.133 | 16 | 0.00252 |
| **2263** | sp|Q5SGD2|PPM1L_HUMAN |  | 1 | 0.99 | 0.1 | 16 | 0.8785 |
| **2264** | tr|B4DRI0|B4DRI0_HUMAN |  | 2 | 0.87 | 0.024 | 16 | 0.004131 |
| **2265** | tr|A8K901|A8K901_HUMAN |  | 2 | 0.85 | 0.033 | 16 | 0.005827 |
| **2266** | tr|Q9NWE9|Q9NWE9_HUMAN |  | 2 | 0.98 | 0.014 | 16 | 0.4705 |
| **2267** | tr|B2RDE8|B2RDE8_HUMAN |  | 2 | 1.43 | 0.042 | 16 | 3.99E-07 |
| **2268** | tr|E5RIM3|E5RIM3_HUMAN |  | 5 | 1.06 | 0.032 | 16 | 0.2027 |
| **2269** | tr|A0A087WW43|A0A087WW43_HUMAN |  | 3 | 1.07 | 0.066 | 16 | 0.2643 |
| **2270** | tr|K7ENX8|K7ENX8_HUMAN |  | 2 | 1 | 0.022 | 16 | 0.8977 |
| **2271** | sp|Q86T82|UBP37_HUMAN |  | 1 | 1.06 | 0.008 | 16 | 0.01305 |
| **2272** | sp|Q8WTU0|DDI1_HUMAN |  | 1 | 1.09 | 0.018 | 16 | 0.01904 |
| **2273** | sp|P23528|COF1_HUMAN |  | 7 | 1.02 | 0.057 | 16 | 0.7228 |
| **2274** | sp|Q9NR50|EI2BG_HUMAN |  | 1 | 0.88 | 0.005 | 16 | 4.07E-06 |
| **2275** | tr|Q9Y4A1|Q9Y4A1_HUMAN |  | 1 | 1.09 | 0.032 | 16 | 0.0629 |
| **2276** | tr|A0A024R9Q1|A0A024R9Q1_HUMAN |  | 23 | 0.94 | 0.009 | 16 | 0.02234 |
| **2277** | tr|Q86UL7|Q86UL7_HUMAN |  | 1 | 1.21 | 0.198 | 16 | 0.07652 |
| **2278** | sp|Q9Y4G6|TLN2_HUMAN |  | 18 | 0.91 | 0.006 | 16 | 0.0003829 |
| **2279** | tr|F5H8K3|F5H8K3_HUMAN |  | 1 | 1.04 | 0.037 | 16 | 0.4454 |
| **2280** | sp|Q9BS26|ERP44_HUMAN |  | 1 | 0.98 | 0.038 | 16 | 0.6441 |
| **2281** | tr|D3DWB6|D3DWB6_HUMAN |  | 24 | 0.87 | 0.001 | 16 | 7.10E-11 |
| **2282** | sp|P36507|MP2K2_HUMAN |  | 1 | 0.95 | 0.046 | 16 | 0.3275 |
| **2283** | tr|J3KT55|J3KT55_HUMAN |  | 1 | 1.49 | 0.143 | 16 | 0.0001223 |
| **2284** | tr|B4DTT4|B4DTT4_HUMAN |  | 3 | 1.06 | 0.028 | 16 | 0.1492 |
| **2285** | sp|P15169|CBPN_HUMAN |  | 2 | 1.29 | 0.018 | 16 | 3.60E-07 |
| **2286** | tr|A0A068LKQ0|A0A068LKQ0_HUMAN |  | 1 | 1.11 | 0.062 | 16 | 0.1002 |
| **2287** | sp|Q9NY65|TBA8_HUMAN |  | 2 | 0.91 | 0.007 | 16 | 0.000369 |
| **2288** | tr|O95408|O95408_HUMAN |  | 1 | 0.88 | 0.016 | 16 | 0.001924 |
| **2289** | sp|Q9UDY4|DNJB4_HUMAN |  | 3 | 1.02 | 0.006 | 16 | 0.4192 |
| **2290** | tr|E9RKB4|E9RKB4_HUMAN |  | 3 | 1.2 | 0.018 | 16 | 2.59E-05 |
| **2291** | sp|P41218|MNDA_HUMAN |  | 4 | 1.79 | 0.058 | 16 | 1.25E-09 |
| **2292** | sp|Q71U36|TBA1A_HUMAN |  | 1 | 0.95 | 0.031 | 16 | 0.2821 |
| **2293** | sp|Q7Z6K5|ARPIN_HUMAN |  | 1 | 1.1 | 0.108 | 16 | 0.2458 |
| **2294** | tr|J3KN29|J3KN29_HUMAN |  | 5 | 0.88 | 0.009 | 16 | 9.69E-05 |
| **2295** | tr|M0R3C8|M0R3C8_HUMAN |  | 1 | 1.26 | 0.24 | 16 | 0.05122 |
| **2296** | sp|Q92626|PXDN_HUMAN |  | 1 | 1.11 | 0.028 | 16 | 0.01712 |
| **2297** | sp|P35609|ACTN2_HUMAN |  | 2 | 1.08 | 0.015 | 16 | 0.02162 |
| **2298** | sp|Q8TC07|TBC15_HUMAN |  | 1 | 0.96 | 0.146 | 16 | 0.6817 |
| **2299** | tr|B4E1E2|B4E1E2_HUMAN |  | 10 | 0.99 | 0.005 | 16 | 0.5739 |
| **2300** | tr|B2RDR2|B2RDR2_HUMAN |  | 5 | 1.13 | 0.016 | 16 | 0.0009221 |
| **2301** | tr|Q7Z612|Q7Z612_HUMAN |  | 1 | 0.84 | 0.108 | 16 | 0.07472 |
| **2302** | sp|P54725|RD23A_HUMAN |  | 3 | 1.07 | 0.008 | 16 | 0.00707 |
| **2303** | tr|A0A090N7U2|A0A090N7U2_HUMAN |  | 5 | 0.92 | 0.014 | 16 | 0.01834 |
| **2304** | tr|B4DXW1|B4DXW1_HUMAN |  | 7 | 1.02 | 0.005 | 16 | 0.3805 |
| **2305** | tr|S4R3G2|S4R3G2_HUMAN |  | 1 | 1.33 | 0.015 | 16 | 2.38E-08 |
| **2306** | sp|P01768|HV307_HUMAN |  | 1 | 0.81 | 0.037 | 16 | 0.001137 |
| **2307** | sp|P16403|H12_HUMAN |  | 2 | 1.44 | 0.051 | 16 | 1.31E-06 |
| **2308** | tr|J3KSR7|J3KSR7_HUMAN |  | 1 | 0.73 | 0.015 | 16 | 3.47E-07 |
| **2309** | sp|O43633|CHM2A_HUMAN |  | 6 | 0.93 | 0.002 | 16 | 2.50E-06 |
| **2310** | tr|B3KMW4|B3KMW4_HUMAN |  | 3 | 1.02 | 0.009 | 16 | 0.3209 |
| **2311** | tr|K7ENK9|K7ENK9_HUMAN |  | 1 | 1.06 | 0.105 | 16 | 0.4661 |
| **2312** | sp|P07225|PROS_HUMAN |  | 1 | 0.93 | 0.012 | 16 | 0.01772 |
| **2313** | tr|B7Z2C6|B7Z2C6_HUMAN |  | 4 | 0.92 | 0.006 | 16 | 0.0007459 |
| **2314** | tr|R4GN98|R4GN98_HUMAN |  | 2 | 1.17 | 0.045 | 16 | 0.005322 |
| **2315** | sp|P00742|FA10_HUMAN |  | 3 | 0.99 | 0.036 | 16 | 0.8608 |
| **2316** | tr|Q53GU8|Q53GU8_HUMAN |  | 6 | 0.97 | 0.009 | 16 | 0.2144 |
| **2317** | tr|A0N5G3|A0N5G3_HUMAN |  | 1 | 1.01 | 0.036 | 16 | 0.7724 |
| **2318** | sp|P01615|KV202_HUMAN |  | 1 | 1.14 | 0.069 | 16 | 0.04359 |
| **2319** | tr|E9PQL5|E9PQL5_HUMAN |  | 1 | 1.41 | 0.179 | 16 | 0.001448 |
| **2320** | tr|Q59EM6|Q59EM6_HUMAN |  | 1 | 0.77 | 0.026 | 16 | 4.66E-05 |
| **2321** | tr|Q8IWP6|Q8IWP6_HUMAN |  | 3 | 0.92 | 0.005 | 16 | 0.0002441 |
| **2322** | tr|U3KQS8|U3KQS8_HUMAN |  | 1 | 1.31 | 0.133 | 16 | 0.003601 |
| **2323** | tr|K7EIJ0|K7EIJ0_HUMAN |  | 6 | 1.09 | 0.011 | 16 | 0.004565 |
| **2324** | tr|H7C3X5|H7C3X5_HUMAN |  | 2 | 0.95 | 0.013 | 16 | 0.1228 |
| **2325** | tr|B3KQF5|B3KQF5_HUMAN |  | 3 | 1.11 | 0.009 | 16 | 0.0003029 |
| **2326** | tr|A0A024R1T5|A0A024R1T5_HUMAN |  | 6 | 1.02 | 0.017 | 16 | 0.5791 |
| **2327** | tr|H3BT57|H3BT57_HUMAN |  | 2 | 0.73 | 0.053 | 16 | 0.0003172 |
| **2328** | tr|A0A024R462|A0A024R462_HUMAN |  | 47 | 1.01 | 0.017 | 16 | 0.6648 |
| **2329** | tr|H3BSW0|H3BSW0_HUMAN |  | 4 | 0.93 | 0.011 | 16 | 0.01631 |
| **2330** | tr|Q9H7J2|Q9H7J2_HUMAN |  | 3 | 0.91 | 0.02 | 16 | 0.02081 |
| **2331** | tr|B7Z871|B7Z871_HUMAN |  | 1 | 0.78 | 0.155 | 13 | 0.06149 |
| **2332** | tr|H7C3R6|H7C3R6_HUMAN |  | 2 | 1.06 | 0.014 | 16 | 0.07381 |
| **2333** | sp|P38606|VATA_HUMAN |  | 21 | 1.01 | 0.004 | 16 | 0.6958 |
| **2334** | tr|B5BUD5|B5BUD5_HUMAN |  | 6 | 1.11 | 0.014 | 16 | 0.00176 |
| **2335** | sp|P09104|ENOG_HUMAN |  | 4 | 1.03 | 0.022 | 16 | 0.4018 |
| **2336** | tr|B2R5M8|B2R5M8_HUMAN |  | 6 | 0.86 | 0.024 | 16 | 0.002457 |
| **2337** | tr|A0A024RBT8|A0A024RBT8_HUMAN |  | 1 | 0.77 | 0.01 | 16 | 1.12E-07 |
| **2338** | tr|V9GY58|V9GY58_HUMAN |  | 1 | 1.12 | 0.279 | 15 | 0.3837 |
| **2339** | tr|A7MAE2|A7MAE2_HUMAN |  | 2 | 0.92 | 0.083 | 16 | 0.2636 |
| **2340** | tr|A6PW57|A6PW57_HUMAN |  | 2 | 1.03 | 0.018 | 16 | 0.4184 |
| **2341** | tr|K7EIY6|K7EIY6_HUMAN |  | 2 | 0.99 | 0.022 | 16 | 0.7716 |
| **2342** | sp|O95786|DDX58_HUMAN |  | 1 | 1.14 | 0.035 | 16 | 0.0103 |
| **2343** | sp|Q9UBV8|PEF1_HUMAN |  | 5 | 1.01 | 0.004 | 16 | 0.6554 |
| **2344** | tr|B4DQ92|B4DQ92_HUMAN |  | 1 | 1.01 | 0.001 | 16 | 0.3946 |
| **2345** | tr|H7C3J5|H7C3J5_HUMAN |  | 1 | 1.18 | 0.043 | 16 | 0.003314 |
| **2346** | tr|E9PIR9|E9PIR9_HUMAN |  | 1 | 1.1 | 0.03 | 16 | 0.03866 |
| **2347** | sp|P11021|GRP78_HUMAN |  | 10 | 1.19 | 0.007 | 16 | 8.57E-08 |
| **2348** | tr|B2RCW0|B2RCW0_HUMAN |  | 2 | 0.91 | 0.017 | 16 | 0.01061 |
| **2349** | sp|Q9P0J7|KCMF1_HUMAN |  | 3 | 0.94 | 0.02 | 16 | 0.1042 |
| **2350** | sp|P01606|KV114_HUMAN |  | 1 | 0.97 | 0.023 | 16 | 0.4332 |
| **2351** | tr|B7Z2B4|B7Z2B4_HUMAN |  | 1 | 0.84 | 0.016 | 16 | 0.0002022 |
| **2352** | sp|Q9Y3Q8|T22D4_HUMAN |  | 5 | 0.96 | 0.008 | 16 | 0.1461 |
| **2353** | sp|Q9NRF8|PYRG2_HUMAN |  | 4 | 1 | 0.014 | 16 | 0.91 |
| **2354** | tr|Q99557|Q99557_HUMAN |  | 1 | 1.64 | 0.644 | 16 | 0.006268 |
| **2355** | sp|Q9UL15|BAG5_HUMAN |  | 3 | 1.08 | 0.069 | 16 | 0.2538 |
| **2356** | tr|B4DNJ5|B4DNJ5_HUMAN |  | 1 | 1.08 | 0.146 | 16 | 0.4427 |
| **2357** | tr|F8WDP8|F8WDP8_HUMAN |  | 1 | 0.9 | 0.007 | 16 | 0.0002784 |
| **2358** | tr|B4DW31|B4DW31_HUMAN |  | 6 | 1.01 | 0.004 | 16 | 0.7078 |
| **2359** | tr|A2J1N4|A2J1N4_HUMAN |  | 1 | 0.99 | 0.074 | 16 | 0.8616 |
| **2360** | tr|Q59GP7|Q59GP7_HUMAN |  | 2 | 1.28 | 0.042 | 16 | 6.86E-05 |
| **2361** | tr|B4DXP9|B4DXP9_HUMAN |  | 1 | 1.02 | 0.099 | 16 | 0.7936 |
| **2362** | tr|B3KVK6|B3KVK6_HUMAN |  | 2 | 0.99 | 0.008 | 16 | 0.6945 |
| **2363** | sp|Q0VDF9|HSP7E_HUMAN |  | 1 | 1.87 | 3.29 | 16 | 0.0755 |
| **2364** | tr|B6VEX5|B6VEX5_HUMAN |  | 1 | 0.96 | 0.023 | 16 | 0.3347 |
| **2365** | tr|Q0ZCF6|Q0ZCF6_HUMAN |  | 1 | 1.05 | 0.041 | 16 | 0.3882 |
| **2366** | tr|B1AKG0|B1AKG0_HUMAN |  | 4 | 1.08 | 0.022 | 16 | 0.04199 |
| **2367** | sp|P45974|UBP5_HUMAN |  | 25 | 1.09 | 0.002 | 16 | 2.10E-07 |
| **2368** | tr|J3KSP1|J3KSP1_HUMAN |  | 1 | 1.21 | 0.089 | 16 | 0.01381 |
| **2369** | tr|Q003U9|Q003U9_HUMAN |  | 1 | 0.93 | 0.041 | 16 | 0.1756 |
| **2370** | tr|D6RC49|D6RC49_HUMAN |  | 1 | 1.28 | 0.055 | 16 | 0.0002234 |
| **2371** | sp|Q5VW36|FOCAD_HUMAN |  | 1 | 0.85 | 0.02 | 16 | 0.0007531 |
| **2372** | tr|H3BV23|H3BV23_HUMAN |  | 1 | 1.1 | 0.033 | 16 | 0.04009 |
| **2373** | sp|P13727|PRG2_HUMAN |  | 1 | 3.9 | 5.886 | 16 | 0.0002465 |
| **2374** | tr|K7ENH3|K7ENH3_HUMAN |  | 1 | 0.98 | 0.032 | 16 | 0.5977 |
| **2375** | tr|H7BXS9|H7BXS9_HUMAN |  | 1 | 2.22 | 7.523 | 16 | 0.09465 |
| **2376** | tr|F8VRR3|F8VRR3_HUMAN |  | 4 | 0.87 | 0.016 | 16 | 0.0006424 |
| **2377** | tr|A5D904|A5D904_HUMAN |  | 3 | 0.85 | 0.018 | 16 | 0.0003484 |
| **2378** | tr|B3KRR1|B3KRR1_HUMAN |  | 1 | 0.67 | 0.008 | 16 | 2.02E-10 |
| **2379** | tr|V9HWC6|V9HWC6_HUMAN |  | 1 | 1.42 | 0.063 | 16 | 7.33E-06 |
| **2380** | sp|Q9NTJ4|MA2C1_HUMAN |  | 7 | 1 | 0.011 | 16 | 0.9132 |
| **2381** | tr|J3QRP6|J3QRP6_HUMAN |  | 1 | 3.38 | 13.89 | 16 | 0.02219 |
| **2382** | tr|B4DT77|B4DT77_HUMAN |  | 14 | 1.02 | 0.012 | 16 | 0.5828 |
| **2383** | tr|A8K646|A8K646_HUMAN |  | 2 | 1.27 | 0.011 | 16 | 3.30E-08 |
| **2384** | sp|Q13576|IQGA2_HUMAN |  | 18 | 0.98 | 0.006 | 16 | 0.2684 |
| **2385** | tr|J3KSR8|J3KSR8_HUMAN |  | 1 | 2.03 | 0.029 | 16 | 2.10E-13 |
| **2386** | tr|A5YAK2|A5YAK2_HUMAN |  | 2 | 1.12 | 0.136 | 16 | 0.1995 |
| **2387** | sp|Q9HCI5|MAGE1_HUMAN |  | 1 | 1.31 | 0.223 | 16 | 0.01791 |
| **2388** | sp|P48507|GSH0_HUMAN |  | 6 | 0.98 | 0.009 | 16 | 0.4829 |
| **2389** | tr|E9PRY0|E9PRY0_HUMAN |  | 1 | 0.94 | 0.075 | 16 | 0.3753 |

**Table S2 The list of up-regulated significant differentially expressed protein between high and low** **neuroticism by iTRAQ analysis**

| Protein_ID | **Description** | **Ratio** | ***P*** |
| --- | --- | --- | --- |
| tr|V9H0D6|V9H0D6_HUMAN | Complement C4A3 | 1.34 | 0.04492^**^ |
| tr|Q53T09|Q53T09_HUMAN | Putative uncharacterized protein XRCC5 | 1.55 | 2.17E-08^**^ |
| sp|Q00169|PIPNA_HUMAN | Epididymis secretory protein Li 36 | 1.21 | 0.005594^**^ |
| tr|W8QEY1|W8QEY1_HUMAN | Lactoferrin | 2.23 | 7.01E-06^**^ |
| tr|D6W5C0|D6W5C0_HUMAN | Spectrin, beta, non-erythrocytic 1, isoform CRA_b | 1.22 | 9.85E-07^**^ |
| tr|A0A096LP07|A0A096LP07_HUMAN | COP9 signalosome complex subunit 1 | 1.24 | 9.81E-06^**^ |
| tr|C9J8R4|C9J8R4_HUMAN | DCN1-like protein | 1.28 | 3.47E-06^**^ |
| tr|D6RF44|D6RF44_HUMAN | Heterogeneous nuclear ribonucleoprotein D0 | 1.49 | 1.75E-05^**^ |
| tr|K7EMV3|K7EMV3_HUMAN | Histone H3 | 2.17 | 5.39E-08^**^ |
| tr|Q7Z497|Q7Z497_HUMAN | SF3B1 protein | 1.35 | 0.0344^**^ |
| tr|Q9BXA2|Q9BXA2_HUMAN | Beta-globin | 1.45 | 2.33E-05^**^ |
| sp|P02774|VTDB_HUMAN | Epididymis secretory protein Li 51 | 1.29 | 6.31E-06^**^ |
| sp|Q9NR12|PDLI7_HUMAN | PDZ and LIM domain protein 7 | 1.29 | 0.006661^**^ |
| sp|P63104|1433Z_HUMAN | Epididymis luminal protein 4 | 1.26 | 4.16E-08^**^ |
| sp|P08311|CATG_HUMAN | Cathepsin G | 2.12 | 5.28E-10^**^ |
| sp|A8MVM7|YD021_HUMAN | Putative uncharacterized protein ENSP00000382790 | 1.26 | 0.03911^**^ |
| sp|P02647|APOA1_HUMAN | Apolipoprotein A-I, isoform CRA_a | 1.21 | 3.28E-06^**^ |
| sp|P02743|SAMP_HUMAN | Amyloid P component, serum | 1.42 | 1.23E-08^**^ |
| sp|P22792|CPN2_HUMAN | Carboxypeptidase N subunit 2 | 1.25 | 7.35E-09^**^ |
| tr|H3BN55|H3BN55_HUMAN | Ras-related protein Rab-27A | 1.25 | 3.10E-06^**^ |
| tr|H0YKU7|H0YKU7_HUMAN | Transient receptor potential cation channel subfamily M member 1 | 1.23 | 0.009705^**^ |
| tr|B4DMH3|B4DMH3_HUMAN | Coronin | 1.22 | 2.14E-06^**^ |
| tr|Q569I7|Q569I7_HUMAN | Uncharacterized protein | 1.31 | 0.005027^**^ |
| sp|P58397|ATS12_HUMAN | A disintegrin and metalloproteinase with thrombospondin motifs 12 | 1.8 | 0.005083^**^ |
| sp|Q52LW3|RHG29_HUMAN | Rho GTPase-activating protein 29 | 1.91 | 0.02874^**^ |
| tr|A2N0T3|A2N0T3_HUMAN | VH6DJ protein | 1.4 | 0.01061^**^ |
| sp|P11215|ITAM_HUMAN | Integrin alpha-M | 1.36 | 0.004657^**^ |
| tr|C9JJU7|C9JJU7_HUMAN | Protein FAM207A | 1.3 | 0.0001405^**^ |
| sp|P38919|IF4A3_HUMAN | DEAD (Asp-Glu-Ala-Asp) box polypeptide 48, isoform CRA_a | 1.22 | 0.00777^**^ |
| tr|A0A087X234|A0A087X234_HUMAN | Tubulin polyglutamylase TTLL7 | 4.07 | 0.006126^**^ |
| tr|Q8N1A3|Q8N1A3_HUMAN | Glycoprotein 2 (Zymogen granule membrane) | 1.28 | 9.96E-05^**^ |
| tr|Q6DC98|Q6DC98_HUMAN | LMNB1 protein | 1.44 | 4.50E-05^**^ |
| tr|F8VYN5|F8VYN5_HUMAN | Heterogeneous nuclear ribonucleoprotein A1 | 1.3 | 0.001368^**^ |
| sp|Q9BT73|PSMG3_HUMAN | Uncharacterized protein | 1.6 | 0.01756^**^ |
| tr|Q59HG6|Q59HG6_HUMAN | Rho GTPase activating protein 6 isoform 1 variant | 1.31 | 3.42E-05^**^ |
| tr|E7ER27|E7ER27_HUMAN | Peroxisomal multifunctional enzyme type 2 | 1.35 | 0.0065^**^ |
| tr|J3QLH1|J3QLH1_HUMAN | ARF GTPase-activating protein GIT1 | 1.25 | 0.003339^**^ |
| tr|Q53FW2|Q53FW2_HUMAN | Phosphoribosyl pyrophosphate synthetase 1 variant | 1.21 | 3.63E-10^**^ |
| tr|Q9BSE8|Q9BSE8_HUMAN | ZNF607 protein | 1.22 | 0.006071^**^ |
| sp|P62312|LSM6_HUMAN | U6 snRNA-associated Sm-like protein LSm6 | 1.26 | 0.00262^**^ |
| sp|Q9NY12|GAR1_HUMAN | Nucleolar protein family A, member 1 (H/ACA small nucleolar RNPs), isoform CRA_a | 1.55 | 1.59E-07^**^ |
| tr|H7C540|H7C540_HUMAN | Ribose-phosphate pyrophosphokinase 2 | 1.31 | 4.75E-10^**^ |
| tr|J3QLE5|J3QLE5_HUMAN | Small nuclear ribonucleoprotein-associated protein N | 1.88 | 1.49E-11^**^ |
| tr|A2VCT2|A2VCT2_HUMAN | OGDH protein | 1.27 | 0.0001319^**^ |
| tr|B3KWP7|B3KWP7_HUMAN | cDNA FLJ43538 fis, clone PLACE7008431, highly similar to Homo sapiens phosphatidylinositol-4-phosphate 5-kinase, type II, gamma (PIP5K2C), mRNA | 1.28 | 4.03E-05^**^ |
| tr|H0Y360|H0Y360_HUMAN | AMP deaminase 2 | 1.29 | 4.02E-05^**^ |
| tr|L7RSM2|L7RSM2_HUMAN | Mitogen-activated protein kinase 14 OS=Homo sapiens GN=MAPK14 PE=4 SV=1 | 1.23 | 5.68E-06^**^ |
| sp|P61626|LYSC_HUMAN | C-type lysozyme OS=Homo sapiens GN=LYZ PE=2 SV=1 | 1.33 | 4.75E-06^**^ |
| tr|B4E3A7|B4E3A7_HUMAN | cDNA FLJ52057, highly similar to Periostin | 1.21 | 0.005753^**^ |
| tr|H0YA27|H0YA27_HUMAN | Cyclin-I | 1.31 | 2.33E-06^**^ |
| sp|P11678|PERE_HUMAN | Eosinophil peroxidase | 1.72 | 2.99E-06^**^ |
| sp|P62304|RUXE_HUMAN | Small nuclear ribonucleoprotein E | 1.39 | 0.0004667^**^ |
| tr|Q8WVW5|Q8WVW5_HUMAN | Putative uncharacterized protein | 1.22 | 3.69E-06^**^ |
| tr|Q9UK54|Q9UK54_HUMAN | Hemoglobin beta subunit variant | 1.47 | 0.0001257^**^ |
| tr|E9PJX0|E9PJX0_HUMAN | Transmembrane protein 135 | 1.56 | 3.51E-05^**^ |
| sp|P62805|H4_HUMAN | Histone H4 | 4.07 | 7.67E-13^**^ |
| tr|B2R5G8|B2R5G8_HUMAN | Serum amyloid A protein | 1.28 | 0.0007963^**^ |
| tr|H7C2Z6|H7C2Z6_HUMAN | Grancalcin | 1.36 | 0.001264^**^ |
| tr|E7EMC6|E7EMC6_HUMAN | Annexin | 1.41 | 1.54E-05^**^ |
| tr|A0PJ62|A0PJ62_HUMAN | RPL14 protein | 1.21 | 0.005595^**^ |
| sp|Q8IXQ3|CI040_HUMAN | Uncharacterized protein C9orf40 | 1.48 | 1.77E-05^**^ |
| tr|Q2HIY3|Q2HIY3_HUMAN | RNF130 protein | 1.27 | 0.0004426^**^ |
| tr|B4E397|B4E397_HUMAN | cDNA FLJ55140, highly similar to SPARC-like protein 1 | 1.21 | 0.001182^**^ |
| sp|O94875|SRBS2_HUMAN | Sorbin and SH3 domain-containing protein 2 | 1.24 | 8.49E-07^**^ |
| sp|Q4G0X9|CCD40_HUMAN | Coiled-coil domain-containing protein 40 | 1.21 | 0.0004823^**^ |
| tr|B1AH77|B1AH77_HUMAN | Ras-related C3 botulinum toxin substrate 2 | 1.21 | 0.0006837^**^ |
| tr|Q5TEC6|Q5TEC6_HUMAN | Histone H3 | 2.13 | 4.72E-06^**^ |
| tr|A0A087X1J7|A0A087X1J7_HUMAN | Glutathione peroxidase | 1.21 | 2.64E-07^**^ |
| tr|B0YJC4|B0YJC4_HUMAN | Vimentin | 2.02 | 2.36E-14^**^ |
| tr|C9JGV7|C9JGV7_HUMAN | Dapper homolog 1 | 4.18 | 0.003618^**^ |
| tr|B4DXG0|B4DXG0_HUMAN | cDNA FLJ57651, highly similar to Ketosamine-3-kinase | 1.22 | 0.0004771^**^ |
| tr|Q5JQ44|Q5JQ44_HUMAN | Putative uncharacterized protein DKFZp547A0616 | 1.24 | 0.0002629^**^ |
| sp|Q92522|H1X_HUMAN | Histone H1x | 2.56 | 2.55E-09^**^ |
| tr|F8WFC3|F8WFC3_HUMAN | Regulator of microtubule dynamics protein 2 | 1.29 | 1.72E-05^**^ |
| tr|Q5STU3|Q5STU3_HUMAN | Spliceosome RNA helicase DDX39B | 1.31 | 5.49E-09^**^ |
| sp|Q15746|MYLK_HUMAN | Myosin light chain kinase, smooth muscle | 1.26 | 0.001419^**^ |
| tr|Q0ZCH4|Q0ZCH4_HUMAN | Immunglobulin heavy chain variable region | 1.26 | 0.0008512^**^ |
| tr|H7BZJ3|H7BZJ3_HUMAN | Protein disulfide-isomerase A3 | 1.29 | 0.0006466^**^ |
| sp|P20160|CAP7_HUMAN | Azurocidin | 1.83 | 1.11E-08^**^ |
| sp|Q99598|TSNAX_HUMAN | Translin-associated factor X, isoform CRA_c | 1.21 | 5.17E-06^**^ |
| tr|B4DXF1|B4DXF1_HUMAN | cDNA FLJ57223 | 1.44 | 4.86E-05^**^ |
| sp|O00299|CLIC1_HUMAN | Chloride intracellular channel 1, isoform CRA_a | 1.33 | 2.98E-07^**^ |
| sp|P06727|APOA4_HUMAN | Apolipoprotein A-IV | 1.29 | 8.18E-07^**^ |
| sp|Q9NYB0|TE2IP_HUMAN | Telomeric repeat-binding factor 2-interacting protein 1 | 1.79 | 0.02098^**^ |
| tr|B4DPN0|B4DPN0_HUMAN | cDNA FLJ51265, moderately similar to Beta-2-glycoprotein 1 (Beta-2-glycoprotein I) | 1.21 | 0.0008805^**^ |
| tr|M0R2L9|M0R2L9_HUMAN | 40S ribosomal protein S19 | 1.21 | 0.0401^**^ |
| sp|P05164|PERM_HUMAN | Myeloperoxidase | 1.74 | 3.18E-10^**^ |
| sp|Q86YV6|MYLK4_HUMAN | Myosin light chain kinase family member 4 | 1.23 | 1.47E-06^**^ |
| tr|E9PK89|E9PK89_HUMAN | Guanine nucleotide exchange factor for Rab-3A | 1.5 | 0.02262^**^ |
| sp|Q9UKK9|NUDT5_HUMAN | ADP-sugar pyrophosphatase | 1.5 | 1.04E-08^**^ |
| tr|A2J1N6|A2J1N6_HUMAN | Rheumatoid factor RF-ET9 | 1.23 | 0.03918^**^ |
| tr|Q7Z4Q5|Q7Z4Q5_HUMAN | Heterogeneous nuclear ribonucleoprotein U (Scaffold attachment factor A), isoform CRA_a | 1.3 | 2.24E-05^**^ |
| tr|D6RAC8|D6RAC8_HUMAN | Matrix extracellular phosphoglycoprotein | 1.43 | 0.0001371^**^ |
| sp|Q96KK5|H2A1H_HUMAN | Histone H2A | 2.74 | 3.27E-11^**^ |
| tr|Q53FR6|Q53FR6_HUMAN | Cartilage oligomeric matrix protein variant | 1.63 | 0.02135^**^ |
| tr|B2R960|B2R960_HUMAN | cDNA, FLJ94230, highly similar to Homo sapiens thioredoxin-like 1 (TXNL1), mRNA | 1.23 | 5.76E-06^**^ |
| tr|B2MUD5|B2MUD5_HUMAN | Neutrophil elastase | 2.05 | 3.34E-09^**^ |
| sp|P01703|LV105_HUMAN | Ig lambda chain V-I region NEWM | 1.26 | 0.005752^**^ |
| sp|P61970|NTF2_HUMAN | Nuclear transport factor 2, isoform CRA_a | 1.26 | 5.83E-05^**^ |
| tr|Q0IJ56|Q0IJ56_HUMAN | ST13 protein | 1.41 | 2.29E-05^**^ |
| tr|Q6PIX2|Q6PIX2_HUMAN | SFPQ protein | 1.39 | 6.36E-05^**^ |
| sp|P62306|RUXF_HUMAN | Small nuclear ribonucleoprotein F | 1.33 | 5.13E-05^**^ |
| tr|U3KQ84|U3KQ84_HUMAN | Dolichyl-diphosphooligosaccharide--protein glycosyltransferase 48 kDa subunit | 1.3 | 1.75E-07^**^ |
| tr|B2RBP3|B2RBP3_HUMAN | cDNA, FLJ95615, highly similar to Homo sapiens ubiquitin-activating enzyme E1C (UBA3 homolog, yeast)(UBE1C), mRNA | 1.21 | 4.84E-06^**^ |
| tr|B4E356|B4E356_HUMAN | cDNA FLJ54993, highly similar to ATP-dependent DNA helicase 2 subunit 1 (EC 3.6.1.-) | 1.49 | 9.59E-12^**^ |
| sp|P62318|SMD3_HUMAN | Small nuclear ribonucleoprotein Sm D3 | 1.26 | 0.0003359^**^ |
| tr|A0A096LPF0|A0A096LPF0_HUMAN | Ras-related protein Rab-7b | 1.24 | 0.0003839^**^ |
| sp|Q13185|CBX3_HUMAN | Chromobox homolog 3 (HP1 gamma homolog, Drosophila) | 1.38 | 0.0003439^**^ |
| tr|B7Z3I9|B7Z3I9_HUMAN | Delta-aminolevulinic acid dehydratase | 1.24 | 6.73E-10^**^ |
| tr|A2NX48|A2NX48_HUMAN | Bactericidal /Permeability Increasing Protein | 1.79 | 6.88E-07^**^ |
| sp|Q9NVW2|RNF12_HUMAN | E3 ubiquitin-protein ligase RLIM | 1.35 | 0.03508^**^ |
| tr|D6RBG2|D6RBG2_HUMAN | Protocadherin-1 | 1.25 | 0.01108^**^ |
| tr|F8W1I5|F8W1I5_HUMAN | Myosin light chain 6B | 1.37 | 1.01E-10^**^ |
| tr|H0Y7N2|H0Y7N2_HUMAN | Ataxin-10 | 1.28 | 0.002059^**^ |
| sp|P12724|ECP_HUMAN | Eosinophil cationic protein | 1.86 | 5.13E-05^**^ |
| tr|B3KP61|B3KP61_HUMAN | cDNA FLJ31217 fis, clone KIDNE2004095, highly similar to RAC-beta serine/threonine-protein kinase (EC 2.7.11.1) | 1.64 | 0.01661^**^ |
| sp|P07900|HS90A_HUMAN | Epididymis luminal secretory protein 52 | 1.21 | 3.40E-06^**^ |
| sp|P07237|PDIA1_HUMAN | Protein disulfide-isomerase | 1.51 | 1.72E-08^**^ |
| tr|B3KX15|B3KX15_HUMAN | cDNA FLJ44468 fis, clone UTERU2026025, moderately similar to SPLICING FACTOR, ARGININE/SERINE-RICH 2 | 1.51 | 0.0008793^**^ |
| sp|Q8NFR7|CC148_HUMAN | Coiled-coil domain-containing protein 148 | 2.79 | 0.005182^**^ |
| tr|F2Z2Y6|F2Z2Y6_HUMAN | U6 snRNA-associated Sm-like protein LSm8 | 1.34 | 3.82E-07^**^ |
| sp|Q9NQH7|XPP3_HUMAN | Probable Xaa-Pro aminopeptidase 3 | 1.25 | 9.20E-08^**^ |
| tr|I6L957|I6L957_HUMAN | HNRNPA2B1 protein | 1.8 | 1.54E-05^**^ |
| sp|Q9Y6I8|PXMP4_HUMAN | Peroxisomal membrane protein 4 | 3.3 | 0.008293^**^ |
| sp|Q9UMX5|NENF_HUMAN | Neudesin | 6.6 | 3.39E-05^**^ |
| sp|Q9HD89|RETN_HUMAN | Resistin | 1.84 | 0.000817^**^ |
| tr|I7HJJ0|I7HJJ0_HUMAN | ADP/ATP translocase 3 | 1.26 | 0.0002552^**^ |
| sp|P63208|SKP1_HUMAN | S-phase kinase-associated protein 1 | 1.22 | 1.42E-05^**^ |
| sp|O15511|ARPC5_HUMAN | Actin-related protein 2/3 complex subunit 5 | 1.25 | 2.26E-06^**^ |
| tr|E7EQB2|E7EQB2_HUMAN | Lactotransferrin | 3.26 | 3.73E-07^**^ |
| tr|H0Y5R6|H0Y5R6_HUMAN | Uroporphyrinogen decarboxylase | 1.34 | 5.45E-05^**^ |
| sp|Q9Y5S9|RBM8A_HUMAN | RNA-binding protein 8A | 1.23 | 2.80E-08^**^ |
| tr|B4DW89|B4DW89_HUMAN | cDNA FLJ52785, highly similar to Protein cereblon | 2.09 | 2.09E-06^**^ |
| tr|Q4LE74|Q4LE74_HUMAN | MYO9B variant protein | 1.24 | 0.000296^**^ |
| tr|B4E0N9|B4E0N9_HUMAN | Glutamate dehydrogenase | 2.02 | 0.0071^**^ |
| tr|Q5T0D2|Q5T0D2_HUMAN | UMP-CMP kinase | 1.27 | 0.0008348^**^ |
| tr|F8WDD7|F8WDD7_HUMAN | Actin-related protein 2/3 complex subunit 4 | 1.27 | 0.002728^**^ |
| sp|P04438|HV208_HUMAN | Ig heavy chain V-II region SESS | 1.68 | 2.01E-05^**^ |
| tr|Q53FG4|Q53FG4_HUMAN | Neutrophil cytosolic factor 4 (40kD) isoform 1 variant | 1.37 | 0.0001369^**^ |
| sp|P14780|MMP9_HUMAN | Matrix metalloproteinase-9 | 1.87 | 1.61E-07^**^ |
| sp|P29144|TPP2_HUMAN | Tripeptidyl-peptidase 2 | 1.21 | 0.0003016^**^ |
| sp|P01605|KV113_HUMAN | Ig kappa chain V-I region Lay | 1.21 | 0.006895^**^ |
| tr|H3BQD4|H3BQD4_HUMAN | Ribosome biogenesis protein TSR3 homolog | 1.46 | 4.97E-05^**^ |
| tr|Q762B6|Q762B6_HUMAN | ATP7A protein | 1.34 | 0.000539^**^ |
| tr|B3KPF2|B3KPF2_HUMAN | cDNA FLJ31717 fis, clone NT2RI2006640, highly similar to Dynamin-3 (EC 3.6.5.5) | 1.5 | 0.008923^**^ |
| tr|U3KPS2|U3KPS2_HUMAN | Myeloblastin | 2.01 | 1.98E-08^**^ |
| tr|E9PJP1|E9PJP1_HUMAN | Mth938 domain-containing protein | 1.27 | 0.0004009^**^ |
| tr|B4DV64|B4DV64_HUMAN | cDNA FLJ61420, highly similar to Homo sapiens olfactomedin 4 (OLFM4), mRNA | 1.54 | 1.07E-06^**^ |
| sp|P48426|PI42A_HUMAN | Phosphatidylinositol 5-phosphate 4-kinase type-2 alpha | 1.21 | 6.73E-08^**^ |
| sp|Q93079|H2B1H_HUMAN | Histone H2B type 1-H | 4.31 | 3.00E-14^**^ |
| tr|B4E366|B4E366_HUMAN | cDNA FLJ57781, highly similar to Homo sapiens tRNA-histidine guanylyltransferase 1-like (THG1L), mRNA | 1.24 | 5.13E-05^**^ |
| tr|O15302|O15302_HUMAN | CaM kinase II isoform | 1.3 | 0.009293^**^ |
| tr|I3L0V5|I3L0V5_HUMAN | A-kinase anchor protein 1, mitochondrial | 1.22 | 0.0005106^**^ |
| sp|P01011|AACT_HUMAN | Serpin peptidase inhibitor, clade A (Alpha-1 antiproteinase, antitrypsin), member 3, isoform CRA_c | 1.57 | 2.20E-09^**^ |
| tr|B3KSJ4|B3KSJ4_HUMAN | cDNA FLJ36421 fis, clone THYMU2011259, highly similar to Adapter-related protein complex 4 beta 1 subunit | 1.52 | 1.44E-06^**^ |
| tr|S6BGD4|S6BGD4_HUMAN | IgG H chain | 1.37 | 0.0001422^**^ |
| sp|P01880|IGHD_HUMAN | Ig delta chain C region | 1.25 | 0.0003251^**^ |
| sp|Q712K3|UB2R2_HUMAN | Ubiquitin-conjugating enzyme E2 R2 | 1.37 | 0.008864^**^ |
| sp|P35542|SAA4_HUMAN | Serum amyloid A-4 protein | 1.24 | 0.001118^**^ |
| tr|Q86UW0|Q86UW0_HUMAN | Ovarian epithelial carcinoma-related protein | 1.8 | 1.49E-06^**^ |
| tr|B4DGW3|B4DGW3_HUMAN | cDNA FLJ54625, highly similar to ATP synthase subunit alpha, mitochondrial (EC 3.6.3.14) | 1.21 | 1.30E-07^**^ |
| sp|Q01432|AMPD3_HUMAN | AMP deaminase 3 | 1.28 | 1.82E-08^**^ |
| sp|Q14644|RASA3_HUMAN | Ras GTPase-activating protein 3 | 1.53 | 2.85E-06^**^ |
| tr|B7Z5Q2|B7Z5Q2_HUMAN | cDNA FLJ58075, highly similar to Ceruloplasmin (EC 1.16.3.1) | 1.69 | 2.34E-10^**^ |
| tr|Q14730|Q14730_HUMAN | La 4.1 protein | 1.35 | 4.98E-05^**^ |
| tr|F8VS29|F8VS29_HUMAN | Centrosomal protein of 290 kDa | 1.21 | 0.0009425^**^ |
| sp|O00602|FCN1_HUMAN | Ficolin-1 | 1.22 | 0.0006718^**^ |
| tr|H0Y786|H0Y786_HUMAN | Nebulin | 1.37 | 0.0002004^**^ |
| tr|B4DKL7|B4DKL7_HUMAN | Transmembrane protein 104 | 1.3 | 0.0005537^**^ |
| tr|B3KT02|B3KT02_HUMAN | cDNA FLJ37371 fis, clone BRAMY2024711, highly similar to PDZ domain-containing RING finger protein 4 | 1.55 | 0.0007711^**^ |
| tr|Q69YH0|Q69YH0_HUMAN | PRPF38B protein | 1.24 | 0.01411^**^ |
| sp|P23527|H2B1O_HUMAN | Histone H2B type 1-O | 3.29 | 7.91E-16^**^ |
| tr|L8E7U6|L8E7U6_HUMAN | Alternative protein ZNF192 | 1.25 | 0.03465^**^ |
| sp|Q96DL1|NXPE2_HUMAN | NXPE family member 2 | 1.75 | 0.0008355^**^ |
| tr|A0JLQ2|A0JLQ2_HUMAN | Kinesin-like protein | 1.48 | 0.001154^**^ |
| sp|Q9UM07|PADI4_HUMAN | Protein-arginine deiminase type-4 | 1.45 | 1.05E-07^**^ |
| sp|P84090|ERH_HUMAN | Enhancer of rudimentary homolog | 1.98 | 1.21E-07^**^ |
| sp|Q13287|NMI_HUMAN | N-myc-interactor | 1.34 | 0.009692^**^ |
| sp|P84095|RHOG_HUMAN | ARHG protein | 1.36 | 0.0003102^**^ |
| tr|B4DM63|B4DM63_HUMAN | Actin-related protein 2/3 complex subunit 3 | 1.28 | 3.82E-06^**^ |
| tr|B7Z2X4|B7Z2X4_HUMAN | cDNA FLJ53327, highly similar to Gelsolin | 1.25 | 2.11E-12^**^ |
| sp|Q8TDL5|BPIB1_HUMAN | BPI fold-containing family B member 1 | 1.23 | 0.0002438^**^ |
| tr|Q4W5L2|Q4W5L2_HUMAN | Alpha-synuclein | 1.4 | 1.30E-05^**^ |
| tr|E9PN45|E9PN45_HUMAN | Pleckstrin homology domain-containing family A member 7 | 1.25 | 0.02362^**^ |
| tr|A0A087WTK4|A0A087WTK4_HUMAN | Protein IGKV1-16 | 1.35 | 0.03819^**^ |
| sp|P04792|HSPB1_HUMAN | Epididymis secretory protein Li 102 | 1.33 | 0.003463^**^ |
| tr|A8K931|A8K931_HUMAN | cDNA FLJ78742 | 1.21 | 0.002627^**^ |
| tr|J9JIH1|J9JIH1_HUMAN | Cytosolic carboxypeptidase 2 | 1.51 | 0.01183^**^ |
| tr|E9PCB6|E9PCB6_HUMAN | Neurolysin, mitochondrial | 2.18 | 3.17E-06^**^ |
| tr|Q96K98|Q96K98_HUMAN | cDNA FLJ14414 fis, clone HEMBA1004847, highly similar to SIGNAL RECOGNITION PARTICLE 68 KD PROTEIN | 1.26 | 6.14E-06^**^ |
| sp|Q5SQ64|LY66F_HUMAN | Lymphocyte antigen 6 complex locus protein G6f | 1.46 | 0.006848^**^ |
| sp|Q8WZA0|LZIC_HUMAN | Leucine zipper and CTNNBIP1 domain containing, isoform CRA_a | 1.57 | 2.30E-06^**^ |
| sp|A0AVT1|UBA6_HUMAN | Ubiquitin-activating enzyme E1-like 2, isoform CRA_a | 1.25 | 8.47E-06^**^ |
| sp|O75367|H2AY_HUMAN | Core histone macro-H2A.1 | 1.22 | 0.0006141^**^ |
| tr|Q96CA8|Q96CA8_HUMAN | CDC2L2 protein | 1.29 | 0.0006709^**^ |
| sp|B1AK53|ESPN_HUMAN | Espin | 1.26 | 0.0002001^**^ |
| tr|B4DXV1|B4DXV1_HUMAN | cDNA FLJ58642, highly similar to Homo sapiens elongation protein 3 homolog (ELP3), mRNA | 1.25 | 0.0018^**^ |
| sp|O15144|ARPC2_HUMAN | Actin related protein 2/3 complex, subunit 2, 34kDa, isoform CRA_a | 1.23 | 1.59E-05^**^ |
| tr|K7EJL1|K7EJL1_HUMAN | AP-1 complex subunit mu-1 | 1.3 | 0.002403^**^ |
| sp|P59768|GBG2_HUMAN | Guanine nucleotide-binding protein G(I)/G(S)/G(O) subunit gamma-2 | 1.35 | 0.002955^**^ |
| tr|A0A087X2D0|A0A087X2D0_HUMAN | Serine/arginine-rich-splicing factor 3 | 1.29 | 3.60E-07^**^ |
| tr|Q5QPM0|Q5QPM0_HUMAN | RNA-binding protein Raly | 2.03 | 2.03E-06^**^ |
| tr|B4DE89|B4DE89_HUMAN | cDNA FLJ59472, highly similar to Tripeptidyl-peptidase 1 | 1.43 | 4.95E-06^**^ |
| sp|Q12765|SCRN1_HUMAN | Secernin 1 OS=Homo sapiens GN=SCRN1 PE=4 SV=1 | 1.22 | 0.004028^**^ |
| tr|B4DGH0|B4DGH0_HUMAN | cDNA FLJ60107, highly similar to DNA replication complex GINS protein PSF1 | 1.29 | 0.03971^**^ |
| sp|Q9H3P7|GCP60_HUMAN | Acyl-Coenzyme A binding domain containing 3, isoform CRA_a | 1.54 | 0.001426^**^ |
| tr|S6B2B6|S6B2B6_HUMAN | IgG H chain | 1.21 | 0.02172^**^ |
| tr|D3DP16|D3DP16_HUMAN | Fibrinogen gamma chain, isoform CRA_a | 1.54 | 0.00208^**^ |
| tr|B4DP62|B4DP62_HUMAN | Solute carrier family 25 (Mitochondrial carrier citrate transporter), member 1, isoform CRA_b | 2.61 | 0.02489^**^ |
| tr|I3L397|I3L397_HUMAN | Eukaryotic translation initiation factor 5A-1 | 1.42 | 8.63E-08^**^ |
| sp|Q9Y2Y8|PRG3_HUMAN | Proteoglycan 3 | 1.36 | 0.03724^**^ |
| tr|Q96LQ7|Q96LQ7_HUMAN | Cadherin-like 24, isoform CRA_a | 2.12 | 0.04235^**^ |
| tr|Q8N7A2|Q8N7A2_HUMAN | cDNA FLJ25868 fis, clone CBR02068 | 1.44 | 0.007857^**^ |
| sp|Q9GZP4|PITH1_HUMAN | PITH domain-containing protein 1 | 1.29 | 8.72E-09^**^ |
| tr|B4DKU9|B4DKU9_HUMAN | Coronin | 1.24 | 0.04683^**^ |
| tr|Q6LCG1|Q6LCG1_HUMAN | Fas-associating protein | 1.22 | 0.009045^**^ |
| sp|P59666|DEF3_HUMAN | Defensin, alpha 3, neutrophil-specific | 2.99 | 4.69E-05^**^ |
| tr|M0QYT0|M0QYT0_HUMAN | Uncharacterized protein | 1.33 | 0.00252^**^ |
| tr|B2RDE8|B2RDE8_HUMAN | cDNA, FLJ96580, highly similar to Homo sapiens hepatoma-derived growth factor (high-mobility group protein 1-like) (HDGF), mRNA | 1.43 | 3.99E-07^**^ |
| tr|J3KT55|J3KT55_HUMAN | Receptor-type tyrosine-protein phosphatase mu (Fragment) | 1.49 | 0.0001223^**^ |
| sp|P15169|CBPN_HUMAN | Carboxypeptidase N catalytic chain | 1.29 | 3.60E-07^**^ |
| sp|P41218|MNDA_HUMAN | Myeloid cell nuclear differentiation antigen, isoform CRA_a | 1.79 | 1.25E-09^**^ |
| tr|S4R3G2|S4R3G2_HUMAN | Tectonic-1 | 1.33 | 2.38E-08^**^ |
| sp|P16403|H12_HUMAN | Histone H1.2 | 1.44 | 1.31E-06^**^ |
| tr|E9PQL5|E9PQL5_HUMAN | Uncharacterized protein C11orf57 | 1.41 | 0.001448^**^ |
| tr|U3KQS8|U3KQS8_HUMAN | DNA-directed RNA polymerases I, II, and III subunit RPABC2 | 1.31 | 0.003601^**^ |
| tr|Q99557|Q99557_HUMAN | NF-IL6 | 1.64 | 0.006268^**^ |
| tr|Q59GP7|Q59GP7_HUMAN | Interleukin enhancer binding factor 3 isoform c variant | 1.28 | 6.86E-05^**^ |
| tr|J3KSP1|J3KSP1_HUMAN | Abhydrolase domain-containing protein 3 | 1.21 | 0.01381^**^ |
| tr|D6RC49|D6RC49_HUMAN | Short transient receptor potential channel 3 | 1.28 | 0.0002234^**^ |
| sp|P13727|PRG2_HUMAN | Bone marrow proteoglycan | 3.9 | 0.0002465^**^ |
| tr|V9HWC6|V9HWC6_HUMAN | Peptidyl-prolyl cis-trans isomerase | 1.42 | 7.33E-06^**^ |
| tr|J3QRP6|J3QRP6_HUMAN | Na(+)/H(+) exchange regulatory cofactor NHE-RF1 | 3.38 | 0.02219^**^ |
| tr|A8K646|A8K646_HUMAN | cDNA FLJ75699, highly similar to Homo sapiens osteoclast stimulating factor 1 (OSTF1), mRNA | 1.27 | 3.30E-08^**^ |
| tr|J3KSR8|J3KSR8_HUMAN | Serine/arginine-rich-splicing factor 1 | 2.03 | 2.10E-13^**^ |
| sp|Q9HCI5|MAGE1_HUMAN | Melanoma-associated antigen E1 | 1.31 | 0.01791^**^ |

**P<0.05;**P<0.01*

**Table S3 The list of down-regulated significant** differentially expressed protein between high and low neuroticism by iTRAQ analysis

| **Protein ID** | **Description** | **Ratio** | ***P*** |
| --- | --- | --- | --- |
| tr|X5D767|X5D767_HUMAN | Adenosine deaminase isoform A (Fragment) | 0.83 | 6.50E-05^**^ |
| sp|P29218|IMPA1_HUMAN | Inositol(Myo)-1(Or4)-monophosphatase 1, isoform CRA_a | 0.78 | 2.95E-07^**^ |
| tr|B3KX27|B3KX27_HUMAN | cDNA FLJ44542 fis, clone UTERU3005585, highly similar to Rhophilin-2 | 0.81 | 0.002299^**^ |
| tr|Q05BN7|Q05BN7_HUMAN | MAN2B2 protein | 0.77 | 1.10E-07^**^ |
| tr|B4DEW1|B4DEW1_HUMAN | Alternative protein FUT9 | 0.79 | 3.48E-05^**^ |
| sp|P01624|KV306_HUMAN | Ig kappa chain V-III region POM | 0.82 | 0.02184^**^ |
| tr|B4DHX4|B4DHX4_HUMAN | cDNA FLJ52902, highly similar to Rab GDP dissociation inhibitor alpha | 0.83 | 0.03721^**^ |
| tr|Q9H3K5|Q9H3K5_HUMAN | My017 protein | 0.74 | 0.002687^**^ |
| tr|H3BTA2|H3BTA2_HUMAN | Serine/threonine-protein phosphatase (Fragment) | 0.79 | 0.01856^**^ |
| sp|P11277|SPTB1_HUMAN | Spectrin beta chain, erythrocytic | 0.81 | 5.37E-06^**^ |
| tr|B1Q3B3|B1Q3B3_HUMAN | Ferritin (Fragment) | 0.78 | 0.0005802^**^ |
| tr|E5RFX8|E5RFX8_HUMAN | Cyclin-C (Fragment) | 0.8 | 7.20E-08^**^ |
| tr|O14724|O14724_HUMAN | Beta-spectrin (Fragment) | 0.61 | 4.39E-08^**^ |
| tr|Q9BTI6|Q9BTI6_HUMAN | FLOT2 protein | 0.79 | 0.001533^**^ |
| tr|A8KA24|A8KA24_HUMAN | cDNA FLJ78648, highly similar to Homo sapiens vacuolar protein sorting 54 (yeast) (VPS54), transcript variant 2, mRNA | 0.75 | 7.67E-05^**^ |
| tr|Q9H3Z3|Q9H3Z3_HUMAN | Uridine-cytidine kinase-like 1 (Fragment) | 0.83 | 0.003124^**^ |
| tr|A0A024RB67|A0A024RB67_HUMAN | Exportin, tRNA (Nuclear export receptor for tRNAs), isoform CRA_a | 0.77 | 4.01E-09^**^ |
| tr|E7EXB4|E7EXB4_HUMAN | 60 kDa heat shock protein, mitochondrial (Fragment) | 0.63 | 0.00104^**^ |
| sp|O95373|IPO7_HUMAN | Importin-7 | 0.8 | 2.89E-09^**^ |
| tr|B4DHN4|B4DHN4_HUMAN | cDNA FLJ61116 | 0.76 | 3.39E-06^**^ |
| tr|H0YDA1|H0YDA1_HUMAN | Nuclear pore complex protein Nup98-Nup96 (Fragment) | 0.78 | 5.27E-05^**^ |
| tr|S6BGD6|S6BGD6_HUMAN | IgG L chain | 0.82 | 0.0003996^**^ |
| sp|P62633|CNBP_HUMAN | Cellular nucleic acid-binding protein | 0.67 | 0.002125^**^ |
| tr|D6RBM3|D6RBM3_HUMAN | Caspase-6 subunit p18 | 0.83 | 0.0007742^**^ |
| sp|Q9NX55|HYPK_HUMAN | HCG2003792, isoform CRA_b | 0.79 | 8.89E-05^**^ |
| tr|B2R4X4|B2R4X4_HUMAN | cDNA, FLJ92255, highly similar to Homo sapiens ring finger protein 7 (RNF7), mRNA | 0.78 | 2.68E-05^**^ |
| tr|B3KNN3|B3KNN3_HUMAN | cDNA FLJ30033 fis, clone 3NB692001433, highly similar to HEAT repeat-containing protein 2 | 0.75 | 0.001043^**^ |
| sp|Q99733|NP1L4_HUMAN | Nucleosome assembly protein 1-like 4, isoform CRA_b | 0.83 | 1.51E-08^**^ |
| tr|H7BZI1|H7BZI1_HUMAN | Nucleobindin-1 (Fragment) | 0.83 | 8.49E-05^**^ |
| sp|Q15172|2A5A_HUMAN | Serine/threonine-protein phosphatase 2A 56 kDa regulatory subunit alpha isoform | 0.83 | 0.004951^**^ |
| tr|A0A024R674|A0A024R674_HUMAN | Spectrin, beta, erythrocytic (Includes spherocytosis, clinical type I), isoform CRA_e | 0.72 | 2.41E-07^**^ |
| sp|Q8TDY2|RBCC1_HUMAN | RB1-inducible coiled-coil protein 1 | 0.82 | 0.0001213^**^ |
| tr|H7C492|H7C492_HUMAN | Mitochondrial chaperone BCS1 (Fragment) | 0.79 | 0.000403^**^ |
| tr|B4E2V5|B4E2V5_HUMAN | cDNA FLJ52062, highly similar to Erythrocyte band 7 integral membrane protein | 0.82 | 0.0007149^**^ |
| sp|Q6UXB2|VCC1_HUMAN | VEGF coregulated chemokine 1 | 0.54 | 4.20E-07^**^ |
| sp|P53004|BIEA_HUMAN | Biliverdin reductase A | 0.8 | 0.0008497^**^ |
| tr|D6R9W4|D6R9W4_HUMAN | Drebrin (Fragment) | 0.83 | 4.96E-06^**^ |
| tr|Q05CK9|Q05CK9_HUMAN | SYNCRIP protein (Fragment) | 0.79 | 8.41E-09^**^ |
| tr|A0A0A0MTP3|A0A0A0MTP3_HUMAN | M-phase phosphoprotein 9 | 0.71 | 1.96E-06^**^ |
| sp|Q63HN8|RN213_HUMAN | E3 ubiquitin-protein ligase RNF213 | 0.79 | 0.0002068^**^ |
| tr|A2J1M8|A2J1M8_HUMAN | Rheumatoid factor RF-IP12 (Fragment) | 0.79 | 0.016^**^ |
| tr|K7ER46|K7ER46_HUMAN | Beclin-1 (Fragment) | 0.75 | 0.0003288^**^ |
| tr|E9PI78|E9PI78_HUMAN | 39S ribosomal protein L49, mitochondrial | 0.76 | 4.79E-05^**^ |
| tr|Q8N4L6|Q8N4L6_HUMAN | ATP11B protein | 0.83 | 0.003985^**^ |
| tr|Q6ZRN6|Q6ZRN6_HUMAN | cDNA FLJ46220 fis, clone TESTI4013774 | 0.72 | 0.001376^**^ |
| tr|Q6GMX6|Q6GMX6_HUMAN | IGH@ protein | 0.75 | 8.17E-05^**^ |
| tr|A2JA14|A2JA14_HUMAN | Anti-mucin1 heavy chain variable region (Fragment) | 0.68 | 1.04E-08^**^ |
| tr|A8K2S1|A8K2S1_HUMAN | cDNA FLJ75258, highly similar to Homo sapiens phospholipase A2, group IVA (cytosolic, calcium-dependent) (PLA2G4A), mRNA | 0.82 | 1.98E-05^**^ |
| sp|Q8NB15|ZN511_HUMAN | Zinc finger protein 511 | 0.83 | 0.001744^**^ |
| tr|H0Y6F2|H0Y6F2_HUMAN | Proline-rich protein 5 (Fragment) | 0.82 | 7.99E-05^**^ |
| tr|D6RGE2|D6RGE2_HUMAN | Isochorismatase domain-containing protein 1 (Fragment) | 0.74 | 6.40E-08^**^ |
| tr|Q7Z6G4|Q7Z6G4_HUMAN | HBA2 (Fragment) | 0.76 | 4.45E-08^**^ |
| tr|M0QX44|M0QX44_HUMAN | Amino acid transporter (Fragment) | 0.52 | 1.98E-06^**^ |
| tr|B4DF70|B4DF70_HUMAN | cDNA FLJ60461, highly similar to Peroxiredoxin-2 (EC 1.11.1.15) | 0.83 | 0.01257^**^ |
| tr|B4E318|B4E318_HUMAN | BRCA2 and CDKN1A-interacting protein | 0.68 | 0.0002868^**^ |
| tr|H7C169|H7C169_HUMAN | COMM domain-containing protein 1 (Fragment) | 0.79 | 0.0002884^**^ |
| sp|P16452|EPB42_HUMAN | Erythrocyte membrane protein band 4.2 | 0.74 | 1.49E-06^**^ |
| sp|Q9H1A4|APC1_HUMAN | Anaphase-promoting complex subunit 1 | 0.76 | 4.98E-07^**^ |
| sp|Q9UJV8|PURG_HUMAN | Purine-rich element-binding protein gamma | 0.54 | 3.24E-07^**^ |
| tr|G3V1P3|G3V1P3_HUMAN | Loss of heterozygosity 12 chromosomal region 1 protein | 0.83 | 4.09E-05^**^ |
| tr|Q0PNF2|Q0PNF2_HUMAN | FEX1 | 0.82 | 0.03528^**^ |
| tr|Q6PIL8|Q6PIL8_HUMAN | IGK@ protein | 0.79 | 0.03704^**^ |
| tr|E7EUT5|E7EUT5_HUMAN | Glyceraldehyde-3-phosphate dehydrogenase | 0.83 | 1.18E-08^**^ |
| tr|Q8TAS2|Q8TAS2_HUMAN | LOC645212 protein (Fragment) | 0.66 | 0.01655^**^ |
| sp|O75396|SC22B_HUMAN | Vesicle-trafficking protein SEC22b | 0.82 | 0.001834^**^ |
| tr|Q8N5Z7|Q8N5Z7_HUMAN | 60S ribosomal protein L6 | 0.75 | 3.11E-10^**^ |
| sp|Q15814|TBCC_HUMAN | Tubulin-specific chaperone C | 0.66 | 5.72E-07^**^ |
| tr|E5RJP9|E5RJP9_HUMAN | Centrosomal protein CEP57L1 (Fragment) | 0.55 | 2.08E-06^**^ |
| tr|A0A087WSY5|A0A087WSY5_HUMAN | Carboxypeptidase B2 | 0.77 | 0.0001167^**^ |
| sp|Q15813|TBCE_HUMAN | Tubulin-specific chaperone E | 0.83 | 0.003807^**^ |
| tr|Q53T47|Q53T47_HUMAN | Putative uncharacterized protein POLR2D (Fragment) | 0.79 | 0.0004975^**^ |
| tr|Q14484|Q14484_HUMAN | Beta-globin (Fragment) | 0.82 | 0.04372^**^ |
| tr|A0A024R5J9|A0A024R5J9_HUMAN | Ribosomal protein S6 kinase, 70kDa, polypeptide 2, isoform CRA_a | 0.82 | 0.04082^**^ |
| sp|O94919|ENDD1_HUMAN | Endonuclease domain-containing 1 protein | 0.67 | 2.33E-07^**^ |
| sp|P02730|B3AT_HUMAN | Band 3 anion transport protein | 0.62 | 4.37E-08^**^ |
| tr|K9JIK7|K9JIK7_HUMAN | Glycophorin A | 0.4 | 2.78E-09^**^ |
| tr|Q5T7U0|Q5T7U0_HUMAN | General transcription factor 3C polypeptide 5 (Fragment) | 0.83 | 3.63E-06^**^ |
| tr|Q3Y9I8|Q3Y9I8_HUMAN | Hemoglobin beta (Fragment) | 0.78 | 0.0007223^**^ |
| tr|A0A024QZR0|A0A024QZR0_HUMAN | Leucine rich repeat containing 20, isoform CRA_a | 0.83 | 0.01002^**^ |
| sp|Q96PD5|PGRP2_HUMAN | N-acetylmuramoyl-L-alanine amidase | 0.68 | 0.0003171^**^ |
| tr|B3KRI9|B3KRI9_HUMAN | cDNA FLJ34383 fis, clone HCHON1000015, highly similar to Four and a half LIM domains protein 1 | 0.81 | 8.88E-10^**^ |
| sp|Q96IC2|REXON_HUMAN | Exonuclease NEF-sp, isoform CRA_a | 0.75 | 1.36E-06^**^ |
| sp|P11586|C1TC_HUMAN | C-1-tetrahydrofolate synthase, cytoplasmic | 0.78 | 3.09E-06^**^ |
| sp|Q15185|TEBP_HUMAN | Prostaglandin E synthase 3 (Cytosolic), isoform CRA_a | 0.82 | 4.34E-05^**^ |
| tr|H0YIA2|H0YIA2_HUMAN | Serine dehydratase-like (Fragment) | 0.8 | 3.08E-06^**^ |
| tr|B4DWN1|B4DWN1_HUMAN | cDNA FLJ52285, highly similar to Vesicular integral-membrane protein VIP36 | 0.67 | 0.000222^**^ |
| tr|Q8TEJ7|Q8TEJ7_HUMAN | FLJ00198 protein (Fragment) | 0.83 | 6.80E-05^**^ |
| tr|Q86U25|Q86U25_HUMAN | Full-length cDNA clone CS0DA007YG23 of Neuroblastoma of Homo sapiens (human) | 0.79 | 0.001045^**^ |
| tr|L8E853|L8E853_HUMAN | von Willebrand factor | 0.76 | 1.92E-08^**^ |
| tr|B4DQG2|B4DQG2_HUMAN | cDNA FLJ55390, highly similar to Ran-binding protein 17 | 0.82 | 5.52E-06^**^ |
| tr|Q9NXK9|Q9NXK9_HUMAN | cDNA FLJ20187 fis, clone COLF0433 | 0.68 | 2.23E-06^**^ |
| sp|Q7Z2W4|ZCCHV_HUMAN | Zinc finger CCCH-type antiviral protein 1 | 0.79 | 8.55E-07^**^ |
| sp|P02100|HBE_HUMAN | Hemoglobin, epsilon 1 | 0.74 | 8.34E-05^**^ |
| tr|Q9UM85|Q9UM85_HUMAN | Beta-globin protein (Fragment) | 0.78 | 0.0007631^**^ |
| sp|P51843|NR0B1_HUMAN | Nuclear receptor DAX1 | 0.71 | 0.03306^**^ |
| sp|P07954|FUMH_HUMAN | Fumarate hydratase, mitochondrial | 0.83 | 0.01677^**^ |
| sp|O00507|USP9Y_HUMAN | Probable ubiquitin carboxyl-terminal hydrolase FAF-Y | 0.68 | 1.49E-05^**^ |
| sp|Q15744|CEBPE_HUMAN | CCAAT/enhancer-binding protein epsilon | 0.79 | 5.47E-09^**^ |
| tr|Q4VB86|Q4VB86_HUMAN | EPB41 protein | 0.81 | 7.20E-05^**^ |
| tr|H0YLI6|H0YLI6_HUMAN | Isocitrate dehydrogenase [NAD] subunit alpha, mitochondrial (Fragment) | 0.66 | 5.83E-07^**^ |
| sp|Q15126|PMVK_HUMAN | PMVK protein (Fragment) | 0.79 | 0.0004081^**^ |
| sp|Q9BUH6|CI142_HUMAN | Uncharacterized protein C9orf142 | 0.79 | ** |
| tr|H0UI76|H0UI76_HUMAN | Aminopeptidase-like 1, isoform CRA_d | 0.78 | 0.0004685^**^ |
| tr|Q6ZTF9|Q6ZTF9_HUMAN | cDNA FLJ44699 fis, clone BRACE3016020 | 0.82 | 0.0002223^**^ |
| tr|G3V2S6|G3V2S6_HUMAN | V-type proton ATPase subunit D | 0.81 | 6.63E-06^**^ |
| sp|P49427|UB2R1_HUMAN | Cell division cycle 34, isoform CRA_a | 0.75 | 9.13E-06^**^ |
| tr|A0A087WW77|A0A087WW77_HUMAN | Lethal(2) giant larvae protein homolog 1 | 0.82 | 0.02677^**^ |
| tr|B4DRD5|B4DRD5_HUMAN | cDNA FLJ50176, moderately similar to Homo sapiens SLD5 homolog (SLD5), mRNA | 0.68 | 0.0001294^**^ |
| tr|A0A087X0Q4|A0A087X0Q4_HUMAN | Protein IGKV2-40 | 0.72 | 0.0008425^**^ |
| tr|J3KRK5|J3KRK5_HUMAN | Serine hydroxymethyltransferase, cytosolic | 0.62 | 2.29E-07^**^ |
| tr|B3KPC7|B3KPC7_HUMAN | Actin-related protein 2/3 complex subunit 5 | 0.68 | 4.59E-05^**^ |
| tr|B4DPP8|B4DPP8_HUMAN | cDNA FLJ53075, highly similar to Kininogen-1 | 0.82 | 2.23E-06^**^ |
| sp|Q12792|TWF1_HUMAN | Twinfilin-1 | 0.78 | 0.0002217^**^ |
| tr|B7Z7V7|B7Z7V7_HUMAN | cDNA FLJ61481, highly similar to PAS domain-containing serine/threonine-protein kinase (EC 2.7.11.1) | 0.75 | 0.0006929^**^ |
| tr|H7BZM7|H7BZM7_HUMAN | Zinc finger protein ZPR1 (Fragment) | 0.8 | 0.002155^**^ |
| tr|H7C548|H7C548_HUMAN | Cadherin-related family member 3 (Fragment) | 0.73 | 1.25E-06^**^ |
| tr|M0R261|M0R261_HUMAN | 6-phosphogluconolactonase (Fragment) | 0.83 | 0.0001983^**^ |
| tr|H0YNE9|H0YNE9_HUMAN | Ras-related protein Rab-8B (Fragment) | 0.63 | 1.67E-07^**^ |
| tr|B2R944|B2R944_HUMAN | cDNA, FLJ94203, highly similar to Homo sapiens stomatin (EPB72)-like 3 (STOML3), mRNA | 0.76 | 0.0007453^**^ |
| tr|Q5TD07|Q5TD07_HUMAN | Ribosyldihydronicotinamide dehydrogenase [quinone] | 0.83 | 0.0128^**^ |
| tr|V9HW35|V9HW35_HUMAN | Epididymis secretory protein Li 55 | 0.83 | 3.27E-06^**^ |
| tr|C9JPH9|C9JPH9_HUMAN | Fascin (Fragment) | 0.66 | 0.0001784^**^ |
| tr|H7BZ93|H7BZ93_HUMAN | Histone-lysine N-methyltransferase SETD2 (Fragment) | 0.46 | 2.64E-06^**^ |
| tr|H0YC44|H0YC44_HUMAN | Tumor protein D52 (Fragment) | 0.83 | 0.0253^**^ |
| tr|J3QLD1|J3QLD1_HUMAN | MIF4G domain-containing protein (Fragment) | 0.83 | 6.45E-05^**^ |
| sp|Q9NZZ3|CHMP5_HUMAN | Charged multivesicular body protein 5 | 0.77 | 5.66E-08^**^ |
| sp|P10155|RO60_HUMAN | TROVE domain family, member 2, isoform CRA_b | 0.83 | 1.64E-05^**^ |
| tr|B3KVN0|B3KVN0_HUMAN | cDNA FLJ16785 fis, clone NT2RI2015342, highly similar to Solute carrier family 2, facilitated glucose transporter member 1 | 0.55 | 7.64E-09^**^ |
| tr|A0A068LKQ2|A0A068LKQ2_HUMAN | Ig heavy chain variable region (Fragment) | 0.78 | 0.001062^**^ |
| tr|E9PGT6|E9PGT6_HUMAN | COP9 signalosome complex subunit 8 | 0.81 | 0.0001103^**^ |
| tr|E9PBM9|E9PBM9_HUMAN | Neutral ceramidase soluble form | 0.75 | 0.007508^**^ |
| tr|A6NJA2|A6NJA2_HUMAN | Ubiquitin carboxyl-terminal hydrolase | 0.83 | 0.01464^**^ |
| tr|Q0VGA5|Q0VGA5_HUMAN | SARS protein | 0.82 | 2.91E-05^**^ |
| tr|A0A024RB10|A0A024RB10_HUMAN | Cyclin-dependent kinase 2, isoform CRA_a | 0.8 | 0.00112^**^ |
| tr|A0A075B7G8|A0A075B7G8_HUMAN | Amyloid beta A4 precursor protein-binding family B member 1 | 0.74 | 0.001318^**^ |
| tr|A2MYE2|A2MYE2_HUMAN | A30 protein (Fragment) | 0.78 | 4.34E-05^**^ |
| tr|Q5QPQ1|Q5QPQ1_HUMAN | Acyl-protein thioesterase 2 (Fragment) | 0.81 | 0.0006273^**^ |
| tr|C3W5P5|C3W5P5_HUMAN | TLR9 (Fragment) | 0.74 | 0.002435^**^ |
| tr|B3KQJ1|B3KQJ1_HUMAN | cDNA FLJ90539 fis, clone OVARC1000255, highly similar to Tyrosine-protein kinase SYK (EC 2.7.10.2) | 0.79 | 0.0005402^**^ |
| sp|Q12955|ANK3_HUMAN | Ankyrin-3 | 0.82 | 4.24E-05^**^ |
| sp|Q9P209|CEP72_HUMAN | Centrosomal protein of 72 kDa | 0.82 | 0.004922^**^ |
| tr|A0A087X0P6|A0A087X0P6_HUMAN | Protein IGKV2D-29 | 0.67 | 7.20E-08^**^ |
| tr|H7BYY1|H7BYY1_HUMAN | Tropomyosin 1 (Alpha), isoform CRA_m | 0.73 | 1.97E-07^**^ |
| tr|Q5T4K5|Q5T4K5_HUMAN | CREB-regulated transcription coactivator 2 | 0.81 | 0.000623^**^ |
| sp|Q13627|DYR1A_HUMAN | Dual specificity tyrosine-phosphorylation-regulated kinase 1A | 0.79 | 2.20E-05^**^ |
| tr|A0A024RAV4|A0A024RAV4_HUMAN | Cold shock domain protein A, isoform CRA_b | 0.78 | 1.37E-07^**^ |
| tr|I3L1Q9|I3L1Q9_HUMAN | G1/S-specific cyclin-E1 (Fragment) | 0.64 | 1.37E-06^**^ |
| sp|Q02413|DSG1_HUMAN | Desmoglein-1 | 0.59 | 9.15E-06^**^ |
| sp|Q8ND04|SMG8_HUMAN | Protein SMG8 | 0.83 | 0.008405^**^ |
| tr|F8VX22|F8VX22_HUMAN | Phosphofurin acidic cluster sorting protein 2 | 0.58 | 6.47E-05^**^ |
| sp|P48637|GSHB_HUMAN | Glutathione synthetase | 0.81 | 0.04668^**^ |
| tr|J3QR40|J3QR40_HUMAN | Plexin domain-containing protein 1 (Fragment) | 0.81 | 0.009855^**^ |
| sp|P78509|RELN_HUMAN | Reelin | 0.77 | 0.0001789^**^ |
| sp|P50238|CRIP1_HUMAN | Cysteine-rich protein 1 | 0.67 | 1.39E-08^**^ |
| tr|F8W020|F8W020_HUMAN | Nucleosome assembly protein 1-like 1 (Fragment) | 0.83 | 0.003057^**^ |
| tr|B7Z229|B7Z229_HUMAN | cDNA FLJ58057, highly similar to RING finger protein 14 | 0.8 | 1.84E-05^**^ |
| tr|B3KY29|B3KY29_HUMAN | cDNA FLJ46695 fis, clone TRACH3013043, highly similar to Homo sapiens poly (ADP-ribose) polymerase family, member 10 (PARP10), mRNA | 0.8 | 1.77E-08^**^ |
| tr|F4MH86|F4MH86_HUMAN | Ubiquitously transcribed tetratricopeptide repeat protein Y-linked transcript variant 166 | 0.82 | 0.0282^**^ |
| sp|P01699|LV101_HUMAN | Ig lambda chain V-I region VOR | 0.66 | 0.0006919^**^ |
| tr|G3V4X5|G3V4X5_HUMAN | Proteasome subunit alpha type-3 | 0.75 | 0.02966^**^ |
| sp|O43488|ARK72_HUMAN | Aldo-keto reductase family 7, member A2 (Aflatoxin aldehyde reductase) | 0.76 | 0.001938^**^ |
| sp|P61966|AP1S1_HUMAN | Adaptor-related protein complex 1, sigma 1 subunit, isoform CRA_b | 0.77 | 5.34E-06^**^ |
| tr|F8W118|F8W118_HUMAN | Nucleosome assembly protein 1-like 1 (Fragment) | 0.75 | 0.01442^**^ |
| tr|E9PHY5|E9PHY5_HUMAN | Band 4.1-like protein 2 | 0.81 | 1.58E-07^**^ |
| tr|B4DS85|B4DS85_HUMAN | cDNA FLJ56829, highly similar to Neurogenic differentiation factor 6 | 0.79 | 0.002573^**^ |
| sp|P53680|AP2S1_HUMAN | AP-2 complex subunit sigma | 0.77 | 3.29E-06^**^ |
| sp|Q00534|CDK6_HUMAN | Cyclin-dependent kinase 6 | 0.77 | 3.39E-05^**^ |
| tr|H3BSN6|H3BSN6_HUMAN | Lysine--tRNA ligase (Fragment) | 0.75 | 0.00111^**^ |
| sp|P01768|HV307_HUMAN | Ig heavy chain V-III region CAM | 0.81 | 0.001137^**^ |
| tr|J3KSR7|J3KSR7_HUMAN | U3 small nucleolar RNA-associated protein 18 homolog (Fragment) | 0.73 | 3.47E-07^**^ |
| tr|Q59EM6|Q59EM6_HUMAN | Internexin neuronal intermediate filament protein, alpha variant (Fragment) | 0.77 | 4.66E-05^**^ |
| tr|H3BT57|H3BT57_HUMAN | Protein PML | 0.73 | 0.0003172^**^ |
| tr|A0A024RBT8|A0A024RBT8_HUMAN | Coiled-coil domain containing 62, isoform CRA_a | 0.77 | 1.12E-07^**^ |
| tr|B3KRR1|B3KRR1_HUMAN | cDNA FLJ34725 fis, clone MESAN2005958, highly similar to RNA-binding protein Luc7-like 2 | 0.67 | 2.02E-10^**^ |

**P<0.05;**P<0.01*
